# Red Light-Based Dual Photoredox Strategy Resembling the Z-Scheme of Natural Photosynthesis

**DOI:** 10.1021/jacsau.2c00265

**Published:** 2022-06-10

**Authors:** Felix Glaser, Oliver S. Wenger

**Affiliations:** Department of Chemistry, University of Basel, St. Johanns-Ring 19, 4056 Basel, Switzerland

**Keywords:** photocatalysis, spectroscopy, mechanistic analysis, electron transfer, energy transfer

## Abstract

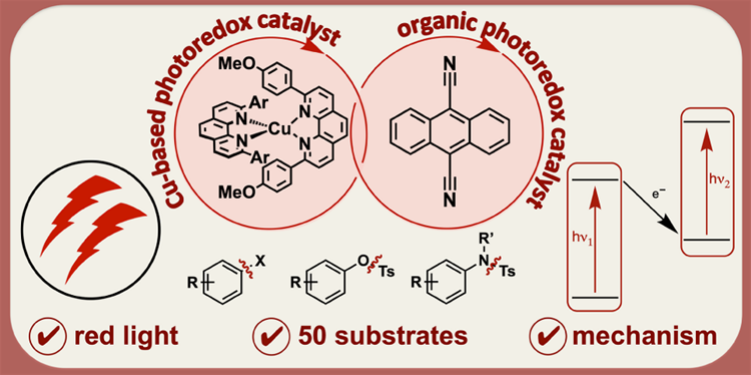

Photoredox catalysis typically relies on the use of single chromophores, whereas strategies, in which two different light absorbers are combined, are rare. In photosystems I and II of green plants, the two separate chromophores P_680_ and P_700_ both absorb light independently of one another, and then their excitation energy is combined in the so-called Z-scheme, to drive an overall reaction that is thermodynamically very demanding. Here, we adapt this concept to perform photoredox reactions on organic substrates with the combined energy input of two red photons instead of blue or UV light. Specifically, a Cu^I^ bis(α-diimine) complex in combination with *in situ* formed 9,10-dicyanoanthracenyl radical anion in the presence of excess diisopropylethylamine catalyzes ca. 50 dehalogenation and detosylation reactions. This dual photoredox approach seems useful because red light is less damaging and has a greater penetration depth than blue or UV radiation. UV–vis transient absorption spectroscopy reveals that the subtle change in solvent from acetonitrile to acetone induces a changeover in the reaction mechanism, involving either a dominant photoinduced electron transfer or a dominant triplet–triplet energy transfer pathway. Our study illustrates the mechanistic complexity in systems operating under multiphotonic excitation conditions, and it provides insights into how the competition between desirable and unwanted reaction steps can become more controllable.

## Introduction

**Figure 1:**
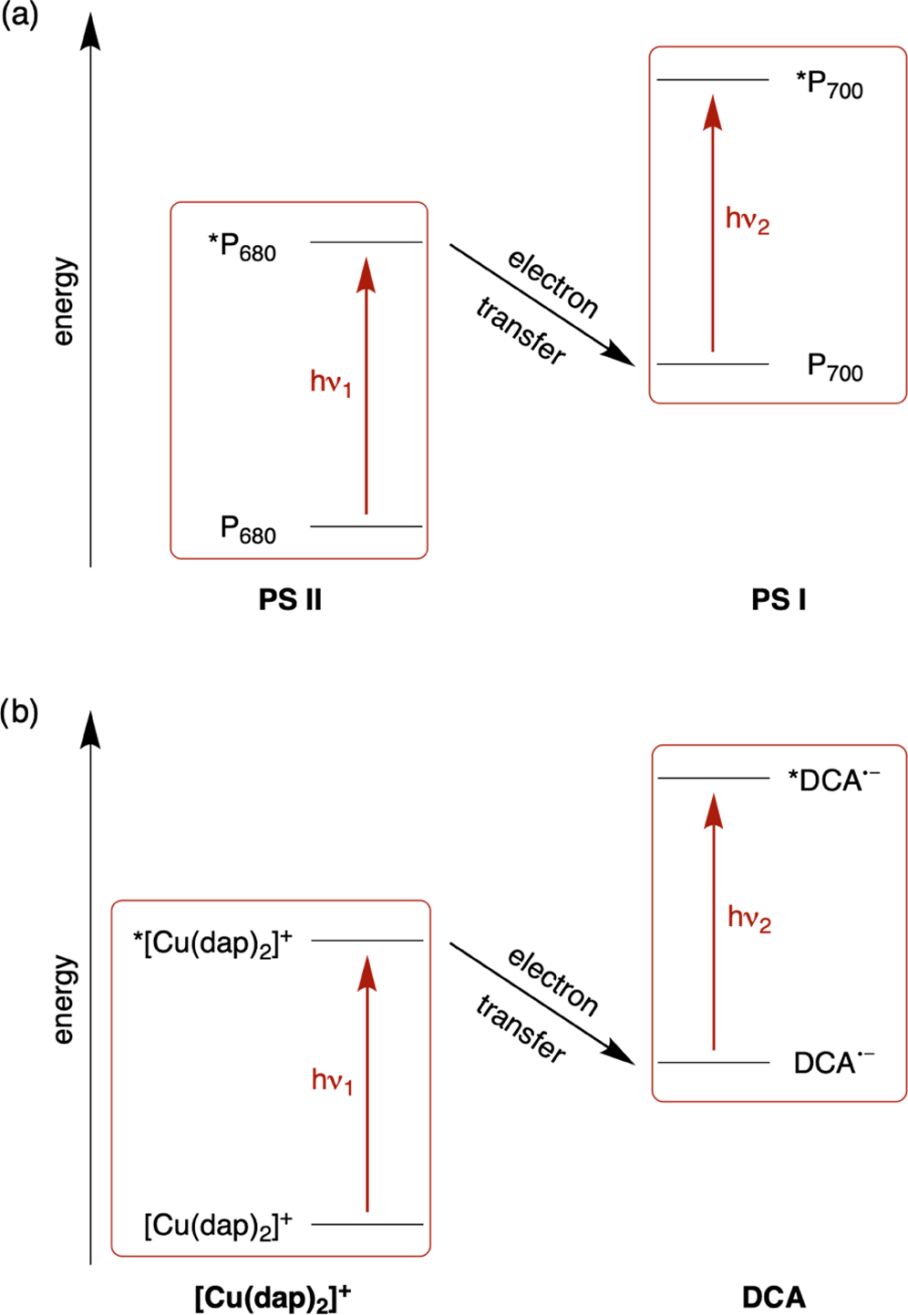
(a) Z-scheme of bacterial photosynthesis with the two key chromophores P_680_ of photosystem II (PS II) and P_700_ of photosystem I (PS I). Redox cofactors between P_680_ and P_700_ are not shown for simplicity. P_700_ is excited in its charge-neutral form and only accepts an electron from PS II once the photoexcited P_700_ (*P_700_) has been quenched oxidatively by the primary electron acceptor of PS I. (b) Combination of chromophores used herein for red light-driven photocatalysis. In a key mechanistic pathway, 9,10-dicyanoanthracene (DCA) is reduced by the photoexcited Cu^I^ complex (dap = 2,9-dianisyl-1,10-phenanthroline) and is then itself photoexcited.

Merging photoredox catalysis with other fields of chemistry has become increasingly popular over the past decade, including combinations with transition-metal catalysis,^1−7^ organocatalysis,^8−10^ biocatalysis,^11−14^ electrochemistry,^15−19^ or asymmetric catalysis.^20,21^ While an increasing number of photoredox strategies rely on biphotonic excitation involving the consecutive absorption of two (visible) photons, combinations of two independent photoactive catalysts are underexplored.^22,23^ In natural photosynthesis, two separate chlorophyll molecules called P_680_ and P_700_ absorb light with maxima in the red spectral range at 680 and 700 nm (Figure 1a), and their combined excitation energy is used to drive an overall reaction that would be unattainable based on the absorption of a single visible light quantum.^24^ The electron transfer chain and the photoexcitation events in photosystems I and II as drawn in Figure 1a resemble the letter Z, and consequently have been termed Z-scheme. In the context of artificial photosynthesis and photochemical water splitting, many researchers have made use of the Z-scheme strategy,^25,26^ but in synthetic organic photoredox catalysis, this approach seems underexplored yet. In the work presented herein, we have sought to apply the Z-scheme concept to an artificial photoredox system, operating based on low-energy input radiation (red light) to catalyze chemical reactions of organic molecules that would normally require blue or UV illumination. Our principal motivation was to explore to what extent the mimicry of a natural photoredox strategy can be applied in a useful manner to modern photocatalysis. From a more practical viewpoint, the use of a single photocatalyst absorbing blue or UV photons looks most straightforward at first glance, yet the consecutive absorption of two lower-energy photons can be advantageous because red or near-infrared light causes substantially less photodamage and has typically much greater penetration depth into colored solutions in reaction vessels.^27−29^ Red light furthermore provides an opportunity to excite photocatalysts more selectively, to prevent undesirable side reactions.^27−30^ On the other hand, red photons provide a significantly smaller amount of energy per photon than blue or UV photons: for instance, a blue photon with a wavelength (λ) of 410 nm carries an energy of 3.0 eV, while a red photon with a wavelength (λ) of 620 nm bears only 2.0 eV. Consequently, the synthetic opportunities for monophotonic applications with red light are considerably more limited than with blue light, due to the lower photonic energy.

Dual organic photoredox catalysis with two independent photocatalysts is very rare yet.^31,32^ Somewhat more common is the use of systems in which two closely related photocatalysts collaborate,^33,34^ including several examples where one of the two catalysts converts to the other under light irradiation.^35−39^ In those approaches, however, the photophysical and photochemical properties of the two catalysts cannot be optimized independently of each other, and a rational method development is only possible with limited degrees of freedom. When using biphotonic excitation strategies, typically a multitude of (productive and unproductive) mechanistic steps are viable,^40,41^ making the deliberate tuning of electron transfer, energy transfer, or triplet–triplet annihilation steps all the more desirable,^22^ and this is best possible in a system with two mutually independent photocatalysts. The exploration of this strategy seems relevant not only to accomplish new reactions that were so far unattainable with monophotonic excitation, but furthermore to perform known reactions that are thermodynamically demanding with lower-energy input light than previously possible.^22,31^

This latter aspect attracted our interest for the present study, in particular against the background of the recent surge of interest in red light-driven photocatalysis. As noted above, red and near-infrared photons have comparatively low energy content, and therefore monophotonic excitation strategies as used in the majority of studies performed with red light until now typically only permit the turnover of activated substrates.^27−30,42−50^

**Figure 2:**
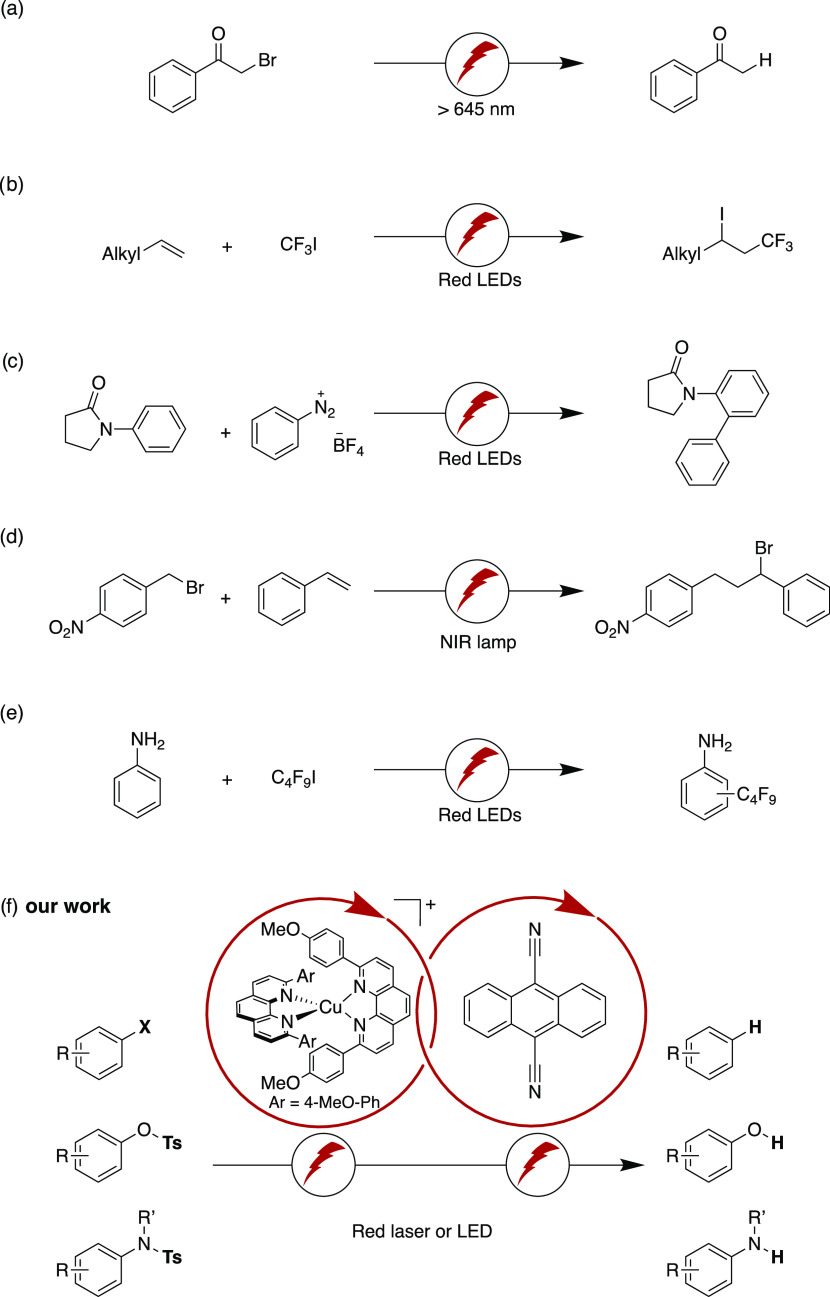
(a–e) Previously reported examples of red light-driven transformations.^28,42,43,45,52^ (f) Photocatalytic system used herein.

For instance, this includes α-brominated ketones (Figure 2a),^27,42^ the trifluoromethylation of alkenes based on CF_3_I (Figure 2b),^43,51^ the transition-metal co-catalyzed cross-coupling based on aryl diazonium salts (Figure 2c),^28^ an atom transfer radical addition (ATRA) reaction with benzyl bromide (Figure 2d),^52^ and the fluoroalkylation of aniline on the basis of C_4_F_9_I (Figure 2e).^45^ The examples in Figure 2 collectively illustrate the point that until now, red light has been mostly employed for activated substrates including α-functionalized ketones, polyfluorinated alkyl iodides, diazonium salts, and benzyl halides. Thus, the development of a biphotonic excitation strategy to push the limits of what is thermodynamically possible with red light seemed a worthy goal to us, in addition to demonstrating a new concept in photoredox catalysis.

Blue or green light-absorbing photocatalysts are widespread,^53−55^ whereas alternatives that feature sizeable extinction coefficients in the red spectral range are less common.^28,42−45,47−49,52^ Osmium polypyridyls are a well-known option,^28,56−62^ but in the spirit of our research program geared at the development of new photocatalysts based on Earth-abundant transition metals,^63−65^ Cu^I^ complexes attracted our attention. Many tetrahedral complexes of this type have long been known but received much attention for photoredox catalysis only recently.^10,66−74^ Most of them absorb predominantly in the blue or green,^72,75−81^ whereas the [Cu(dap)_2_]^+^ compound (dap = 2,9-dianisyl-1,10-phenanthroline, Figure 2f) stands out in its capacity to absorb up to ca. 650 nm.^70,82−84^ With its photoactive excited state storing 2.05 eV and an excited-state oxidation potential of −1.4 V *vs* SCE,^70,83,84^ [Cu(dap)_2_]^+^ looked like an attractive alternative to precious Os^II^ polypyridyls and was therefore chosen as the primary photocatalyst (Figure 1b).

The choice of the secondary photocatalyst was inspired by recent photoredox studies, in which radical anions (or their derivatives) were invoked as catalytically active species,^35,36,85−89^ including biphotonic as well as monophotonic (photoelectrochemical) excitation strategies.^16,17,19,90−95^ The radical anion of 9,10-dicyanoanthracene (DCA^•–^, Figure 1b) absorbs not only in the blue and green spectral ranges as exploited previously,^35^ but furthermore features prominent bands at 642 and 706 nm.^96^ Thus, we anticipated that a steady concentration of DCA^•–^ could be formed upon continuous red irradiation of [Cu(dap)_2_]^+^ in the presence of excess diisopropylethylamine, and that furthermore DCA^•–^ could be excited with the same light to reach a highly reactive excited state (Figure 1b). The respective doublet excited-state lifetimes of such anion radicals are usually in the subnanosecond time range and do not permit diffusion-controlled reactions.^97−102^ We speculated that photoreaction could nevertheless occur upon long-term irradiation, for example on the basis of preaggregated DCA^•–^ and substrates.^93,103−105^

Our study shows that this expectation is fulfilled for ca. 50 examples of dehalogenation and detosylation reactions, which typically require blue light as energy input. Transient absorption studies reveal an unanticipated complexity of the overall reaction mechanism, yet support the view of the Z-scheme-like process in Figure 1b as a key contributor.

## Results and Discussion

We started our investigation with fluorinated bromobenzonitrile (**1**) as substrate and initially employed a continuous-wave (cw) laser with an output wavelength of 635 nm and a power of 500 mW for the photoexcitation. Using 1 mol % of [Cu(dap)_2_]Cl along with 10 mol % of DCA (Table 1, entry 1), we determined a yield of 91% for product **1-P** in MeCN-*d*_3_ after illumination over 16 h. After 6 h of irradiation under these conditions, 79% of the starting material **1** was already consumed (entry 2). Since the cw laser irradiates a comparatively small area with high excitation density (due to its collimated beam), a high-power light-emitting diode (LED, 3.8 W) with output wavelengths centered at 623 nm was used to achieve a more homogeneous irradiation of the reaction vessel. This resulted in a yield of 86% for **1-P** after 6 h (entry 3), and therefore we continued our investigations with this light source. Analysis of the reaction progress over time (Supporting Information, Figure S1) revealed that the reaction dramatically slows once a conversion near 80–85% is reached. Screening of different additives (see the Supporting Information, Section 2.3.2) indicated that the addition of 0.5 equiv of cesium carbonate enables essentially complete conversion of the starting material **1** within 5 h (entry 4). This added salt can have diverse possible effects (see further discussion in Section 2.3.3 in the Supporting Information), but this was not investigated in detail. Control experiments in the absence of copper catalyst, DCA, sacrificial electron donor, or light resulted in no conversion of substrate **1** (entries 5–8).

**Table 1:**
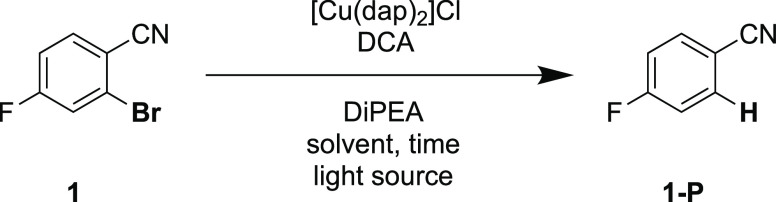
Optimization of a Photocatalytic Debromination Reaction with Red Light at 20 °C^a^

entry	[Cu(dap)_2_]Cl/mol %	DCA/mol %	solvent	additive	time/h	light source	yield (conv.)/%^b^
1	1	10	MeCN-*d*_3_	none	16	635 nm cw laser	91 (91)
2	1	10	MeCN-*d*_3_	none	6	635 nm cw laser	79 (79)
3	1	10	MeCN-*d*_3_	none	6	623 nm LED	86 (87)
4	1	10	MeCN-*d*_3_	0.5 equiv Cs_2_CO_3_	5	623 nm LED	95 (99)^c^
5	0	10	MeCN-*d*_3_	0.5 equiv Cs_2_CO_3_	5	623 nm LED	0 (0)
6	1	0	MeCN-*d*_3_	0.5 equiv Cs_2_CO_3_	6	623 nm LED	2 (2)
7	1	10	MeCN-*d*_3_	0.5 equiv Cs_2_CO_3_	6	623 nm LED	0 (0)^d^
8	1	10	MeCN-*d*_3_	0.5 equiv Cs_2_CO_3_	16	no light	0 (0)

a Reaction conditions: 25 mM substrate **1** and 20 equiv DiPEA (diisopropylethylamine) in 2 mL of deaerated MeCN-*d*_3_. Sample irradiated in a quartz cuvette under an argon atmosphere at 20 °C.

b Yields and conversions (in parentheses) were determined by quantitative ^19^F{^1^H}-NMR analysis using 4-fluorotoluene as the internal standard.

c Analysis of solutions under identical conditions in nondeuterated solvent on an analytical high-performance liquid chromatography (HPLC) setup as a complementary method gave a yield (conversion) of 95% (95%).

d Reaction performed in the absence of DiPEA.

**Figure 3:**
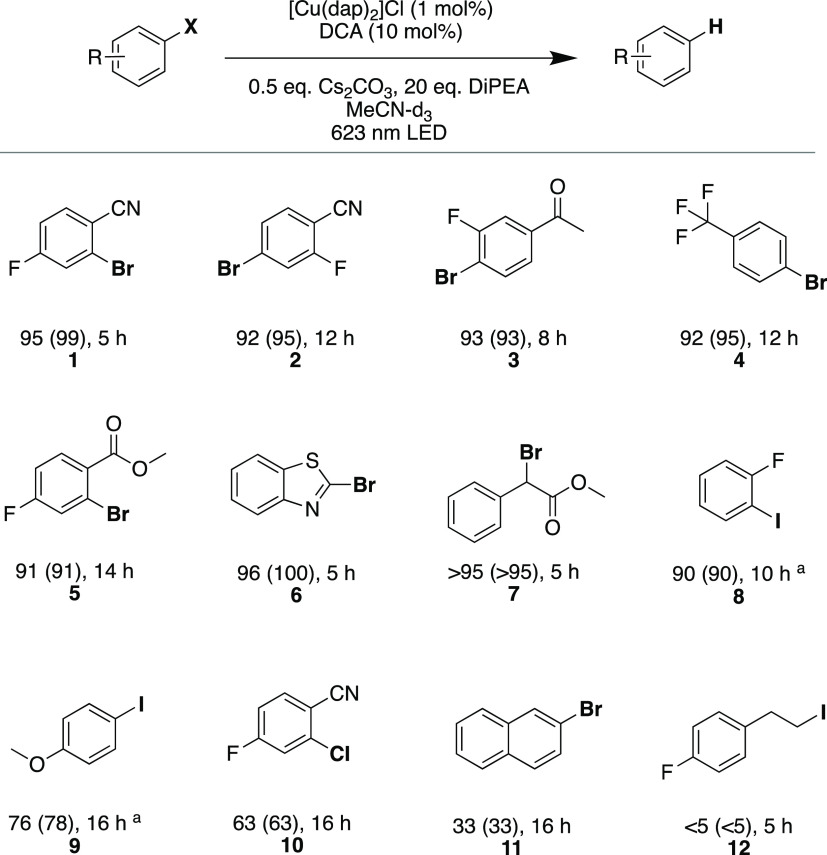
Hydrodehalogenation of selected aryl halides by red light-driven photoredox catalysis. Reaction conditions: 25 mM substrate, 1 mol % [Cu(dap)_2_]Cl, 10 mol % DCA, 0.5 equiv Cs_2_CO_3_, and 20 equiv DiPEA in 2 mL of MeCN-*d*_3_ irradiated with a 623 nm high-power LED (Thorlabs Solis-623C, 3.8 W) under argon at 20 °C. Yields and conversions (in parentheses) were determined by quantitative ^19^F{^1^H}-NMR or ^1^H-NMR analysis using 4-fluorotoluene or mesitylene as internal standards. ^a^Substrate concentration lowered to 20 mM.

With these optimized conditions, we then investigated different types of light-driven reactions. Dehalogenations of (activated) aryl halides (as a class of frequently used substrates for reductive transformations)^35,36,106,107^ served as first benchmark reactions. Reductive debrominations of aromatic substrates (Figure 3) such as benzonitriles (**1** and **2**), acetophenone (**3**), trifluormethylbenzene (**4**), benzoic ester (**5**), and benzothiazole (**6**) are readily possible in excellent yields. Furthermore, benzylic debromination (**7**) as well as deiodination of aryl substrates without (**8**) and with electron-donating substituents (**9**) are achievable with good to excellent yields. Unsurprisingly, for the more challenging dechlorination^108^ of activated aryl chloride (**10**) and the debromination of unactivated naphthyl bromide (**11**) only comparatively low conversions and yields were observed. Investigation of a substrate with an aliphatic iodide (**12**) furthermore demonstrated the limitation of our system with respect to reductive dehalogenation reactions. The lower yield despite longer irradiation times for aryl chlorides is in line with the reactivity observed in a previous study that used DCA and white light for carbon–carbon bond formation reactions between aryl radicals and suitable radical interceptors.^35^ For the light-induced reductive dehalogenation, that previous study reported similar performance as we observe for our [Cu(dap)_2_]Cl/DCA system. For example, the previous study reported a yield of 85% for 4-bromobenzonitrile in 5 h with white light,^35^ whereas we observe a yield of 95% with red light (Figure 3, substrate **1**). Based on this comparison, we expect a comparable substrate scope of aryl halides for carbon–carbon bond formation reactions with our catalytic system as in the previous study.

**Figure 4:**
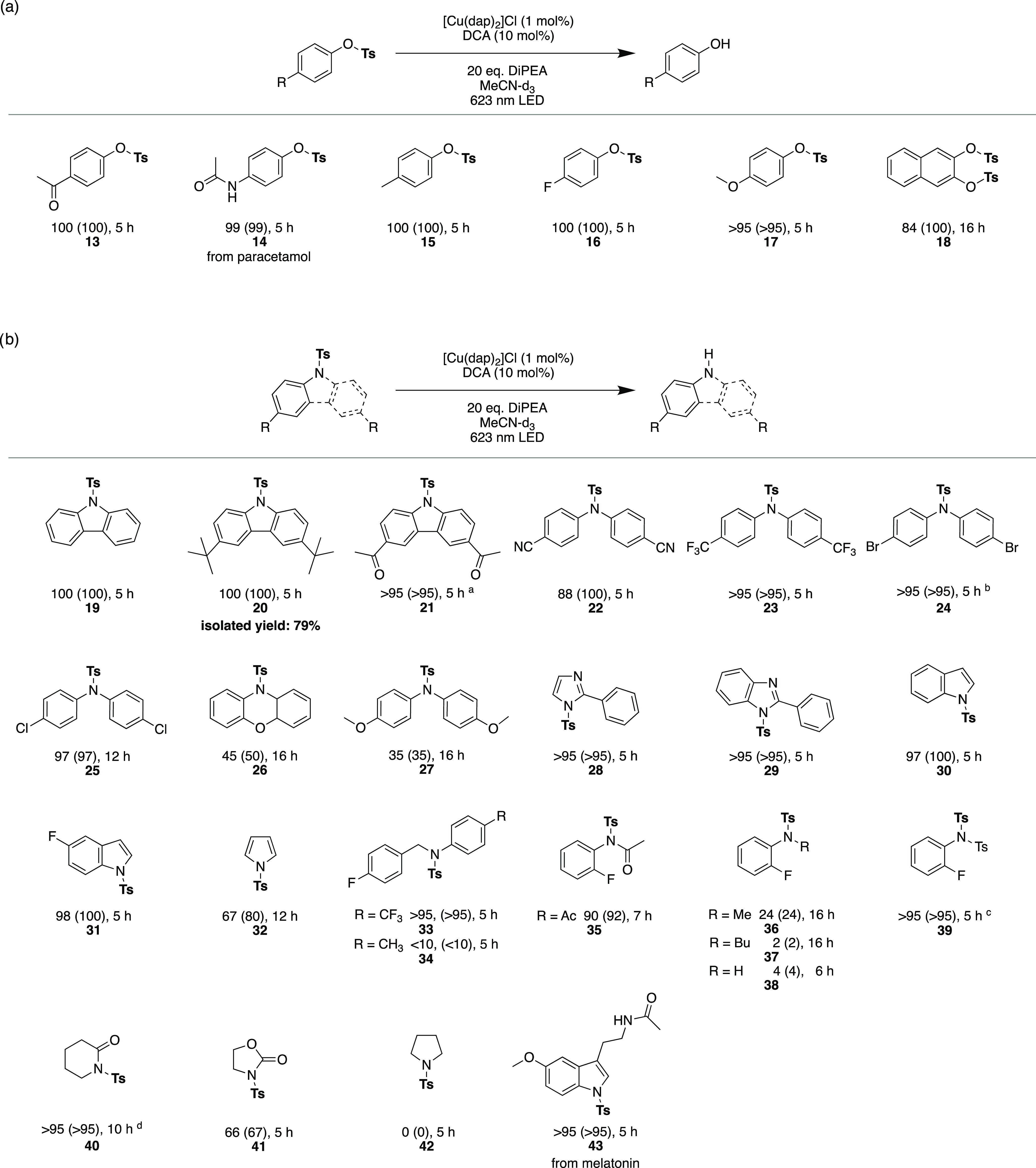
Red light-driven photoredox detosylation of protected phenolic (a) and nitrogen-containing substrates (b). Reaction conditions: 25 mM substrate, 1 mol % [Cu(dap)_2_]Cl, 10 mol % DCA and 20 equiv DiPEA in 2 mL of MeCN-*d*_3_ irradiated with a 623 nm high-power LED (Thorlabs Solis-623C, 3.8 W) under argon at 20 °C. Yields and conversions (in parentheses) were determined by quantitative ^19^F{^1^H}-NMR or ^1^H-NMR analyses using 4-fluorotoluene or mesitylene as internal standards. The experiment with substrate **20** and product isolation was performed on a 220 μmol scale. Further details are in the Supporting Information. ^a^Conditions changed to 10 mM substrate, 2 mol % [Cu(dap)_2_]Cl and 15 mol % DCA. ^b^Substrate concentration lowered to 20 mM. ^c^Only mono-detosylation observed. ^d^5 equiv of DiPEA used.

As a next class of reactions, we investigated detosylations of phenol substrates. Detosylations have recently been investigated with other photocatalytic systems, but typically blue light is needed for these reactions.^38,109−112^ All detosylation reactions of phenols with different electron-donating as well as electron-withdrawing substituents (**13**–**17**) resulted in very high conversions, and NMR yields above 95% within 5 h (Figure 4a). The double detosylation of naphthalene-diol **18** was possible in 84% yield, although a longer irradiation time of 16 h was needed. For the detosylation reactions explored here, full conversion was achievable without the addition of Cs_2_CO_3_, contrasting our findings above in the hydrodehalogenation reactions. This observation suggests that halide anions as leaving groups interfere with our catalytic system, whereas byproducts related to tosylates as leaving groups seem to be less problematic. Indeed, titration of bromide ions to [Cu(dap)_2_]Cl without irradiation suggested decreasing stability of this complex with increasing bromide concentration (see the Supporting Information, Section 4.3.5). Furthermore, anion-induced quenching of the excited state can have a significant impact on (unproductive) static excited-state deactivation of [Cu(dap)_2_]^+^ and could serve as a reasonable explanation for the observable reactivity depending on the leaving group and additive of the reaction.^113−116^ Due to the complexity of the overall system, further investigations regarding this aspect were not performed. A previous study of reductive dehalogenation with copper(I) photocatalysts did not observe a reactivity dependence on halide anions resulting from the dehalogenation reaction.^117^

In addition to protected phenols, tosylated nitrogen-containing groups furthermore attracted our attention^118^ as an additional class of compounds that might be suitable as substrates for energy-demanding red light-driven reduction reactions (Figure 4b). Experiments with unsubstituted (**19**) and *tert*-butyl-substituted carbazoles (**20**) resulted in excellent yields, and a carbazole with electron-withdrawing acetyl substituents (**21**) also worked very well. Furthermore, diarylamines with cyano (**22**), trifluormethyl (**23**), and bromo substituents (**24**) were successfully detosylated with excellent conversions and yields within 5 h, while a chlorinated analogue (**25**) needed an extended irradiation time of 12 h to achieve similar conversion and yield. Phenoxazine (**26)** and di(*p*-anisyl)amine (**27**) required significantly longer reaction times and only comparatively modest product yields were obtained even after 16 h of irradiation. Aromatic heterocycles such as imidazoles (**28**), benzimidazoles (**29**), and indoles (**30** and **31**) gave excellent yields, whereas tosylated pyrrole (**32**) was deprotected with a considerably lower yield of 67% within 12 h. The red light-driven detosylation reaction was furthermore extendable to substrates with nonaromatic substituents attached to the protected nitrogen atom. The direct comparison of benzyl anilines with a trifluoromethyl (**33**) and a methyl group (**34**) reveals that the electron-withdrawing trifluoromethyl-substituent is beneficial. A similar effect is seen for a tosylated acetamide (**35**), which reacts much better than more electron-rich methylated (**36**), butylated (**37**) analogues, or the primary aniline **38**. A twofold tosyl-protected aniline (**39**) reacted selectively to the mono-detosylated product with very good yields (in line with the observed poor reactivity of the mono-tosylated substrate **38**, which is the product of this reaction). Moving onward to nonaromatic ring structures further underscored that electron-withdrawing ketone functional groups, as in protected lactames **(40)** or oxazolidone (**41**), are beneficial for substrate activation. With purely aliphatic substrates such as pyrrolidone (**42**), no reaction occurred. Protected melatonin **(43**) was chosen as a representative example for a substrate bearing several functional groups, and excellent conversion and yield were achieved within 5 h in this case.

**Figure 5:**
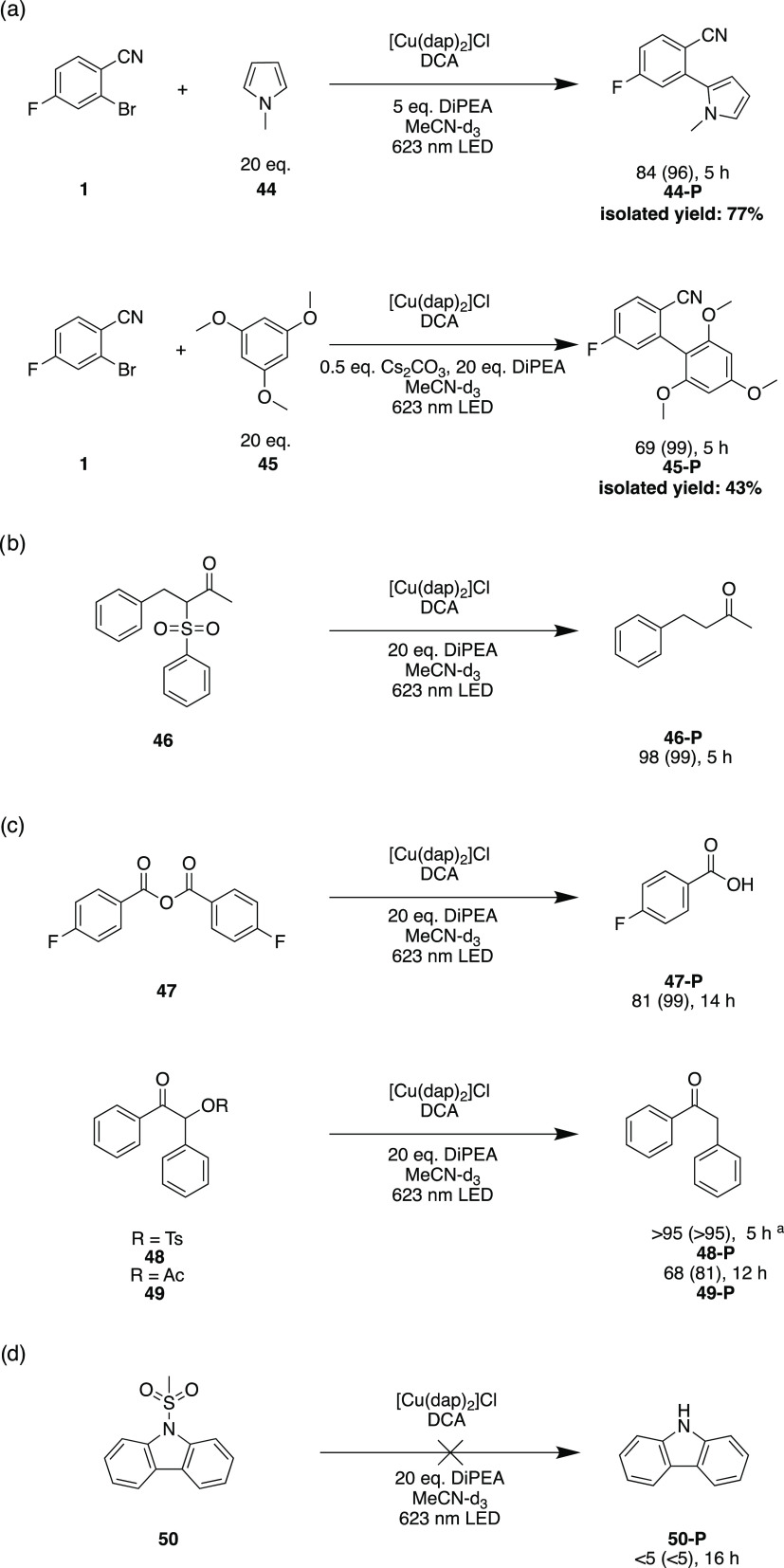
Investigations of carbon–carbon bond formation reactions (a); carbon–sulfur (b) and carbon–oxygen (c) bond cleavage reactions as well as an attempted demesylation reaction (d) using red light-driven photoredox catalysis. Reaction conditions: 25 mM substrate, 1 mol % [Cu(dap)_2_]Cl, 10 mol % DCA and 20 equiv DiPEA in 2 mL of MeCN-*d*_3_ irradiated with a 623 nm high-power LED (Thorlabs Solis-623C, 3.8 W) under argon at 20 °C. Yields and conversions (in parentheses) were determined by quantitative ^19^F{^1^H}-NMR or ^1^H-NMR analyses using 4-fluorotoluene or mesitylene as internal standards. The reactions, in which **44-P** and **45-P** were isolated, were performed on a 250 μmol scale. Further details are in the Supporting Information, Section 2.2. ^a^Substrate concentration lowered to 15 mM.

A preparative scale reaction was performed with substrate **20** on a 220 μmol scale (details in the Supporting Information, Section 2.2) and resulted in 79% of isolated product. Whilst the focus of our dehalogenations and detosylations was on the replacement of a functional or protective group by a hydrogen atom, the involved radical intermediates can in principle also be trapped by suitable radical interceptors.^35,119,120^ For example, *N*-methylpyrrole **44** and 1,3,5-trimethoxybenzene **45** were used successfully as aryl radical trapping reagents for substrate **1** with 84% yield of product **44-P** and 69% yield of product **45-P** (Figure 5a). Both of these reactions were furthermore performed on a 250 μmol scale, resulting in isolated product yields of 77 and 43%, respectively.

Finally, we concentrated on substrates with carbon–sulfur or carbon–oxygen bonds and explored the possibility of reductive C–S and C–O bond cleavage reactions (Figure 5b/c). While substrate **46** reacted smoothly within 5 h, the cleavage of an anhydride (**47**) needed prolonged irradiation times of 14 h. For protected benzoins, tosylates (**48**) as well as acetates (**49**) are both suitable leaving groups in our catalytic system (Figure 5c).^121^ It is worth mentioning that for substrates **48** and **49** a cleavage of the C–O bond is observed, unlike in the detosylation reactions of Figure 4, in which the O–S bonds are cleaved. In contrast to successful detosylations with carbazoles, a demesylation reaction (**50**) is not possible (Figure 5d).

## Mechanistic Investigations

In catalytic systems relying on biphotonic excitation, the elucidation of the reaction mechanism is often very challenging because a multitude of light-induced elementary steps are usually conceivable. This is the case for example in catalytic systems operating on the basis of triplet–triplet annihilation upconversion,^122,123^ consecutive photoinduced electron transfer mechanisms,^123^ or two-photon absorption pathways.^124^ Complete mechanistic study of catalytic systems with two photoactive species or with two (competing) reaction pathways is even more challenging.^125,126^ In exceptional cases tailor-made systems can give valuable insights into the biphotonic mechanisms,^32,127,128^ but this is not (readily) possible, or not investigated in studies with more synthetically oriented focus.^27,129^ In our catalytic system, [Cu(dap)_2_]^+^ absorbs up to 650 nm hence transient UV–vis absorption studies are only viable at longer wavelengths. The copper catalyst and DCA^•–^ absorb in the same range of the visible spectrum (see the Supporting Information, Section 4.1), and therefore selective excitation of either one of these two species in the presence of the other is not possible, thereby further complicating mechanistic studies by time-resolved laser spectroscopy. This imposes clear limits regarding the level of detail at which mechanistic investigations can be performed with our catalytic system.

**Figure 6:**
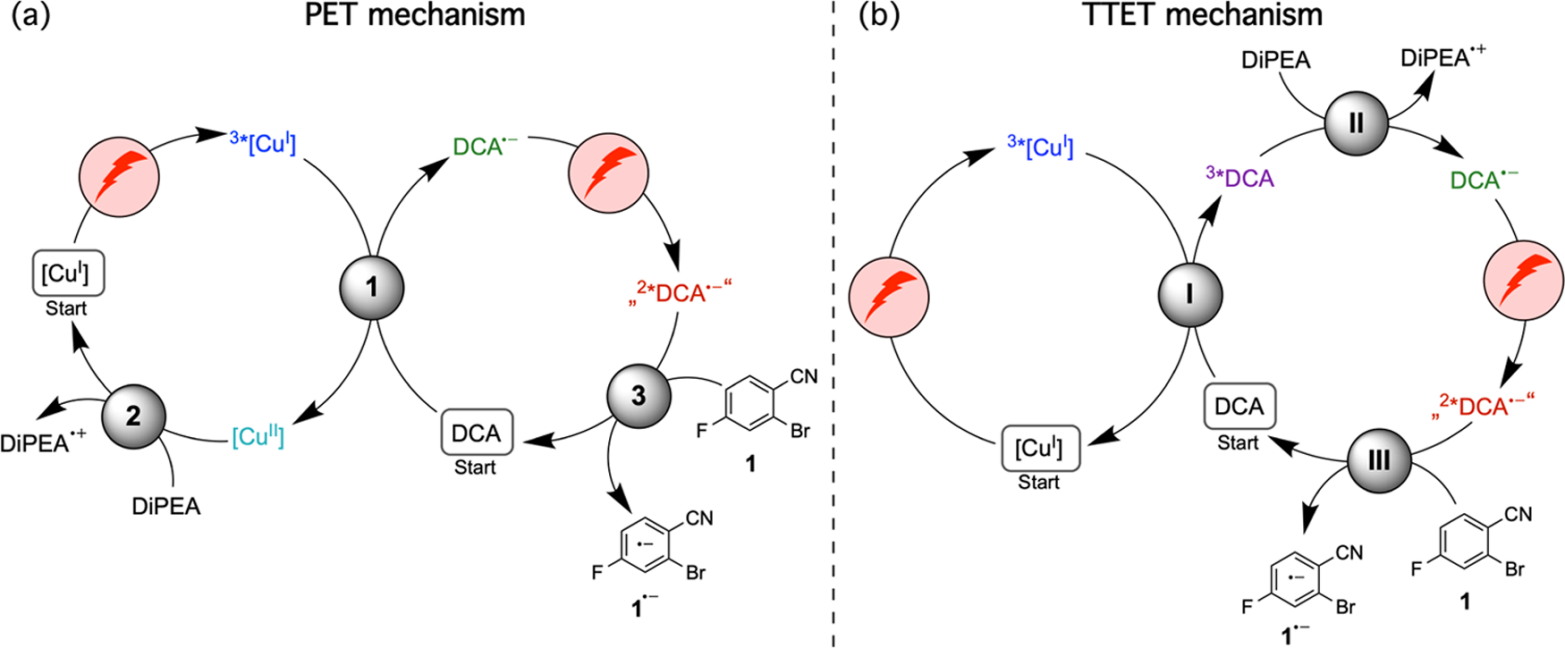
(a) Reaction mechanism based on an initial photoinduced electron transfer (PET) step between ^3^MLCT-excited [Cu(dap)_2_]Cl and 9,10-dicyanoanthracene (DCA). Gray circles mark the elementary reaction steps of (1) oxidative quenching of ^3*^[Cu(dap)_2_]^+^ (abbreviated as ^3*^[Cu^I^]) by DCA, (2) spontaneous reduction of the oxidized copper photocatalyst ([Cu^II^]) by DiPEA, and (3) substrate activation after excitation of the DCA radical anion. (b) Reaction mechanism based on an initial triplet–triplet energy transfer (TTET) step between ^3^MLCT-excited [Cu(dap)_2_]Cl and DCA. Gray circles mark the elementary reaction steps of (I) TTET, (II) reductive quenching of ^3*^DCA by DiPEA, and (III) substrate activation after excitation of DCA^•–^. The doublet excited state of that radical anion is extremely short-lived,^101^ and therefore, ^2*^DCA^•–^ is set in quotation marks, to emphasize the possibility that the photoreaction could in fact predominantly occur from preaggregated DCA^•–^/substrate encounter complexes, or could even involve some DCA photodegradation products.

The mechanistic proposal in Figure 1b involves photoinduced electron transfer (PET) from [Cu(dap)_2_]^+^ to DCA, and the DCA radical anion as photoexcitable species leading to substrate activation.^35,90,130^ However, when considering the photophysical and photochemical characteristics of the overall catalytic system, this is not a priori the only possible mechanistic interpretation of its observable photoreactivity. In the following, we discuss two different plausible mechanisms, one based on the above-mentioned initial photoinduced electron transfer step (PET mechanism in Figure 6a), and the other based on an initial triplet–triplet energy transfer (TTET) step between ^3^MLCT-excited [Cu(dap)_2_]^+^ and DCA (TTET mechanism in Figure 6b).

In both mechanisms, initially only [Cu(dap)_2_]Cl is excited because this is initially the only species absorbing the red cw laser or LED light (Figure S5). PET from ^3*^[Cu(dap)_2_]Cl to DCA (step 1 in Figure 6a) is exergonic by 0.5 eV (see the Supporting Information, Section 4.2.1); hence, DCA^•–^ should indeed be accessed directly. DiPEA could then reduce the oxidized copper complex ([Cu^II^]) back to its initial Cu^I^ form (step 2 in Figure 6a), to close the catalytic copper cycle.

An alternative pathway that could lead to the formation DCA^•–^ is a so-called sensitization-initiated electron transfer as presented in Figure 6b. This pathway must be considered because the initial TTET step from ^3*^[Cu(dap)_2_]Cl to DCA to yield ^3*^DCA (step I in Figure 6b) is exergonic by 0.25 eV.^83,84,131^ Depending on exact conditions (solvent, ionic strength), the initial (exergonic) PET and TTET elementary steps in Figure 6a/b will therefore compete directly with one another. Based on the triplet energy of DCA (1.8 eV) and its ground state reduction potential (−0.93 V *vs* SCE), an excited-state reduction potential of roughly 0.87 V *vs* SCE can be estimated,^132,133^ which should be sufficient to oxidize DiPEA (step II in Figure 6b).^134^ Consequently, if ^3*^DCA is formed, then onward reaction to DCA^•–^ via spontaneous electron transfer from DiPEA seems plausible.

Regardless of whether DCA^•–^ is formed directly via PET (Figure 6a) or via a sequence of TTET and electron donation from DiPEA (Figure 6b), the next productive step of the overall catalytic mechanism must excite DCA^•–^, ultimately leading to substrate activation (step 3 in Figure 6a and step III in Figure 6b) and completion of one catalytic turnover.

**Figure 7:**
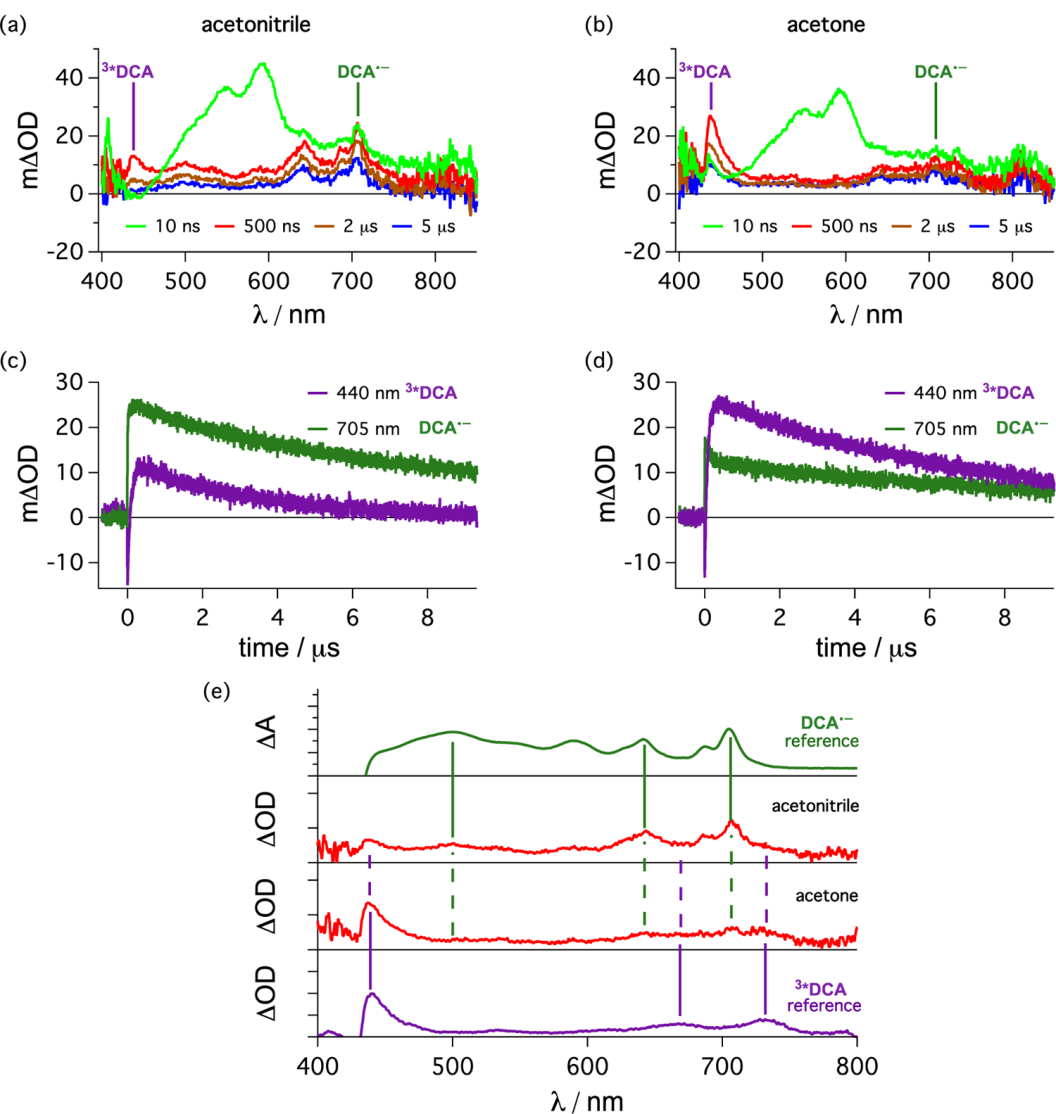
[Cu(dap)_2_]Cl (100 μM) in deaerated acetonitrile (a) and in deaerated acetone (b) was excited at 532 nm (30 mJ) in the presence of DCA (500 μM), and transient UV–vis absorption spectra were recorded with different time delays after the laser pulse (see insets), time-integrated over 200 ns. Kinetic traces over the first 9 μs after the laser pulse monitoring the transient absorption signals at 440 nm (main contribution from ^3*^DCA, violet traces) and 705 nm (main contribution from DCA^•–^, green traces) for the same solutions as in (a) and (b) are presented in (c) for acetonitrile and in (d) for acetone. A comparison of the spectral traces recorded with a delay of 500 ns from (a) and (b) in both solvents (red traces, middle) to the electrochemically generated DCA^•–^ reference in acetonitrile (top) and the transient signals of ^3*^DCA reference generated by energy transfer from [Ru(bpy)_3_]^2+^ in acetonitrile are presented in (e). Further details are provided in the text and in Section 4.1 in the Supporting Information.

Pulsed excitation of an acetonitrile solution of [Cu(dap)_2_]Cl (100 μM) at 532 nm in the presence of 500 μM of DCA (corresponding to the solubility limit) results initially in a dominant transient absorption band around 590 nm, corresponding to ^3*^[Cu(dap)_2_]^+^ (green trace in Figure 7a). At the same time, the characteristic spectroscopic features of the DCA radical anion with absorption maxima at 642 and 705 nm (the latter wavelength is marked by a vertical green line in Figure 7a) are already detectable. The same two absorption bands are observable in the UV–vis spectrum of electrochemically generated DCA^•–^ (top trace in Figure 7e). After longer time delays following the laser pulse (500 ns delay shown as a brown trace in Figure 7a), a (weak) new absorption band around 440 nm appears. Based on comparison to the literature and a reference experiment with sensitized TTET from [Ru(bpy)_3_]^2+^ (bottom trace in Figure S7e), the transient absorption band at 440 nm in Figure 7a/e is unambiguously attributable to the lowest triplet excited state of DCA (^3*^DCA).^135^ Evidently, both the PET (Figure 6a) and TTET (Figure 6b) mechanisms operate in acetonitrile, as suspected from the outset (see above).

To gain some semiquantitative insight into the relative importance of these PET and TTET mechanisms, it is useful to consider the kinetic traces in Figure 7c. Following excitation of [Cu(dap)_2_]^+^ at 532 nm and a time delay of 500 ns, ^3*^[Cu(dap)_2_]^+^ has largely disappeared and the remaining signals are predominantly due to DCA^•–^ (mΔOD = 23.5 at 705 nm) and ^3*^DCA (mΔOD = 10.1 at 440 nm). Assuming that the molar extinction coefficient of DCA^•–^ at 705 nm (ε_705_) is 8400 M^–1^ cm^–1^ (as reported previously),^96^ and further assuming that the molar extinction coefficient of ^3*^DCA at 440 nm (ε_440_) is 9000 M^–1^ cm^–1^,^135^ one estimates maximum concentrations of 2.80 μM for DCA^•–^ and 1.12 μM for ^3*^DCA. The simple comparison of these two concentrations suggests that the PET mechanism of Figure 6a contributes to roughly 70%, whereas the TTET mechanism of Figure 6b contributes to approximately 30% under these conditions in acetonitrile. This crude estimation is associated with considerable uncertainty, given the experimental limitations of the catalytic system and considering certain simplifications implicit to the above analysis (see the Supporting Information, Section 4.3 for details). However, the key point is that the PET mechanism (Figure 6a) is dominant, whereas the TTET is less relevant in acetonitrile.

Analogous experiments performed under the same conditions in acetone yielded the opposite result (Figure 7b/d). In this solvent, the maximum transient absorbance and consequently the concentration of DCA^•–^ (green trace detected at 705 nm) is clearly lower than that of ^3*^DCA (violet trace detected at 440 nm). A direct comparison to reference spectra is provided in Figure 7e, indicating a dominant energy transfer pathway and less contribution from direct electron transfer. Using the same analysis as described above, we estimate that the TTET mechanism now dominates with about 70% over the PET mechanism, which contributes with roughly 30% to the photoreaction of the excited copper complex. Thus, the data in Figure 7 illustrate that even subtle changes in the reaction conditions (here a change in solvent from acetonitrile to acetone) can lead to a change in the dominant mechanistic pathway.^136^ This does not lead to a drastic change in the observed overall chemical reactivity in our system (for dehalogenation reactions in acetone, see the Supporting Information, Table S1), presumably because both mechanisms of Figure 6 are similarly productive, as they both ultimately lead to DCA^•–^ as key species.

Based on steady-state and time-resolved luminescence quenching experiments (Figures S18–S20), the rate constant for the initial reaction step leading to deactivation of the ^3^MLCT-excited state of [Cu(dap)_2_]^+^ is on the order of ∼(6–7) × 10^9^ M^–1^ s^–1^ in both acetone and acetonitrile (details in the Supporting Information, Section 4). This rate constant is roughly a factor of 2 below the diffusion limit for bimolecular quenching in acetonitrile (2 × 10^10^ M^–1^ s^–1^) at 20 °C.^131^ Our luminescence quenching experiments with [Cu(dap)_2_]^+^ and DCA revealed a static component in addition to the dynamic quenching, suggesting that the PET and TTET elementary steps can also occur in preaggregated [Cu(dap)_2_]^+^/DCA adducts (see the Supporting Information, Section 4.3.2).^137^ A more detailed analysis of the rate for formation of DCA^•–^ and ^3*^DCA tentatively points toward a static quenching mechanism for the initial PET step, while for the TTET step a dynamic quenching step is detectable (Supporting Information, Section 4). Control experiments with substrate **1** or DiPEA as a quencher for excited [Cu(dap)_2_]^+^ result in rate constants of 7 × 10^6^ M^–1^ s^–1^ or even below 10^6^ M^–1^ s^–1^, respectively. For the synthetically relevant DiPEA (0.5 M) and substrate concentrations (25 mM), this results in pseudo-first-order rate constants of 5 × 10^5^ and ∼2 × 10^5^ s^–1^, both being substantially below the pseudo-first-order rate constant for the reaction of ^3*^[Cu(dap)_2_]^+^ with 0.5 mM DCA (∼(6–7) × 10^9^ M^–1^ s^–1^ × 0.0005 *M* = ∼(3–4) × 10^6^ s^–1^). This short analysis confirms that direct oxidative quenching by the substrate and reductive quenching by the sacrificial donor are not kinetically competitive with the PET and TTET steps in Figure 6.

In the PET mechanism, the copper catalyst is recovered with the sacrificial electron donor (step 2 in Figure 6a) while in the sensitized TTET mechanism [Cu(dap)_2_]^+^ is not redox-active and ^3*^DCA is quenched by DiPEA (step II in Figure 6b). In the following, we focus on these reactions with the sacrificial electron donor. By monitoring the kinetics of the UV–vis transient absorption signal associated with [Cu(dap)_2_]^2+^ at 380 nm (Supporting Information, Figure S26 and Section 4.3.3) as a function of DiPEA concentration in acetonitrile, a rate constant of ∼1 × 10^7^ M^–1^ s^–1^ is determined for electron transfer from DiPEA to [Cu(dap)_2_]^2+^. An analogous experiment monitoring the reduction of ^3*^DCA by DiPEA in acetone provided a rate constant of 2.5 × 10^6^ M^–1^ s^–1^ (Figure S25). Evidently, both of these rate constants are substantially below the diffusion limit, which likely reflects the fact they both occur with only small driving forces.

The last step of our proposed catalytic cycle is the substrate activation by ^2*^DCA^•–^ (step 3 in Figure 6a, and step III in Figure 6b). While earlier studies reported a lifetime in the range of several nanoseconds for ^2*^DCA^•–^,^138,139^ this was questioned later, and in particular the luminescence of ^2*^DCA^•–^ was doubted. Lifetimes on the picosecond timescale seem more realistic for ^2*^DCA^•–^,^101,140−142^ in line with the excited-state lifetimes reported for other radical anions.^97,102,143−145^ Furthermore, concerning the reactivity and stability of DCA^•–^, a variety of very different observations were reported in the literature, including claims of reasonably good stability of DCA^•–^,^146^ as well as observations of comparatively rapid degradation reactions with the solvent, reaction intermediates or oxygen.^140,147−149^ Overall, the stability and reactivity of DCA^•–^ seem to be highly dependent on the actual conditions in solution, and it seems that these aspects are sometimes overlooked. In our catalytic system, it seems plausible that DCA^•–^ accumulates over time as a result of [Cu(dap)_2_]Cl irradiation in the presence of excess DiPEA. Consequently, after some time, the two proposed catalytic cycles in Figure 2f are effectively decoupled. Given the very short lifetime of ^2*^DCA^•–^ it seems furthermore plausible that preassociation between DCA^•–^ and substrate might play an important role in successful product formation,^103,150,151^ and we cannot rigorously exclude the possibility that some of its photodegradation products interfere in the overall mechanism.^99,102,141,149,152^ Under cw laser irradiation of [Cu(dap)_2_]^+^ at 635 nm with a power of 500 mW, our photocatalytic system exhibits reasonably good stability (Supporting Information, Section 4.3.5). Indirect analysis of the substrate activation step in our photocatalytic system revealed conversions of over 80% for substrates with reduction potentials below −2.3 V *vs* SCE and a notable decrease for substrates with more negative reduction potentials. This observation seems in good agreement with the estimated excited-state reduction potential of −2.6 V *vs* SCE for ^2*^DCA^•–^ (see the Supporting Information, Section 4.3.4 for details).^101^

In summary, the two mechanisms in Figure 6 contribute to different extents in different solvents, and furthermore, other photoactive species related to the copper complex or DCA could contribute to the overall reaction.^147−149^ One specific possibility not discussed here is for example triplet–triplet annihilation upconversion of ^3*^DCA^•–^ to yield ^1*^DCA^•–^, followed by reduction of the latter with DiPEA. The PET versus TTET competition illustrated in Figure 6 captures however the main essence of the [Cu(dap)_2_]Cl/DPA dual photoredox system.

## Summary and Conclusions

**Figure 8:**
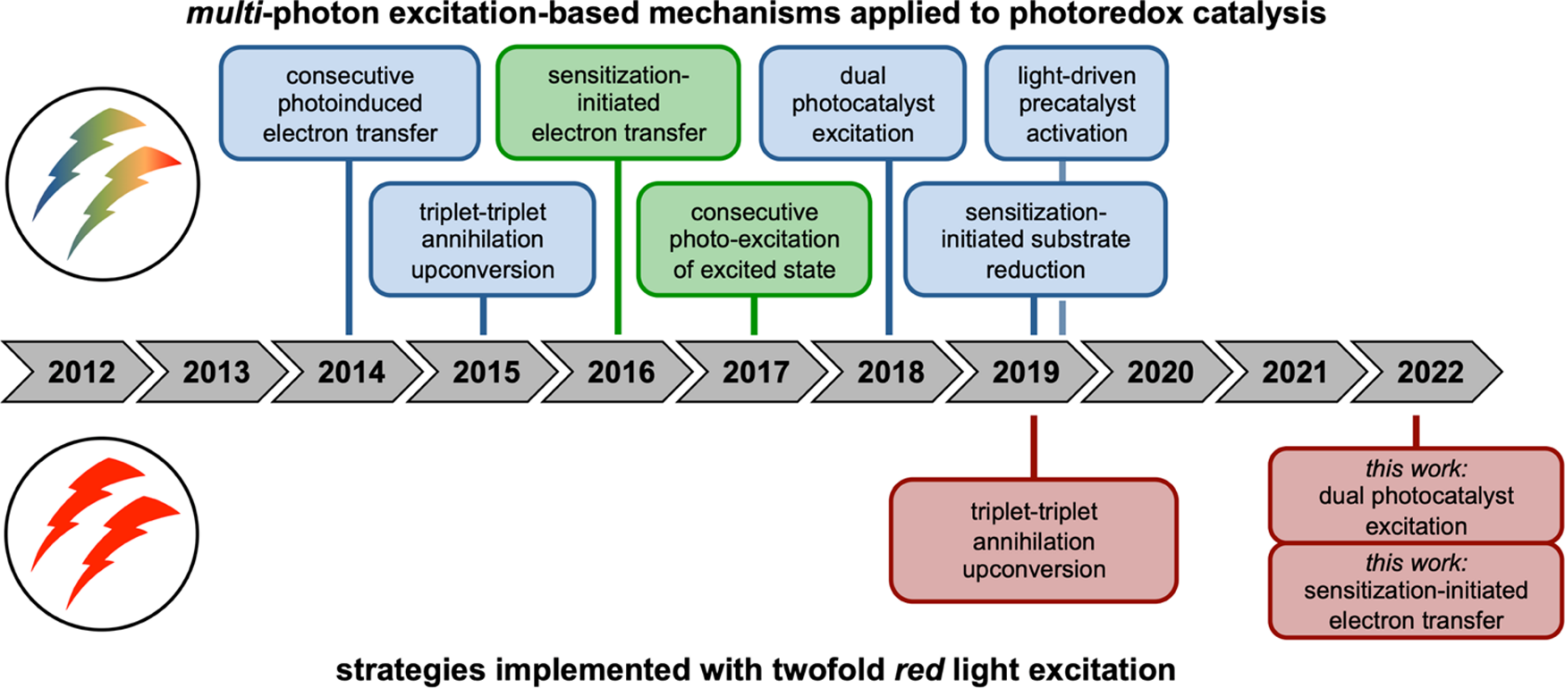
On a timeline, different multiphotonic mechanistic strategies for photoredox catalysis with visible light are assigned to the year within the last decade, in which they became popular (top) and when these were adapted to systems with red or near-IR light excitation (bottom).^22^ In the years 2014,^36^ 2015,^122^ 2016,^31^ 2017,^153^ 2018,^32^ and 2019,^33,126^ different mechanisms were reported with blue and green excitation light (background color classifies the excitation light color), while in 2019, an example with red to near-IR irradiation was reported.^27^

The concept of dual photocatalysis (Figure 1b), in which two different photoredox catalysts are combined, allows the use of red light for thermodynamically demanding reduction reactions. Roughly 50 examples of chemical transformations including dehalogenations of aryl halides, detosylations, as well as carbon–carbon bond formations illustrate the good catalytic performance of the [Cu(dap)_2_]Cl/DCA couple. Our approach of mimicking the Z-scheme of natural photosynthesis (Figure 1) pools the energy of two red photons, and consequently the scope of chemical transformations that can be driven by red light is considerably broadened beyond the current state of the art (Figure 2). Multiphoton excitation-based mechanisms that rely on red light are yet very rare (lower part of Figure 8),^27^ and most studies performed in this context until now relied on blue or green light (upper part of Figure 8).^22^

Mechanistic studies of the different reaction types in Figure 6 are particularly tricky because several different (competing) reaction pathways are usually opened up by multiphoton excitation.^111,120,154−156^ Our study illustrates this aspect quite clearly. In the initial photoinduced elementary step, electron transfer and triplet–triplet energy transfer compete with one another as seen unambiguously in transient absorption spectroscopy (Figure 7), and while the electron transfer process dominates in acetonitrile, triplet–triplet energy transfer becomes dominant in acetone. Since both of these elementary reaction steps ultimately lead to the formation of the key catalytic species (DCA^•–^), this does not affect the overall catalytic performance. For other photocatalytic systems, it is however conceivable that a subtle change of conditions activates unproductive or counterproductive reaction steps, and this could then drastically affect the reaction outcome and yield. Our study furthermore illustrates the point that a photoredox reaction does not necessarily follow a single mechanism, but that in fact multiple mechanisms can run in parallel and all contribute to product formation. The more complex the photocatalytic systems become, the more likely this probably gets.^136,157,158^

The combination of [Cu(dap)_2_]^+^ and DCA complements and expands the known photochemical applications of these two individual components when used separately.^68,77,83,89,117,159−167^ Red light-driven applications play important roles in other important contexts, for example, hydrogen production,^47,48,168,169^ medical applications,^158,169−173^ and polymerizations.^174−178^ Now, red light as well as multiphoton excitation-based mechanisms seem to become of increasing interest for synthetic organic photoredox chemistry,^50,126,179−181^ and we hope the insights gained from our work will be useful in that greater context.

## Supporting Information Available

Synthetic protocols and characterization data, description of methods and equipment, and additional spectroscopic and electrochemical data (PDF)

## Abbreviations

cw: continuous wave
Cu: copper
dap: 2,9-bis(4-methoxyphenyl)-1,10-phenanthroline
DCA: 9,10-dicyanoanthracene
DiPEA: diisopropylethylamine
PET: photoinduced electron transfer
LED: light-emitting diode
MeCN: acetonitrile
TTET: triplet–triplet energy transfer
