# Supplementary material for: Red Light-Based Dual Photoredox Strategy Resembling the Z-Scheme of Natural Photosynthesis
Source: JACS Au. 2022 Jun 10;2(6):1488–503. doi: 10.1021/jacsau.2c00265 (PMC9241018; doi:10.1021/jacsau.2c00265)
Supplement: Supplementary file 1 — au2c00265_si_001.pdf [file au2c00265_si_001.pdf]

Supporting Information for:

Red Light Based Dual Photoredox Strategy Resembling the Z-Scheme of Natural Photosynthesis

Felix Glaser and Oliver S. Wenger*

Department of Chemistry, University of Basel, St. Johanns-Ring 19, 4056 Basel, Switzerland

Table of Contents

1 General experimental details S3

2 Synthetic procedures and photoredox catalysis experiments S5

2.1 Synthetic procedures for substrates S5

2.1.1 General procedure 1 for tosylation of phenols S5

2.1.2 General procedure 2 for tosylation of anilines and amines S5

2.2 General procedures and isolated products S32

2.2.1 Photocatalytic reactions – general procedure S32

2.3 Reaction optimization S35

2.3.1 General optimization of reaction conditions S35

2.3.2 Conversion and yield over time S36

2.3.3 Effect of caesium carbonate S36

3 Irradiation setup S38

4 Optical spectroscopic measurements S39

4.1 Optical spectroscopic properties of $[Cu(dap)_2]Cl$ and DCA S39

4.1.1 Photophysical characterization of $[Cu(dap)_2]Cl$ and DCA S39

4.1.2 Transient absorption spectroscopy: reference spectrum of ^{3*}DCA S41

4.2 Cyclic voltammetry and spectro-electrochemical study S42

4.2.1 Cyclic voltammetry of catalysts S42

4.2.2 Spectro-electrochemical study of oxidized and reduced catalysts S43

4.2.3 Cyclic voltammetry of substrates S44

4.3 Mechanistic investigations S48

4.3.1 General overview S48

4.3.2 Formation of $DCA^{\bullet-}$ S49

4.3.3 Reduction of $[Cu(dap)_2]^{2+}$ by DiPEA S56

4.3.4 Substrate activation by $^{2*}DCA^{\bullet-}$: insights based on reactivity S57

4.3.5 Stability measurements: Photostability and UV-vis analysis S58

4.3.6 Proposed mechanism for detosylation reactions S60

5 NMR data S61

6 References S87

1 General experimental details

All deuterated solvents were purchased from Cambridge Isotope Laboratories Inc or Apollo Scientific. All reagents were purchased from Fluorochem, Alfar Aesar, Acros Organics or Sigma-Aldrich/Merck in "reagent grade" purity or better and were used as received. [Cu(dap)$_2$]Cl and DCA were purchased from Sigma-Aldrich/Merck. Solvents for spectroscopic measurements were purchased "extra dry" in 99.8% purity from Acros Organics.

NMR spectra were recorded on a Bruker Avance III instrument operating at 400 MHz proton frequency. All samples were recorded at 295 K in 5 mm diameter tubes. Chemical shifts were referenced internally to residual solvent peaks using δ values as reported by GOTTLIEB *et al.*[1] Starting material consumption and product formation were determined from ^{1}H-NMR-decoupled ^{19}F-NMR measurements (^{19}F{^{1}H}: 376 MHz, 16 scans) or ^{1}H-NMR measurements (^{1}H: 400 MHz, 16 scans) in NMR tubes against 4-fluorotoluene or mesitylene as internal standards. Elemental analyses were performed on a Vario Micro Cube instrument by Ms. Sylvie Mittelheisser (Department of Chemistry at the University of Basel).

Sample preparation for photoreduction reactions and spectrophotometric measurements were performed in screw cap quartz cuvettes. All solutions were purged with argon to remove oxygen and sealed under argon with septum caps. For photocatalytic reactions, a balloon with argon was installed during the time of the reaction.

Separation of reaction mixtures by analytical HPLC was carried out on a Shimadzu system (equipped with a SPD-M-20A photodiode array detector) on an Agilent ZORBAX XDB-C18 column (3.5 µm, XDB-C18, 75 x 4.6 mm, water:acetonitrile). After different times of irradiation, the reaction mixtures were diluted (10 × or 20 ×) with acetonitrile prior to HPLC analysis (buffer A: 0.1% formic acid (v/v) in ultrapure Millipor Milli-Q® water (specific resistance, 18.2 MΩcm); buffer B: 0.1% formic acid (v/v) in acetonitrile; gradient (expressed as percentage of buffer B in buffer A): 5% (1 min) → 35% (1.5 min) → 40% (8.5 min) → 99% (9 min) → 99% (10.5 min) → 5% (11.5 min) → 5% (13.5 min), flow rate 3 mL/min). Conversion and yield were calculated based on peak areas determined by integration at suitable wavelengths and calculated based on calibration curves (retention times: 4-fluorotoluene: 7.2 min, **1**: 4.9 min, **1-P**: 3.4 min).

Absorption spectra were recorded on a Cary 5000 UV-Vis-NIR spectrometer from Varian. Photoluminescence spectra were recorded on a Fluorolog-322 instrument from Horiba Jobin-Yvon. For laser flash photolysis, an LP920-KS apparatus from Edinburgh Instruments was used. A frequency-tripled Nd:YAG laser (Quantel Brilliant, ca. 10 ns pulse width) equipped with an OPO from Opotek and a beam expander (GBE02-A from Thorlabs) in the beam path were used for excitation with visible light. The direct output of another frequency-doubled Nd:YAG laser (Quantel Q-smart 450 mJ, ca. 10 ns pulse width) with a beam expander (GBE02-A from Thorlabs) in the beam path was used for excitation at 532 nm. The excitation energies were varied by the Q-switch delays and measured with a pyroelectric detector. Typically, pulse energies of ~50 mJ were used for the measurements with 532 nm. Detection of transient absorption and time-resolved emission spectra was performed with an iCCD camera (Andor). Kinetics at single wavelengths were recorded using a photomultiplier tube.

Cyclic voltammetry was performed using a Versastat3-200 potentiostat from Princeton Applied Research. A saturated calomel electrode (SCE) served as reference electrode, a glassy carbon disk electrode was employed as working electrode, and a silver wire was used as counter electrode. Measurements were performed with potential sweep rates of 100 mV/s in dry de-aerated solvent with 0.1 M $TBAPF_6$ (tetra-*n*-butylammonium hexafluorophosphate) as supporting electrolyte. Sample concentrations were adjusted to values between 1 mM and 5 mM of analyte.

Spectro-electrochemical measurements were performed in a quartz cuvette using the abovementioned potentiostat and the UV-Vis-NIR spectrometer. A platinum grid electrode served as working electrode, a platinum wire was used as counter electrode and an SCE was employed as reference electrode.

As light source for cw-laser experiments in photocatalysis, a 635 nm (optical output up to 500 mW) continuous wave (cw) laser (Roithner Lasertechnik) with precisely adjustable radiative power and high output stability (< 1%) was used. For measurements with a 623 nm LED, a high power LED (Thorlabs Solis-623C, 3.8 W) was used. Output spectra of the LED and cw laser are provided (Figure S5).

2 Synthetic procedures and photoredox catalysis experiments

2.1 Synthetic procedures for substrates

2.1.1 General procedure 1 for tosylation of phenols

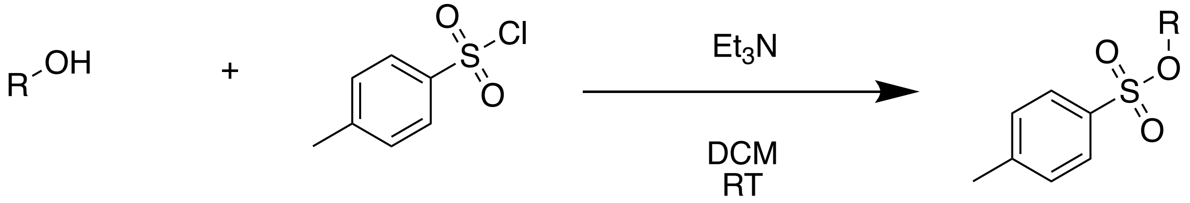

In a round-bottom flask the corresponding phenol (1 eq.) was dissolved in dichloromethane and triethylamine (1.5 eq.) and tosyl chloride (1.2 eq.) were added. The reaction mixture was stirred at room temperature and the reaction progress was monitored by TLC. After complete consumption of the substrate (typically 5-20 h), the reaction was quenched by the addition of water and extracted with dichloromethane (3 ×). The combined organic phases were dried over Na_2SO_4, the solvent was removed under reduced pressure and the crude mixture was purified by flash column chromatography to obtain the desired tosylated product.

2.1.2 General procedure 2 for tosylation of anilines and amines

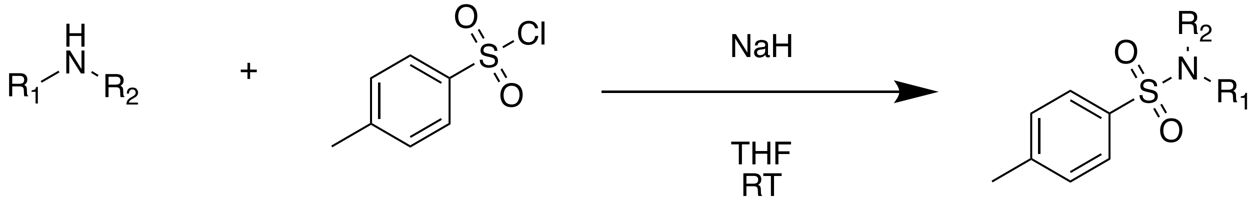

In a round-bottom flask the corresponding aniline (1 eq.) was dissolved in THF (10 mL for 1-2.5 mmol substrate) and NaH (60% in mineral oil, 2.5 eq.) and tosyl chloride (1.5 eq.) were slowly added. The reaction mixture was stirred at room temperature and the reaction progress was monitored by TLC. After complete consumption of the substrate (typically 5-20 h), the reaction was quenched by the addition of water and extracted with dichloromethane (3 ×). The combined organic phases were dried over Na_2SO_4, the solvent was removed under reduced pressure and the crude mixture was purified by flash column chromatography to obtain the desired tosylated product.

Methyl 2-bromo-4-fluorobenzoate (5)

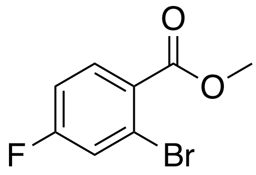

5

2-Bromo-4-fluorobenzoic acid (250 mg, 1.14 mmol, 1 eq.) and 1,8-diazabicyclo(5.4.0)undec-7-ene (255 µL, 1.71 mmol, 1.5 eq.) were dissolved in acetone (5 mL) and methyl iodide (1.44 µL, 2.28 mmol, 2.0 eq.) was added. The reaction mixture was stirred at room temperature for 16 h, the solvent was evaporated, aqueous HCl (1 M, 25 mL) was added and the mixture was extracted with ethyl acetate (3 × 30 mL). The combined organic phases were washed with brine, dried over Na_2SO_4 and the solvent was removed under reduced pressure. The crude mixture was purified by flash column chromatography (SiO_2, cyclohexane / ethyl acetate 1/0 → 4/1) to obtain the desired methyl 2-bromo-4-fluorobenzoate (**5**, 153 mg, 661 µmol, 58%) as white solid.

^{1}H-NMR (400 MHz, $CDCl_3$): δ 7.87 (dd, $J_{H,H}$ = 8.8 Hz, $J_{H,F}$ = 6.0 Hz, 1H), 7.40 (dd, $J_{H,F}$ = 8.3, $J_{H,H}$ = 2.5 Hz, 1H), 7.07 (ddd, $J_{H,H}$ = 8.9, $J_{H,F}$ = 7.6, $J_{H,H}$ = 2.5 Hz, 1H), 3.92 (s, 3H).

^{19}F{^{1}H}-NMR (376 MHz, $CDCl_3$): δ -105.76 (s, 1F).

^{13}C-NMR (101 MHz, $CDCl_3$): δ 164.0 (d, $^1J_{C,F}$ = 257.1 Hz), 133.5 (d, $^3J_{C,F}$ = 9.3 Hz), 128.1 (d, $^4J_{C,F}$ = 3.5 Hz), 123.3 (d, $^3J_{C,F}$ = 9.9 Hz), 122.0 (d, $^2J_{C,F}$ = 24.6 Hz), 114.7 (d, $^2J_{C,F}$ = 21.3 Hz), 52.6 ppm.

These analytical data are in agreement with previously reported characterization data for this compound.[2]

1-Fluoro-4-(2-iodoethyl)benzene (12)

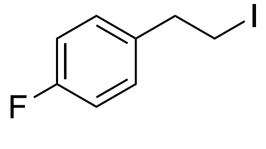

12

1-Fluoro-4-(2-bromoethyl)benzene (420 µL, 3.0 mmol, 1 eq.) was dissolved in acetone (10 mL), NaI (2.33 g, 15.5 mmol, 5.2 eq.) was added and the reaction mixture was refluxed at 60 °C for 16 h. The solvent was removed under reduced pressure and the crude mixture dissolved in dichloromethane and filtrated. The solvent was removed again under reduced pressure, and the crude mixture was purified by flash column chromatography (SiO_2, cyclohexane) to obtain the desired 1-fluoro-4-(2-iodoethyl)benzene (**12**, 604 mg, 2.42 mmol, 81%) as a colourless oil.

^{1}H-NMR (400 MHz, $CDCl_3$): δ 7.16 (dd, $J_{H,H}$ = 8.5, $J_{H,F}$ = 5.5 Hz, 1H), 7.01 (t, $J_{H,H;H,F}$ = 8.6 Hz, 1H), 3.33 (t, $^3J_{H,H}$ = 7.6 Hz, 1H), 3.15 (t, $^3J_{H,H}$ = 7.6 Hz, 1H) ppm.

^{19}F{^{1}H}-NMR (376 MHz, $CDCl_3$): δ -115.76 (s, 1F).

^{13}C-NMR (101 MHz, $CDCl_3$): δ 161.9 (d, $^{1}J_{C,H}$ = 245.1 Hz), 136.4 (d, $^{3}J_{C,H}$ = 3.3 Hz), 129.9 (dd, $^{3}J_{C,H}$ = 8.0 Hz), 115.6 (d, $^{2}J_{C,H}$ = 21.3 Hz), 39.5 (s), 5.9 (s) ppm.

These analytical data are in agreement with previously reported characterization data for this compound.[3]

4-Acetylphenyl 4-methylbenzenesulfonate (13)

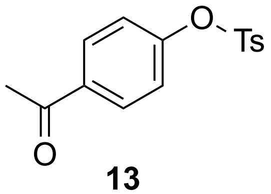

13

The general procedure 1 for tosylation using 4-hydroxyacetophenone (1.63 g, 7.49 mmol, 1.0 eq.) in dichloromethane (10 mL) yielded 4-acetylphenyl 4-methylbenzenesulfonate (**13**, 383 mg, 1.02 mol, 76%) as beige solid after flash column chromatography (SiO_2, cyclohexane / ethyl acetate 1/0 → 6/1).

^{1}H-NMR (400 MHz, $CDCl_3$): δ 7.90 (d, $^{3}J_{H,H}$ = 8.8 Hz, 1H), 7.71 (d, $^{3}J_{H,H}$ = 8.3 Hz, 2H), 7.33 (d, $^{3}J_{H,H}$ = 8.4 Hz, 2H), 7.09 (d, $^{3}J_{H,H}$ = 8.7 Hz, 1H), 2.58 (s, 3H), 2.46 (s, 3H) ppm.

^{13}C-NMR (101 MHz, $CDCl_3$): δ 196.8, 153.1, 145.9, 135.8, 132.2, 130.2, 130.1, 128.6, 122.6, 26.7, 21.9 ppm.

These analytical data are in agreement with previously reported characterization data for this compound.[4]

N-(4-hydroxyphenyl)acetamide (S1)

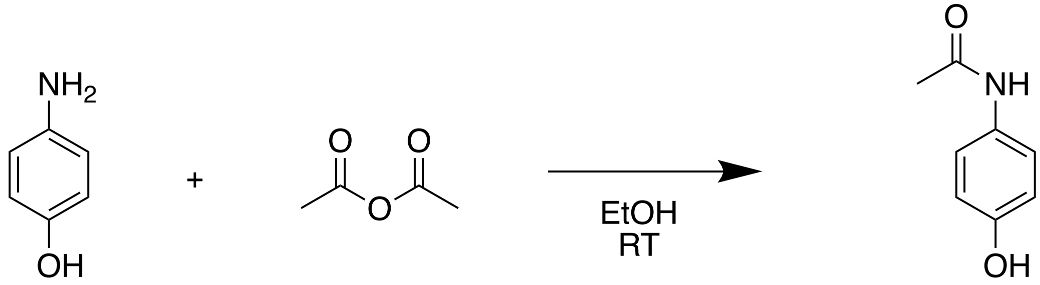

S1

4-Aminophenol (1.02 g, 9.35 mmol, 1.0 eq.) and acetic anhydride (950 µL, 9.81 mmol, 1.05 eq.) were dissolved in ethanol (20 mL) and stirred over night at room temperature. The solvent was evaporated under reduced pressure and the crude product was purified by flash column chromatography (SiO_2, cyclohexane), dispersed in hexane (15 mL). The solid was filtered off. The white solid was dried *in-vacuo* to obtain the desired *N*-(4-hydroxyphenyl)acetamide (**S1**, 1.31 g, 8.67 mmol, 93%).

^{1}**H-NMR** (400 MHz, acetone-d_6): δ 8.99 (s, 1H), 8.15 (s, 1H), 7.44 (d, $^3J_{H,H}$ = 8.9 Hz, 2H), 6.75 (d, $^3J_{H,H}$ = 8.9 Hz, 2H), 2.02 (s, 3H) ppm.

^{13}**C-NMR** (101 MHz, acetone-d_6): δ 168.2, 154.2, 132.7, 121.7, 115.9, 24.0 ppm.

These analytical data are in agreement with previously reported characterization data for this compound.[5]

4-Acetamidophenyl 4-methylbenzenesulfonate (14)

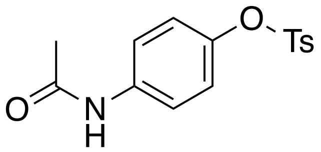

14

The general procedure 1 for tosylation using *N*-(4-hydroxyphenyl)acetamide (**S1**, 230 mg, 1.52 mmol, 1.0 eq.) in dichloromethane (10 mL) yielded 4-acetamidophenyl 4-methylbenzenesulfonate (**14**, 401 mg, 1.31 mol, 86%) as white solid after flash column chromatography (SiO_2, cyclohexane / ethyl acetate 4/1 → 1/1).

^{1}**H-NMR** (400 MHz, $CDCl_3$): δ 7.67 (d, $^3J_{H,H}$ = 8.2 Hz, 2H), 7.62 (s, 1H), 7.42 (d, $^3J_{H,H}$ = 9.0 Hz, 2H), 7.30 (d, $^3J_{H,H}$ = 8.2 Hz, 2H), 6.88 (d, $^3J_{H,H}$ = 9.0 Hz, 2H), 2.44 (s, 3H), 2.13 (s, 3H) ppm.

^{13}**C-NMR** (101 MHz, $CDCl_3$): δ 168.7, 145.7, 145.5, 137.1, 132.2, 129.9, 128.6, 123.0, 120.7, 24.6, 21.9 ppm.

These analytical data are in agreement with previously reported characterization data for this compound.[6]

p-Tolyl 4-methylbenzenesulfonate (15)

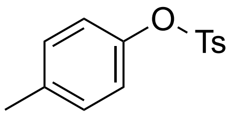

15

The general procedure 1 for tosylation using *p*-cresol (420 µL, 4.01 mmol, 1.0 eq.) in acetonitrile (20 mL) yielded *p*-tolyl 4-methylbenzenesulfonate (**15**, 1.02 g, 3.90 mol, 97%) as white solid after flash column chromatography (SiO_2, cyclohexane / ethyl acetate 1/0 → 6/1).

^{1}**H-NMR** (400 MHz, $CDCl_3$): δ 7.72 (d, $^3J_{H,H}$ = 8.4 Hz, 2H), 7.32 (d, $^3J_{H,H}$ = 8.1 Hz, 2H), 7.09 (d, $^3J_{H,H}$ = 8.4 Hz, 2H), 6.87 (d, $^3J_{H,H}$ = 8.6 Hz, 2H), 2.47 (s, 3H), 2.33 (s, 3H) ppm.

^{13}**C-NMR** (101 MHz, $CDCl_3$): δ 147.6, 145.3, 137.1, 132.6, 130.2, 129.8, 128.7, 122.2, 21.8, 21.0 ppm.

These analytical data are in agreement with previously reported characterization data for this compound.[7]

4-Fluorophenyl 4-methylbenzenesulfonate (16)

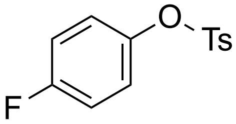

16

The general procedure 1 for tosylation using 4-fluorophenol (551 mg, 4.92 mmol, 1.0 eq.) in dichloromethane (20 mL) yielded 4-fluorophenyl 4-methylbenzenesulfonate (**16**, 851 mg, 3.20 mol, 65%) as white solid after flash column chromatography (SiO_2, cyclohexane / ethyl acetate 1/0 → 6/1).

^{1}H-NMR (400 MHz, $CDCl_3$): δ 7.71 (d, $^3J_{H,H}$ = 8.3 Hz, 1H), 7.34 (d, $^3J_{H,H}$ = 8.1 Hz, 2H), 7.02 – 6.94 (m, 4H), 2.48 (s, 3H) ppm.

^{19}F{^{1}H}-NMR (376 MHz, $CDCl_3$): δ -114.56 (s, 1F).

^{13}C-NMR (101 MHz, $CDCl_3$): δ 161.1 (d, $^1J_{C,F}$ = 246.6 Hz), 145.7, 145.5 (d, $^4J_{C,F}$ = 3.0 Hz), 132.2, 130.0, 128.7, 124.2 (d, $^3J_{C,F}$ = 8.8 Hz), 116.5 (d, $^2J_{C,F}$ = 23.7 Hz), 21.9 ppm.

These analytical data are in agreement with previously reported characterization data for this compound.[7]

4-Methoxyphenyl 4-methylbenzenesulfonate (17)

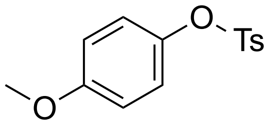

17

The general procedure 1 for tosylation using 4-methoxyphenol (490 mg, 3.95 mmol, 1.0 eq.) in dichloromethane (20 mL) yielded 4-methoxyphenyl 4-methylbenzenesulfonate (**17**, 996 mg, 3.58 mol, 91%) as white solid after flash column chromatography (SiO_2, cyclohexane / cyclohexane 9/1 → 1/1).

^{1}H-NMR (400 MHz, $CDCl_3$): δ 7.69 (d, $^3J_{H,H}$ = 8.4 Hz, 2H), 7.30 (d, $^3J_{H,H}$ = 8.5 Hz, 2H), 6.88 (d, $^3J_{H,H}$ = 9.2 Hz, 2H), 6.76 (d, $^3J_{H,H}$ = 9.1 Hz, 2H), 3.76 (s, 3H), 2.45 (s, 3H) ppm.

^{13}C-NMR (101 MHz, $CDCl_3$): δ 158.3, 145.3, 143.2, 132.5, 129.8, 128.7, 123.5, 114.6, 55.7, 21.9 ppm.

These analytical data are in agreement with previously reported characterization data for this compound.[7]

Naphthalene-2,3-diyl bis(4-methylbenzenesulfonate) (18)

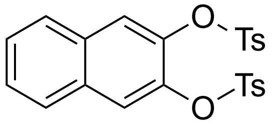

18

Naphthalene-2,3-diol (400 mg, 2.50 mmol, 1.0 eq.), sodium hydroxide (500 mg, 12.5 mmol, 5.0 eq.) and tosyl chloride (1.2 g, 6.29 mmol, 2.5 eq.) were dissolved in THF (15 mL) and stirred over night at room temperature. The reaction mixture was quenched by the addition of water (25 mL) and extracted with dichloromethane (3 × 40 mL). The combined organic phases were dried over Na_2SO_4 and the solvent was removed under reduced pressure. The crude mixture was purified by flash column chromatography (SiO_2, cyclohexane / ethyl acetate 1/0 → 20/1) and the product was recrystallized from hot ethanol / ethyl acetate (1:1) to obtain the desired naphthalene-2,3-diyl bis(4-methylbenzenesulfonate) (**18**, 695 mg, 1.48 mmol, 59%) as a white solid.

These analytical data are in agreement with previously reported characterization data for this compound.[8]

^{1}H-NMR (400 MHz, MeCN-d_3): δ 7.87 (dd, $J_{H,H}$ = 6.3 Hz, $J_{H,H}$ = 3.3 Hz, 2H), 7.70 (s, 2H), 7.65 (d, $J_{H,H}$ = 8.4 Hz, 4H), 7.59 (dd, $J_{H,H}$ = 6.3 Hz, $J_{H,H}$ = 3.2 Hz, 4H), 7.38 – 7.34 (m, 2H), 2.44 (d, J = 0.8 Hz, 3H) ppm.

^{13}C-NMR (101 MHz, MeCN-d_3): δ 147.5, 140.3, 132.9, 132.6, 131.0, 129.4, 128.7, 128.7, 123.3, 21.8 ppm.

Elemental analysis for $C_{24}H_{20}O_6S_2$ found (calculated): C 61.52 (61.52), H 4.35 (4.30), N 0 (0).

High resolution ESI-MS (m/z) for $C_{24}H_{20}O_6S_2$+Na [**18**+Na]$^+$: 491.0600 (calculated: 491.0594).

9-Tosyl-9H-carbazole (19)

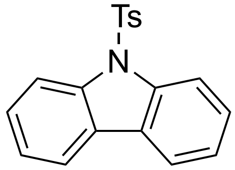

19

Carbazole (1.01 g, 6.04 mmol, 1 eq.) was dissolved in THF (25 mL) and KOH (1.20 g, 21.4 mmol, 3.5 eq.) and tosyl chloride (1.90 g, 10.0 mmol, 1.65 eq.) were slowly added. The reaction mixture was stirred at room temperature for 16 h, quenched by the addition of water (50 mL) and extracted with dichloromethane (3 × 50 mL). The combined organic phases were dried over Na_2SO_4 and the solvent was removed under reduced pressure. The crude mixture was purified by flash column chromatography (SiO_2, cyclohexane / dichloromethane 1/0 → 4/1) to obtain the desired 9-tosyl-9*H*-carbazole (**19**, 1.60 g, 4.98 mmol, 82%) as a white solid.

^{1}H-NMR (400 MHz, $CDCl_3$): δ 8.34 (d, $J_{H,H}$ = 8.4 Hz, 2H), 7.90 (d, $J_{H,H}$ = 7.7, 2H), 7.70 (d, $J_{H,H}$ = 8.4 Hz, 2H), 7.49 (dd, $J_{H,H}$ = 8.5 Hz, $J_{H,H}$ = 7.3 Hz, 2H), 7.36 (dd, $J_{H,H}$ = 7.7 Hz, $J_{H,H}$ = 7.3 Hz, 2H), 7.09 (d, $J_{H,H}$ = 8.4 Hz, 2H), 2.25 (s, 3H) ppm.

^{13}**C-NMR** (101 MHz, $CDCl_3$): δ 144.9, 138.4, 135.0, 129.7, 127.4, 126.5, 126.4, 123.9, 120.0, 115.2, 21.5 ppm. These analytical data are in agreement with previously reported characterization data for this compound.[9]

3,6-Di-tert-butyl-9-tosyl-9H-carbazole (20)

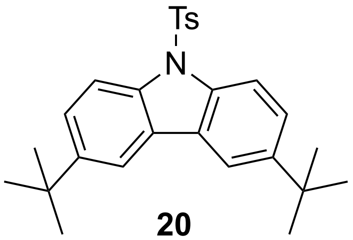

20

3,6-Di-*tert*-butyl-carbazole (550 mg, 1.98 mmol, 1 eq.) was dissolved in THF (10 mL) and KOH (280 mg, 4.92 mmol, 2.5 eq.) and tosyl chloride (560 mg, 2.95 mmol, 1.5 eq.) were slowly added. The reaction mixture was stirred at room temperature for 16 h, quenched by the addition of water (25 mL) and extracted with dichloromethane (3 × 40 mL). The combined organic phases were dried over Na_2SO_4, the solvent was removed under reduced pressure and the crude mixture was purified by flash column chromatography (SiO_2, cyclohexane / ethylacetate 1/0 → 3/1) to obtain the desired 3,6-di-*tert*-butyl-9-tosyl-9*H*-carbazole (**20**, 811 mg, 1.87 mmol, 95%) as an off-white solid.

^{1}**H-NMR** (400 MHz, $CDCl_3$): δ 8.20 (d, $J_{H,H}$ = 8.8 Hz, 2H), 7.90 (d, $J_{H,H}$ = 2.1 Hz, 2H), 7.72 (d, $J_{H,H}$ = 8.0 Hz, 2H), 7.51 (dd, $J_{H,H}$ = 8.8 Hz, $J_{H,H}$ = 2.0 Hz, 2H), 7.10 (d, $J_{H,H}$ = 8.0 Hz, 1H), 2.26 (s, 3H), 1.42 (s, 18H).ppm.

^{13}**C-NMR** (101 MHz, $CDCl_3$): δ 146.9, 144.6, 136.6, 135.3, 129.6, 126.6, 126.3, 124.9, 116.1, 114.5, 34.8, 31.7, 21.5 ppm.

Elemental analysis for $C_{27}H_{31}NO_2S$•0.05(CH_2Cl_2) found (calculated): C 74.13 (74.20), H 7.23 (7.16), N 3.22 (3.20).

High resolution ESI-MS (m/z) for $C_{27}H_{31}NO_2S$+Na [**20**+Na]$^+$: 456.1967 (calculated: 456.1968).

1,1'-(9H-Carbazole-3,6-diyl)bis(ethan-1-one) (S2)

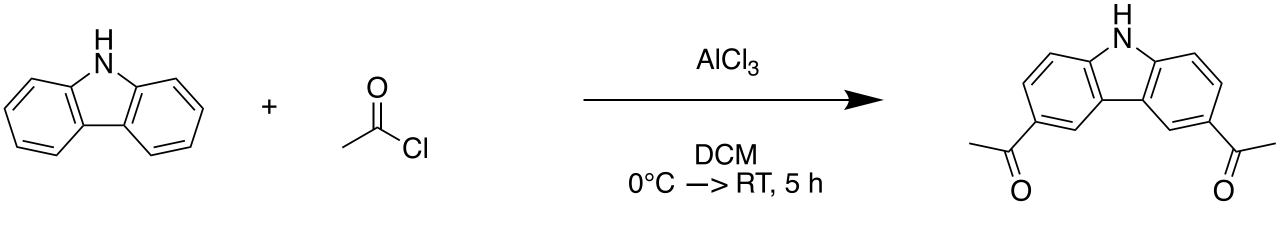

S2

Following a literature procedure,[10] carbazole (750 mg, 4.49 mmol, 1.0 eq.) was slowly added to a suspension of $AlCl_3$ (1.8 g, 13.5 mmol, 3.0 eq.) and acetyl chloride (1.43 mL, 20.2 mmol, 4.5 eq.) in DCM (25 mL) at 0 °C and

stirred for 5 h at room temperature. The reaction mixture was slowly poured into water (400 mL), extracted with DCM (3 × 200 mL) and the combined organic phases were dried over Na_2SO_4. The solvent was removed under reduced pressure and the crude mixture was purified by flash column chromatography (SiO_2, cyclohexane / ethyl acetate 1/0 → 0/1) to obtain 1,1'-(9*H*-carbazole-3,6-diyl)bis(ethan-1-one) (**S1**, 750 mg, 2.98 mmol, 67%) as a beige solid.

^{1}H-NMR (400 MHz, $CDCl_3$): δ 8.79 (d, $J_{H,H}$ = 1.7 Hz, 2H), 8.15 (dd, $J_{H,H}$ = 8.5 Hz, $J_{H,H}$ = 1.7 Hz, 2H), 7.51 (d, $J_{H,H}$ = 8.5 Hz, 2H), 2.75 (s, 6H) ppm.

^{13}C-NMR (101 MHz, $CDCl_3$): δ 197.7, 143.0, 130.5, 127.4, 123.5, 122.2, 111.0, 26.9 ppm.

These analytical data are in agreement with previously reported characterization data for this compound.[11]

1,1'-(9-Tosyl-9H-carbazole-3,6-diyl)bis(ethan-1-one) (21)

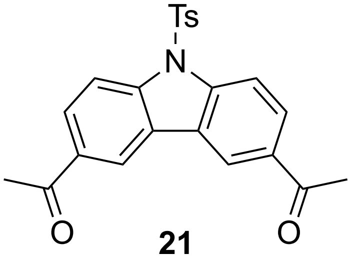

21

The general procedure 2 for tosylation using substrate **S2** (502 mg, 2.0 mmol, 1 eq.) in THF (15 mL) yielded 1,1'-(9-tosyl-9*H*-carbazole-3,6-diyl)bis(ethan-1-one) (**21**). The crude product was purified by flash column chromatography (SiO_2, cyclohexane / ethyl acetate 4/1) followed by recrystallization from ethanol to obtain **21** (380 mg, 938 µmol, 47%) as a white solid.

^{1}H-NMR (400 MHz, $CDCl_3$): δ 8.61 (d, $J_{H,H}$ = 1.8 Hz, 2H), 8.40 (d, $J_{H,H}$ = 8.8 Hz, 2H), 8.17 (dd, $J_{H,H}$ = 8.8 Hz, $J_{H,H}$ = 1.8 Hz, 2H), 7.74 (d, $J_{H,H}$ = 8.4 Hz, 2H), 7.16 (d, $J_{H,H}$ = 8.4 Hz, 2H), 2.72 (s, 6H), 2.30 (s, 3H) ppm.

^{13}C-NMR (101 MHz, $CDCl_3$): δ 197.0, 145.7, 141.6, 134.5, 133.3, 129.9, 128.3, 126.4, 125.8, 120.8, 114.76, 26.7, 21.5 ppm.

Elemental analysis for $C_{23}H_{19}NO_4S$ found (calculated): C 67.93 (68.13), H 4.72 (4.72), N 3.40 (3.45).

High resolution ESI-MS (m/z) for $(C_{23}H_{19}NO_4S)_2$+Na [(**21**)$_2$+Na]$^+$: 833.1957 (calculated: 833.1962).

4,4'-Azanediyldibenzonitrile (S3)

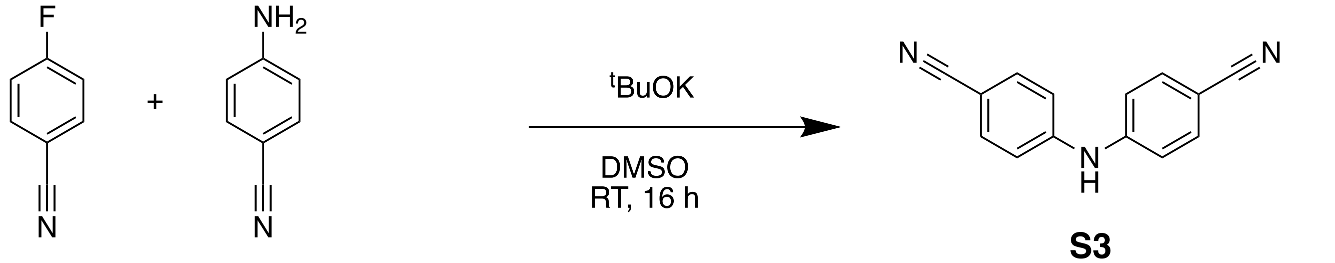

S3

4-Aminobenzonitrile (590 mg, 4.99 mmol, 1.0 eq.), 4-fluorobenzonitrile (644 mg, 5.32 mmol, 1.05 eq.) and potassium *tert*-butoxide (1.12 g, 10.0 mmol, 2.0 eq.) were dissolved in DMSO (15 mL) and stirred over night at room temperature. The reaction mixture was poured into water (400 mL), and the precipitate filtered off. The crude product was purified by recrystallization from ethanol to obtain 4,4'-azanediyldibenzonitrile (**S2**, 575 mg, 2.62 mmol, 52%) as a pale red solid.

^{1}H-NMR (400 MHz, $CDCl_3$): δ 7.59 (d, $J_{H,H}$ = 8.8 Hz, 4H), 7.15 (d, $J_{H,H}$ = 8.8 Hz, 4H), 6.34 (s, 1H) ppm.

^{13}C-NMR (101 MHz, $CDCl_3$): δ 144.9, 133.9, 119.0, 117.8, 105.0 ppm.

These analytical data are in agreement with previously reported characterization data for this compound.[12]

N,N-Bis(4-cyanophenyl)-4-methylbenzenesulfonamide (22)

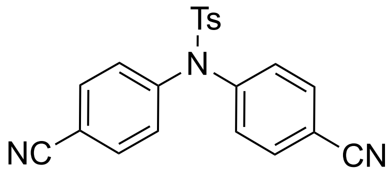

22

The general procedure 2 for tosylation using substrate **S3** (295 mg, 1.35 mmol, 1.0 eq.) in THF (10 mL) yielded *N,N*-bis(4-cyanophenyl)-4-methylbenzenesulfonamide (**22**, 383 mg, 1.02 mol, 76%) as beige solid after flash column chromatography (SiO_2, cyclohexane / ethyl acetate 1/0 → 2/1).

^{1}H-NMR (400 MHz, $CDCl_3$): δ 8.61 (d, $J_{H,H}$ = 1.8 Hz, 2H), 8.40 (d, $J_{H,H}$ = 8.8 Hz, 2H), 8.17 (dd, $J_{H,H}$ = 8.8 Hz, $J_{H,H}$ = 1.8 Hz, 2H), 7.74 (d, $J_{H,H}$ = 8.4 Hz, 2H), 7.16 (d, $J_{H,H}$ = 8.4 Hz, 2H), 2.72 (s, 6H), 2.30 (s, 3H) ppm.

^{13}C-NMR (101 MHz, $CDCl_3$): δ 197.0, 145.7, 141.6, 134.5, 133.3, 129.9, 128.3, 126.4, 125.8, 120.8, 114.8, 26.7, 21.5 ppm.

Elemental analysis for $C_{21}H_{15}N_3O_2S$ found (calculated): C 67.52 (67.54), H 4.13 (4.05), N 11.25 (11.25).

High resolution ESI-MS (m/z) for $C_{21}H_{15}N_3O_2S$+Na [**22**+Na]$^+$: 396.0772 (calculated: 396.0777).

Bis(4-(trifluoromethyl)phenyl)amine (S4)

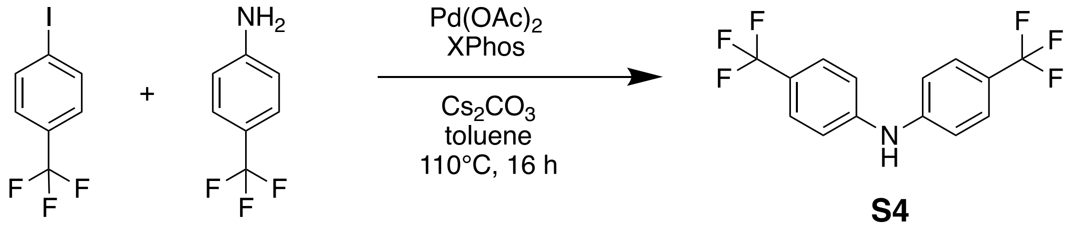

S4

4-Iodobenzotrifluoride (1.01 g, 3.71 mmol, 1.0 eq.), 4-(trifluoromethyl)aniline (598 mg, 3.71 mmol, 1.0 eq.) and caesium carbonate (2.42 g, 7.43 mmol, 2.0 eq.) were dissolved in toluene (10 mL). The reaction mixture was degassed and $Pd(OAc)_2$ (83.0 mg, 371 μmol, 0.1 eq.) and XPhos (177 mg, 371 μmol, 0.1 eq.) were added under a nitrogen atmosphere. The reaction mixture was refluxed over night and then cooled to room temperature. Water (50 mL) was added and the mixture extracted with ethyl acetate (3 × 50 mL). The combined organic phases were dried over Na_2SO_4, the solvent was removed under reduced pressure and the crude mixture was purified by column chromatography (SiO_2, cyclohexane / ethyl acetate 1/0 → 20/1) to obtain bis(4-(trifluoromethyl)phenyl)amine (**S4**, 820 mg, 2.69 mmol, 72%) as a beige solid.

^{1}H-NMR (400 MHz, $CDCl_3$): δ 7.55 (d, $^3J_{H,H}$ = 8.4 Hz, 4H), 7.16 (d, $^3J_{H,H}$ = 8.4 Hz, 4H), 6.10 (s, 1H) ppm.

^{19}F{^{1}H}-NMR (376 MHz, $CDCl_3$): δ -61.76 (s, 6F) ppm.

^{13}C-NMR (101 MHz, $CDCl_3$): δ 144.9 (q, $^4J_{C,F}$ = 1.2 Hz), 127.0 (q, $^3J_{C,F}$ = 3.8 Hz), 124.5 (q, $^1J_{C,F}$ = 270.7 Hz), 123.7 (q, $^2J_{C,F}$ = 32.8 Hz), 117.5 ppm.

These analytical data are in agreement with previously reported characterization data for this compound.[13]

4-Methyl-N,N-bis(4-(trifluoromethyl)phenyl)benzenesulfonamide (23)

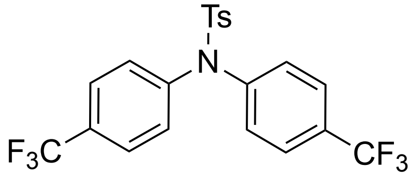

23

The general procedure 2 for tosylation using substrate **S4** (610 mg, 2.00 mmol, 1.0 eq.) in THF (10 mL) yielded 4-methyl-*N*,*N*-bis(4-(trifluoromethyl)phenyl)benzenesulfonamide (**S3**, 400 mg, 871 μmol, 44%) as a white solid after flash column chromatography (SiO_2, cyclohexane / dichloromethane 9/1 → 1/1).

^{1}H-NMR (400 MHz, $CDCl_3$): δ 7.63 - 7.57 (m, 6H), 7.35 (d, $J_{H,H}$ = 8.3 Hz, 4H), 7.32 (d, $J_{H,H}$ = 8.4 Hz, 2H), 2.45 (s, 3H) ppm.

^{19}F{^{1}H}-NMR (376 MHz, $CDCl_3$): δ -61.62 (s, 6F) ppm.

^{13}C-NMR (101 MHz, $CDCl_3$): δ 197.0 145.7, 141.6, 134.5, 133.3, 129.9, 128.3, 126.4, 125.8, 120.8, 114.8, 26.7, 21.5 ppm.

Elemental analysis for $C_{21}H_{15}F_6NO_2S$ found (calculated): C 54.75 (54.90), H 3.31 (3.29), N 3.10 (3.05).
High resolution ESI-MS (m/z) for $C_{21}H_{15}F_6NO_2S$+Na [**23**+Na]$^+$: 482.0621 (calculated: 482.0620).

N,N-Bis(4-bromophenyl)-4-methylbenzenesulfonamide (24)

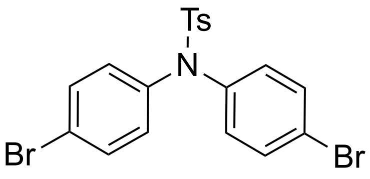

24

The general procedure 2 for tosylation using bis(4-bromophenyl)amine (980 mg, 3.00 mmol, 1.0 eq.) in THF (15 mL) yielded *N,N*-bis(4-bromophenyl)-4-methylbenzenesulfonamide (**24**, 575 mg, 1.19 mmol, 40%) as a white solid after flash column chromatography (SiO_2, cyclohexane / ethyl acetate 20/1) and washing with cold pentane (10 mL).

^{1}H-NMR (400 MHz, $CDCl_3$): δ 7.49 (d, $J_{H,H}$ = 8.3 Hz, 2H), 7.40 - 7.34 (m, 4H), 7.22 (d, $J_{H,H}$ = 8.2 Hz, 2H), 7.07 - 7.00 (m, 4H), 2.38 (s, 3H) ppm.

^{13}C-NMR (101 MHz, $CDCl_3$): δ 144.3, 140.4, 137.0, 132.7, 129.9, 129.8, 127.9, 121.6, 21.8 ppm.
Elemental analysis for $C_{19}H_{15}Br_2NO_2S$ found (calculated): C 47.37 (47.42), H 3.19 (3.13), N 3.04 (2.91).
High resolution ESI-MS (m/z) for $C_{19}H_{15}Br_2NO_2S$+Na [**24**+Na]$^+$: 501.9077 (calculated: 501.9082).

Bis(4-chlorophenyl)amine (S5)

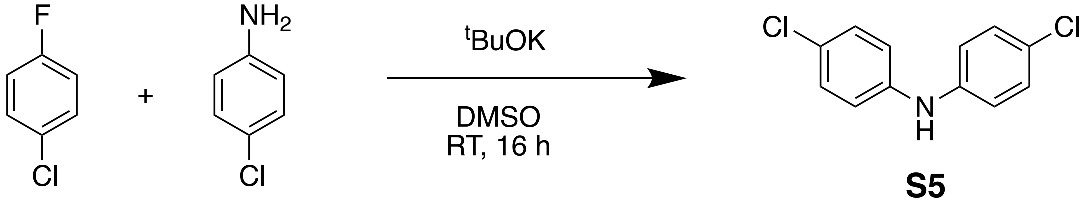

S5

1-Chloro-4-fluorobenzene (620 mg, 4.75 mmol, 1.0 eq.) and 4-chloroaniline (530 µL, 5.94 mmol, 1.25 eq.) were dissolved in DMSO (7 mL), potassium *tert*-butoxide (1.12 g, 10.0 mmol, 2.0 eq.) was added and the reaction mixture was stirred at room temperature over night. The reaction mixture was poured into water (50 mL), extracted with dichloromethane (3 × 75 mL), the combined organic phases were dried over Na_2SO_4 and the solvent was removed under reduced pressure. The crude product was purified by flash column chromatography (SiO_2, cyclohexane / ethyl acetate 1/0 → 4/1) to obtain bis(4-chlorophenyl)amine (**S5**, 508 mg, 2.13 mmol, 45%) as a pale white solid.

^{1}H-NMR (400 MHz, $CDCl_3$): δ 7.72 (d, $J_{H,H}$ = 8.8 Hz, 4H), 6.96 (d, $J_{H,H}$ = 8.8 Hz, 4H), 5.63 (s, 1H) ppm.

^{13}C-NMR (101 MHz, $CDCl_3$): δ 141.5, 129.5, 126.2, 119.3 ppm.

These analytical data are in agreement with previously reported characterization data for this compound.[14]

N,N-Bis(4-chlorophenyl)-4-methylbenzenesulfonamide (25)

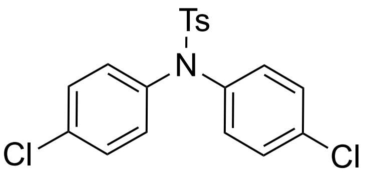

25

The general procedure 2 for tosylation using substrate **S5** (500 mg, 2.10 mmol, 1.0 eq.) in THF (12 mL) yielded *N,N*-bis(4-chlorophenyl)-4-methylbenzenesulfonamide (**25**, 130 mg, 306 µmol, 15%) as a white solid after flash column chromatography (SiO_2, cyclohexane / ethyl acetate 1/0 → 4/1).

^{1}H-NMR (400 MHz, $CDCl_3$): δ 7.56 (d, $J_{H,H}$ = 8.4 Hz, 2H), 7.32 – 7.26 (m, 6H), 7.17 (d, $J_{H,H}$ = 8.8 Hz, 4H), 2.44 (s, 3H) ppm.

^{13}C-NMR (101 MHz, $CDCl_3$): δ 144.3, 139.9, 137.0, 133.6, 129.9, 129.7, 129.5, 127.9, 21.8 ppm.

Elemental analysis for $C_{19}H_{15}Cl_2NO_2S$ found (calculated): C 58.27 (58.17), H 4.03 (3.82), N 3.71 (3.57).

High resolution ESI-MS (m/z) for $C_{19}H_{15}Cl_2NO_2S$+Na [**25**+Na]$^+$: 414.0095 (calculated: 414.0093).

10-Tosyl-10H-phenoxazine (26)

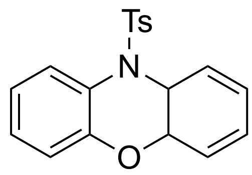

26

Phenoxazine (550 mg, 3.00 mmol, 1.0 eq.) was dissolved in dichloromethane (10 mL) and triethylamine (1.64 mL, 12.0 mmol, 4.0 eq.) and tosyl chloride (860 mg, 4.51 mmol, 1.5 eq.) were slowly added. The reaction mixture was stirred at room temperature for 24 h, quenched by the addition of water (25 mL) and the mixture was extracted with dichloromethane (3 × 50 mL). The combined organic phases were dried over Na_2SO_4, the solvent was removed under reduced pressure and the crude mixture was purified by flash column chromatography (SiO_2, cyclohexane / ethyl acetate 1/0 → 3/1) followed by recrystallization from hexane / ethyl acetate (5/1) to obtain the desired 10-tosyl-10*H*-phenoxazine (**26**, 650 mg, 1.93 mmol, 64%) as white crystals.

^{1}H-NMR (400 MHz, $CDCl_3$): δ 7.67 (dd, $J_{H,H}$ = 7.7 Hz, $J_{H,H}$ = 1.9 Hz, 2H), 7.20 (td, $J_{H,H}$ = 7.7 Hz, $J_{H,H}$ = 1.9 Hz, 1H), 7.15 (td, $J_{H,H}$ = 7.6 Hz, $J_{H,H}$ = 1.7 Hz, 2H), 7.03 (d, $J_{H,H}$ = 8.1 Hz, 2H), 6.96 (d, $J_{H,H}$ = 8.3 Hz, 2H), 6.80 (dd, $J_{H,H}$ = 7.9 Hz, $J_{H,H}$ = 1.7 Hz, 2H), 2.35 (s, 3H) ppm.

^{13}C-NMR (101 MHz, $CDCl_3$): δ 151.3, 144.5, 132.7, 129.2, 128.5, 128.2, 127.9, 126.5, 123.9, 116.4, 21.8 ppm.
These analytical data are in agreement with previously reported characterization data for this compound.[15]

N,N-Bis(4-methoxyphenyl)-4-methylbenzenesulfonamide (27)

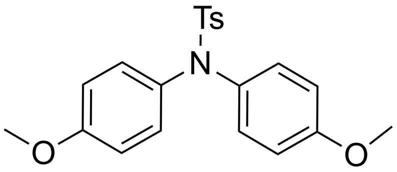

27

The general procedure 2 for tosylation using bis(4-methoxyphenyl)amine (1.50 g, 6.54 mmol, 1.0 eq.) in THF (20 mL) yielded *N,N*-bis(4-methoxyphenyl)-4-methylbenzenesulfonamide (**27**, 722 mg, 1.88 mmol, 29%) as a pale violet solid after flash column chromatography (SiO_2, cyclohexane / ethyl acetate 1/0 → 4/1) followed by recrystallization from hexane / ethyl acetate (2/1).

^{1}H-NMR (400 MHz, $CDCl_3$): δ 7.56 (d, $J_{H,H}$ = 8.3 Hz, 2H), 7.26 (d, $J_{H,H}$ = 7.8 Hz, 2H), 7.18 (d, $J_{H,H}$ = 9.0 Hz, 4H), 6.81 (d, $J_{H,H}$ = 9.0 Hz, 4H), 3.77 (s, 6H), 2.44 (s, 3H) ppm.

^{13}C-NMR (101 MHz, $CDCl_3$): δ 158.89, 143.5, 137.7, 134.6, 129.9, 129.6, 128.0, 114.5, 55.6, 21.7 ppm.

High resolution ESI-MS (m/z) for $C_{21}H_{21}NO_4S$+Na [**27**+Na]$^+$: 406.1086 (calculated: 406.1083).

2-Phenyl-1-tosyl-1H-imidazole (28)

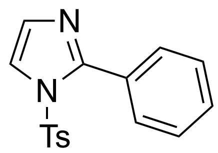

28

The general procedure 2 for tosylation using 2-phenyl-1*H*-imidazole (721 mg, 5.01 mmol, 1.0 eq.) in THF (20 mL) yielded 2-phenyl-1-tosyl-1*H*-imidazole (**28**, 1.42 g, 4.76 mmol, 95%) as a white solid after flash column chromatography (SiO_2, pentane / ethyl acetate 9/1 → 1/1).

^{1}H-NMR (400 MHz, $CDCl_3$): δ 7.63 (d, $J_{H,H}$ = 1.7 Hz, 1H), 7.49 - 7.41 (m, 1H), 7.41 - 7.38 (m, 2H), 7.37 -7.33 (m, 2H), 7.27 (d, $J_{H,H}$ = 8.4 Hz, 2H), 7.11 (d, $J_{H,H}$ = 8.0 Hz, 2H), 7.10 (d, $J_{H,H}$ = 1.7 Hz, 1H), 2.36 (s, 4H) ppm.

^{13}C-NMR (101 MHz, $CDCl_3$): δ 148.7, 145.9, 134.7, 130.7, 130.0, 129.8, 129.6, 128.8, 127.8, 127.6, 120.9, 21.8 ppm.

These analytical data are in agreement with previously reported characterization data for this compound.[16]

2-Phenyl-1-tosyl-1H-benzoimidazole (29)

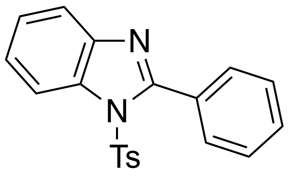

29

The general procedure 2 for tosylation using 2-phenyl-1*H*-benzoimidazole (920 mg, 4.74 mmol, 1.0 eq.) in THF (20 mL) yielded 2-phenyl-1-tosyl-1*H*-benzoimidazole (**29**, 1.25 g, 3.60 mmol, 76%) as a pale orange solid after flash column chromatography (SiO_2, cyclohexane / ethyl acetate 9/1 → 1/1).

^{1}H-NMR (400 MHz, $CDCl_3$): δ 8.21 (d, $J_{H,H}$ = 8.0 Hz, 1H), 7.73 (dd, $J_{H,H}$ = 7.5 Hz, $J_{H,H}$ = 0.9 Hz, 1H), 7.61 (dd, $J_{H,H}$ = 8.3 Hz, $J_{H,H}$ = 1.3 Hz, 2H), 7.55 (t, $J_{H,H}$ = 7.5 Hz, 1H), 7.48 - 7.37 (m, 4H), 7.33 (d, $J_{H,H}$ = 8.4 Hz, 2H), 7.09 (d, J = 7.8 Hz, 2H), 2.32 (s, 3H) ppm.

^{13}C-NMR (101 MHz, $CDCl_3$): δ 154.2 145.8, 142.8, 135.2, 134.0, 131.0, 130.6, 130.2, 129.8, 127.8, 127.1, 125.6, 125.4, 120.5, 115.3, 21.7 ppm.

These analytical data are in agreement with previously reported characterization data for this compound.[17]

1-Tosyl-1H-indole (30)

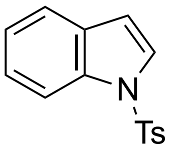

30

The general procedure 2 for tosylation using 1*H*-indole (580 mg, 4.95 mmol, 1.0 eq.) in THF (20 mL) yielded 1-tosyl-1*H*-indole (**30**, 1.31 g, 4.83 mmol, 98%) as off-white solid after flash column chromatography (SiO_2, cyclohexane / ethyl acetate 9/1 → 2/1).

^{1}H-NMR (400 MHz, $CDCl_3$): δ 7.99 (dd, $J_{H,H}$ = 8.3 Hz, $J_{H,H}$ = 0.9 Hz, 1H), 7.76 (d, $J_{H,H}$ = 8.4 Hz, 2H), 7.57 (d, $J_{H,H}$ = 3.7 Hz, 1H), 7.52 (dt, $J_{H,H}$ = 7.8 Hz, $J_{H,H}$ = 1.0 Hz, 1H), 7.31 (ddd, $J_{H,H}$ = 8.4, $J_{H,H}$ = 7.2 Hz, $J_{H,H}$ = 1.3 Hz, 1H), 7.25 - 7.17 (m, 3H), 6.65 (dd, $J_{H,H}$ = 3.7, 0.8 Hz, 1H), 2.33 (s, 3H) ppm.

^{13}C-NMR (101 MHz, $CDCl_3$): δ 145.3, 135.7, 135.2, 131.1, 130.2, 127.2, 126.7, 124.9, 123.6, 121.7, 113.92, 109.4, 21.9 ppm.

These analytical data are in agreement with previously reported characterization data for this compound.[16]

5-Fluoro-1-tosyl-1H-indole (31)

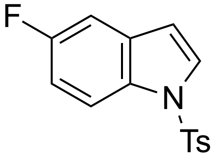

31

The general procedure 2 for tosylation using 5-fluoro-1*H*-indole (540 mg, 4.01 mmol, 1.0 eq.) in THF (20 mL) yielded 5-fluoro-1-tosyl-1*H*-indole (**31**, 985 mg, 3.40 mmol, 85%) as white solid after flash column chromatography (SiO_2, cyclohexane / ethyl acetate 9/1 → 2/1).

^{1}H-NMR (400 MHz, $CDCl_3$): δ 7.92 (dd, $^{3}J_{H,H}$ = 9.0 Hz, $J_{H,H}$ = 4.4 Hz, 1H), 7.74 (d, $^{3}J_{H,H}$ = 8.4 Hz, 2H), 7.59 (d, $^{3}J_{H,H}$ = 3.7 Hz, 1H), 7.23 (d, $^{3}J_{H,H}$ = 8.1 Hz, 2H), 7.17 (dd, $J_{H,F}$ = 8.8 Hz, $J_{H,H}$ = 2.5 Hz, 1H), 7.03 (td, $^{3}J_{H,H}$ = 9.1 Hz, $^{4}J_{H,H}$ = 2.6 Hz, 1H), 6.61 (dd, $^{3}J_{H,H}$ = 3.6 Hz, $^{4}J_{H,F}$ = 0.8 Hz, 1H), 2.34 (s, 3H) ppm.

^{19}F{^{1}H}-NMR (376 MHz, $CDCl_3$): δ -120.0 (s, 1F) ppm.

^{13}C-NMR (101 MHz, $CDCl_3$): δ 159.7 (d, $^{1}J_{C,H}$ = 240.0 Hz), 145.2, 135.2, 131.8 (d, $^{3}J_{C,H}$ = 10.1 Hz), 131.3 (d, $^{4}J_{C,H}$ = 1.1 Hz), 130.1, 128.2, 126.9, 114.7 (d, $^{1}J_{C,H}$ = 9.4 Hz), 112.7 (d, $^{2}J_{C,H}$ = 25.7 Hz), 109.0 (d, $^{4}J_{C,H}$ = 4.1 Hz), 107.0 (d, $^{2}J_{C,F}$ = 24.0 Hz), 21.7 ppm.

These analytical data are in agreement with previously reported characterization data for this compound.[16]

1-Tosyl-1H-pyrrole (32)

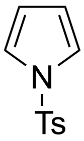

32

The general procedure 2 for tosylation using pyrrole (260 µL, 3.76 mmol, 1.0 eq.) in THF (20 mL) yielded 1-tosyl-1*H*-pyrrole (**32**, 692 mg, 3.13 mmol, 84%) as a white solid after flash column chromatography (SiO_2, pentane / ethyl acetate 1/0 → 0/1).

^{1}H-NMR (400 MHz, $CDCl_3$): δ 7.74 (d, $J_{H,H}$ = 8.4 Hz, 2H), 7.28 (d, $J_{H,H}$ = 7.9 Hz, 2H), 7.18 – 7.12 (m, 2H), 6.29 – 6.26 (m, 2H), 2.40 (s, 3H) ppm.

^{13}C-NMR (101 MHz, $CDCl_3$): δ 145.1, 136.3, 130.1, 127.0, 120.9, 113.6, 21.8 ppm.

These analytical data are in agreement with previously reported characterization data for this compound.[18]

N-(4-Fluorobenzyl)-4-(trifluoromethyl)aniline (S6)

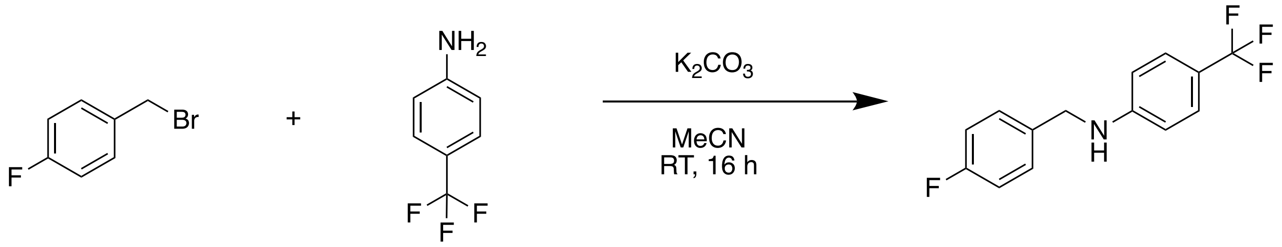

S6

4-Flurobenzyl bromide (625 µL, 5.00 mmol, 1.0 eq.) and 4-(trifluoromethyl)aniline (944 µL, 7.50 mmol, 1.5 eq.) were dissolved in MeCN (10 mL) and potassium carbonate (2.07 g, 15.0 mmol, 3.0 eq.) was added. The reaction mixture was stirred at room temperature over night, the solvent was removed under reduced pressure, water (25 mL) was added and the resulting mixture was extracted with dichloromethane (3 × 50 mL). The combined organic phases were dried over Na_2SO_4, the solvent was removed under reduced pressure and the crude mixture was purified by flash column chromatography (SiO_2, cyclohexane / dichloromethane 1/0 → 4/1) to obtain *N*-(2-fluorophenyl)-*N*,4-dimethylbenzene-sulfonamide (**S6**, 500 mg, 1.86 mmol, 37%) as pale yellow solid.

^{1}H-NMR (400 MHz, $CDCl_3$): δ 7.32 (d, $J_{H,H}$ = 8.5 Hz, 2H), 7.24 (dd, $J_{H,H}$ = 8.6 Hz, $J_{H,H}$ = 5.4 Hz, 2H), 6.96 (t, $J_{H,H}$ = 8.6 Hz, 2H), 6.54 (d, $J_{H,H}$ = 8.4 Hz, 2H), 4.27 (s, 2H), 4.26 (s, 1H) ppm.

^{19}F{^{1}H}-NMR (376 MHz, $CDCl_3$): δ -61.07 (s, 3F), -115.07 (s, 1F) ppm.

^{13}C-NMR (101 MHz, $CDCl_3$): δ 162.3 (d, $J_{C,F}$ = 245.7 Hz), 150.4 (d, $J_{C,F}$ = 1.1 Hz), 134.3 (d, $J_{C,F}$ = 3.1 Hz), 129.1 (d, $J_{C,F}$ = 8.1 Hz), 126.8 (q, $J_{C,F}$ = 3.8 Hz), 125.0 (q, $J_{C,F}$ = 270.3 Hz), 119.4 (q, $J_{C,F}$ = 32.7 Hz), 115.8 (d, $J_{C,F}$ = 21.5 Hz), 112.2, 47.3 (d, $J_{C,F}$ = 0.7 Hz) ppm.

These analytical data are in agreement with previously reported characterization data for this compound.[19]

N-(4-Fluorobenzyl)-4-methyl-N-(4-(trifluoromethyl)phenyl)benzene-sulfonamide (33)

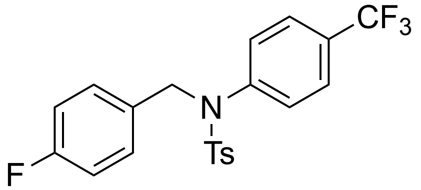

33

The general procedure 2 for tosylation using substrate **S6** (450 mg, 1.67 mmol, 1.0 eq.) in THF (10 mL) yielded *N*-(4-fluorobenzyl)-4-methyl-*N*-(4-(trifluoromethyl)phenyl)benzene-sulfonamide (**33**, 180 mg, 425 µmol, 25%) as a white solid after flash column chromatography (SiO_2, cyclohexane / ethyl acetate 1/0 → 1/3).

^{1}H-NMR (400 MHz, $CDCl_3$): δ 7.52 (d, $J_{H,H}$ = 8.3 Hz, 2H), 7.48 (d, $J_{H,H}$ = 8.0 Hz, 2H), 7.30 (d, $J_{H,H}$ = 7.9 Hz, 2H), 7.18 (dd, $J_{H,H}$ = 8.6 Hz, $J_{H,F}$ = 5.4 Hz, 1H), 7.11 (d, $J_{H,H}$ = 7.9 Hz, 2H), 6.92 (t, $J_{H,H}$ = 8.7 Hz, 2H), 4.71 (s, 2H), 2.45 (s, 3H) ppm.

^{19}F{^{1}H}-NMR (376 MHz, $CDCl_3$): δ -62.61 (s, 3F), -114.10 (s, 1F) ppm.

^{13}C-NMR (101 MHz, $CDCl_3$): δ 162.5 (d, $J_{C,F}$ = 246.8 Hz), 144.2, 142.3, 135.3, 131.2 (d, $J_{C,F}$ = 3.2 Hz), 130.3 (d, $J_{C,F}$ = 8.3 Hz), 129.9, 129.9 (q, $J_{C,F}$ = 32.9 Hz), 129.0, 127.8, 126.2 (q, $J_{C,F}$ = 3.7 Hz), 123.8 (q, $J_{C,F}$ = 272.3 Hz), 115.7 (d, $J_{C,F}$ = 21.6 Hz), 53.8, 21.7 ppm.

Elemental analysis for $C_{21}H_{17}F_4NO_2S{\cdot}0.15(C_6H_{12})$ found (calculated): C 60.27 (60.32), H 4.58 (4.35), N 3.25 (3.21).

High resolution ESI-MS (m/z) for $C_{21}H_{17}F_4NO_2S$+Na [**33**+Na]$^+$: 446.0802 (calculated: 446.0808).

N-(4-Fluorobenzyl)-4-methylaniline (S7)

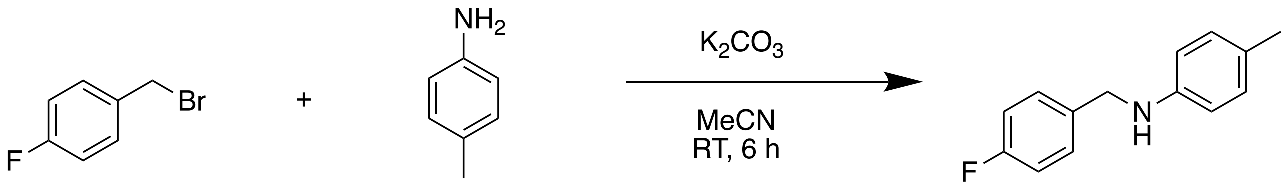

S7

4-Fluorobenzyl bromide (650 µL, 5.19 mmol, 1.0 eq.) and 4-methylaniline (803 mg, 7.50 mmol, 1.5 eq.) were dissolved in MeCN (15 mL) and potassium carbonate (2.15 g, 15.6 mmol, 3.0 eq.) was added. The reaction mixture was stirred at room temperature over 6 hours, the solvent was removed under reduced pressure, water (25 mL) was added and the resulting mixture was extracted with dichloromethane (3 × 50 mL). The combined organic phases were dried over Na_2SO_4, the solvent was removed under reduced pressure and the crude mixture was purified by flash column chromatography (SiO_2, cyclohexane / dichloromethane 1/0 → 4/1) to obtain *N*-(4-fluorobenzyl)-4-methylaniline (**S7**, 502 mg, 2.32 mmol, 45%) as oily liquid.

^{1}H-NMR (400 MHz, $CDCl_3$): δ 7.33 (dd, $J_{H,H}$ = 8.5 Hz, $^{3}J_{H,F}$ = 5.5 Hz, 2H), 7.03 (d, $J_{H,H}$ = 8.7 Hz, 2H), 7.01 – 6.98 (m, 2H), 6.55 (d, $^{3}J_{H,H}$ = 8.4 Hz, 2H), 4.28 (s, 2H), 3.90 (s, 1H), 2.24 (s, 3H) ppm.

^{19}F{^{1}H}-NMR (376 MHz, $CDCl_3$): δ -115.80 (s, 1F) ppm.

^{13}C-NMR (101 MHz, $CDCl_3$): δ 162.0 (d, $^{1}J_{C,F}$ = 244.8 Hz), 145.7, 135.3 (d, $^{4}J_{C,F}$ = 3.2 Hz), 129.8, 129.0 (d, $^{3}J_{C,F}$ = 8.0 Hz), 127.0, 115.4 (d, $^{2}J_{C,F}$ = 21.5 Hz), 113.0, 47.9, 20.4 ppm.

These analytical data are in agreement with previously reported characterization data for this compound.[20,21]

N-(4-Fluorobenzyl)-4-methyl-N-(p-tolyl)benzenesulfonamide (34)

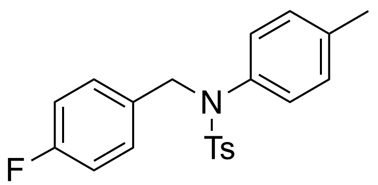

34

The general procedure 2 for tosylation using substrate **S7** (500 mg, 2.32 mmol, 1.0 eq.) in THF (6 mL) yielded *N*-(4-fluorobenzyl)-4-methyl-*N*-(4-(trifluoromethyl)phenyl)benzene-sulfonamide (**34**, 220 mg, 595 µmol, 26%) as a white solid after flash column chromatography (SiO_2, cyclohexane / dichloromethane 9/1 → 1/1).

^{1}H-NMR (400 MHz, $CDCl_3$): δ 7.54 (d, $^{3}J_{H,H}$ = 8.3 Hz, 2H), 7.28 (d, $^{3}J_{H,H}$ = 8.1 Hz, 2H), 7.18 (dd, $^{3}J_{H,H}$ = 8.5 Hz, $^{4}J_{H,F}$ = 5.5 Hz, 2H), 7.00 (d, $^{3}J_{H,H}$ = 8.1 Hz, 2H), 6.90 (t, $^{3}J_{H,H,H,F}$ = 8.7 Hz, 2H), 6.82 (d, $^{3}J_{H,H}$ = 8.3 Hz, 2H), 4.65 (s, 1H), 2.44 (s, 3H), 2.27 (s, 3H).

^{19}F{^{1}H}-NMR (376 MHz, $CDCl_3$): δ -114.81 (s, 1F) ppm.

^{13}C-NMR (101 MHz, $CDCl_3$): δ 162.3 (d, $^{1}J_{C,F}$ = 245.9 Hz), 143.5, 138.0, 136.2, 135.8, 132.0 (d, $^{4}J_{C,F}$ = 3.2 Hz), 130.4 (d, $^{3}J_{C,F}$ = 8.2 Hz), 129.7, 129.6, 128.8, 127.9, 115.4 (d, $^{2}J_{C,F}$ = 21.5 Hz), 54.2, 21.7, 21.2 ppm.

Elemental analysis for $C_{21}H_{20}FNO_2S$•0.05(CH_2Cl_2) found (calculated): C 67.54 (67.66), H 5.40 (5.42), N 3.69 (3.75).

High resolution ESI-MS (m/z) for $C_{21}H_{20}FNO_2S$+Na [**34**+Na]$^+$: 392.1095 (calculated: 392.1091).

N-(2-Fluorophenyl)-N-tosylacetamide (35)

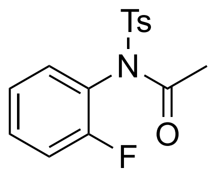

35

N-(2-fluorophenyl)-4-methylbenzenesulfonamide (**38**, 385 mg, 1.45 mmol, 1.0 eq.) and triethylamine (402 µL, 2.90 mmol, 2.0 eq.) were dissolved in dichloromethane (5 mL) at 0 °C and acyl chloride (155 µL, 2.18 mmol, 1.5 eq.) was slowly added. The reaction mixture was stirred at room temperature for 4 h, quenched by the addition of saturated aqueous NH_4Cl solution (25 mL) and extracted with dichloromethane (3 × 50 mL). The combined organic phases were dried over Na_2SO_4, the solvent was removed under reduced pressure and the crude mixture was purified by flash column chromatography (SiO_2, cyclohexane / ethyl acetate 1/0 → 1/1) to obtain *N*-(2-fluorophenyl)-*N*-tosylacetamide (**35**, 370 mg, 1.20 mmol, 82%) as a white solid.

^{1}H-NMR (400 MHz, $CDCl_3$): δ 7.95 (d, $J_{H,H}$ = 8.1 Hz, 2H), 7.55 – 7.44 (m, 1H), 7.42 (t, $J_{H,H}$ = 7.5 Hz, 1H), 7.34 (d, $J_{H,H}$ = 8.1 Hz, 2H), 7.29 (t, $J_{H,H}$ = 7.5 Hz, 1H), 7.23 (d, $J_{H,H}$ = 8.7 Hz, 1H), 2.45 (s, 3H), 1.90 (s, 3H) ppm.

$^{19}F\{^1H\}$-NMR (376 MHz, $CDCl_3$): δ -118.65 (s, 1F) ppm.

^{13}C-NMR (101 MHz, $CDCl_3$): δ 169.6, 158.9 (d, $J_{C,F}$ = 252.1 Hz), 145.3, 135.9, 132.3, 132.2 (d, $J_{C,F}$ = 7.9 Hz), 129.5 (d, $J_{C,F}$ = 1.4 Hz), 129.4, 125.4 (d, $J_{C,F}$ = 4.0 Hz), 124.7 (d, $J_{C,F}$ = 13.6 Hz), 117.1 (d, $J_{C,F}$ = 20.0 Hz), 24.4, 21.9 ppm.

Elemental analysis for $C_{15}H_{14}FNO_3S$ found (calculated): C 58.48 (58.62), H 4.61 (4.59), N 4.39 (4.56).

High resolution ESI-MS (m/z) for $C_{15}H_{14}FNO_3S$+Na [**35**+Na]$^+$: 330.0574 (calculated: 330.0571).

N-(2-Fluorophenyl)-N,4-dimethylbenzenesulfonamide (36)

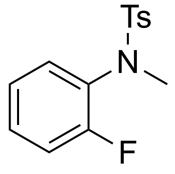

36

N-(2-fluorophenyl)-4-methylbenzenesulfonamide (**38**, 500 mg, 1.88 mmol, 1.0 eq.) was dissolved in DMF (5 mL). Potassium carbonate (1.55 g, 11.22 mmol, 6.0 eq.) and methyl iodide (176 µL, 2.85 mmol, 1.5 eq.) were added. The reaction mixture was stirred at room temperature over night, quenched by the addition of water (25 mL) and extracted with dichloromethane (3 × 50 mL). The combined organic phases were dried over Na_2SO_4, the solvent was removed under reduced pressure and the crude mixture was purified by flash column chromatography (SiO_2, cyclohexane / ethyl acetate 20/1 → 4/1) to obtain *N*-(2-fluorophenyl)-*N*,4-dimethylbenzenesulfonamide (**36**, 430 mg, 1.54 mmol, 82%) as an off-white solid.

^1H-NMR (400 MHz, $CDCl_3$): δ 7.52 (d, $J_{H,H}$ = 8.1 Hz, 2H), 7.32 - 7.18 (m, 4H), 7.08 (td, $J_{H,H}$ = 7.7 Hz, $J_{H,H}$ = 1.5 Hz, 1H), 6.98 (ddd, $J_{H,H}$ = 10.8, $J_{H,H}$ = 8.2, $J_{H,H}$ = 1.5 Hz, 1H), 3.16 (d, $J_{H,H}$ = 1.5 Hz, 3H), 2.39 (s, 3H) ppm.

$^{19}F\{^1H\}$-NMR (376 MHz, $CDCl_3$): δ -118.72 (s, 1F) ppm.

^{13}C-NMR (101 MHz, $CDCl_3$): δ 159.4 (d, $^1J_{C,F}$ = 252.8 Hz), 143.7, 135.2, 131.6 (d, $^4J_{C,F}$ = 1.5 Hz), 129.8 (d, $^3J_{C,F}$ = 8.0 Hz), 129.6, 128.7 (d, $^2J_{C,F}$ = 11.6 Hz), 127.8, 124.6 (d, $^4J_{C,F}$ = 3.7 Hz), 116.8 (d, $^2J_{C,F}$ = 20.1 Hz), 38.21 (d, $J_{C,F}$ = 3.8 Hz), 21.7 ppm.

These analytical data are in agreement with previously reported characterization data for this compound.[22]

N-Butyl-N-(2-fluorophenyl)-4-methylbenzenesulfonamide (37)

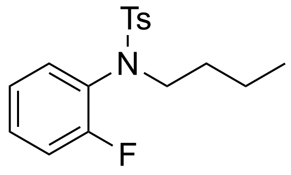

37

N-(2-fluorophenyl)-4-methylbenzenesulfonamide (**38**, 300 mg, 1.13 mmol, 1.0 eq.) were dissolved in DMF (5 mL) and potassium carbonate (781 mg, 5.65 mmol, 5.0 eq.) and butyl iodide (193 µL, 1.70 mmol, 1.5 eq.) were added. The reaction mixture was stirred at room temperature over 3 hours, quenched by the addition of water (25 mL) and extracted with dichloromethane (3 × 50 mL). The combined organic phases were dried over Na_2SO_4, the solvent was removed under reduced pressure and the crude mixture was purified by flash column chromatography (SiO_2, cyclohexane / ethyl acetate 1/0 → 4/1) to obtain *N*-butyl-*N*-(2-fluorophenyl)-4-methylbenzenesulfonamide (**37**, 271 mg, 843 µmol, 75%) as a colourless oil.

^{1}H-NMR (400 MHz, $CDCl_3$): δ 7.56 (d, $^3J_{H,H}$ = 8.3 Hz, 2H), 7.33 – 7.27 (m, 1H), 7.24 (d, $^3J_{H,H}$ = 8.1 Hz, 2H), 7.21 (dd, $^3J_{H,H}$ = 7.7 Hz, $J_{H,F}$ = 1.8 Hz, 1H), 7.12 (td, $^3J_{H,H}$ = 7.7, $J_{H,F}$ = 1.4 Hz, 1H), 7.03 (ddd, $J_{H,H}$ = 10.5 Hz, $J_{H,H}$ = 8.3 Hz, $J_{H,H}$ = 1.4 Hz, 1H), 3.53 (t, $^3J_{H,H}$ = 7.0 Hz, 2H), 2.42 (s, 3H), 1.40 – 1.23 (m, 4H), 0.85 (t, J = 7.1 Hz, 2H) ppm.

^{19}F{^{1}H}-NMR (376 MHz, $CDCl_3$): δ -118.14 (s, 1F) ppm.

^{13}C-NMR (101 MHz, $CDCl_3$): δ 160.1 (d, $^1J_{C,F}$ = 252.8 Hz), 143.5, 136.4, 132.7 (d, $^2J_{C,F}$ = 1.3 Hz), 130.0 (d, $^3J_{C,F}$ = 8.1 Hz), 129.6, 127.7, 126.5 (d, $^3J_{C,F}$ = 11.5 Hz), 124.5 (d, $^4J_{C,F}$ = 3.8 Hz), 116.8 (d, $^2J_{C,F}$ = 20.3 Hz), 50.2 (d, $^4J_{C,F}$ = 3.2 Hz), 30.8, 21.7, 19.7, 13.8 ppm.

Elemental analysis for $C_{17}H_{20}FNO_2S$ found (calculated): C 63.70 (63.53), H 6.27 (6.22), N 4.33 (4.36).

High resolution ESI-MS (m/z) for $C_{17}H_{20}FNO_2S$+Na [**37**+Na]$^+$: 344.1093 (calculated: 344.1091).

N-(2-Fluorophenyl)-4-methylbenzenesulfonamide (38)

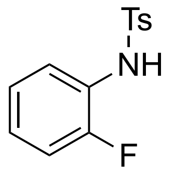

38

2-Fluoroaniline (870 µL, 9.00 mmol, 1.0 eq.), pyridine (4.34 mL, 54.0 mmol, 6.0 eq.) and tosyl chloride (1.89 g, 9.90 mmol, 1.1 eq.) were dissolved in dichloromethane (35 mL). The reaction mixture was stirred at room temperature for 1 h, quenched by the addition of aqueous HCl solution (1 M, 25 mL) and extracted with dichloromethane (3 × 50 mL). The combined organic phases were dried over Na_2SO_4, the solvent was removed

under reduced pressure and the crude mixture was purified by column chromatography (SiO_2, cyclohexane / ethyl acetate 1/0 → 1/1) to obtain *N*-(2-fluorophenyl)-4-methylbenzenesulfonamide (**38**, 1.77 g, 6.67 mmol, 74%) as a white solid.

^{1}H-NMR (400 MHz, $CDCl_3$): δ 7.66 (d, $J_{H,H}$ = 8.4 Hz, 2H), 7.59 (td, $J_{H,H}$ = 7.9, $J_{H,H}$ = 2.3 Hz, 1H), 7.22 (d, $J_{H,H}$ = 8.1 Hz, 2H), 7.12 ‑ 7.02 (m, 2H), 6.98 ‑ 6.92 (m, 1H), 6.72 (s, 1H), 2.38 (s, 3H) ppm.

^{19}F{^{1}H}-NMR (376 MHz, $CDCl_3$): δ -129.90 (s, 1F) ppm.

^{13}C-NMR (101 MHz, $CDCl_3$): δ 154.0 (d, $J_{C,F}$ = 244.4 Hz), 144.3, 136.0, 129.8, 127.3, 126.2 (d, $J_{C,F}$ = 7.5 Hz), 124.9 (d, $J_{C,F}$ = 4.0 Hz), 124.8, 123.3 (d, $J_{C,F}$ = 0.7 Hz), 115.5 (d, $J_{C,F}$ = 19.4 Hz) 21.7 ppm.

These analytical data are in agreement with previously reported characterization data for this compound.[23]

N-(2-Fluorophenyl)-4-methyl-N-tosylbenzenesulfonamide (39)

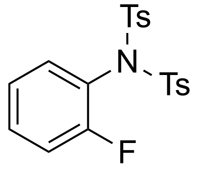

39

2-Fluoroaniline (870 µL, 9.00 mmol, 1.0 eq.), triethylamine (2.6 mL, 19.2 mmol, 2.2 eq.) and tosyl chloride (3.43 g, 18.0 mmol, 2.0 eq.) were dissolved in dichloromethane (20 mL). The reaction mixture was stirred at room temperature over night, quenched by the addition of aqueous HCl solution (1 M, 20 mL) and extracted with dichloromethane (3 × 50 mL). The combined organic phases were dried over Na_2SO_4, the solvent was removed under reduced pressure and the crude mixture was purified by flash column chromatography (SiO_2, cyclohexane / ethyl acetate 5/1 → 3/2) to obtain *N*-(2-fluorophenyl)-4-methyl-*N*-tosylbenzenesulfonamide (**39**, 1.60 g, 3.81 mmol, 42%) as a white solid.

^{1}H-NMR (400 MHz, $CDCl_3$): δ 7.83 (d, $J_{H,H}$ = 8.5 Hz, 4H), 7.47 – 7.40 (m, 1H), 7.33 (d, $J_{H,H}$ = 8.1 Hz, 4H), 7.18 – 7.07 (m, 3H), 2.47 (s, 6H) ppm.

^{19}F{^{1}H}-NMR (376 MHz, $CDCl_3$): δ -115.79 (s, 1F) ppm.

^{13}C-NMR (101 MHz, $CDCl_3$): δ 160.4 (d, $^1J_{C,F}$ = 255.5 Hz), 145.3, 136.6 (d, $J_{C,F}$ = 0.6 Hz), 133.6, 132.4 (d, $^3J_{C,F}$ = 8.1 Hz), 129.7, 128.8 (d, $^4J_{C,F}$ = 0.7 Hz), 124.6 (d, $^3J_{C,F}$ = 3.9 Hz), 122.3 (d, $^2J_{C,F}$ = 13.5 Hz), 116.9 (d, $^2J_{C,F}$ = 20.1 Hz), 21.9 ppm.

Elemental analysis for $C_{20}H_{18}FNO_4S_2$•0.05(CH_2Cl_2) found (calculated): C 56.85 (66.83), H 4.26 (4.31), N 3.34 (3.31).

High resolution ESI-MS (m/z) for $C_{20}H_{18}FNO_4S_2$+Na [**39**+Na]$^+$: 442.0560 (calculated: 442.0553).

1-Tosylpiperidin-2-one (40)

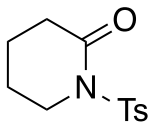

40

The general procedure 2 for tosylation using piperidin-2-one (199 µL, 2.15 mmol, 1.0 eq.) in THF (10 mL) yielded tosylpiperidin-2-one (**40**, 200 mg, 790 µmol, 37%) as a white solid after flash column chromatography (SiO_2, cyclohexane / ethyl acetate 9/1).

^{1}H-NMR (400 MHz, $CDCl_3$): δ 7.91 (d, $J_{H,H}$ = 8.4 Hz, 2H), 7.31 (d, $J_{H,H}$ = 8.5 Hz, 2H), 3.91 (t, $J_{H,H}$ = 6.0 Hz, 2H), 2.45 – 2.38 (m, 5H), 1.96 – 1.82 (m, 2H), 1.83 – 1.74 (m, 2H) ppm.

^{13}C-NMR (101 MHz, $CDCl_3$): δ 170.3, 144.9, 129.4, 128.9 (2x), 47.1, 34.7, 23.5, 21.8, 20.6 ppm.

These analytical data are in agreement with previously reported characterization data for this compound.[24,25]

3-Tosyloxazolidin-2-one (41)

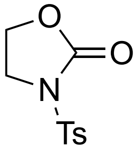

41

The general procedure 2 for tosylation using oxazolidin-2-one (350 mg, 4.02 mmol, 1.0 eq.) in THF (15 mL) yielded 3-tosyloxazolidin-2-one (**41**, 501 mg, 2.08 mmol, 52%) as a white solid after flash column chromatography (SiO_2, cyclohexane / ethyl acetate 9/1 → 1/1).

^{1}H-NMR (400 MHz, $CDCl_3$): δ 7.95 (d, $^3J_{H,H}$ = 8.4 Hz, 2H), 7.37 (d, $^3J_{H,H}$ = 8.1 Hz, 2H), 4.36 (dd, $^3J_{H,H}$ = 8.6 Hz, $^3J_{H,H}$ = 6.9 Hz, 2H), 4.05 (dd, $^3J_{H,H}$ = 8.6 Hz, $^3J_{H,H}$ = 6.9 Hz, 2H), 2.46 (s, 3H) ppm.

^{13}C-NMR (101 MHz, $CDCl_3$): δ 152.2, 146.0, 134.0, 130.1, 128.5, 62.4, 44.8, 21.9 ppm.

Elemental analysis for $C_{10}H_{11}NO_4S$ found (calculated): C 49.77 (49.78), H 4.69 (4.60), N 5.88 (5.81).

High resolution ESI-MS (m/z) for $C_{10}H_{11}NO_4S$+Na [**41**+Na]$^+$: 264.0305 (calculated: 264.0301).

1-Tosylpyrrolidine (42)

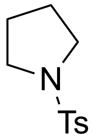

42

The general procedure 2 for tosylation using pyrrolidine (400 µL, 4.02 mmol, 1.0 eq.) in THF (15 mL) yielded 3-tosyloxazolidin-2-one (**42**, 1.01 g, 4.48 mmol, 90%) as a white solid after flash column chromatography (SiO_2, cyclohexane / ethyl acetate 9/1 → 2/1).

^{1}H-NMR (400 MHz, $CDCl_3$): δ 7.71 (d, $^3J_{H,H}$ = 8.3 Hz, 2H), 7.32 (d, $^3J_{H,H}$ = 8.0 Hz, 2H), 3.23 (t, $^3J_{H,H}$ = 6.8 Hz, 4H), 2.42 (s, 3H), 1.74 (t, $^3J_{H,H}$ = 6.9 Hz, 4H) ppm.

^{13}C-NMR (101 MHz, $CDCl_3$): δ 143.4, 134.1, 129.7, 127.7, 48.1, 25.3, 21.7 ppm.

These analytical data are in agreement with previously reported characterization data for this compound.[26]

N-(2-(5-Methoxy-1-tosyl-1H-indol-3-yl)ethyl)acetamide (43)

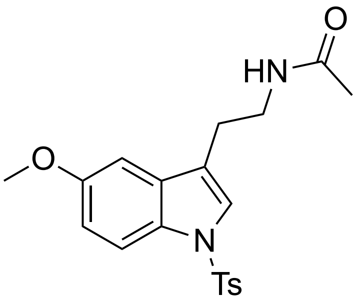

43

Following a literature procedure,[27] melatonin (450 mg, 1.94 mmol, 1.0 eq.) was dissolved in dichloromethane (20 mL) and potassium hydroxide (360 mg, 6.42 mmol, 3.3 eq.), tetra-*n*-butylammonium bromide (370 mg, 1.16 mmol, 0.6 eq.) and tosyl chloride (430 mg, 2.26 mmol, 1.16 eq.) were added. The reaction mixture was stirred at room temperature over night, quenched by the addition of water (25 mL) and extracted with ethyl acetate (3 × 50 mL). The combined organic phases were dried over Na_2SO_4, the solvent was removed under reduced pressure and the crude mixture was purified by flash column chromatography (SiO_2, dichloromethane / methanol 99/1) to obtain tosylated melatonin (**43**, 225 mg, 582 µmol, 30%) as solid white crystals.

^{1}H-NMR (400 MHz, $CDCl_3$): δ 7.90 – 7.82 (m, 1H), 7.70 (d, $J_{H,H}$ = 8.3 Hz, 2H), 7.31 (s, 1H), 7.19 (d, $J_{H,H}$ = 8.1 Hz, 2H), 6.96 – 6.88 (m, 2H), 5.72 (s, 1H), 3.80 (s, 3H), 3.50 (q, $J_{H,H}$ = 6.8 Hz, 2H), 2.82 (t, $J_{H,H}$ = 6.8 Hz, 2H), 2.32 (s, 3H), 1.92 (s, 3H) ppm.

^{13}C-NMR (101 MHz, $CDCl_3$): δ 170.3, 156.6, 145.0, 135.2, 131.8, 130.1, 129.9, 126.8, 124.2, 120.1, 114.8, 114.0, 102.0, 55.8, 39.0, 25.3, 23.4, 21.7 ppm.

These analytical data are in agreement with previously reported characterization data for this compound.[28]

4-Phenyl-3-(phenylsulfonyl)butan-2-one (46)

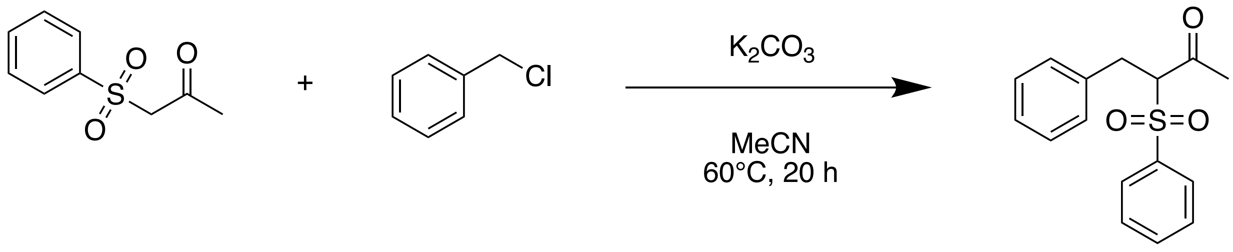

46

Following a literature procedure,[29] 1-(phenylsulfonyl)propan-2-one (412 mg, 2.08 mmol, 1.0 eq.) and K_2CO_3 (400 mg, 2.89 mmol, 1.39 eq.) were suspended in acetonitrile (10 mL) and benzyl chloride (290 µL, 252 mmol, 1.21 eq.) was added. The reaction mixture was stirred for 20 h at 60 °C. The reaction was quenched by the addition of water (25 mL) and the two layers were separated. The aqueous layer was extracted with ethyl acetate (3 × 25 mL), the organic phases were combined, extracted with brine (25 mL), and dried over Na_2SO_4. The solvent was removed under reduced pressure and the crude mixture was purified by flash column chromatography (SiO_2, hexane / Et_2O 20/1 → 5/1) and recrystallized from hexane / ethyl acetate (1/1) to obtain a white solid (**46**, 201 mg, 2.08 mmol, 34%).

The NMR spectra are in line with previously reported data.[29]

^{1}H-NMR (400 MHz, $CDCl_3$): δ 7.85 (m, 2H), 7.72 (m, 1H), 7.60 (m, 2H), 7.22 (m, 3H), 7.04 (m, 2H), 4.41 (dd, $J_{H,H}$ = 11.7 Hz, $J_{H,H}$ = 3.2 Hz 1H), 3.26 (dd, $J_{H,H}$ = 13.5 Hz, $J_{H,H}$ = 3.2 Hz 1H), 3.12 (dd, $J_{H,H}$ = 13.5 Hz, $J_{H,H}$ = 11.7 Hz 1H), 2.18 (s, 3H) ppm.

^{13}C-NMR (101 MHz, $CDCl_3$): δ 199.8, 136.6, 135.9, 134.6, 129.6, 129.4, 129.1, 128.9, 127.4, 76.8, 33.1, 32.8 ppm.

These analytical data are in agreement with previously reported characterization data for this compound.[29]

4-Fluorobenzoic anhydride (47)

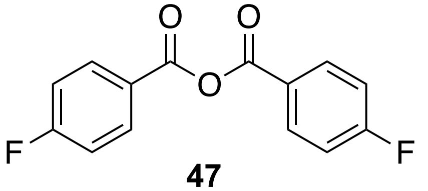

47

4-Fluorobenzoic acid (565 mg, 4.03 mmol, 1.0 eq.), K_2CO_3 (830 mg, 6.05 mmol, 1.5 eq.) and tosyl chloride (380 mg, 2.02 mmol, 0.5 eq.) were dissolved in acetonitrile (20 mL) and stirred over night at room temperature. The reaction was quenched by the addition of water (50 mL) and the two layers were separated. The aqueous layer was extracted with dichloromethane (3 × 50 mL), the organic phases were combined and dried over Na_2SO_4. The

solvent was removed under reduced pressure and the crude mixture was purified by flash column chromatography (SiO_2, cyclohexane / ethyl acetate 1/0 → 7/1) to obtain a white solid (450 mg, 1.72 mmol, 85%).

^{1}H-NMR (400 MHz, $CDCl_3$): δ 8.20 (m, 4H), 7.23 (t, $^3J_{H,H}$ = 8.6 Hz, 4H) ppm.

^{19}F{^{1}H}-NMR (376 MHz, $CDCl_3$): δ -102.06 (s, 2F) ppm.

^{13}C-NMR (101 MHz, $CDCl_3$): δ 166.9 (d, $^1J_{C,F}$ = 257.3 Hz), 161.4, 133.5 (d, $^3J_{C,F}$ = 9.7 Hz), 125.1 (d, $^4J_{C,F}$ = 3.0 Hz), 116.4 (d, $^2J_{C,F}$ = 22.3 Hz) ppm.

These analytical data are in agreement with previously reported characterization data for this compound.[30]

Hydroxy(phenyl)-iodaneyl 4-methylbenzenesulfonate (S8)

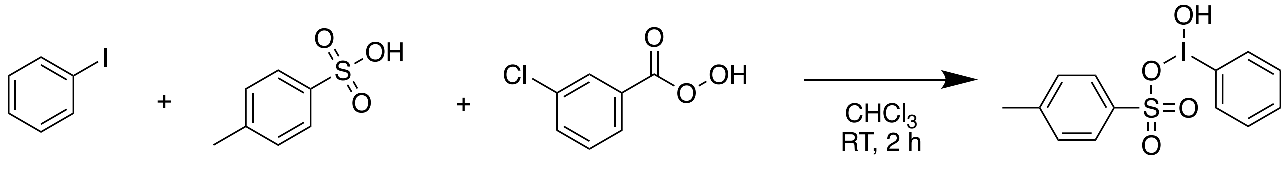

S8

Following a literature procedure,[31] iodobenzene (800 µL, 7.18 mmol, 1.0 eq.), *p*-toluenesulfonic acid monohydrate (1.50 g, 7.89 mmol, 1.0 eq.) and 3-chlorobenzoperoxoic acid (70%, 1.77 g, 7.18 mmol, 1.0 eq.) were dissolved in chloroform (8 mL) and stirred for 2 hours at room temperature. The solvent was removed and diethyl ether was added. The formed white precipitate was filtered off and washed with diethyl ether (2 ×). The white hydroxy(phenyl)-iodaneyl 4-methylbenzenesulfonate (**S8**, 2.50 g, 6.37 mmol, 89%) was dried under vacuum and used in the next step without further purification.

^{1}H-NMR (400 MHz, DMSO-d$_6$): δ 8.22 (d, $^3J_{H,H}$ 7.4 Hz, 2H), 7.71 (t, $^3J_{H,H}$ = 7.4 Hz, 1H), 7.62 (t, $^3J_{H,H}$ = 7.6 Hz, 2H), 7.47 (d, $^3J_{H,H}$ = 8.1 Hz, 2H), 7.11 (d, $^3J_{H,H}$ = 7.8 Hz, 2H), 2.29 (s, 3H) ppm.

These analytical data are in agreement with previously reported characterization data for this compound.[31]

2-Oxo-1,2-diphenylethyl 4-methylbenzenesulfonate (48)

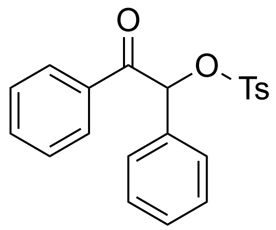

48

1,2-Diphenylethan-1-one (500 mg, 2.55 mmol, 1.0 eq.) and **S8** (1.15 g, 2.93 mmol, 1.2 eq.) were dissolved in acetonitrile (18 mL) and refluxed for 2 hours. The reaction mixture was cooled to room temperature and the solvent

was removed under reduced pressure. The crude mixture was dissolved in dichloromethane and washed with water. The organic phase was dried over Na_2SO_4 and the solvent was removed. The crude product was purified by flash column chromatography (SiO_2, cyclohexane / ethyl acetate 1/0 → 9/1) to obtain a white solid (**48**, 150 mg, 409 µmol, 16%).

^{1}H-NMR (400 MHz, $CDCl_3$): δ 7.84 (d, $^3J_{H,H}$ = 7.2 Hz, 2H), 7.72 (d, $^3J_{H,H}$ = 8.4 Hz, 2H), 7.52 (t, $^3J_{H,H}$ = 7.4 Hz, 1H), 7.41 – 7.35 (m, 4H), 7.33 – 7.27 (m, 3H), 7.22 (d, $^3J_{H,H}$ = 8.0 Hz, 2H), 6.67 (s, 1H), 2.39 (s, 3H) ppm.

^{13}C-NMR (101 MHz, $CDCl_3$): δ 192.2, 145.0, 134.1, 133.9, 133.7, 132.8, 129.8, 129.7, 129.2, 129.1, 128.8, 128.3, 128.2, 82.4, 21.8 ppm.

These analytical data are in agreement with previously reported characterization data for this compound.[32]

2-Oxo-1,2-diphenylethyl acetate (49)

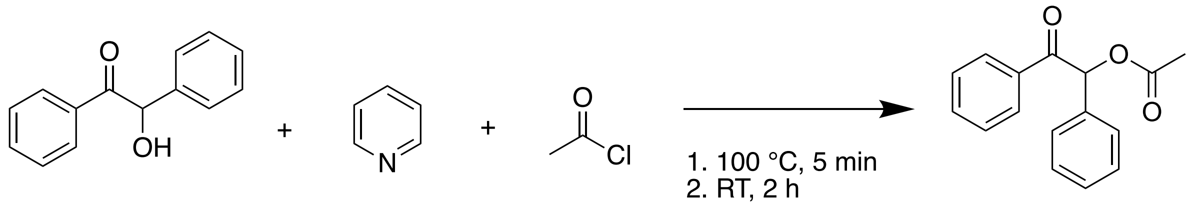

49

Benzoin (1.01 g, 4.76 mmol, 1.0 eq.) and pyridine (763 µL, 9.52 mmol, 2.0 eq.) were added in a round-bottom flask and heated with a heat gun for 5 minutes to 100 °C. The clear solution was cooled to room temperature, stirred for 15 min and acetyl choride (680 µL, 9.52 mmol, 2.0 eq.) was added slowly, heated again with a heat gun to 100 °C for 5 minutes and stirred at room temperature for 30 minutes. The resulting mixture was dissolved in dichloromethane (50 mL) and washed with aqueous sulfuric acid (2 M, 2 × 25 mL) and brine (100 mL). The organic phase was dried over Na_2SO_4 and the solvent was removed under reduced pressure. The crude product was purified by flash column chromatography (SiO_2, cyclohexane / ethyl acetate 1/0 → 20/1) to obtain 2-oxo-1,2-diphenylethyl acetate (**49**, 1.03 g, 4.05 mmol, 85%) as a pale yellow solid.

^{1}H-NMR (400 MHz, $CDCl_3$): δ 7.94 (d, $^3J_{H,H}$ = 7.1 Hz, 2H), 7.58 – 7.44 (m, 3H), 7.44 – 7.31 (m, 5H), 6.87 (s, 1H), 2.21 (s, 3H) ppm.

^{13}C-NMR (101 MHz, $CDCl_3$): δ 193.8, 170.6, 134.7, 133.7, 133.6, 129.5, 129.3, 128.9, 128.8, 128.8, 77.9, 20.9 ppm.

These analytical data are in agreement with previously reported characterization data for this compound.[33]

9-(Methylsulfonyl)-9H-carbazole (50)

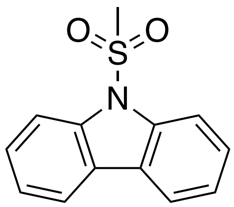

50

Carbazole (860 mg, 5.14 mmol, 1.0 eq.) and sodium hydride (60%, 600 mg, 14.9 mmol, 2.9 eq.) were dissolved in THF (15 mL) and methanesulfonyl chloride (500 µL, 6.43 mmol, 1.3 eq.) was added. The reaction mixture was stirred for 5 hours and quenched by the addition of aqueous sulfuric acid (2 M, 20 mL). The aqueous phase was extracted with dichloromethane (3 × 50 mL), the combined organic phases were dried over Na_2SO_4 and the solvent was removed under reduced pressure. The crude product was purified by flash column chromatography (SiO_2, cyclohexane / ethyl acetate 19/1 → 4/1) to obtain 9-(methylsulfonyl)-9H-carbazole (**50**, 850 mg, 3.47 mmol, 67%) as a white solid.

^{1}H-NMR (400 MHz, $CDCl_3$): δ 8.18 (td, $^3J_{H,H}$ = 8.3 Hz, $J_{H,H}$ = 1.4 Hz, 2H), 8.02 (d, $^3J_{H,H}$ = 7.6 Hz, 2H), 7.51 (t, $^3J_{H,H}$ = 8.5 Hz, $^3J_{H,H}$ = 7.3 Hz, 2H), 7.43 (t, J = 7.5 Hz, 2H), 2.98 (s, 3H) ppm.

^{13}C-NMR (101 MHz, $CDCl_3$): δ 138.5, 127.7, 126.4, 124.3, 120.3, 114.8, 38.8 ppm.

These analytical data are in agreement with previously reported characterization data for this compound.[34]

2.2 General procedures and isolated products

2.2.1 Photocatalytic reactions – general procedure

A quartz cuvette with septum cap was equipped with a magnetic stir bar and charged with substrate (e. g. 50 µmol, 1 eq.), [Cu(dap)$_2$]Cl (441 µg, 1 mol%) and 9,10-dicyanoanthracene (1.14 mg, 10 mol%). MeCN-d$_3$ (2 mL), *N,N*-diisoproylethylamine (174 µL, 20 eq.), internal standard (4-fluorotoluene, 5.5 µL, 1 eq. or mesitylene, 7.0 µL, 1 eq.) and possible additives or trapping reagents (e.g. Cs_2CO_3 or *N*-methylpyrrole) were added. The solution was purged with argon for 8 to 10 minutes, and a balloon of argon was installed on top of the cuvette (using a syringe that penetrated through the silicone septum) for the duration of the irradiation. The stirred solutions were irradiated for specified durations either with a 635 nm diode laser (Roithner Lasertechnik, 500 mW) or a 623 nm high-power LED (Thorlabs Solis-623C, 3.8 W). Product formation was monitored by ^{19}F{^{1}H}-NMR or ^{1}H-NMR spectroscopy. The conversion and the yield were determined against the internal standard.

Isolated yield: 3,6-di-tert-butyl-9H-carbazole (20-P)

A round-bottom flask with septum cap was equipped with a magnetic stir bar and charged with 3,6-di-*tert*-butyl-9-tosyl-9*H*-carbazole (95.1 mg, 219 µmol, 1 eq.), [Cu(dap)$_2$]Cl (1.85 mg, 2.09 µmol, 1 mol%) and 9,10-dicyanoanthracene (5.01 mg, 21.9 µmol, 10 mol%). Dry acetonitrile (10 mL) and *N,N*-diisoproylethylamine (670 µL, 20 eq.) were added. The solution was purged with argon for 10 minutes and a balloon of argon was installed for the duration of the reaction. The stirred solution was irradiated for 5 hours with a 623 nm high-power LED (Thorlabs Solis-623C, 3.8 W). The solvent was removed *in-vacuo* and the crude mixture was purified by flash column chromatography (SiO_2, cyclohexane / dichloromethane 1/0 → 4/1) to obtain pure 3,6-di-*tert*-butyl-9*H*-carbazole (**20-P**, 48.3 mg, 173 µmol, 79%) as off-white solid.

^{1}H-NMR (400 MHz, $CDCl_3$): δ 8.08 (d, $J_{H,H}$ = 2.0 Hz, 2H), 7.88 (s, 1H), 7.46 (dd, $J_{H,H}$ = 8.6 Hz, $J_{H,H}$ = 2.0 Hz, 2H), 7.34 (d, $J_{H,H}$ = 8.5 Hz, 2H), 1.45 (s, 18H) ppm.

^{13}C-NMR (101 MHz, $CDCl_3$): δ 142.4, 138.2, 123.7, 123.5, 116.3, 110.1, 34.8, 32.2 ppm.

These analytical data are in agreement with previously reported characterization data for this compound.[35]

Isolated yield: 4-fluoro-2-(1-methyl-1H-pyrrol-2-yl)benzonitrile (44-P)

A round-bottom flask with septum cap was equipped with a magnetic stir bar and charged with 2-bromo-4-fluorobenzonitrile (**1**, 52.1 mg, 261 µmol, 1 eq.), *N*-methylpyrrole (**44**, 454 µL, 5.21 mmol, 20 eq.), [Cu(dap)$_2$]Cl (2.04 mg, 2.31 µmol, 1 mol%) and 9,10-dicyanoanthracene (5.70 mg, 25.0 µmol, 10 mol%). Dry acetonitrile (10 mL) and *N,N*-diisoproylethylamine (227 µL, 5 eq.) were added. The solution was purged with argon for 10 minutes and a balloon of argon was installed for the duration of the reaction. The stirred solution was irradiated

for 5 hours with a 623 nm high-power LED (Thorlabs Solis-623C, 3.8 W). The solvent was removed *in-vacuo* and the crude mixture was purified by flash column chromatography (SiO_2, cyclohexane / ethyl acetate 20/1 → 5/1) to obtain 4-fluoro-2-(1-methyl-1*H*-pyrrol-2-yl)benzonitrile (**44-P**, 40.1 mg, 200 μmol, 77%) as pale yellow solid.

^{1}H{^{19}F}-NMR (500 MHz, $CDCl_3$): δ 7.74 (d, $J_{H,H}$ = 8.4 Hz, 1H), 7.11 (m, 2H), 6.82 (dd, $J_{H,H}$ = 2.7 Hz, $J_{H,H}$ = 1.8 Hz 1H), 6.45 (d, $J_{H,H}$ = 3.8 Hz, $J_{H,H}$ = 1.7 Hz 1H), 6.25 (dd, $J_{H,H}$ = 3.7 Hz, $J_{H,H}$ = 2.7 Hz 1H), 3.64 (s, 3H) ppm.

^{19}F{^{1}H}-NMR (376 MHz, $CDCl_3$): δ -103.15 (s, 1F) ppm.

^{13}C-NMR (126 MHz, $CDCl_3$): δ 164.6 (d, $^1J_{CF}$ = 246.7 Hz), 139.9 (d, $^1J_{CF}$ = 9.7 Hz), 136.0 (d, $^4J_{CF}$ = 9.9 Hz), 129.0 (s), 125.7 (s), 118.0 (d, $^3J_{CF}$ = 22.7 Hz), 115.1 (d, $^3J_{CF}$ = 22.7 Hz), 112.3 (s), 108.9 (d, $^2J_{CF}$ = 3.3 Hz), 108.7 (s), 35.1 (s) ppm.

High resolution ESI-MS (m/z) for $C_{12}H_9FN_2$+Na [**44-P**+Na]$^+$: 223.0641 (calculated: 233.0642).

These analytical data are in agreement with previously reported characterization data for this compound.[36]

For this reaction, a lower concentration of *N,N*-diisopropylethylamine than for most other substrates investigated here in this series of reactions has been used (5 instead of 20 equivalents). In the specific case considered here, the C-C coupled reaction intermediate formed when the aryl radical is intercepted by *N*-methylpyrrole is a comparatively electron-rich intermediate, which seems thermodynamically competent to re-reduce the oxidized copper catalyst (or which can potentially even donate an electron to ^{3*}DCA).[37] This could be a possible reason why a lower *N,N*-diisopropylethylamine concentration is sufficient in this specific reaction, but we did not investigate this aspect systematically.

Isolated yield: 5-fluoro-2',4',6'-trimethoxy-[1,1'-biphenyl]-2-carbonitrile (45-P)

A round-bottom flask with septum cap was equipped with a magnetic stir bar and charged with 2-bromo-4-fluorobenzonitrile (**1**, 49.2 mg, 246 μmol, 1 eq.), 1,3,5-trimethoxybenzene (**45**, 810 mg, 4.82 mmol, 20 eq.), Cs_2CO_3 (41.2 mg, 126 μmol, 0.5 eq.), [Cu(dap)$_2$]Cl (2.20 mg, 2.49 μmol, 1 mol%) and 9,10-dicyanoanthracene (5.71 mg, 25.0 μmol, 10 mol%). Dry acetonitrile (10 mL) and *N,N*-diisoproylethylamine (430 μL, 10 eq.) were added. The solution was purged with argon for 10 minutes and a balloon of argon was installed for the duration of the reaction as described above. The stirred solution was irradiated for 10 hours with a 623 nm high-power LED (Thorlabs Solis-623C, 3.8 W). The solvent was removed *in-vacuo* and the crude mixture was purified by flash column chromatography (SiO_2, cyclohexane / dichloromethane 1/0 → 3/2) to obtain 5-fluoro-2',4',6'-trimethoxy-[1,1'-biphenyl]-2-carbonitrile (**45-P**, 30.1 mg, 105 μmol, 43%) as white crystalline solid.

^{1}H-NMR (500 MHz, $CDCl_3$): δ 7.68 (dd, $J_{H,H}$ = 8.6 Hz, $J_{H,H}$ = 5.6 Hz, 1H), 7.11 (dd, $J_{H,H}$ = 9.4 Hz, $J_{H,H}$ = 2.6 Hz, 1H), 7.06 (td, $J_{H,H}$ = 8.3 Hz, $J_{H,H}$ = 2.6 Hz, 1H), 3.87 (s, 3H), 3.76 (s, 6H) ppm.

^{19}F{^{1}H}-NMR (376 MHz, $CDCl_3$): δ -105.15 (s, 1F) ppm.

^{13}**C-NMR** (126 MHz, $CDCl_3$): δ 164.4 (d, $^{1}J_{CF}$ = 254.4 Hz), 162.2, 158.2, 141.9 (d, $^{3}J_{CF}$ = 9.9 Hz), 134.5 (d, $^{3}J_{CF}$ = 9.7 Hz), 120.0 (d, $^{2}J_{CF}$ =22.2 Hz), 118.3, 114.5 (d, $^{2}J_{CF}$ = 22.7 Hz), 111.0, 90.8, 55.8, 55.4 ppm.

High resolution ESI-MS (m/z) for $C_{16}H_{14}FNO_3$+Na [**45-P**+Na]$^+$: 310.0855 (calculated: 310.0850).

2.3 Reaction optimization

2.3.1 General optimization of reaction conditions

Table S1. Optimization of photocatalytic dehalogenation reaction with red light at 20 °C. [a]

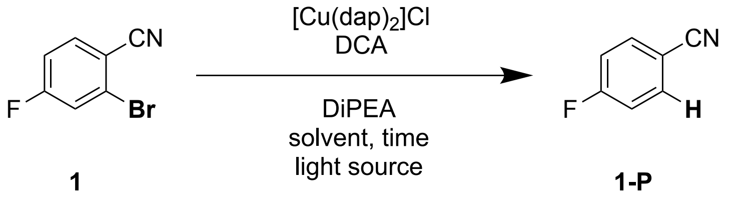

entry	$[Cu(dap)_2]^+$ / mol%	DCA / mol%	additive	time / h	light source	yield (conv.) / % [b]
1 [c]	5	10	-	6	635 nm cw-laser	81 (81)
2	2	10	-	16	635 nm cw-laser	96 (96)
3	1	10	-	16	635 nm cw-laser	91 (91)
4	2	10	-	6	635 nm cw-laser	80 (78)
5	1	10	-	6	635 nm cw-laser	(79)
6	0.5	10	-	6	635 nm cw-laser	56 (56)
7 [d]	1	10	-	6	635 nm cw-laser	70 (70)
8 [e]	1	10	-	6	635 nm cw-laser	55 (55)
9 [f]	1	10	-	6	635 nm cw-laser	45 (45)
10	1	4	-	6	635 nm cw-laser	56 (56)
11	1	10	-	6	623 nm LED	86 (87)
12	1	10	10 eq. epoxide	6	623 nm LED	90 (90)
13	1	10	0.5 eq. Bu_4NPF_6	6	623 nm LED	86 (87)
14	1	10	0.5 eq. $MgSO_4$	5	623 nm LED	82 (82)
15	1	10	0.5 eq. K_2CO_3	5	623 nm LED	93 (94)
16	1	10	2.5 eq. Cs_2CO_3	5	623 nm LED	90 (100)
17	1	10	0.5 eq. Cs_2CO_3	5	623 nm LED	95 (99)
18	1	10	2 eq. DBU	5	623 nm LED	0 (0)
19	-	10	0.5 eq. Cs_2CO_3	5	623 nm LED	0 (0)
20	1	-	0.5 eq. Cs_2CO_3	6	623 nm LED	2 (2)
21 [g]	1	10	0.5 eq. Cs_2CO_3	6	623 nm LED	0 (0)
22	1	10	0.5 eq. Cs_2CO_3	16	no light	0 (0)

a) Reaction conditions: 25 mM substrate **1** and 20 eq. DiPEA in 2 mL de-aerated MeCN-d_3. Sample irradiated in a quartz cuvette under an argon atmosphere at room temperature. b) Yields and conversions (in parentheses) were determined by quantitative $^{19}F\{^1H\}$-NMR analysis using 4-fluorotoluene as internal standard. c) Acetone-d_6 as solvent. d) 5 eq. DiPEA. e) 2 eq. DiPEA. f) Triethylamine as electron donor. g) Reaction performed in the absence of DiPEA.

2.3.2 *Conversion and yield over time*

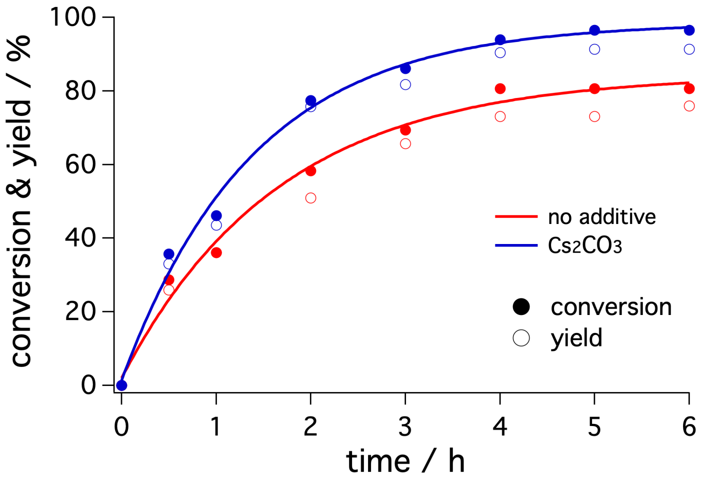

Figure S1. Conversion (filled circles) and yield (empty circles) for the dehalogenation of substrate **1** driven by red light irradiation (623 nm high power LED) measured over time. Conversion and yields were determined by quantitative $^{19}F\{^{1}H\}$-NMR analysis using 4-fluorotoluene as internal standard. Further details are provided in the text.

The reaction progress over time (Figure S1) was determined for substrate **1** with the reaction conditions given in section 2.2.1. The reaction scale given in the general procedure was triplicated and the reaction was performed in a round-bottom flask with a rubber septum under an Argon atmosphere instead of a cuvette with a silicon septum. The reaction progress was monitored for the reaction in the absence (red data points in Figure S1) and in the presence (blue data points) of 0.5 eq. of Cs_2CO_3 as additive. Conversion and yield were determined against 1-fluorotoluene as internal standard.

2.3.3 *Effect of caesium carbonate*

Conversion and yield for substrate **1** in the absence and in the presence of caesium carbonate were determined by $^{19}F\{^{1}H\}$-NMR measurements against an internal standard, and the data is presented in the Figure S1. Analysis of the spectroscopic signals by ^{1}H-NMR measurements indicates partial protonation of DiPEA over time especially in the absence of caesium carbonate (Figure S2). These changes are significantly less pronounced in the presence of caesium carbonate (Figure S3). The increasing concentration of protons over time is presumably caused by decomposition products of DiPEA,[38] resulting in a deactivation of the remaining sacrificial electron donor. In principle, protonation of $DCA^{\bullet-}$ could also occur, thereby introducing possible subsequent degradation pathways for DCA.[39] Furthermore, increasing amounts of insoluble salt – presumably caesium bromide – are observable in the dehalogenation reactions over time in the presence of caesium carbonate. The additional bromide ions liberated into the solution over the course of the photo-reactions in the absence of caesium ions might cause side reactions or catalyst decomposition of the copper complex (see also Figure S29).[40–42]

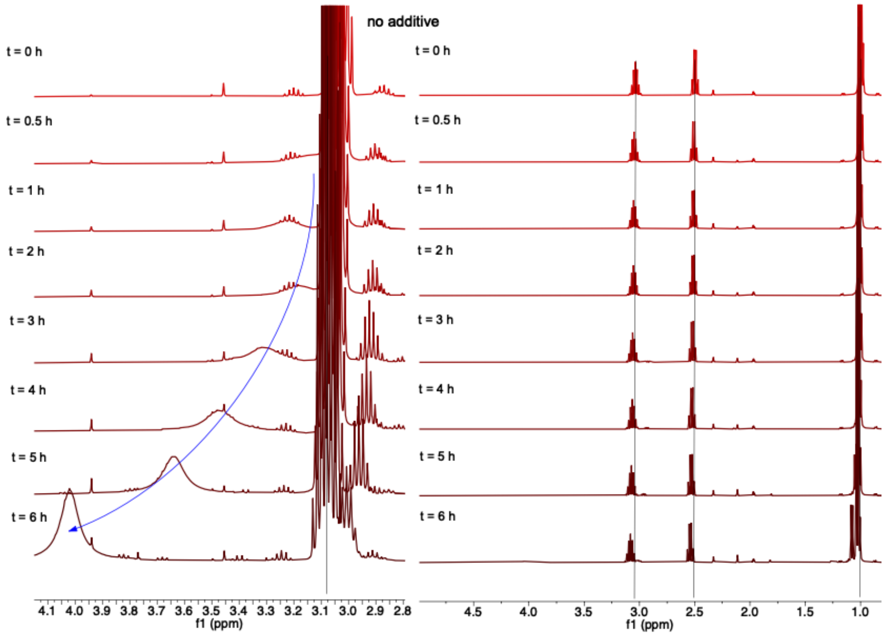

Figure S2. ^{1}H-NMR spectra of a solution containing 25 mM substrate **1**, 1 mol% [Cu(dap)$_2$]Cl, 10 mol% DCA and 20 eq. DiPEA in de-aerated MeCN-d$_3$. Sample irradiated with a high power LED (623 nm) in a stirred round-bottom flask under an Argon atmosphere at room temperature. Over time, protonation of DiPEA occurs, resulting in a shift of the ^{1}H-NMR signals of DiPEA (2.5 ppm and 3.1 ppm) as well as the appearance and shift of the new resonance associated with protonated DiPEA (3.2 ppm to 4.1 ppm).). The latter is highlighted by a blue arrow (left) and vertical straight lines marking the middle of the ^{1}H-NMR signals of DiPEA before irradiation (t = 0 h).

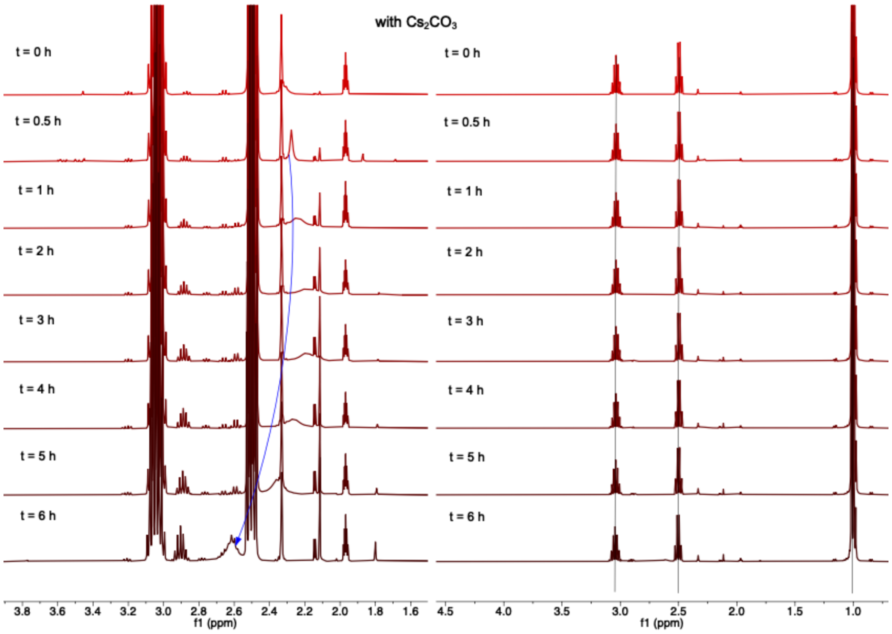

Figure S3. ^{1}H-NMR spectra of a solution containing 25 mM substrate **1**, 1 mol% [Cu(dap)$_2$]Cl, 10 mol% DCA, 20 eq. DiPEA and 0.5 eq. Cs_2CO_3 in de-aerated MeCN-d$_3$. Sample irradiated with a high power LED (623 nm) in a stirred round-bottom flask under an Argon atmosphere at room temperature. Over time, protonation of DiPEA occurs, resulting in a shift of the ^{1}H-NMR signals of DiPEA (2.5 ppm and 3.1 ppm) as well as the appearance of a new resonance associated with the protonated form of DiPEA (2.4 ppm to 2.6 ppm). The latter is highlighted by a blue arrow (left) and vertical straight lines marking the middle of the ^{1}H-NMR signals of DiPEA before irradiation (t = 0 h). In comparison to **Figure S2**, the changes of the respective shifts are significantly smaller.

3 Irradiation setup

The experimental setup used for photoredox reactions with a cw-laser as irradiation source has been published recently.[43] In this study, the light source was changed from a 447 nm cw-laser (as described previously)[43] to a 635 nm laser, but other than that, the setup remained essentially identical. The experiments performed with LED irradiation were performed using the setup shown in Figure S4.

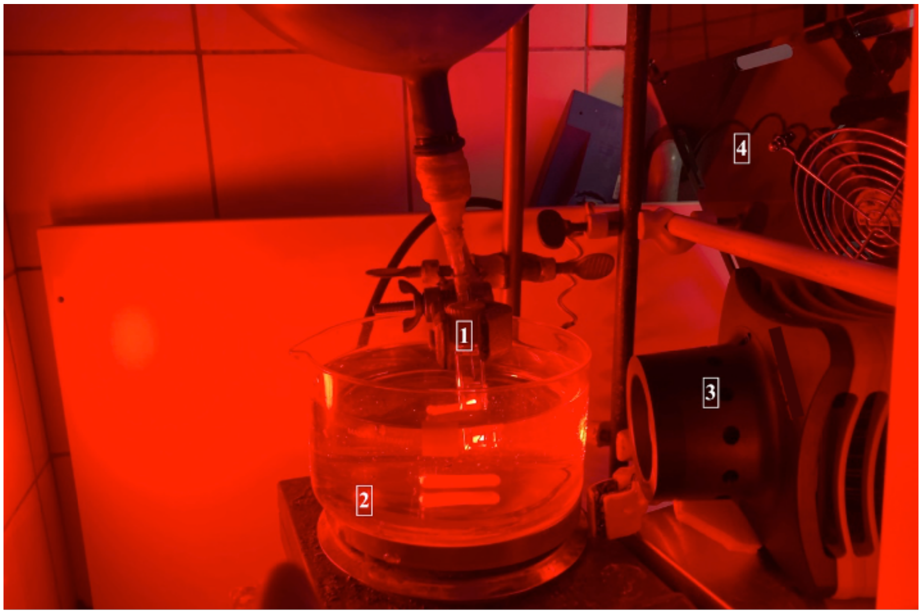

Figure S4. Irradiation setup for photoredox reactions with LED. 1: stirred screw-cap quartz cuvette with reaction mixture under Argon; 2: stirred water bath for cooling of irradiated solution; 3: LED (623 nm, 3.8 W); 4: cooling fan.

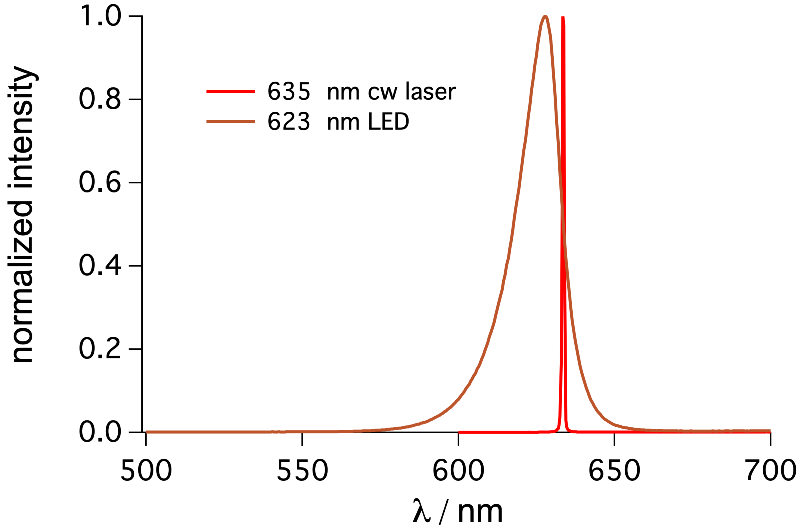

Figure S5. Emission spectra of the 623 nm LED and the 635 nm cw-laser used in this study.

4 Optical spectroscopic measurements

4.1 Optical spectroscopic properties of [Cu(dap)$_2$]Cl and DCA

4.1.1 Photophysical characterization of [Cu(dap)$_2$]Cl and DCA

All measurements were performed in de-aerated solvents at room temperature. Unless otherwise noted, acetonitrile was used as solvent for all optical spectroscopic investigations.

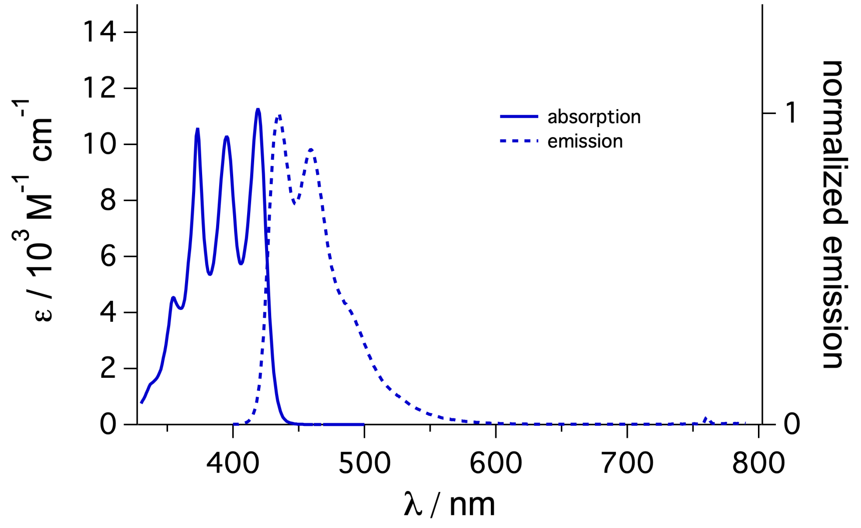

Figure S6. UV-vis absorption (solid blue trace) and emission spectra (dotted blue trace) of DCA in de-aerated acetonitrile at 20 °C. For emission measurements, excitation occurred at 380 nm.

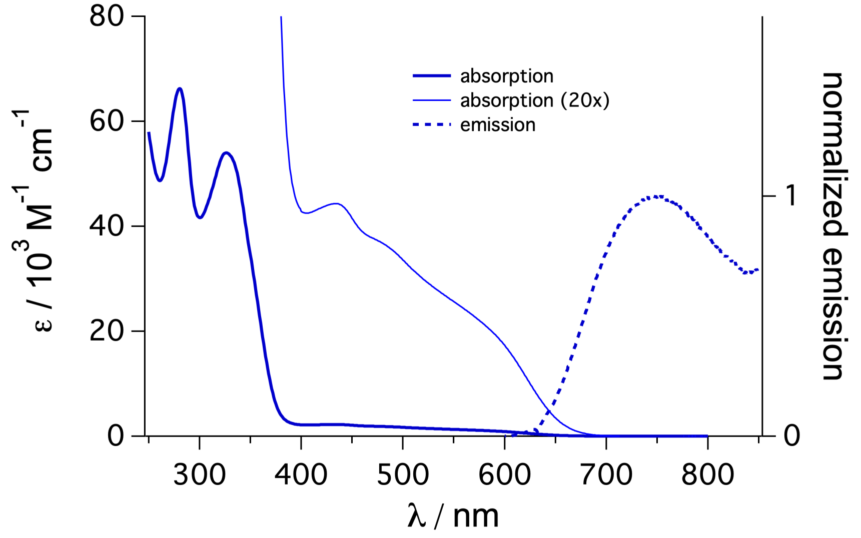

Figure S7. UV-vis absorption (solid blue traces) and emission spectra (dotted blue traces) of [Cu(dap)$_2$]Cl in de-aerated acetonitrile at 20 °C. For emission measurements, excitation occurred at 532 nm. At 623 nm, the molar extinction coefficient is 534 M^{-1} cm^{-1}.

Table S2. Overview of photophysical properties of $[Cu(dap)_2]Cl$ and DCA and some derivatives in acetonitrile at 20 °C.

**compound**	**λ_{abs} (ε) / nm (10^3 M^{-1} cm^{-1})**	**λ_{em} / nm**	**τ_0 / ns**
$[Cu(dap)_2]^+$	280 (66.2)	749	123 (75 ± 5%),
	326 (54.0)		≤ 8 (25 ± 5%)
	434 (2.2)		
DCA	373 (10.6)	434	14.7
	395 (10.3)	460	
	419 (11.3)		
^{3*}DCA [a]	440 (9.0)[44]	- [b]	10^6 [45 d]
	735 (-)[b]		
$DCA^{\bullet-}$ [c]	705 (8.4)[46]	- [b]	0.003 [47]
	640 (-)[b]		
	588 (-)[b]		
$[Cu(dap)_2]^{2+}$ [c]	287 (-)[b]	- [b]	- [b]
	358 (-)[b]		

a) Figure given in section 4.1.2. b) Values not available. c) Figure given in section 4.2.2. d) It was unclear to us in what solvent this literature value was determined.

Absorption spectra, emission spectra for $Cu(dap)_2Cl$ and DCA were similar as in prior studies.[48–50]

In principle, we would expect a mono-exponential decay of the emissive ^{3}MLCT excited state of the $[Cu(dap)_2]^+$ complex in fluid solution at room temperature, similar to what is typically observable for related Cu^I α-diimine luminophores.[51] Some of the early literature suggests that this is indeed the case for $[Cu(dap)_2]^+$,[52,53] including a more recent report.[54] In our experiments with commercial as well as with self-prepared $[Cu(dap)_2]Cl$ (synthesis according to reported procedure)[50], we consistently observed bi-exponential ^{3}MLCT excited state decays by time-resolved luminescence spectroscopy, comprised of an instrumentally limited decay component (≤ 8 ns) and a decay time of 123 ns. The latter seems compatible with the lifetime value reported previously in literature.[49] Similar bi-exponential excited state decay behaviour was observed in a previous study, but this aspect remained undiscussed.[49]

4.1.2 Transient absorption spectroscopy: reference spectrum of ^{3*}DCA

A reference spectrum of ^{3*}DCA was measured using $[Ru(bpy)_3](PF_6)_2$ as sensitizer and 500 µM of DCA with a time delay of 5 µs after the laser pulse (Figure S8). The long delay time ensures the absence of spectroscopic features caused by $^{3*}[Ru(bpy)_3]^{2+}$. The characteristic absorption bands of ^{3*}DCA with maxima around 440 nm, 670 nm and 730 nm are similar to literature reports in acetonitrile[55] or toluene.[44] At a concentration of 500 µM, the strong ground-state absorption of DCA between 370 nm and 430 nm leads to a strong filter effect, which in turn causes the unusual appearance of the transient absorption spectrum in that wavelength range.

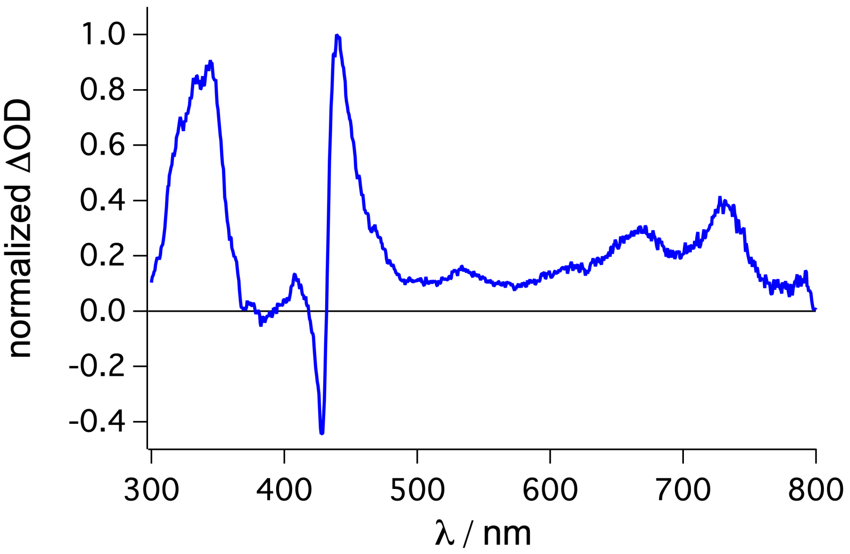

Figure S8. Normalized transient absorption spectrum for ^{3*}DCA. $[Ru(bpy)_3](PF_6)_2$ (75 µM) was excited at 500 nm with nanosecond laser pulses, and the transient absorption spectrum in the presence of 500 µM of DCA was recorded with a time delay of 5 µs in de-aerated acetonitrile at 20 °C, time-integrated over 200 ns.

4.2 Cyclic voltammetry and spectro-electrochemical study

Cyclic voltammetry was performed with a saturated calomel electrode (SCE) as reference electrode, a glassy carbon disk electrode as working electrode, and a silver wire as counter electrode. Measurements were performed in dry de-aerated acetonitrile with 0.1 M $TBAPF_6$ as an electrolyte. For oxidations or reductions with irreversible waves, the peak potential is given.

4.2.1 Cyclic voltammetry of catalysts

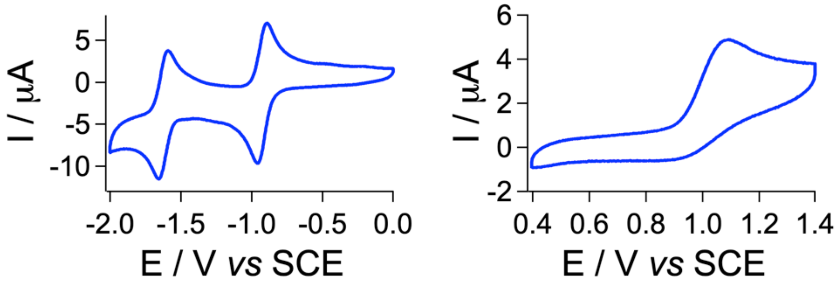

Figure S9. Cyclic voltammograms of DCA measured with $TBAPF_6$ (0.1 M) as supporting electrolyte in de-aerated acetonitrile *vs* SCE. Voltammograms were recorded with scan rate of 100 mV s^{-1}.

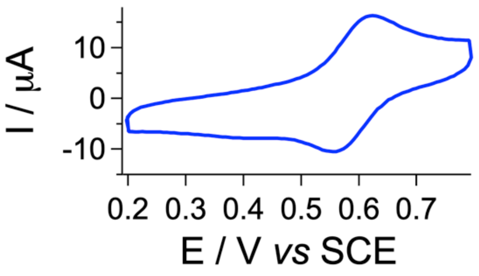

Figure S10. Cyclic voltammograms of $[Cu(dap)_2]Cl$ measured with $TBAPF_6$ (0.1 M) as supporting electrolyte in de-aerated acetonitrile *vs* SCE. Voltammogram was recorded with scan rate of 100 mV s^{-1}.

Table S3. Oxidation and reduction potentials of the two catalysts in acetonitrile reported in Volts *vs* SCE.

**catalyst**	$E_{1/2,ox}$ **/ V *vs* SCE**	$E_{1/2,red}$ **/ V *vs* SCE**
$[Cu(dap)_2]Cl$	0.59	-
DCA	1.09 [a]	-0.93
		-1.62

Conditions: Measurements in de-aerated acetonitrile *vs* SCE with 0.1 M $TBAPF_6$ and a scan rate of 100 mV s^{-1}. a) Irreversible oxidation/reduction peak. The measurements are in line with previously reported values.[53,56]

4.2.2 Spectro-electrochemical study of oxidized and reduced catalysts

Spectro-electrochemical reduction of a solution containing DCA (1 mM) and $TBAPF_6$ (0.1 M) as supporting electrolyte in de-aerated acetonitrile was performed at a potential of -1.0 V *vs* SCE. This resulted in a disappearance of the absorption bands of DCA (negative signals below 450 nm), as well as in the growth of new absorption bands with maxima at 590 nm and 640 nm and 705 nm (Figure S11). This spectrum is in line with previously published spectra for 9,10-dicyanoanthracene radical anion.[46] A spectro-electrochemical oxidation of a solution containing $[Cu(dap)_2]Cl$ (0.25 mM) and $TBAPF_6$ (0.1 M) as supporting electrolyte in de-aerated acetonitrile with an applied potential of 0.75 V *vs* SCE resulted in two new absorption bands with maxima at 388 nm and 360 nm (Figure S12).

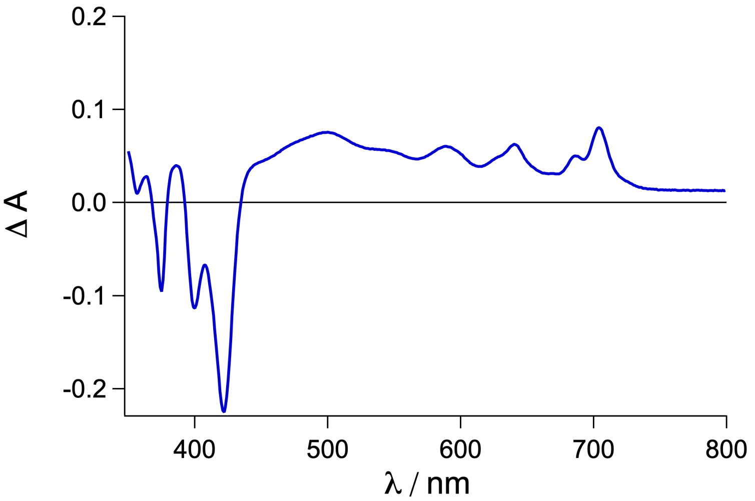

Figure S11. UV-Vis difference spectrum obtained upon electrochemical reduction of DCA in acetonitrile with an applied potential of -1.0 V vs SCE in the presence of $TBAPF_6$ (0.1 M) as supporting electrolyte under Argon. The UV-Vis spectrum without a potential applied served as baseline.

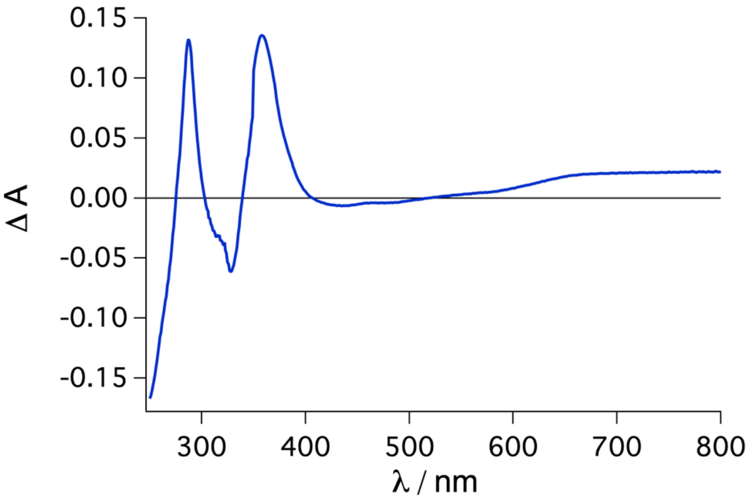

Figure S12. UV-Vis difference spectrum obtained upon electrochemical oxidation of $[Cu(dap)_2]Cl$ in de-aerated acetonitrile with an applied potential of 0.75 V *vs* SCE in the presence of $TBAPF_6$ (0.1 M) as supporting electrolyte under Argon. The UV-Vis spectrum without a potential applied served as baseline.

4.2.3 Cyclic voltammetry of substrates

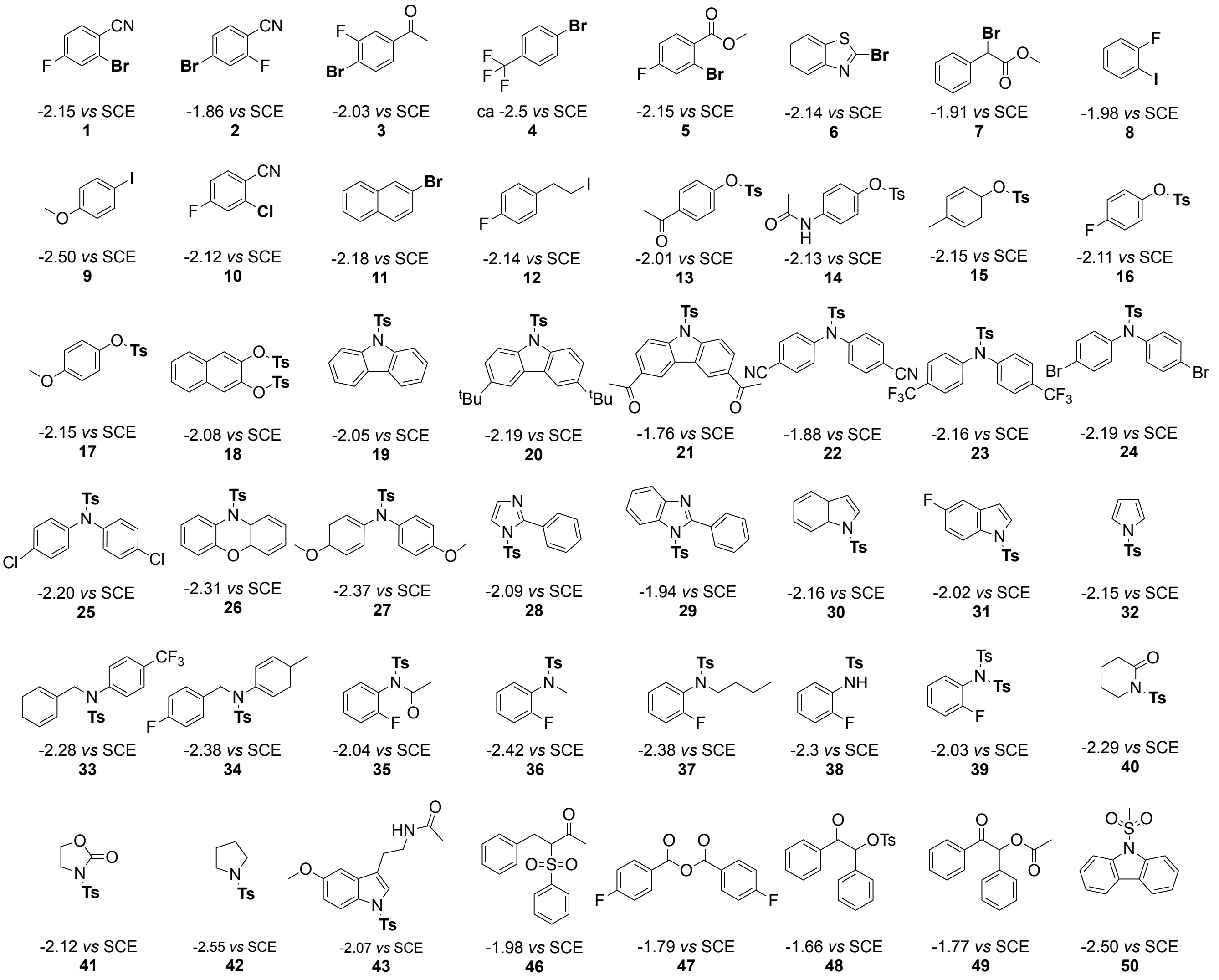

Figure S13. Overview of one-electron reduction peak potentials of all investigated substrates within this study. The respective votammograms are shown in the following figures of this section.

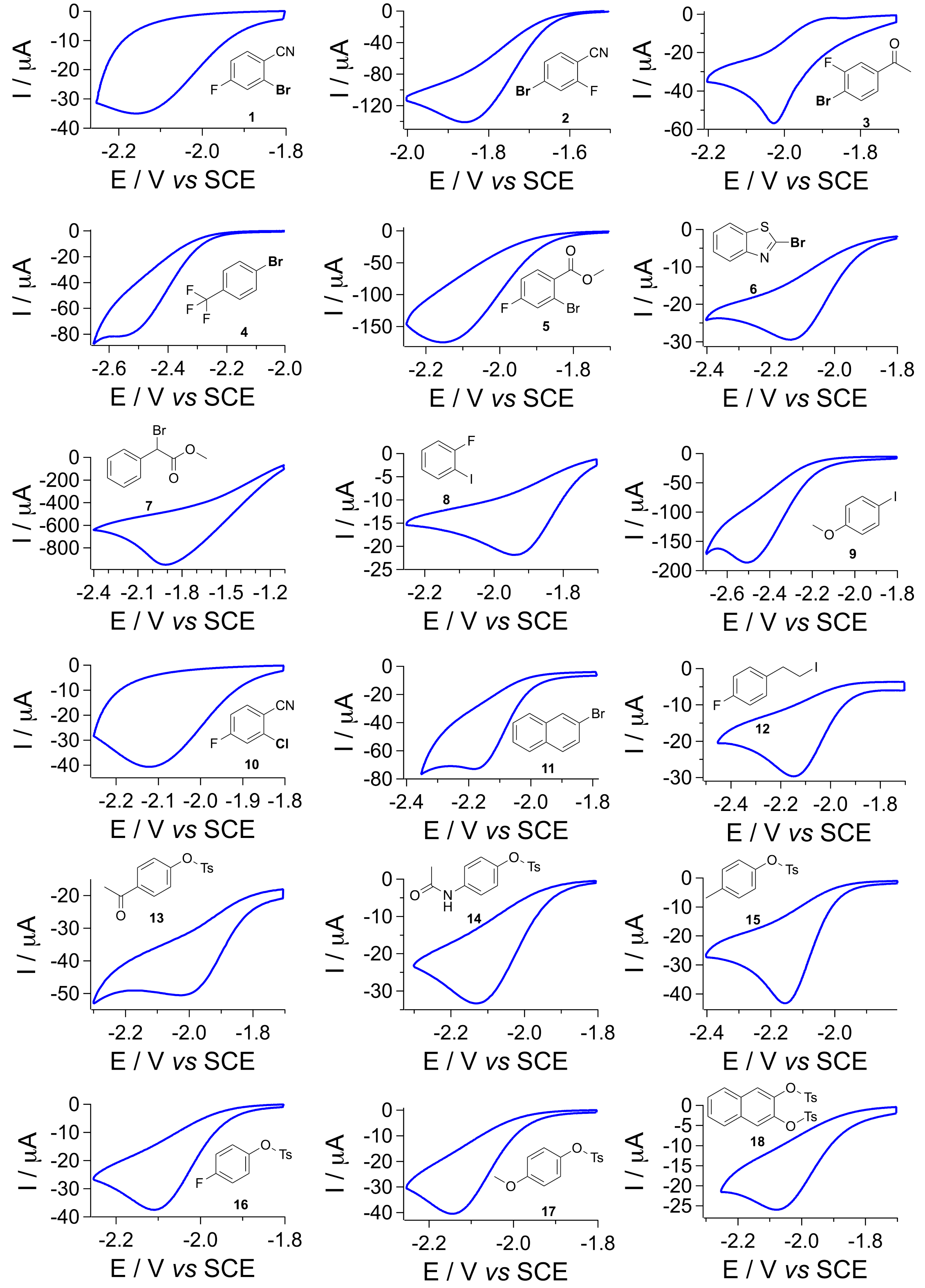

Figure S14. Cyclic voltammograms of halogenated substrates and tosylated phenolic substrates measured with $TBAPF_6$ (0.1 M) as supporting electrolyte in de-aerated acetonitrile. Voltammograms were recorded with scan rates of 100 mV s^{-1}. For the voltammogram of substrate **7,** the scan rate was 500 mV s^{-1}.

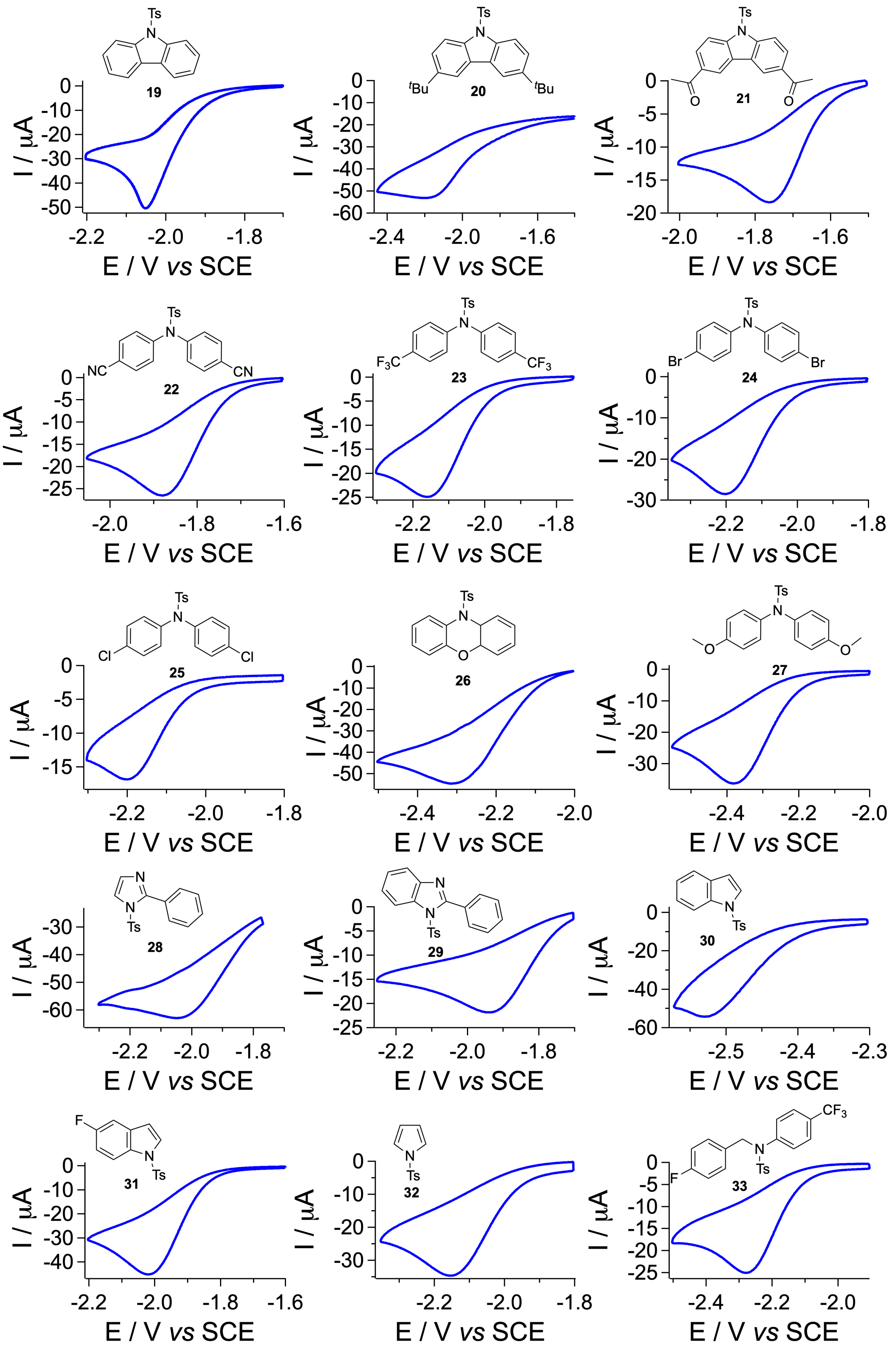

Figure S15. Cyclic voltammograms of tosylated substrates measured with $TBAPF_6$ (0.1 M) as supporting electrolyte in de-aerated acetonitrile. Voltammograms were recorded with scan rates of 100 mV s^{-1}.

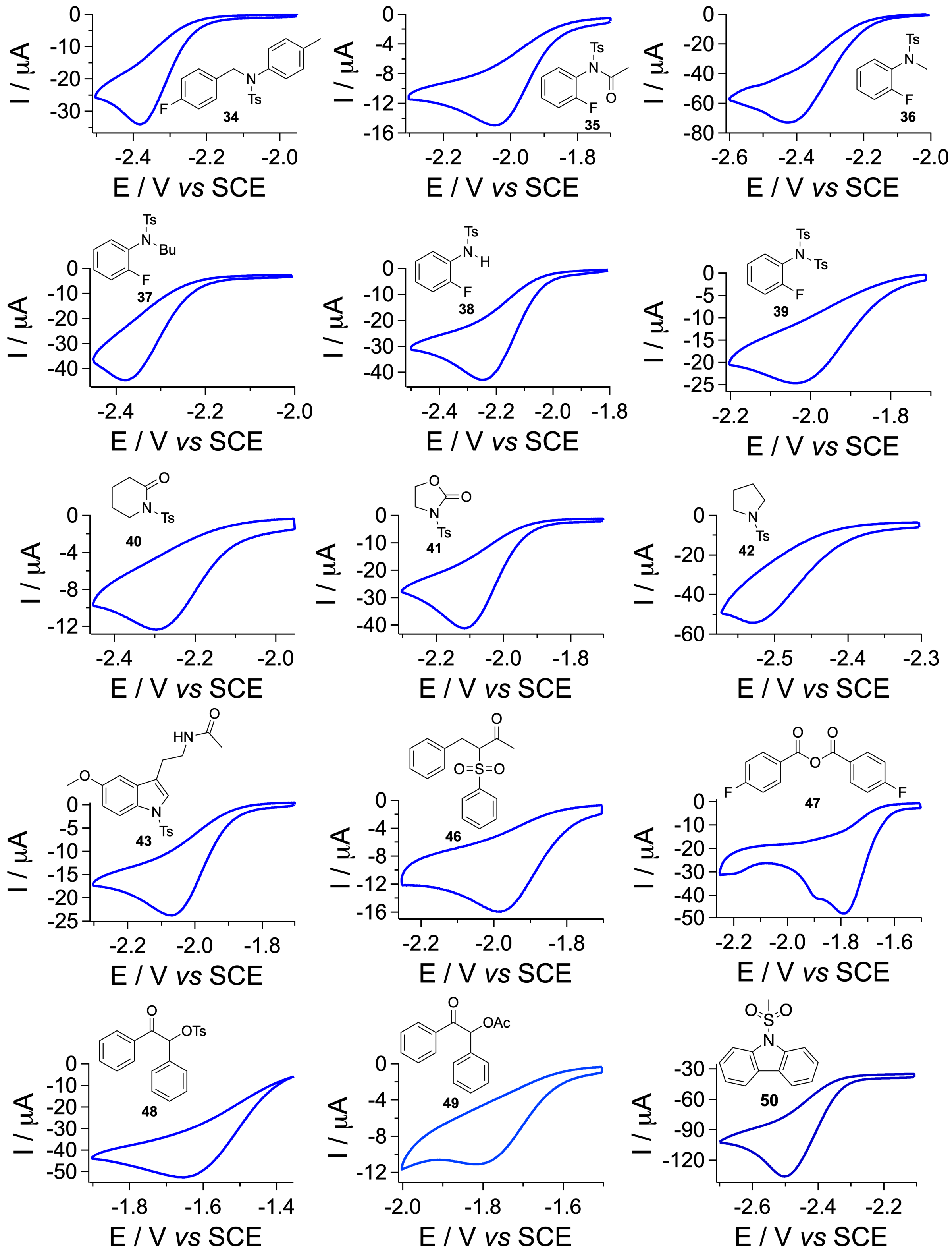

Figure S16. Cyclic voltammograms of tosylated substrates and other substrates measured with $TBAPF_6$ (0.1 M) as supporting electrolyte in de-aerated acetonitrile. Voltammograms were recorded with scan rates of 100 mV s^{-1}.

4.3 Mechanistic investigations

4.3.1 General overview

The two mechanisms from Figure 6 of the main paper are further discussed in this section. For both mechanisms additional spectroscopic data is provided.

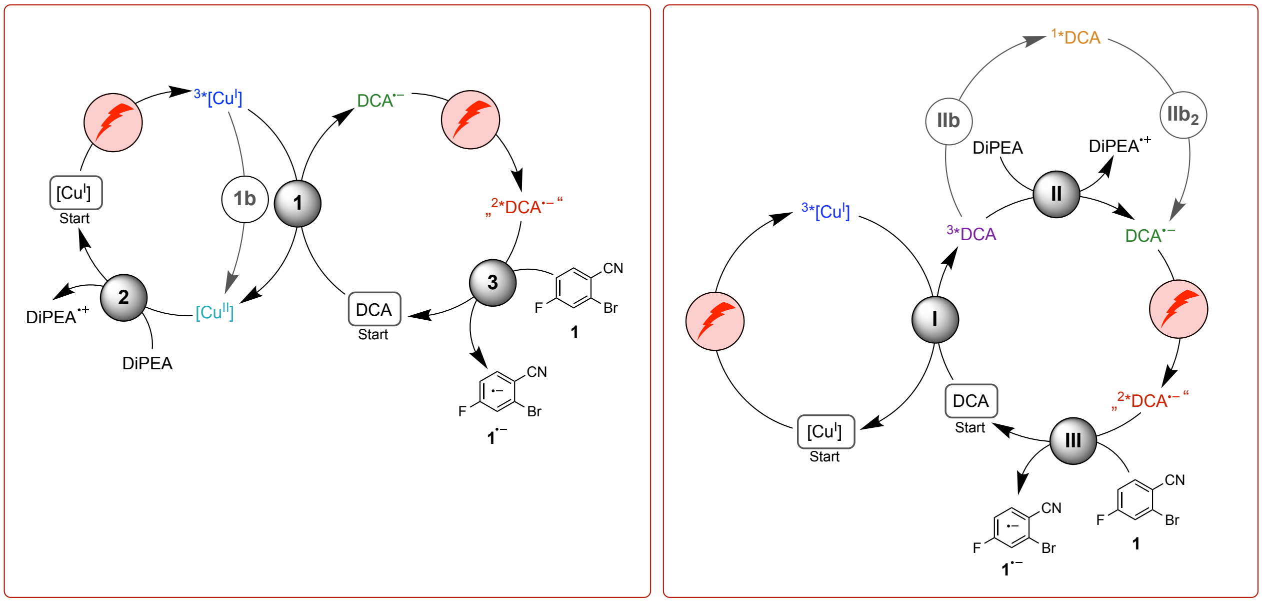

PET mechanism **TTET mechanism**

Figure S17. Two possible mechanisms for red light driven photoredox catalysis with $[Cu(dap)_2]^+$ and DCA, as in Figure 6 of the main manuscript, but here including the additional steps 1b, IIb and IIb_2.

Table S4. Rate constants (*k*) for the individual elementary processes illustrated in Figure 6 and Figure S17. All values were determined for de-aerated acetonitrile at 20 °C, unless otherwise mentioned.

mechanism	step no.	description of step	k / M^{-1} s^{-1}	Figure
PET	1	oxidative quenching of $^{3*}[Cu(dap)_2]^+$ by DCA	$\sim 6 \cdot 10^9$ [a]	Figure S20
PET/TTET	1b	oxidative quenching of $^{3*}[Cu(dap)_2]^+$ by substrate **1**	$7 \cdot 10^6$	Figure S21
PET/TTET	1c	reductive quenching of $^{3*}[Cu(dap)_2]^+$ by DiPEA	$< 10^6$	Figure S22
PET	2	electron transfer from DiPEA to $[Cu(dap)_2]^{2+}$	$\sim 10^7$	Figure S26
PET	3	electron transfer from $^{2*}DCA^{\bullet-}$ to substrate **1**	- [b]	-
TTET	I	triplet-triplet energy transfer (TTET) from $^{3*}[Cu(dap)_2]^+$ to DCA	$\sim 6 \cdot 10^9$	Figure S18
TTET	II	reductive quenching of ^{3*}DCA by DiPEA	$2.5 \cdot 10^6$ [c]	Figure S25
TTET	IIb	triplet-triplet annihilation upconversion of ^{3*}DCA	- [b]	-
TTET	III	electron transfer from $^{2*}DCA^{\bullet-}$ to substrate **1**	- [b]	-

(a) This quenching constant is estimated based on steady-state emission quenching experiments, where static as well as dynamic quenching contribute to the determined rate constant.[57] (b) These steps were not investigated in detail within this study. (c) Quenching constant determined in acetone.

In Figure S17 the two main mechanisms of our catalytic systems are reproduced from Figure 6 of the main manuscript. In the following section additional spectroscopic data is provided and discussed. All considered steps and the determined rate constants are summarized in Table S4.

4.3.2 Formation of DCA•−

As discussed in the main manuscript, our photoreaction has to start with the excitation of $[Cu(dap)_2]Cl$. In contrast to some literature reports (discussion of the literature in section 4.1.1), we found biexponential luminescence decays for $[Cu(dap)_2]Cl$, comprised of an instrumentally limited decay component of ≤ 8 ns and a second decay component of 123 ns (Figure S18) – the latter is in line with previously reported values[49] – although earlier reports reported a lifetime of 270 ns[53] (presumably with dichloromethane as solvent).[54] Excited state quenching by chloride anions can possibly lead to a static quenching contribution, causing the laser-limited decay component, as noted in the main paper.[58,59] In general, for Cu(I) complexes, exciplex formation and solvent coordination in the excited state is well known and might be another explanation for the observable biexponential emission decays.[58–60] When DCA is present, pre-association between the copper complex and DCA can potentially occur already in the electronic ground state, and then contribute to the static quenching.[61]
An accurate determination of the quenching rate constant between $[Cu(dap)_2]^+$ and DCA (ideally including distinguished rate constants for energy and electron transfer) is not trivial due to the low solubility of DCA (~0.5 mM in acetonitrile at 20 °C). Measurements of the luminescence decay and luminescence intensity resulted in quenching constants of $\sim 6 \cdot 10^9$ M^{-1} s^{-1} (based on time-resolved luminescence measurements, Figure S18) and $\sim 6 \cdot 10^9$ M^{-1} s^{-1} (based on steady-state luminescence measurements, Figure S20), using a Stern-Volmer type analysis with an inherent lifetime of 123 ns for ^{3}MLCT-excited $[Cu(dap)_2]^+$ in acetonitrile.
Kinetic transient absorption traces (Figure 7c of the main paper) recorded at 440 nm (mainly contributions from ^{3*}DCA) and at 705 nm (mainly contributions from $DCA^{\bullet -}$) have a different rate of formation. While the formation of $DCA^{\bullet -}$ is essentially laser-limited and spectroscopic signals are already visible 10 ns after the laser pulse in the transient absorption spectrum (Figure 7a of the main paper), the signal of ^{3*}DCA is growing on a longer (and not laser-limited) timescale (Figure 7a and Figure 7c of the main paper). Based on these findings it seems possible that direct PET occurs in the static process whereas TTET occurs predominantly by a dynamic quenching process. Qualitatively analogous observations for the rate of formation are made in acetone (Figure 7b and Figure 7d of the main paper). Due to the low solubility of DCA in acetonitrile at 20 °C, its exact concentration cannot be determined with sufficient accuracy to permit precise determination of the individual rate constants for static and dynamic quenching under these conditions.[57] We observed that small amounts of undissolved DCA result in scattered laser excitation light, and this can lead to an (incorrect) apparent change of the relative intensity ratio between short-lived and long-lived decay components in the respective experiments and this furthermore complicates an analysis

of the static quenching component from time-resolved data. The importance of the short-lived decay component is then inadvertently overestimated (see for example the blue trace in Figure S18), because this experiment with nanosecond time resolution fails to distinguish between scattered excitation laser light and the actual (true) sub-nanosecond excited-state decay. Overall, both (steady-state and time-resolved) luminescence quenching experiments provide a quenching constant of ~$6{\cdot}10^9$ M^{-1} s^{-1} in acetonitrile, indicating that the rates for energy and electron transfer steps are similar. Analogous experiments in acetone gave similar results as in acetonitrile (see Figure S19).

Transient absorption spectroscopy of solutions containing $[Cu(dap)_2]Cl$ in acetonitrile and various concentrations of DCA were recorded at different delay times after the laser pulse (Figure S23a and b). Expectedly, increasing DCA concentrations lead to more pronounced changes in optical density at 705 nm (see Figure S23a and b as well as the kinetic traces at the bottom part of Figure S23c, where the signal intensity between 400 ns and 800 ns increases upon increasing concentration of DCA). On a longer time-scale, the reverse electron transfer between $DCA^{\bullet-}$ and $[Cu(dap)_2]^{2+}$ is observable with a time constant of ~10 μs for the back electron transfer (Figure S23d). Focusing again on the formation step of $DCA^{\bullet-}$, the transient absorption signal at 705 nm (corresponding to $DCA^{\bullet-}$) appears instantaneously after the laser pulse (Figure S23a). In the lower part of Figure S23c, the evolution of the $DCA^{\bullet-}$ signal at 705 nm over the first 800 ns is shown for the solutions mentioned above, together with the emission kinetics of $^{3*}[Cu(dap)_2]^+$ (top, black trace). This direct comparison between the emission and transient absorption traces reveals that the formation of $DCA^{\bullet-}$ is instrumentally limited (Figure S23c, bottom) and therefore correlates with the short-lived component in the $^{3*}[Cu(dap)_2]^+$ emission decay, rather than with its long-lived (123 ns) decay component. Evidently, the formation of $DCA^{\bullet-}$ does not follow the rate of formation expected for a diffusion-controlled quenching process. In the latter case, a diffusion-limited bimolecular quenching in acetonitrile with a rate constant k_{ET} of $2{\cdot}10^{10}$ M^{-1} s^{-1}, a decay time for $^{3*}[Cu(dap)_2]^+$ of 123 ns and a DCA concentration of 0.5 mM would be expected to lead to $DCA^{\bullet-}$ formation with a time constant of 55 ns (calculated based on a rewritten Stern-Volmer equation for the determination of τ: $\tau = (1/\tau_0 + k_{ET}{\cdot}[DCA])^{-1} = (1/123\text{ ns} + 2{\cdot}10^{10}\text{ M}^{-1}\text{ s}^{-1} \cdot 0.5\text{ mM})^{-1} = 55\text{ ns}$).[43] Consequently, for a diffusion-controlled bimolecular reaction, one would expect the $DCA^{\bullet-}$ signal intensity at 705 nm to increase with a time constant of 55 ns, even if the reaction were to occur at the diffusion limit. Since the rise at 705 nm instead occurs with instrumentally limited kinetics (≤ 8 ns), it seems plausible to conclude that a static quenching elementary step between pre-aggregated $^{3*}[Cu(dap)_2]^+$ is DCA is at work. This observation supports the qualitative explanations and conclusions earlier in this section and is furthermore in line with the findings in the main manuscript.

Spectroscopic evidence for the oxidized copper complex $[Cu(dap)_2]^{2+}$, formed as a result of electron transfer to DCA is given in Figure S23 and Figure S26, and discussed in more detail in section 4.3.3.

As mentioned briefly in the main paper, triplet-triplet annihilation upconversion (step IIb in TTET mechanism of Figure S17) could potentially occur in our system, and the resulting $^{1*}DCA$ species could then be quenched by

DiPEA to result in the catalytically relevant $DCA^{\bullet-}$ species. The luminescence spectra in Figure S24 show direct evidence for the formation of $^{1*}DCA$ via upconversion, in the form of delayed fluorescence. The delayed fluorescence is substantially more intense in acetone than in acetonitrile, in line with the predominant initial TTET step in acetone and a predominant initial PET step in acetonitrile.

In the TTET mechanism of Figure 6b, $DCA^{\bullet-}$ is formed by electron transfer from DiPEA to $^{3*}DCA$ (step II in TTET mechanism of Figure S16). The kinetics for this elementary step is discussed in the following. These measurements were performed in acetone, in which TTET is the dominant initial elementary step (Figure S25). In the absence of DiPEA, $^{3*}DCA$ has decays by a combination of a first order decay pathway (corresponding to the natural decay of $^{3*}DCA$ to its electronic ground state) and a second order decay pathway (due to triplet-triplet annihilation).[43,62,63] In our case, the experimentally observable transient absorption decays at 440 nm (corresponding to $^{3*}DCA$) in the absence and the presence of DiPEA (Figure S25) were fitted with a mono-exponential decay function for simplicity. In the absence of DiPEA an approximated unquenched lifetime of τ_0 of 9.84 μs for the natural decay of $^{3*}DCA$ is obtained and a Stern-Volmer-type analysis based on this decay time of $^{3*}DCA$ resulted in a quenching constant k_{ET} of $2.5\cdot10^6$ M^{-1} s^{-1} for the triplet state quenching with DiPEA. At high concentrations of DiPEA (> 40 mM) the accumulation of $DCA^{\bullet-}$ was observable by UV-Vis spectroscopy after the laser experiments. Under photoredox catalysis conditions typically 500 mM of DiPEA are present. In this case, the efficiency η for $^{3*}DCA$ quenching by DiPEA is $\eta = 1-\tau/\tau_0 = 1 - 0.740\ \mu s\ /\ 9.84\ \mu s = 92.5\%$ ($\tau = (1/\tau_0+k_{ET}\cdot[DiPEA])^{-1} = (1/9.84\ \mu s + 2.5\cdot10^6\ M^{-1}\ s^{-1} \cdot 0.5\ M)^{-1} = 0.740\ \mu s$, same equation as introduced above).[43] An analogous analysis for the direct quenching of $^{3*}[Cu(dap)_2]^+$ by DiPEA based on a rate constant k_{ET} of $<10^6$ M^{-1} s^{-1} results in a considerably lower quenching efficiency ($\eta = 1-\tau/\tau_0 = 1 - 116\ ns\ /\ 123\ ns = <5.7\%$). As already mentioned in the main manuscript, this simple comparative analysis shows that electron transfer from DiPEA to $^{3*}DCA$ is kinetically much more favored compared to electron transfer from DiPEA to $^{3*}[Cu(dap)_2]^+$, mainly due to the much longer lifetime of $^{3*}DCA$.

Emission spectroscopy and transient absorption spectroscopy

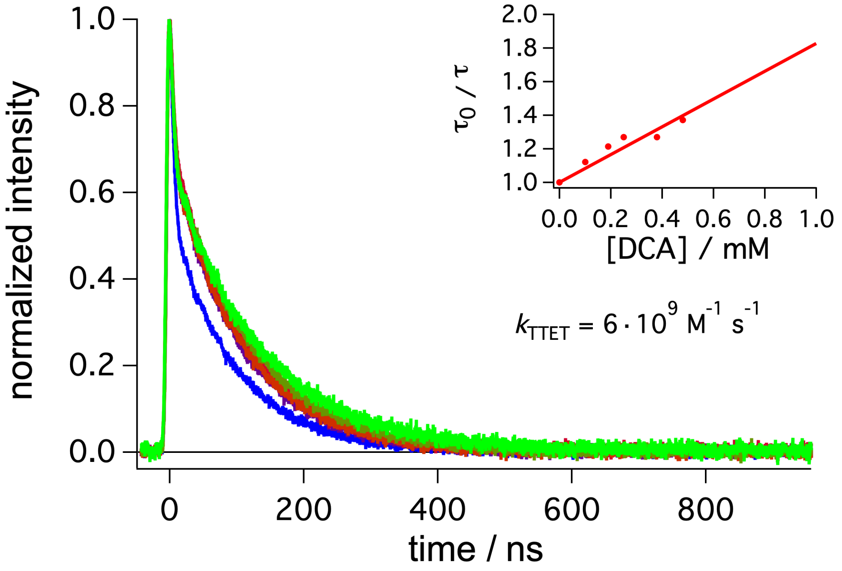

Figure S18. Luminescence quenching of [Cu(dap)$_2$]Cl by DCA in de-aerated acetonitrile at 20 °C. [Cu(dap)$_2$]Cl (100 µM) was excited at 532 nm with nanosecond laser pulses, and the emission decay was monitored at 700 nm in the absence (green) and in the presence of different concentrations of DCA (0.1, 0.19, 0.25, 0.38, 0.48 mM). The Stern-Volmer plot obtained from this data set for the long-lived decay component of a biexponential fit and the resulting bimolecular rate constant for triplet-triplet energy transfer (k_{TTET}) are given in the inset. The short-lived decay component has a lifetime of ≤ 8 ns and is laser-limited in these measurements. Further discussion is provided in the text.

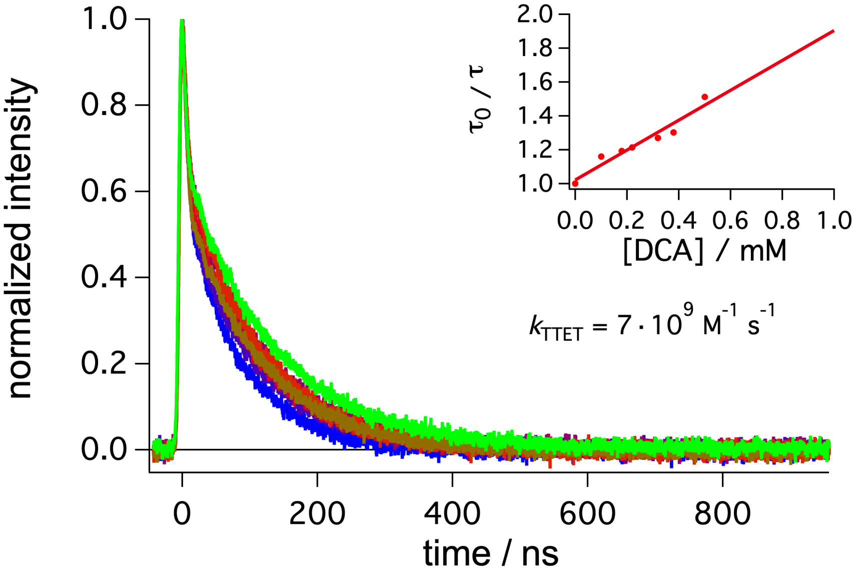

Figure S19. Luminescence quenching of [Cu(dap)$_2$]Cl by DCA in de-aerated acetone at 20 °C. [Cu(dap)$_2$]Cl (100 µM) was excited at 532 nm with nanosecond laser pulses, and the emission decay was monitored at 700 nm in the absence (green) and in the presence of different concentrations of DCA (0.1, 0.18, 0.22, 0.32, 0.38, 0.5 mM). The Stern-Volmer plot obtained from this data set for the long-lived decay component of a biexponential fit and the resulting bimolecular rate constant for triplet-triplet energy transfer (k_{TTET}) are given in the inset. The short-lived decay component has a lifetime of ≤ 8 ns and is laser-limited in these measurements.

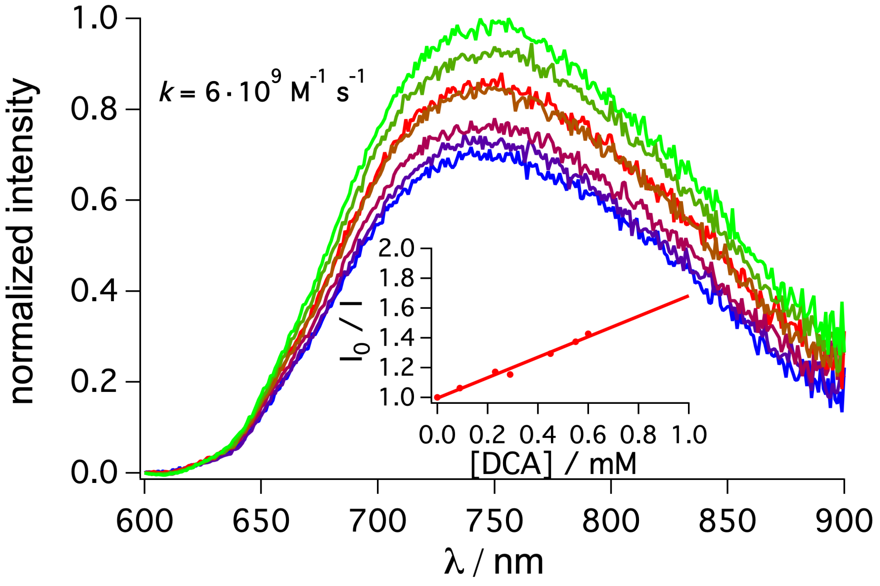

Figure S20. Steady-state emission quenching of $[Cu(dap)_2]Cl$ by DCA in de-aerated acetonitrile at 20 °C. $[Cu(dap)_2]Cl$ (100 μM) was excited at 540 nm, and the emission intensity was monitored in the absence (green) and in the presence of different concentrations of DCA (0.09, 0.23, 0.29, 0.45, 0.55, 0.60 mM). The Stern-Volmer plot obtained from this data set and the resulting quenching rate constant are given in the inset. To obtain the data points in the Stern-Volmer plot, emission spectra were converted to wavenumbers and then fitted with a Gaussian fit function. Integrals of the fit functions represent the emission intensity. A lifetime (τ_0) of 123 ns for $[Cu(dap)_2]Cl$ was used for the calculation of the quenching rate constant *k*.

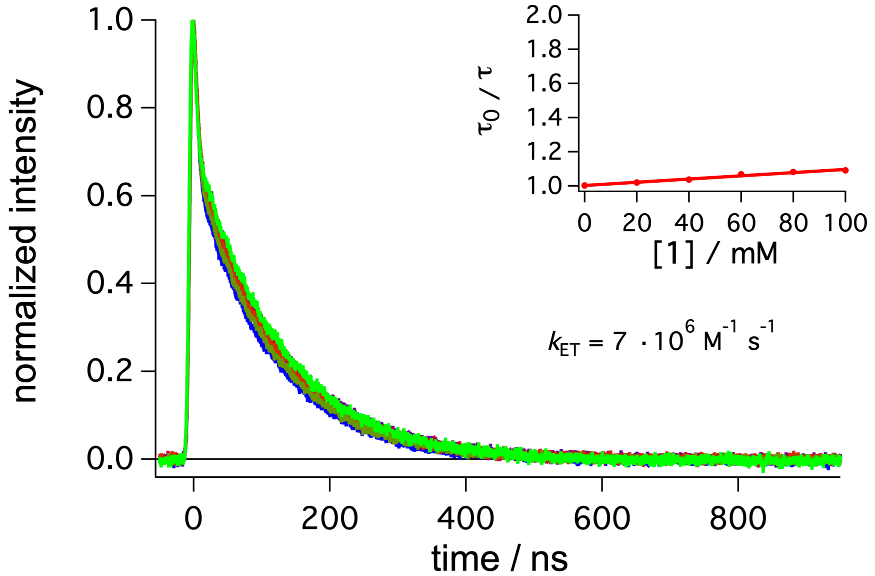

Figure S21. Luminescence quenching of $[Cu(dap)_2]Cl$ by substrate **1**. $[Cu(dap)_2]Cl$ (100 μM) in de-aerated acetonitrile at 20 °C was excited at 532 nm with nanosecond laser pulses and the emission decay was monitored at 700 nm in the absence (green) and in the presence of different concentrations of substrate **1** (20, 40, 60, 80, 100 mM). The Stern-Volmer plot obtained from this data set for the long-lived decay component of biexponential fits and the resulting bimolecular rate constant for electron transfer (k_{ET}) are given in the inset. The short-lived decay component has a lifetime of ≤ 8 ns and is laser-limited in these measurements.

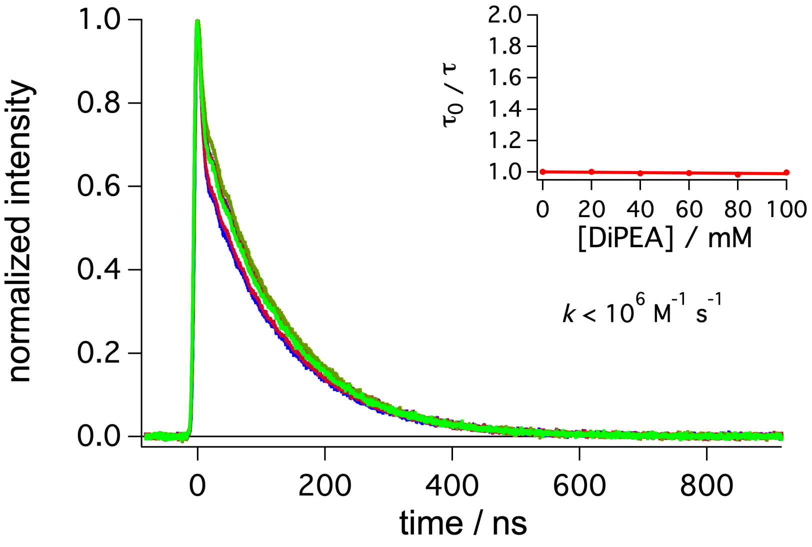

Figure S22. Luminescence decay of [Cu(dap)$_2$]Cl in the absence (light green) and in the presence of increasing concentrations of DiPEA (20, 40, 60, 80, 100 mM). [Cu(dap)$_2$]Cl (100 µM) in de-aerated acetonitrile at 20 °C. [Cu(dap)$_2$]$^+$ was excited at 532 nm with nanosecond laser pulses and the emission decay was monitored at 700 nm. The Stern-Volmer plot obtained from this data set for the long-lived decay component of biexponential fits and the resulting bimolecular rate constant (*k*) are given in the inset. The short-lived decay component has a lifetime of ≤ 8 ns and is laser-limited in these measurements.

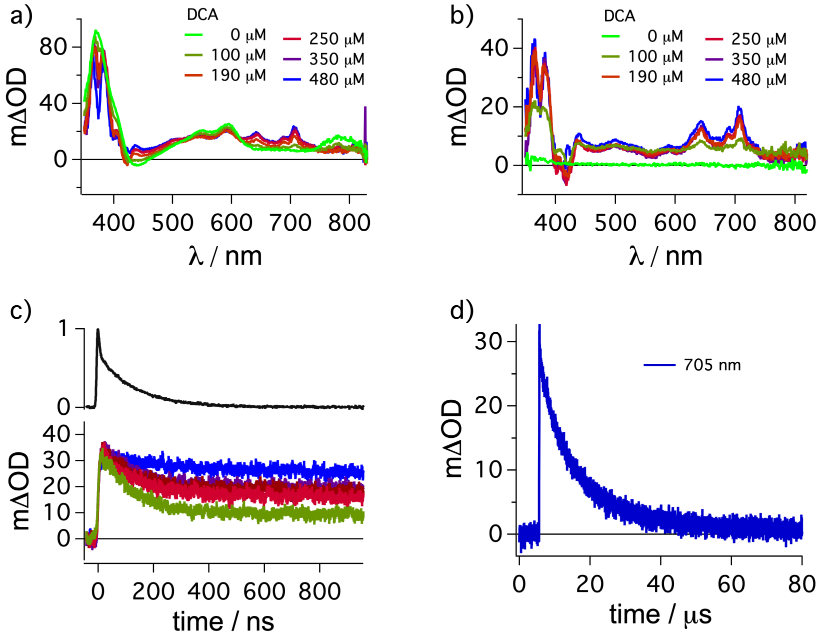

Figure S23. Transient absorption spectra and kinetic traces for excited [Cu(dap)$_2$]Cl in the presence of different concentrations of DCA in de-aerated acetonitrile at 20 °C. [Cu(dap)$_2$]Cl (100 µM) was excited at 532 nm with nanosecond laser pulses, and the transient absorption spectra in the absence (green trace) and presence of different concentrations of DCA (color code for all parts in the insets) were recorded with a time delay of 100 ns (a) and 1 µs (b), time-integrated over 200 ns. The kinetic traces at 705 nm over the first 900 ns after the laser pulse (c, bottom) and the emission decay of [Cu(dap)$_2$]Cl at 700 nm (c, top, black trace) were monitored. d) Transient absorption decay of the DCA radical anion monitored at 705 nm for the sample with 480 µM DCA.

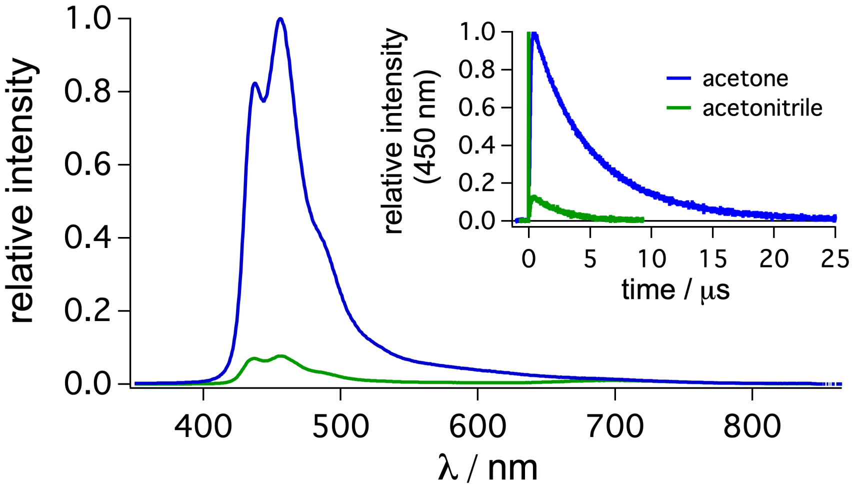

Figure S24. Relative emission intensities for $[Cu(dap)_2]Cl$ in the presence of DCA in different solvents at 20 °C. $[Cu(dap)_2]Cl$ (100 µM) was excited at 532 nm with nanosecond laser pulses in the presence of DCA (450 µM) in acetonitrile (green traces) and acetone (blue traces). Emission spectra were recorded with a time delay of 10 ns after the laser pulse and time-integrated over 30 µs. The inset displays the decay of the emission intensity at 450 nm over time. The apparent fast decay of the signal near t = 0 is attributed to scattered excitation light.

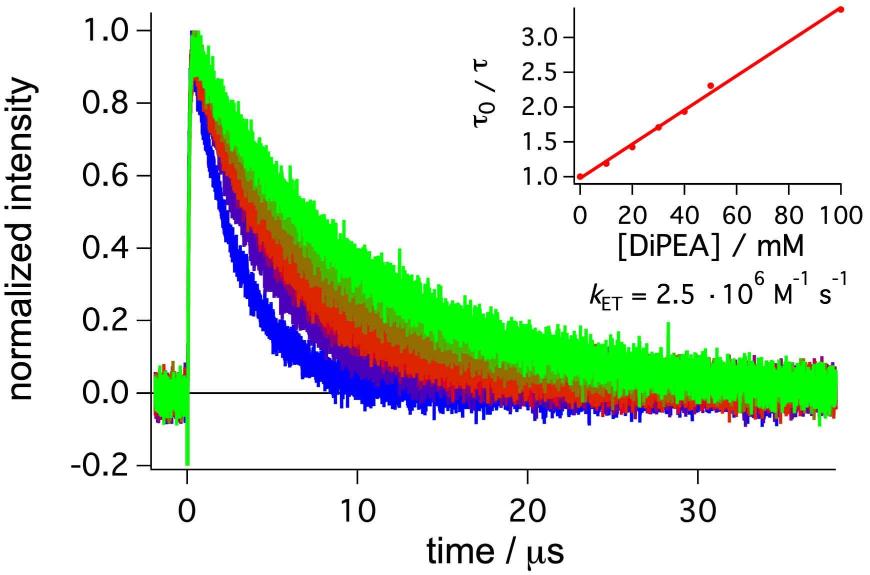

Figure S25. Reduction of ^{3*}DCA by DiPEA. $[Cu(dap)_2]Cl$ (100 µM) was excited at 532 nm with nanosecond laser pulses in the presence of DCA (500 µM) in de-aerated acetone at 20 °C. The decay of transient signals corresponding to ^{3*}DCA was monitored at 440 nm in the absence (green) and in the presence of different concentrations of DiPEA (10, 20, 30, 40, 50, 100 mM). The Stern-Volmer plot obtained based on mono-exponential decay fits and the resulting bimolecular rate constant for electron transfer to DiPEA to ^{3*}DCA (k_{ET}) are given in the inset.

4.3.3 *Reduction of [Cu(dap)$_2$]$^{2+}$ by DiPEA*

Following PET from ^{3*}[Cu(dap)$_2$]$^+$ to DCA according to the mechanism in Figure 6a of the main manuscript, the spectral signature of [Cu(dap)$_2$]$^{2+}$ and DCA$^{\bullet-}$ should be simultaneously detectable by transient absorption spectroscopy. The data in Figure S26a shows that this is indeed the case. The DCA radical anion has characteristic absorption bands in the red spectral range, which appear both in UV-Vis spectro-electrochemistry (green trace in Figure S26a, reproduced from Figure S11) and in a transient absorption measurement (red trace in Figure S26a). The spectro-electrochemical difference spectrum for the formation of [Cu(dap)$_2$]$^{2+}$ from [Cu(dap)$_2$]$^+$ (blue trace in Figure S26a, reproduced from Figure S12) shows mostly changes in the UV and blue spectral range. The transient absorption spectrum recorded after excitation of [Cu(dap)$_2$]$^+$ in the presence of DCA (red trace of Figure S26a) is essentially a linear combination of the spectro-electrochemical difference spectra obtained for DCA$^{\bullet-}$ and [Cu(dap)$_2$]$^{2+}$. This supports the PET mechanism of Figure 6a.

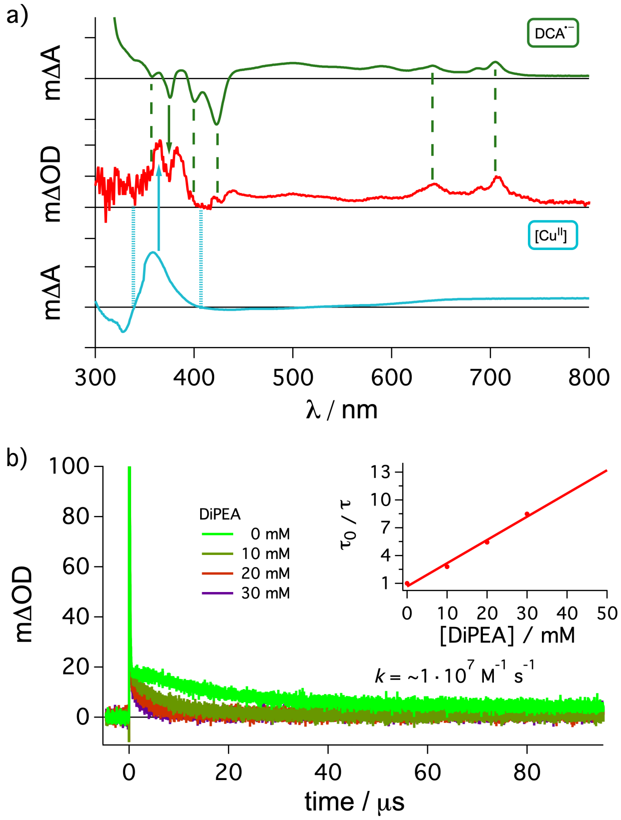

Figure S26. a) Stacked difference spectra of DCA$^{\bullet-}$ (green trace) and [Cu(dap)$_2$]$^{2+}$ (blue trace) obtained from spectro-electrochemical measurements (details in section 4.2.2), together with a transient absorption spectrum (red trace) of [Cu(dap)$_2$]Cl (100 μM) in de-aerated acetonitrile excited at 532 nm with nanosecond laser pulses in the presence of 480 μM DCA. The latter was recorded 1 μs after the laser pulses (the same data is presented in Figure S23b, blue trace). b) The disappearance of [Cu(dap)$_2$]$^{2+}$ and the recovery of [Cu(dap)$_2$]$^+$ is followed at 380 nm for stirred solutions of [Cu(dap)$_2$]Cl (100 μM) excited at 532 nm in acetonitrile with DCA (100 μM) in the absence (green) and in the presence of different concentrations of DiPEA (10, 20, 30 mM). The Stern-Volmer plot obtained from this data set and the resulting rate constant are given in the inset. The intense and - with respect to the detection window - short-lived transient absorbance in the beginning of the measurement is caused by ^{3*}[Cu(dap)$_2$]$^+$ and is therefore not taken into account within this analysis.

Based on the spectrum in Figure S26b, the disappearance of $[Cu(dap)_2]^{2+}$ can be monitored at 380 nm. Upon increasing concentration of DiPEA, the transient absorption decay at 380 nm gets faster, and a quenching constant of roughly ~10^7 M^{-1} s^{-1} for the electron transfer from DiPEA to $[Cu(dap)_2]^{2+}$ to recover $[Cu(dap)_2]^{+}$ is estimated based on an Stern-Volmer type analysis. Mono-exponential fits to the experimental kinetic traces (Figure S26b) were made. At higher concentrations of DiPEA (> 30 mM), direct flash light excitation of DCA and some accumulation of $DCA^{\bullet-}$ in solution complicate the data collection.

4.3.4 Substrate activation by $^{2*}DCA^{\bullet-}$: insights based on reactivity

In the main manuscript we discuss previous reports on excitation of $DCA^{\bullet-}$ and its stability. There has been some debate concerning the lifetime of the lowest electronically excited state of $DCA^{\bullet-}$, and a very recent study indicates that it is only ca. 3 ps in solution at room temperature.[47,64,65] Ultrafast laser spectroscopy might in principle be able to provide insights about pre-association between $DCA^{\bullet-}$ and the substrate in the ground state, or about the reactivity of the excited state,[66–68] but this is beyond the scope of this study. Indirect methods, such as the thermodynamic considerations made in the following can however provide some indirect insight into the excited-state reactivity.[69] In our system, the redox potential E_{red} of $^{2*}DCA^{\bullet-}$ can be estimated based on the equation $(^{*}E_{red}(DCA/^{2*}DCA^{\bullet-}) \approx E_{red}(DCA/DCA^{\bullet-}) - E_{0.0}(^{2*}DCA^{\bullet-}))$, with a ground state potential E_{red} of -0.93 V *vs* SCE (see section 4.2) and an energy difference $E_{0.0}$ between the ground and lowest excited doublet state of $DCA^{\bullet-}$ of ~1.7 eV. $E_{0.0}$ is calculated from on the onset of the lowest absorption band of the radical anion at ~730 nm.[47,66,70] With this data, an oxidation potential of about -2.6 V *vs* SCE can be approxiated for $^{2*}DCA^{\bullet-}$.[47]

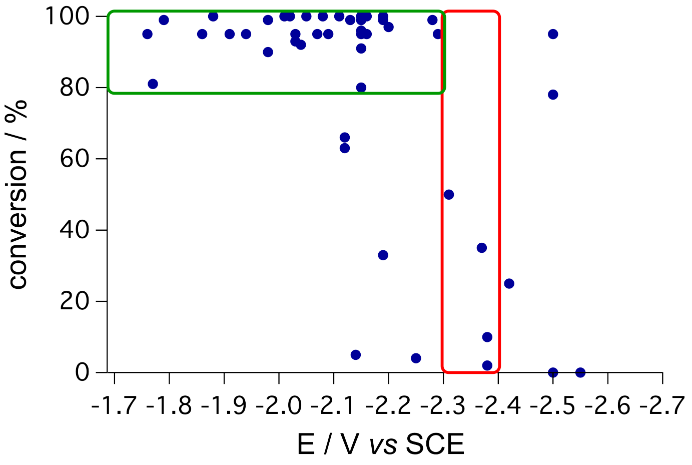

Figure S27. Conversion of substrates after light-driven reaction plotted against substrate reduction potential E_{red}. (Differences in irradiation time are not considered). The specific conversion of each substrate is given in the main manuscript and the respective reduction potentials are summarized in Figure S13.

For all investigated substrates the reduction potential has been determined by cyclic voltammetry (see section 4.2.), and the substrate conversions determined after irradiation for all substrates are plotted against these potentials (Figure S27). It is clearly visible, that almost all substrates with reduction potentials less negative than -2.3 V achieve conversions above 80% (green area in Figure S27) - and for a large majority this is the case within 5 hours of continuous irradiation. Focusing on the substrates with reduction potentials between -2.3 V and -2.4 V *vs* SCE (red area in Figure S27), significantly lower conversions are observable and substrates do not reach full conversion even with prolonged irradiation times. As this analysis ignores differences in the intrinsic reactivity and/or back-electron transfer,[71] as well as structural effects leading to possible pre-aggregation (e.g. the lack of an (electron-deficient) aromatic system in substrate **10**, **12**, **37** or **50**),[68,72] and uncertainty or errors in the determination of (irreversible) reduction potentials,[73,74] the abovementioned range between -2.3 V and -2.4 V *vs* SCE represents only an estimate. Nevertheless, this redox potential range is in reasonable agreement with the abovementioned estimated excited state redox potential of -2.6 V *vs* SCE of the DCA radical anion.

4.3.5 Stability measurements: Photostability and UV-vis analysis

Photostability of [Cu(dap)$_2$]Cl in acetonitrile

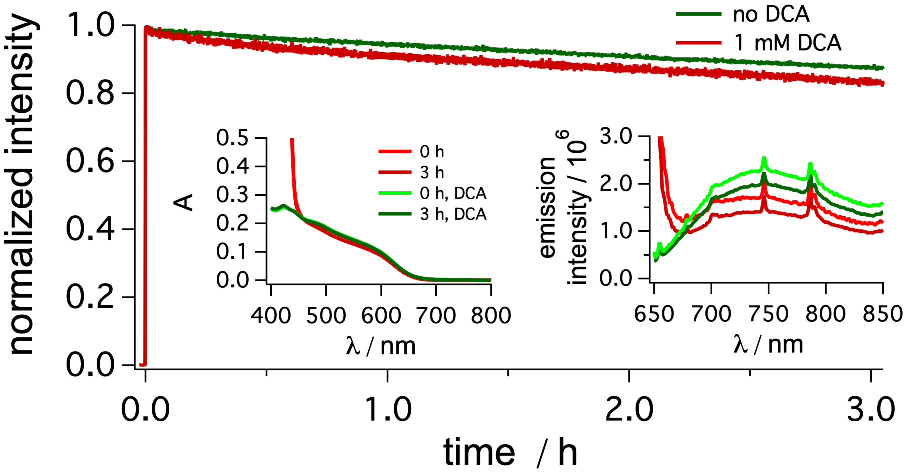

Figure S28. The emission intensity of [Cu(dap)$_2$]Cl (100 µM) in the absence (green trace) and in the presence (red trace) of 1 mM DCA under cw-laser irradiation (635 nm, 500 mW) is detected over time in acetonitrile at 20 °C. The main plot represents the normalized emission intensity detected at 750 nm, while the insets display the recorded UV-vis absorption (left inset) and emission spectra (right inset). In the emission spectra small peaks caused by scattered excitation light are visible.

We investigated the photostability of [Cu(dap)$_2$]Cl to gain insights about possible degradation of the copper complex over time and the influence of the presence of DCA. The general setup for the measurements has been reported previously.[63] Detection of the emission intensity over several hours (Figure S28) reveals only small differences between the data set in the presence of DCA (red trace) compared to the dataset in the absence of DCA (green dataset). After 3 hours of continuous irradiation, over 80% of the initial emission intensity is still observable

in both cases, indicating that the intrinsic photostability of the $[Cu(dap)_2]^+$ allows an application in photocatalysis with irradiation times of several hours.

Titration of $[Cu(dap)_2]Cl$ with bromide ions

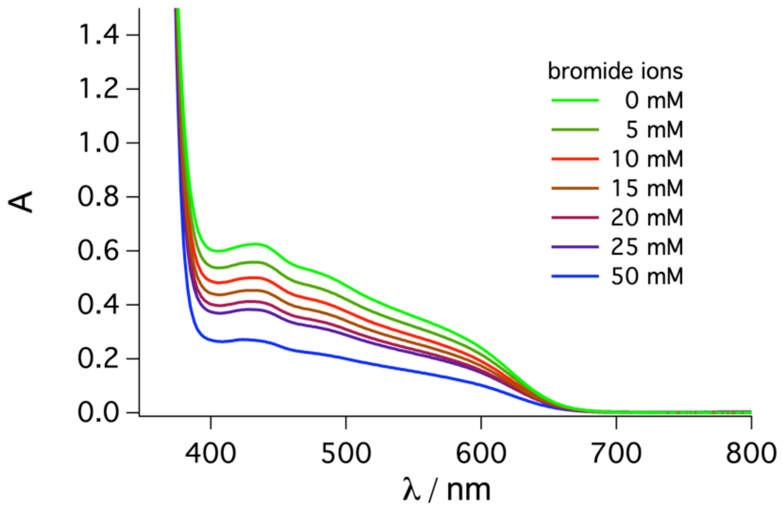

Figure S29. UV-vis absorption spectra of $[Cu(dap)_2]Cl$ (250 μM with different concentrations of tetra-*n*-butylammonium bromide in acetonitrile at 20 °C. The given bromide concentrations are presented in the inset.

4.3.6 Proposed mechanism for detosylation reactions

In Figure 6 of the main manuscript, a mechanistic proposal for the hydrodehalogenation of substrate **1** is given as as an example for a reductive dehalogenation reaction. An analogous mechanistic proposal is made here for the detosylation reaction of substrate **19**, as exemplary case. This proposed detosylation mechanism is in accordance with the literature.[16,38,75,76]

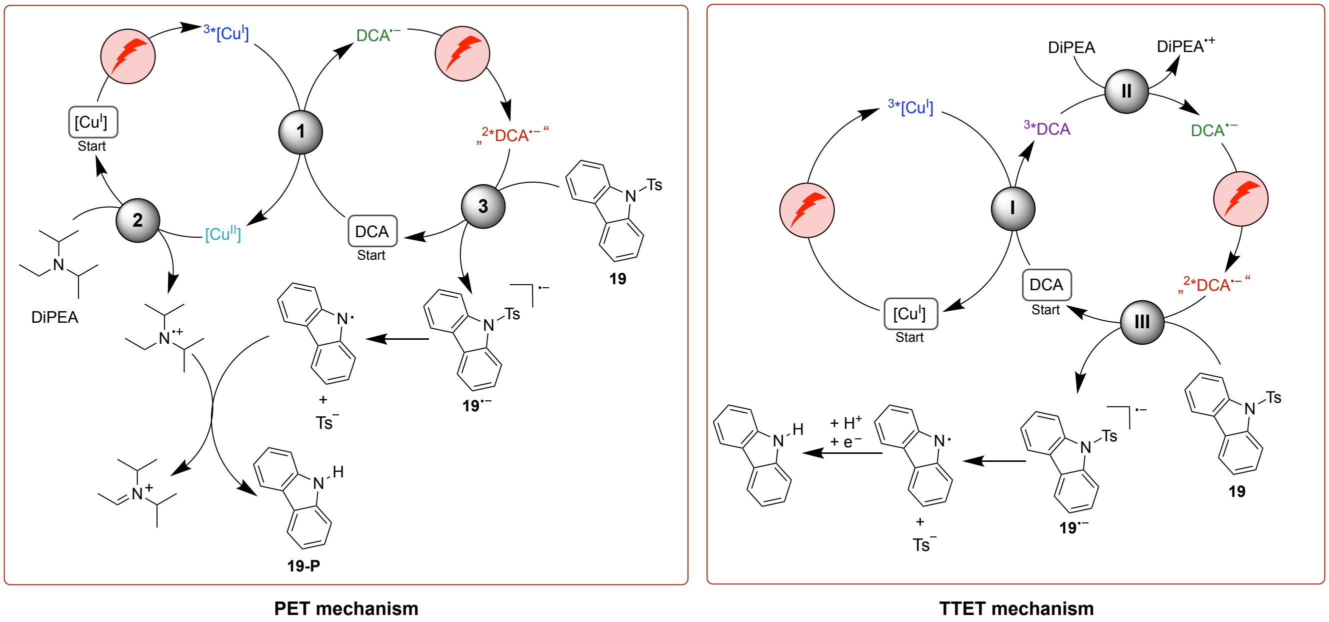

Figure S30. Plausible reaction mechanism for the detosylation reaction based on an initial photoinduced electron transfer (PET) step between ^{3}MLCT-excited [Cu(dap)$_2$]Cl and 9,10-dicyanoanthracene (DCA). Gray circles mark the elementary reaction steps of (1) oxidative quenching of ^{3*}[Cu(dap)$_2$]$^+$ (abbreviated as ^{3*}[Cu^I]) by DCA, (2) spontaneous reduction of the oxidized copper photocatalyst ([Cu^{II}]) by DiPEA, and (3) substrate activation after excitation of the DCA radical anion. (b) Reaction mechanism based on an initial triplet-triplet energy transfer (TTET) step between ^{3}MLCT-excited [Cu(dap)$_2$]Cl and DCA. Gray circles mark the elementary reaction steps of (I) TTET, (II) reductive quenching of ^{3*}DCA by DiPEA, and (III) substrate activation after excitation of DCA$^{•–}$. The doublet excited state of that radical anion is extremely short-lived,[47] and therefore ^{2*}DCA$^{•–}$ is set in quotation marks, to emphasize the possibility that the photoreaction could in fact predominantly occur from pre-aggregated DCA$^{•–}$/substrate encounter complexes, or could even involve some DCA photo-degradation products.

5 NMR data

All fluorinated substrates were analysed by $^{19}F\{^{1}H\}$-NMR spectroscopy. For all other substrates, ^{1}H-NMR spectroscopy was applied to determine conversion and yield against an internal standard.[27] In most cases the product is easily detectable by ^{1}H-NMR spectroscopy (e.g. substrate **6**) and reference spectra are readily available in the literature; for all other cases a product reference spectrum is included for comparison (e.g. substrate **11**). An experimental uncertainty of around 5% is estimated for the determined conversions and yields with our method. In general, all detected signals corresponding to the staring material **(X)**, the product (**X-P**) and internal standard (**IS**) are labelled within the respective figures.

For detosylation reactions, signals corresponding to the cleaved tosyl group furthermore become detectable. While a detailed analysis of decomposition pathways of the tosyl group is beyond the scope of this paper, in some cases *p*-toluensulfonic acid (^{1}H-NMR (400 MHz, CD_3CN): δ 7.76 ($^{3}J_{H,H}$ = 8.1 Hz, 2H), 7.39 (t, $^{3}J_{H,H}$ = 8.1 Hz, 2H), 4.50 (br, 1H), 2.42 (s, 3H) ppm) can be detected (e.g. substrates **21**, **25** or **48**), while in other cases two signals around ~7.25 ppm and ~7.55 ppm, corresponding to an unknown side product, appear (e.g. substrates **13**, **15**, **17**, **24**).

In the special case of substrate **18** (naphthalene-2,3-diyl bis(4-methylbenzenesulfonate), a partial deprotonation of the product was observed by DiPEA in the reaction mixture, and therefore the ^{1}H-NMR shifts of this sample are not matching to a reference sample. The formation of the desired product was confirmed by comparison to a sample of 2,3-naphthalenediol recorded in the presence of DiPEA (Figure S48).

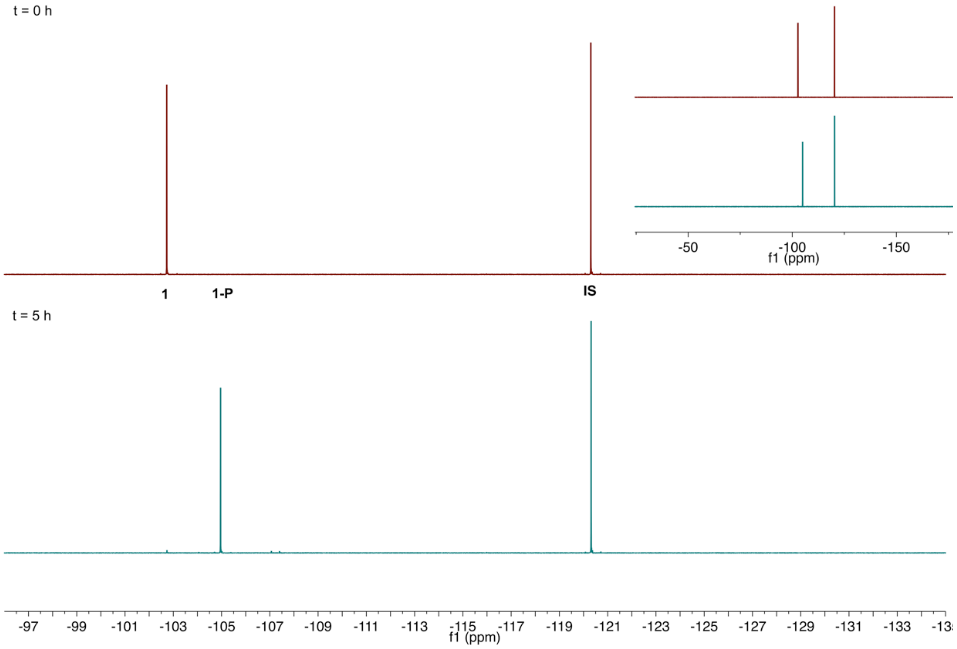

Figure S31. $^{19}F\{^{1}H\}$-NMR spectra monitoring the light-driven reaction progress of substrate **1** over time using a 623 nm high power LED irradiation. Reaction conditions: 25 mM substrate, 1 mol% $[Cu(dap)_2]Cl$, 10 mol% DCA, 0.5 eq Cs_2CO_3 and 20 eq. DiPEA in MeCN-d_3; IS = internal standard (4-fluorotoluene). The inset presents the same spectra (in the same colours) as the main part of the figure over a wider ppm range.

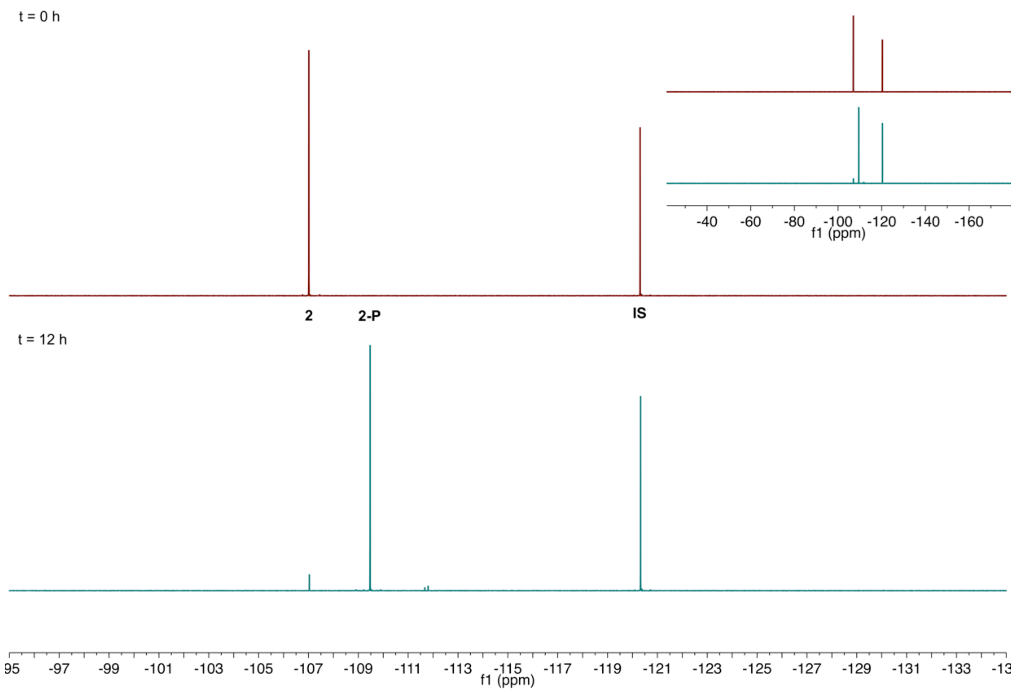

Figure S32. $^{19}F\{^{1}H\}$-NMR spectra monitoring the light-driven reaction progress of substrate **2** over time using a 623 nm high power LED irradiation. Reaction conditions: 25 mM substrate, 1 mol% $[Cu(dap)_2]Cl$, 10 mol% DCA, 0.5 eq Cs_2CO_3 and 20 eq. DiPEA in MeCN-d_3; IS = internal standard (4-fluorotoluene). The inset presents the same spectra (in the same colours) as the main part over a wider ppm range.

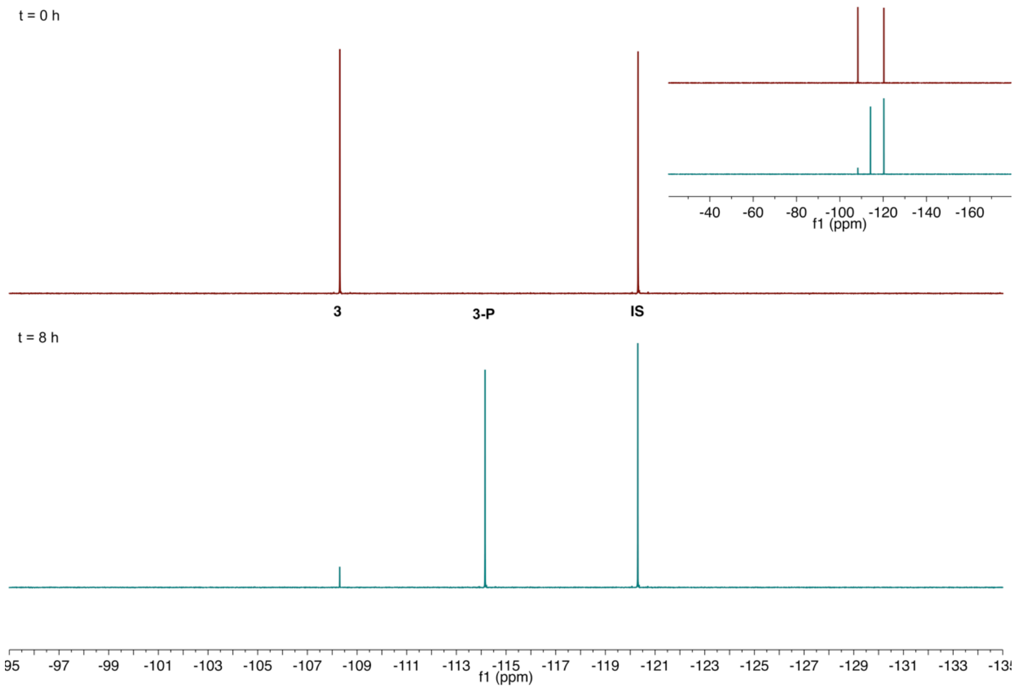

Figure S33. $^{19}F\{^{1}H\}$-NMR spectra monitoring the light-driven reaction progress of substrate **3** over time using a 623 nm high power LED irradiation. Reaction conditions: 25 mM substrate, 1 mol% $[Cu(dap)_2]Cl$, 10 mol% DCA, 0.5 eq Cs_2CO_3 and 20 eq. DiPEA in MeCN-d_3; IS = internal standard (4-fluorotoluene). The inset presents the same spectra (in the same colours) as the main part over a wider ppm range.

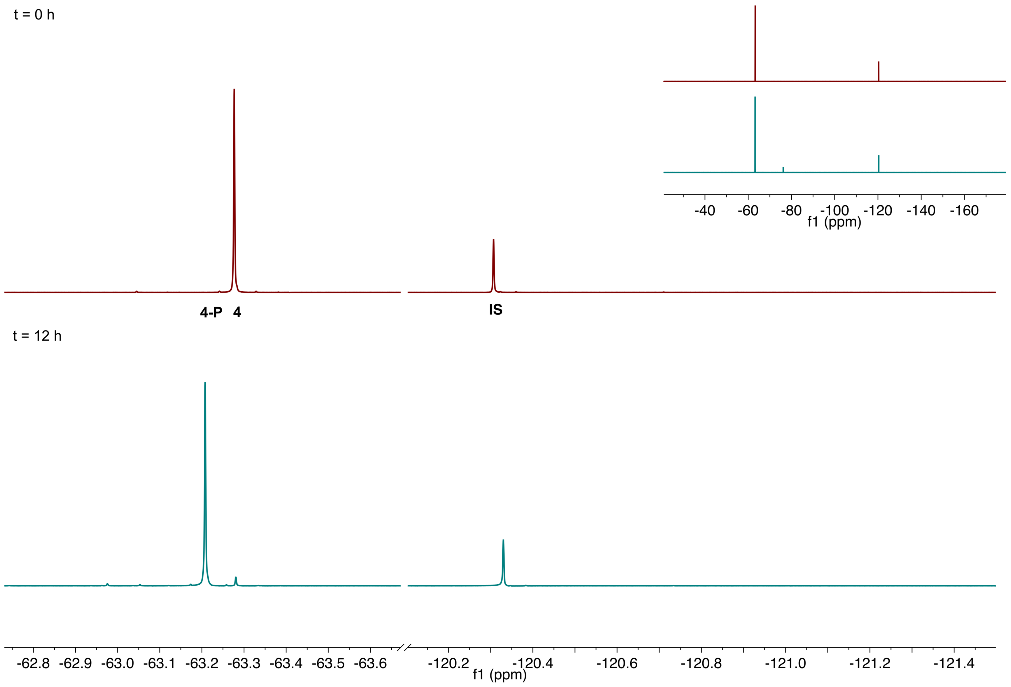

Figure S34. $^{19}F\{^{1}H\}$-NMR spectra monitoring the light-driven reaction progress of substrate **4** over time using a 623 nm high power LED irradiation. Reaction conditions: 25 mM substrate, 1 mol% $[Cu(dap)_2]Cl$, 10 mol% DCA, 0.5 eq Cs_2CO_3 and 20 eq. DiPEA in MeCN-d_3; IS = internal standard (4-fluorotoluene). The inset presents the same spectra (in the same colours) as the main part over a wider range.

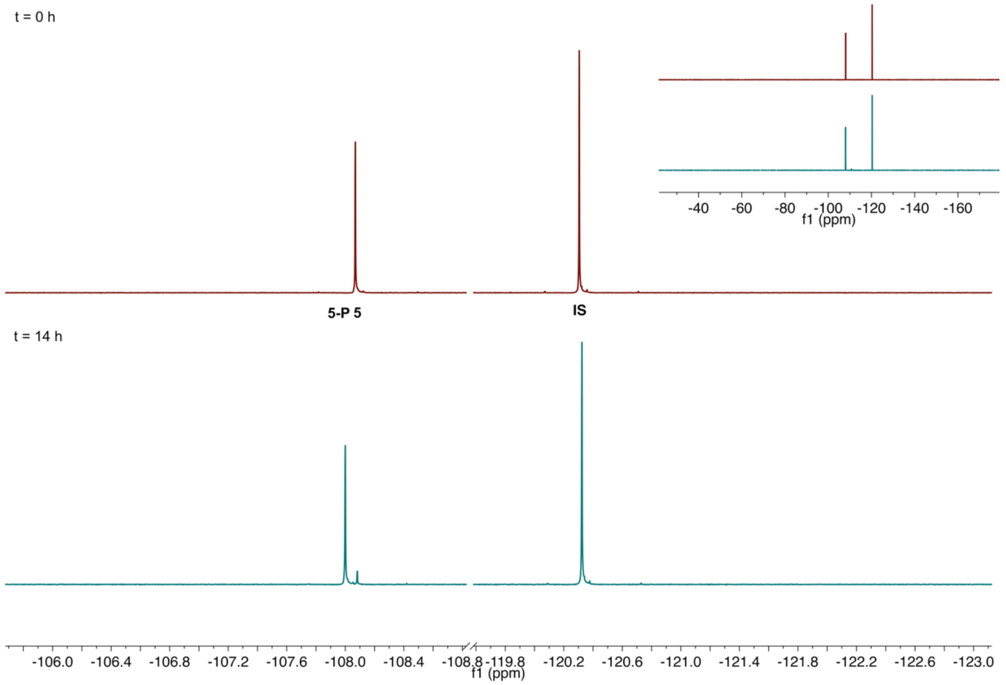

Figure S35. $^{19}F\{^{1}H\}$-NMR spectra monitoring the light-driven reaction progress of substrate **5** over time using a 623 nm high power LED irradiation. Reaction conditions: 25 mM substrate, 1 mol% $[Cu(dap)_2]Cl$, 10 mol% DCA, 0.5 eq Cs_2CO_3 and 20 eq. DiPEA in MeCN-d_3; IS = internal standard (4-fluorotoluene). The inset presents the same spectra (in the same colours) as the main part over a wider ppm range.

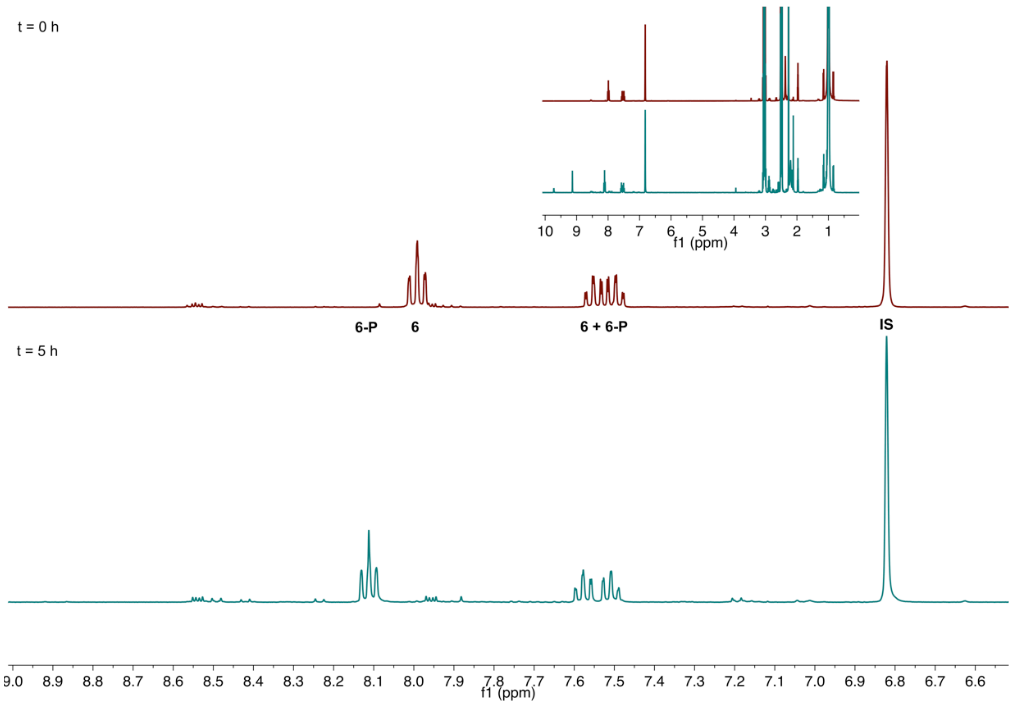

Figure S36. $^{1}H\{^{1}H\}$-NMR spectra monitoring the light-driven reaction progress of substrate **6** over time using a 623 nm high power LED irradiation. Reaction conditions: 25 mM substrate, 1 mol% $[Cu(dap)_2]Cl$, 10 mol% DCA, 0.5 eq Cs_2CO_3 and 20 eq. DiPEA in MeCN-d_3; IS = internal standard (mesitylene). The inset presents the same spectra (in the same colours) as the main part over a wider ppm range.

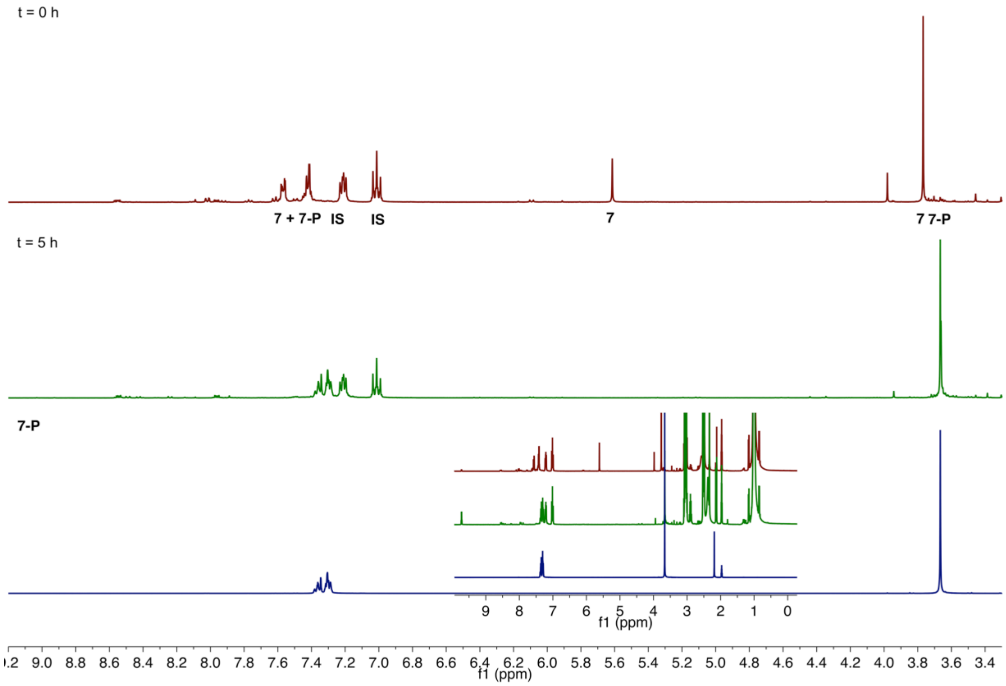

Figure S37. $^{1}H\{^{1}H\}$-NMR spectra monitoring the light-driven reaction progress of substrate **7** over time using a 623 nm high power LED irradiation. Reaction conditions: 25 mM substrate, 1 mol% $[Cu(dap)_2]Cl$, 10 mol% DCA, 0.5 eq Cs_2CO_3 and 20 eq. DiPEA in MeCN-d_3; IS = internal standard (4-fluorotoluene). The inset presents the same spectra (in the same colours) as the main part over a wider ppm range.

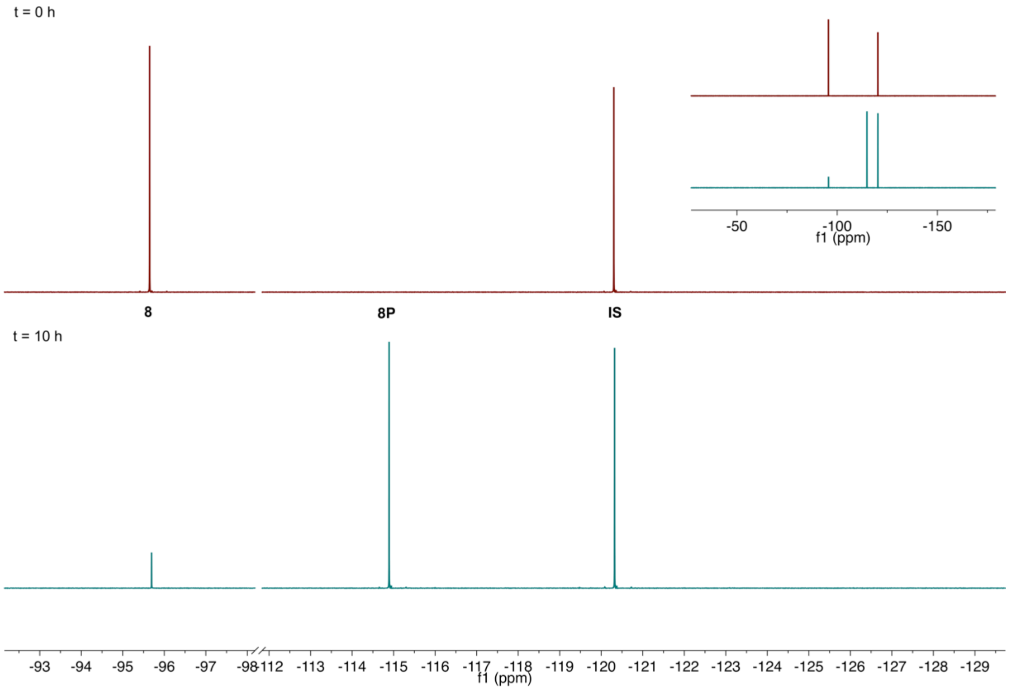

Figure S38. $^{19}F\{^{1}H\}$-NMR spectra monitoring the light-driven reaction progress of substrate **8** over time using a 623 nm high power LED irradiation. Reaction conditions: 20 mM substrate, 1 mol% $[Cu(dap)_2]Cl$, 10 mol% DCA, 0.5 eq Cs_2CO_3 and 20 eq. DiPEA in MeCN-d_3; IS = internal standard (4-fluorotoluene). The inset presents the same spectra (in the same colours) as the main part over a wider ppm range.

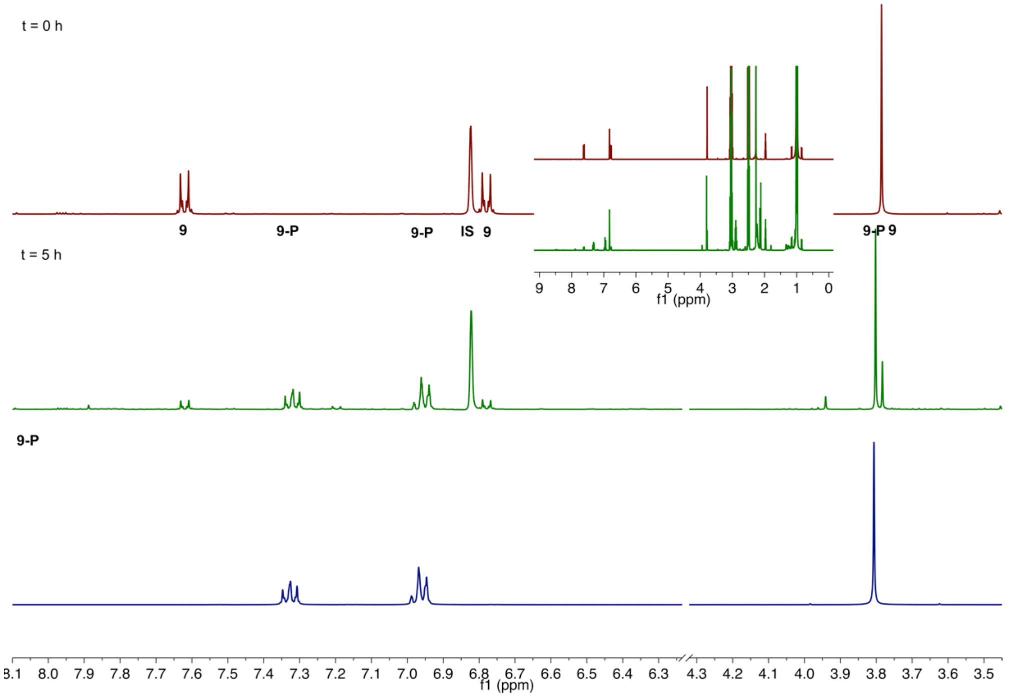

Figure S39. ^{1}H-NMR spectra monitoring the light-driven reaction progress of substrate **9** over time using a 623 nm high power LED irradiation. Reaction conditions: 20 mM substrate, 1 mol% $[Cu(dap)_2]Cl$, 10 mol% DCA, 0.5 eq Cs_2CO_3 and 20 eq. DiPEA in MeCN-d_3; IS = internal standard (mesitylene). The inset presents the same spectra (in the same colours) as the main part over a wider ppm range.

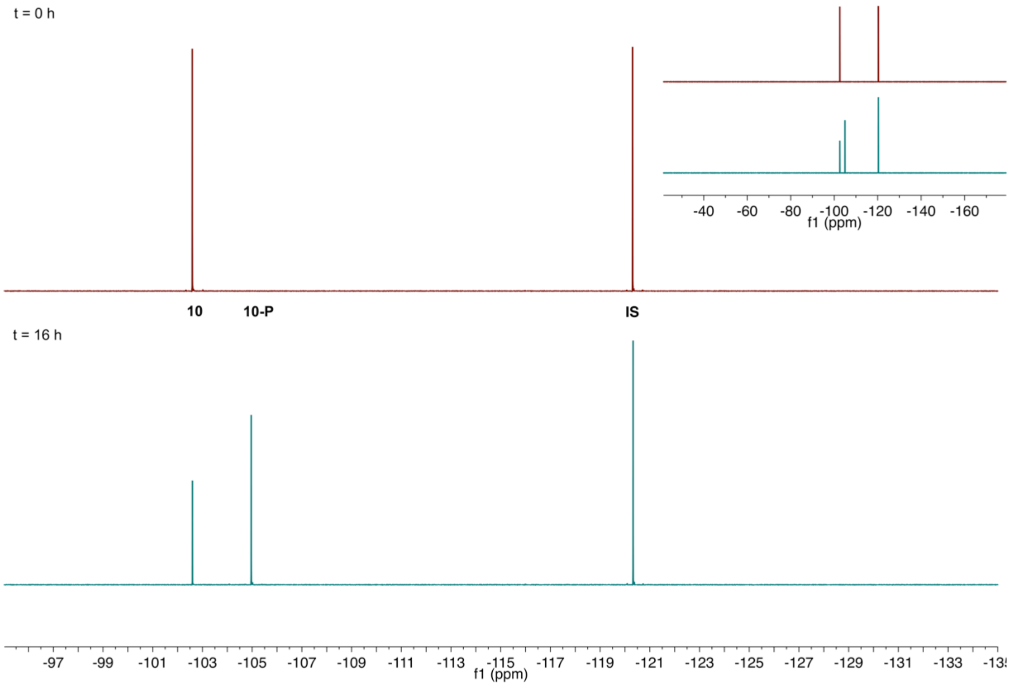

Figure S40. $^{19}F\{^{1}H\}$-NMR spectra monitoring the light-driven reaction progress of substrate **10** over time using a 623 nm high power LED irradiation. Reaction conditions: 25 mM substrate, 1 mol% $[Cu(dap)_2]Cl$, 10 mol% DCA, 0.5 eq Cs_2CO_3 and 20 eq. DiPEA in MeCN-d_3; IS = internal standard (4-fluorotoluene). The inset presents the same spectra (in the same colours) as the main part over a wider ppm range.

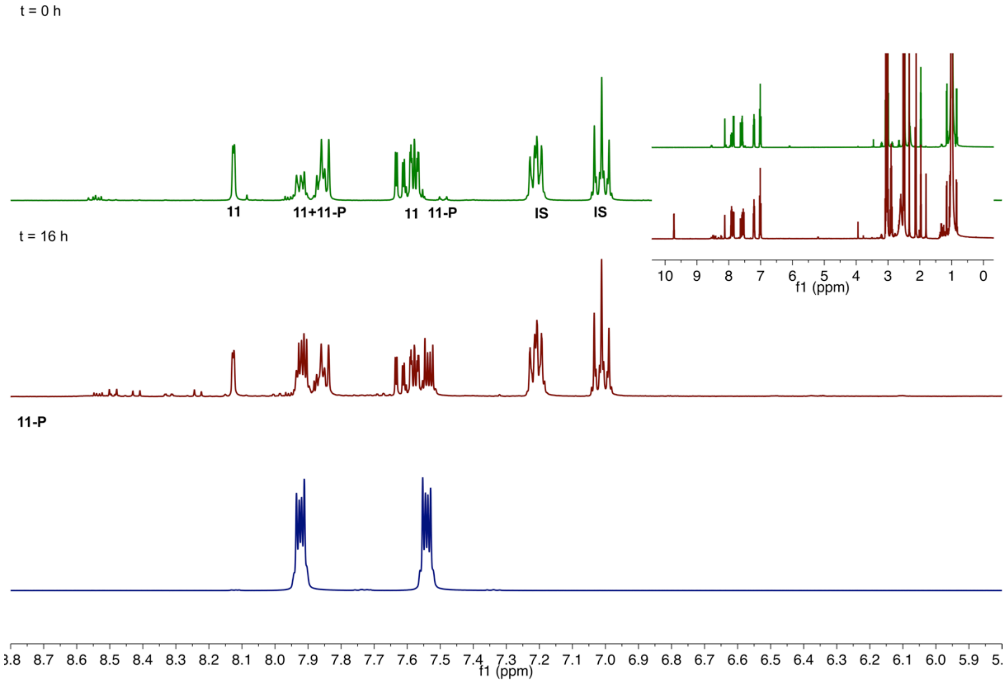

Figure S41. ^{1}H-NMR spectra monitoring the light-driven reaction progress of substrate **11** over time using a 623 nm high power LED irradiation. Reaction conditions: 25 mM substrate, 1 mol% $[Cu(dap)_2]Cl$, 10 mol% DCA, 0.5 eq Cs_2CO_3 and 20 eq. DiPEA in MeCN-d_3; IS = internal standard (4-fluorotoluene). The inset presents the same spectra (in the same colours) as the main part over a wider ppm range.

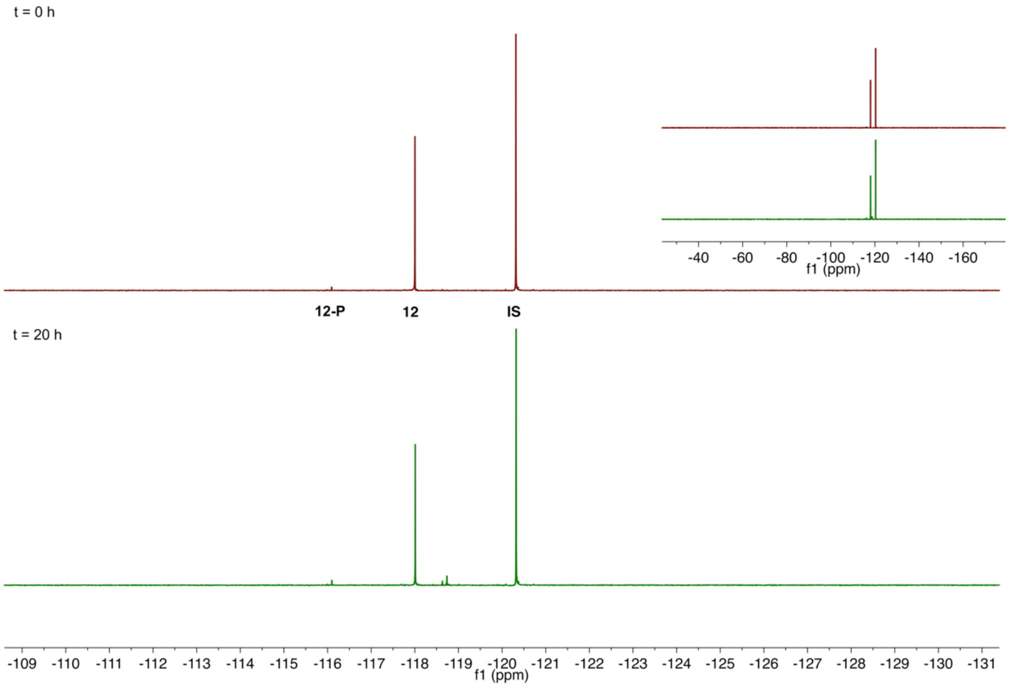

Figure S42. $^{19}F\{^{1}H\}$-NMR spectra monitoring the light-driven reaction progress of substrate **12** over time using a 623 nm high power LED irradiation. Reaction conditions: 25 mM substrate, 1 mol% $[Cu(dap)_2]Cl$, 10 mol% DCA, 0.5 eq Cs_2CO_3 and 20 eq. DiPEA in MeCN-d_3; IS = internal standard (4-fluorotoluene). The inset presents the same spectra (in the same colours) as the main part over a wider ppm range.

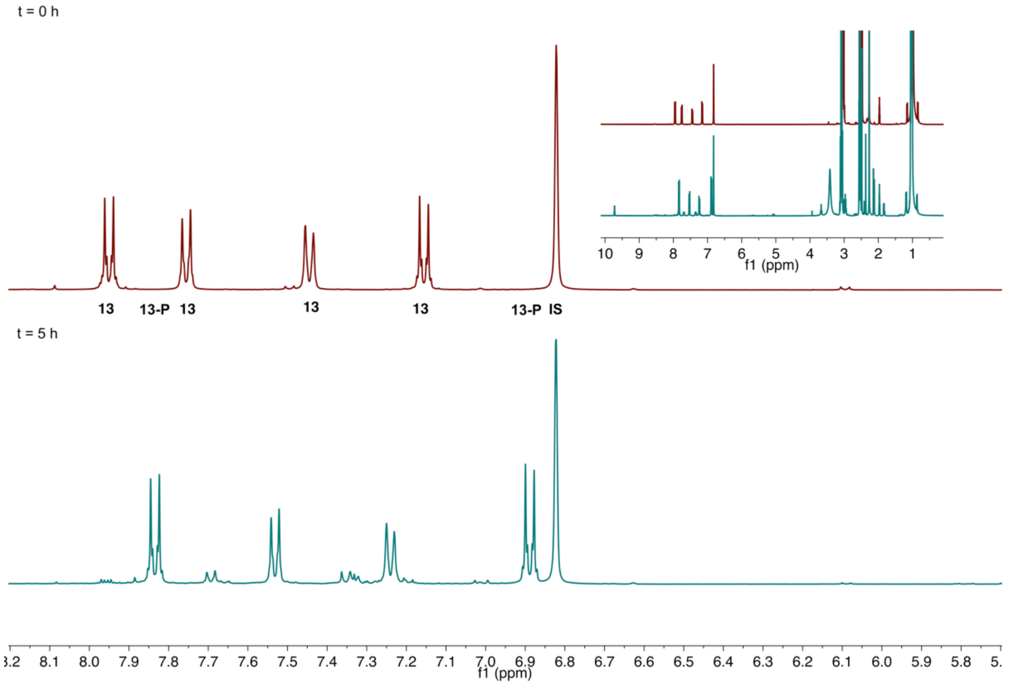

Figure S43. ^{1}H-NMR spectra monitoring the light-driven reaction progress of substrate **13** over time using a 623 nm high power LED irradiation. Reaction conditions: 25 mM substrate, 1 mol% $[Cu(dap)_2]Cl$, 10 mol% DCA and 20 eq. DiPEA in MeCN-d_3; IS = internal standard (mesitylene). The inset presents the same spectra (in the same colours) as the main part over a wider ppm range.

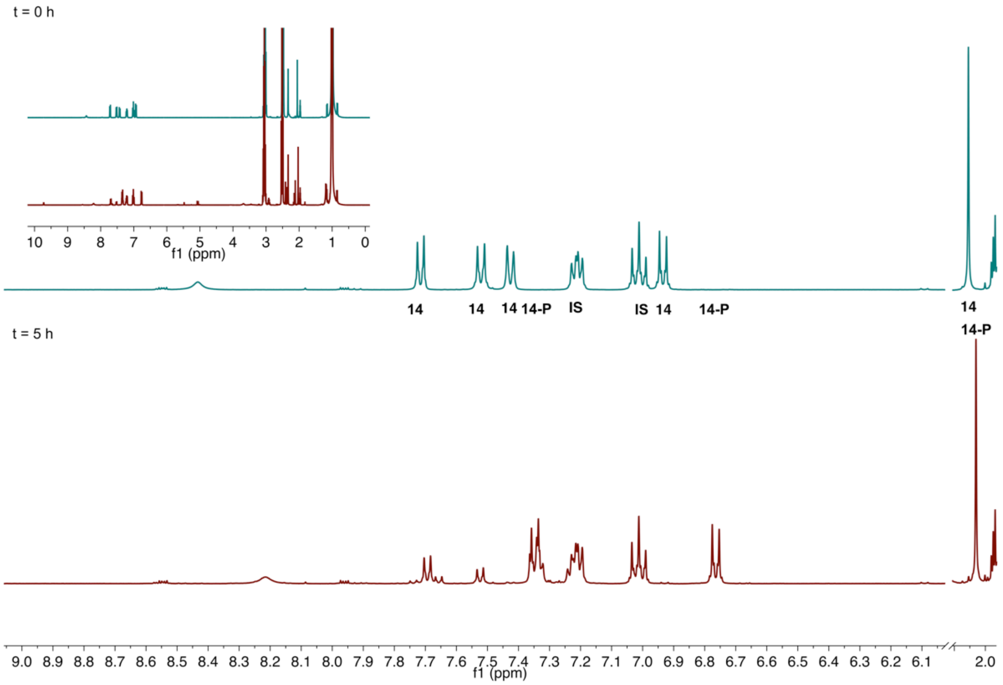

Figure S44. ^{1}H-NMR spectra monitoring the light-driven reaction progress of substrate **14** over time using a 623 nm high power LED irradiation. Reaction conditions: 25 mM substrate, 1 mol% [Cu(dap)$_2$]Cl, 10 mol% DCA and 20 eq. DiPEA in MeCN-d$_3$; IS = internal standard (4-fluorotoluene). The inset presents the same spectra (in the same colours) as the main part over a wider ppm range.

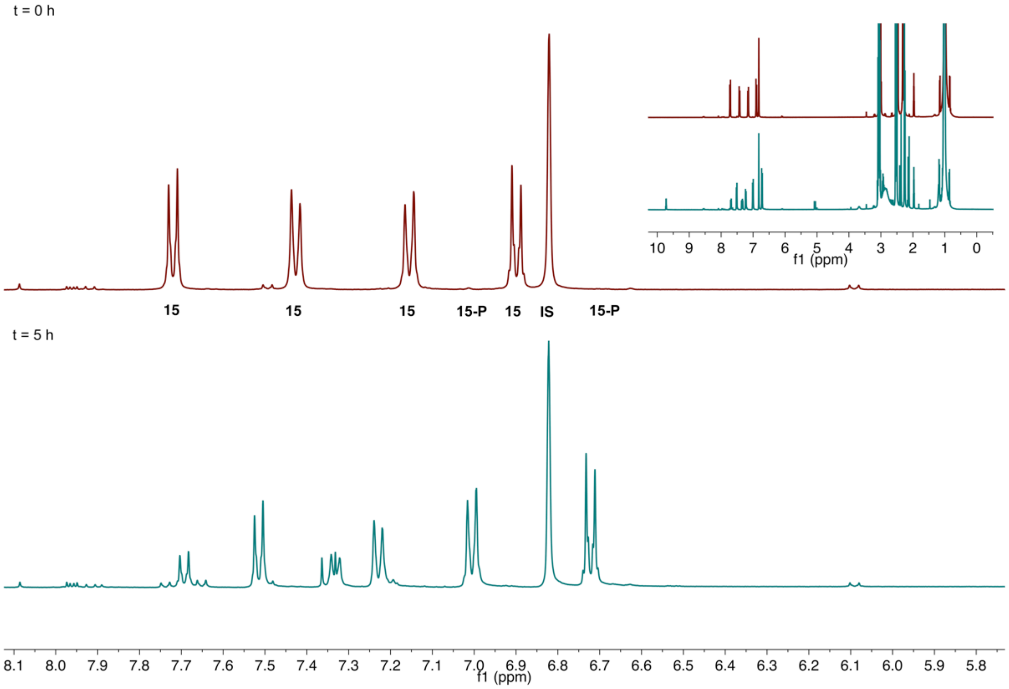

Figure S45. ^{1}H-NMR spectra monitoring the light-driven reaction progress of substrate **15** over time using a 623 nm high power LED irradiation. Reaction conditions: 25 mM substrate, 1 mol% [Cu(dap)$_2$]Cl, 10 mol% DCA and 20 eq. DiPEA in MeCN-d$_3$; IS = internal standard (mesitylene). The inset presents the same spectra (in the same colours) as the main part over a wider ppm range.

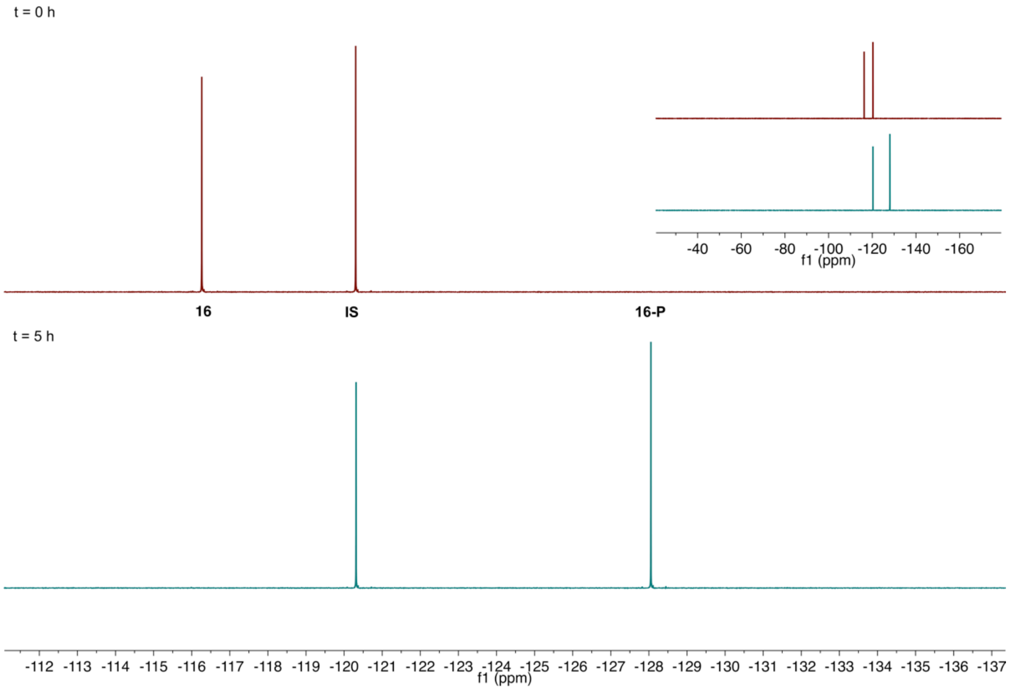

Figure S46. $^{19}F\{^1H\}$-NMR spectra monitoring the light-driven reaction progress of substrate **16** over time using a 623 nm high power LED irradiation. Reaction conditions: 25 mM substrate, 1 mol% [Cu(dap)$_2$]Cl, 10 mol% DCA and 20 eq. DiPEA in MeCN-d$_3$; IS = internal standard (4-fluorotoluene). The inset presents the same spectra (in the same colours) as the main part over a wider ppm range.

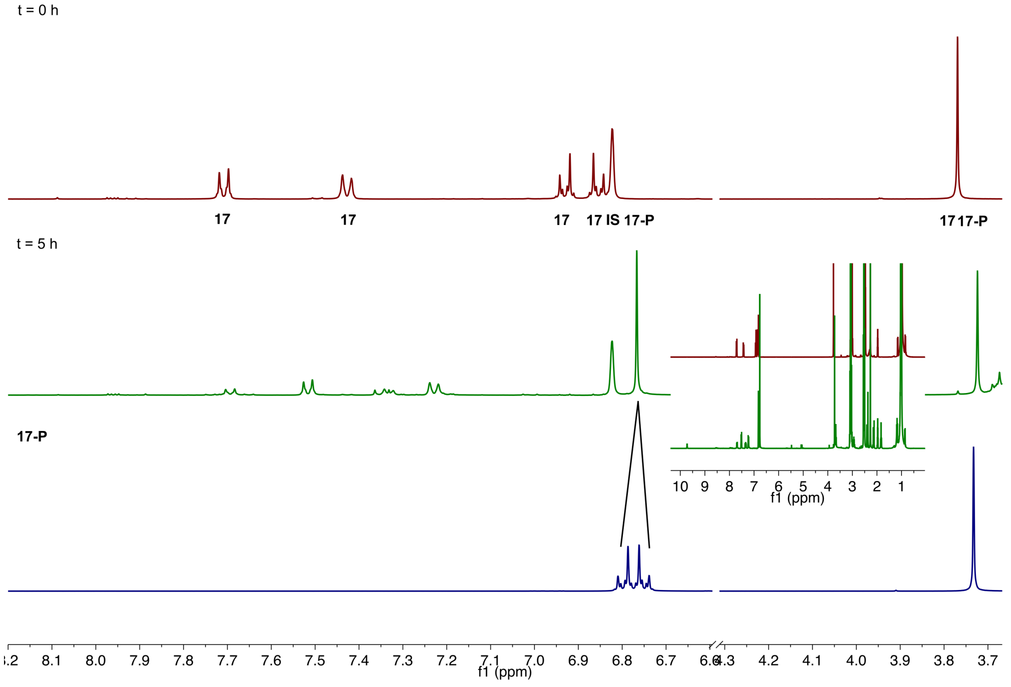

Figure S47. ^1H-NMR spectra monitoring the light-driven reaction progress of substrate **17** over time using a 623 nm high power LED irradiation. Reaction conditions: 25 mM substrate, 1 mol% [Cu(dap)$_2$]Cl, 10 mol% DCA and 20 eq. DiPEA in MeCN-d$_3$; IS = internal standard (mesitylene). The inset presents the same spectra (in the same colours) as the main part over a wider ppm range.

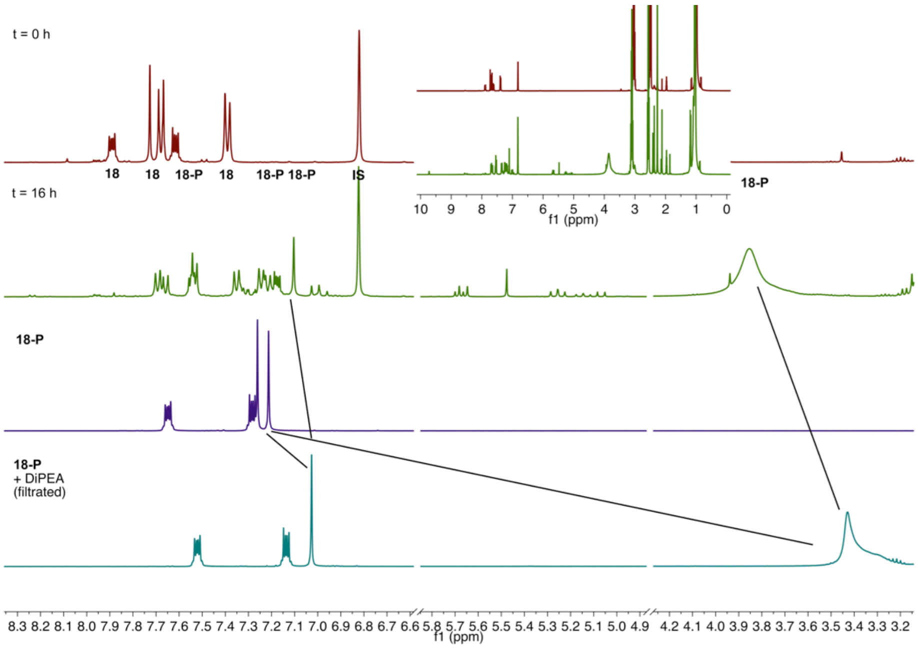

Figure S48. ^{1}H-NMR spectra monitoring the light-driven reaction progress of substrate **18** over time using a 623 nm high power LED irradiation. Reaction conditions: 25 mM substrate, 1 mol% [Cu(dap)$_2$]Cl, 10 mol% DCA and 20 eq. DiPEA in MeCN-d$_3$; IS = internal standard (mesitylene). ^{1}H-NMR shifts change upon deprotonation of 2,3-naphthalenediol (**18-P**) with excess DiPEA. Reference spectra of 2,3-naphthalenediol (**18-P**) in MeCN-d$_3$ and of 2,3-naphthalenediol (**18-P**) in MeCN-d$_3$ in the presence of excess DiPEA are included in the lower half of the figure (further information at the beginning of this section). The inset presents the same spectra (in the same colours) as the main part over a wider ppm range.

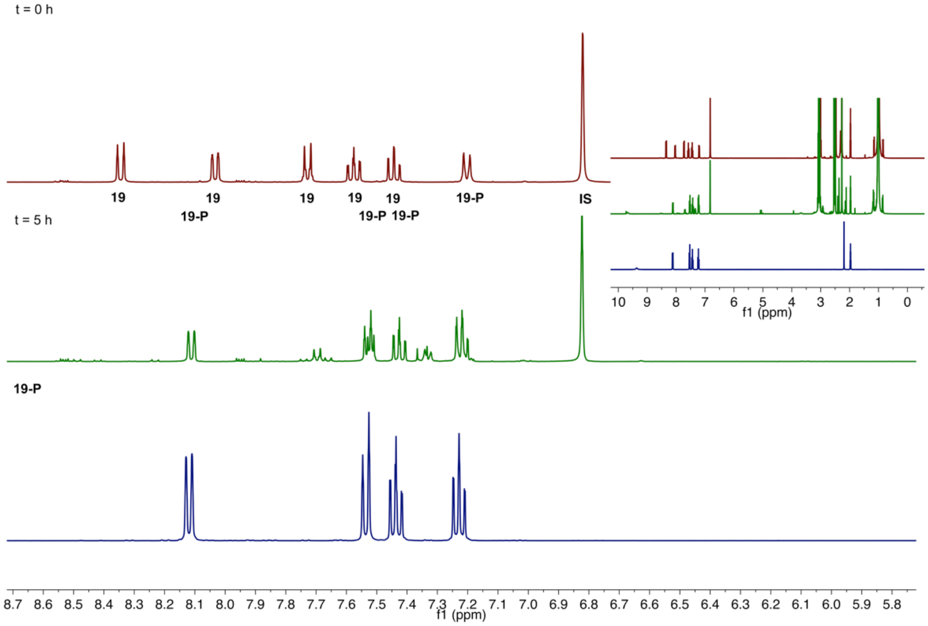

Figure S49. ^{1}H-NMR spectra monitoring the light-driven reaction progress of substrate **19** over time using a 623 nm high power LED irradiation. Reaction conditions: 25 mM substrate, 1 mol% [Cu(dap)$_2$]Cl, 10 mol% DCA and 20 eq. DiPEA in MeCN-d$_3$; IS = internal standard (mesitylene). The inset presents the same spectra (in the same colours) as the main part over a wider ppm range.

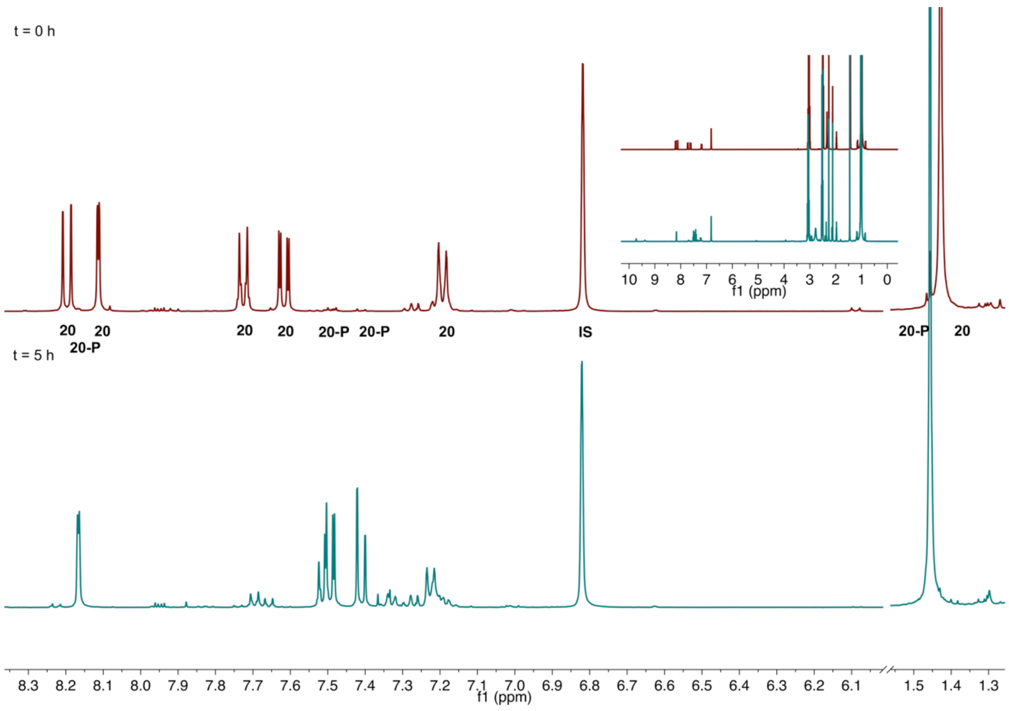

Figure S50. ^{1}H-NMR spectra monitoring the light-driven reaction progress of substrate **20** over time using a 623 nm high power LED irradiation. Reaction conditions: 25 mM substrate, 1 mol% [Cu(dap)$_2$]Cl, 10 mol% DCA and 20 eq. DiPEA in MeCN-d$_3$; IS = internal standard (mesitylene). The inset presents the same spectra (in the same colours) as the main part over a wider ppm range.

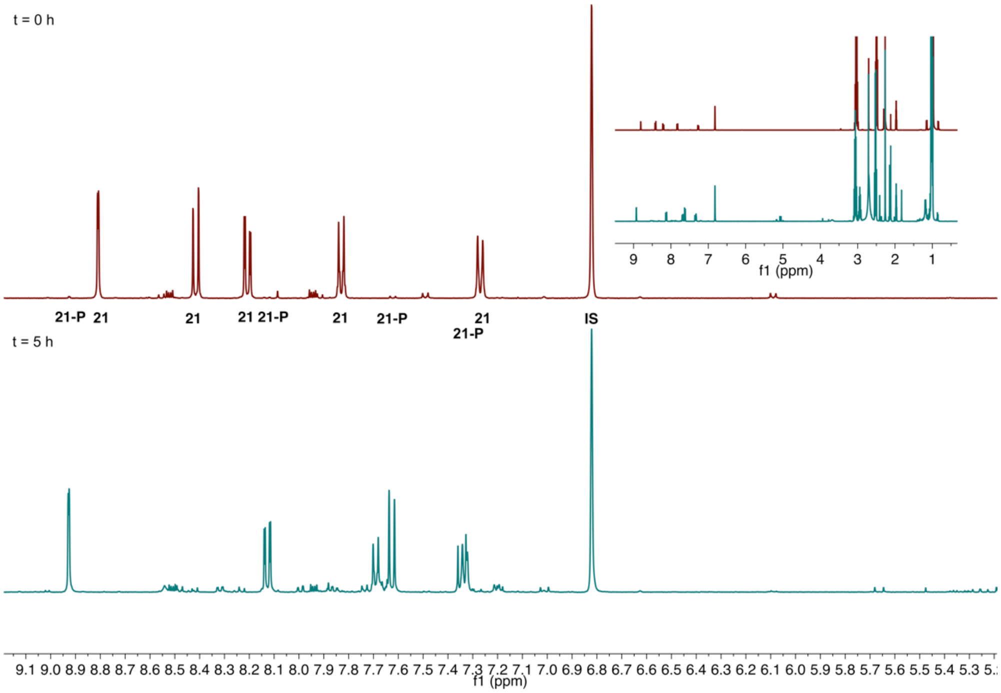

Figure S51. ^{1}H-NMR spectra monitoring the light-driven reaction progress of substrate **21** over time using a 623 nm high power LED irradiation. Reaction conditions: 25 mM substrate, 1 mol% [Cu(dap)$_2$]Cl, 10 mol% DCA and 20 eq. DiPEA in MeCN-d$_3$; IS = internal standard (mesitylene). The inset presents the same spectra (in the same colours) as the main part over a wider ppm range.

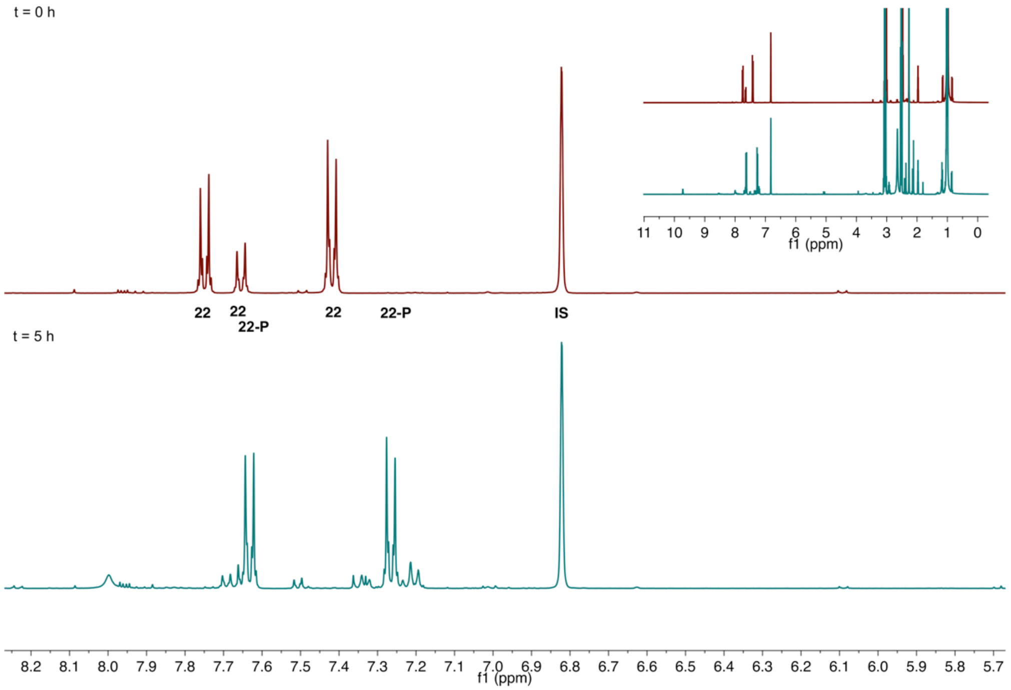

Figure S52. ^{1}H-NMR spectra monitoring the light-driven reaction progress of substrate **22** over time using a 623 nm high power LED irradiation. Reaction conditions: 25 mM substrate, 1 mol% $[Cu(dap)_2]Cl$, 10 mol% DCA and 20 eq. DiPEA in MeCN-d_3; IS = internal standard (mesitylene). The inset presents the same spectra (in the same colours) as the main part over a wider ppm range.

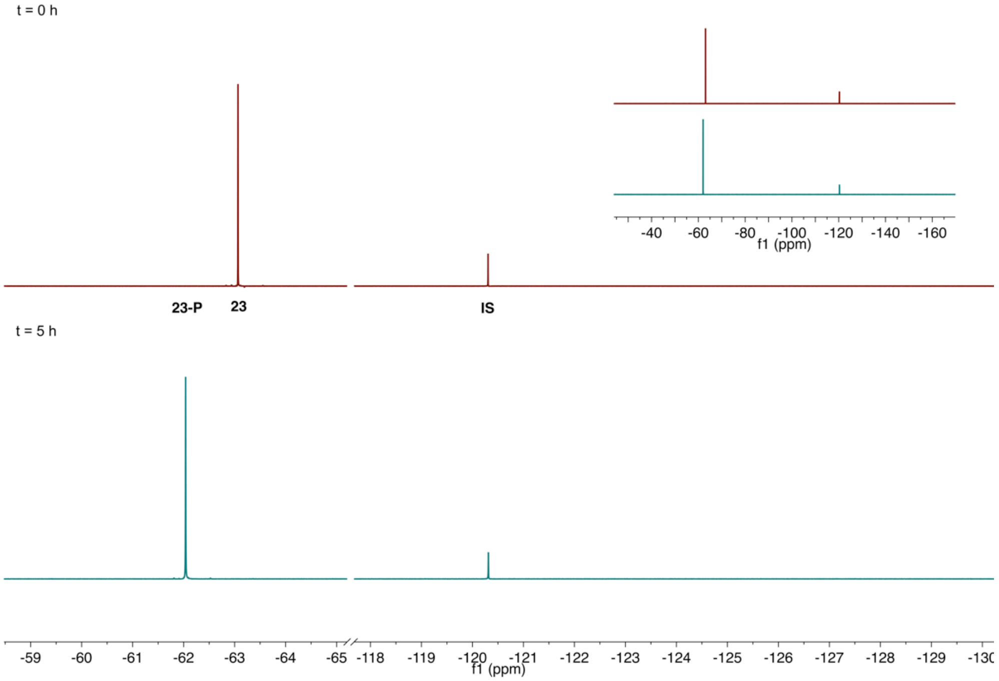

Figure S53. ^{19}F{^{1}H}-NMR spectra monitoring the light-driven reaction progress of substrate **23** over time using a 623 nm high power LED irradiation. Reaction conditions: 25 mM substrate, 1 mol% $[Cu(dap)_2]Cl$, 10 mol% DCA and 20 eq. DiPEA in MeCN-d_3; IS = internal standard (4-fluorotoluene). The inset presents the same spectra (in the same colours) as the main part over a wider ppm range.

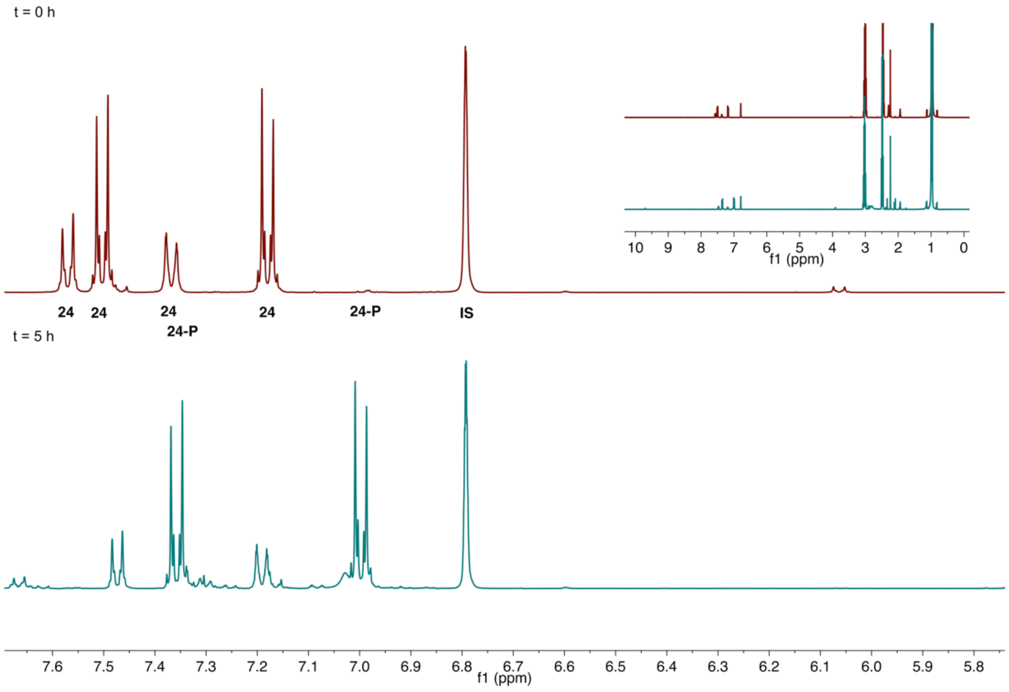

Figure S54. ^{1}H-NMR spectra monitoring consumption of substrate **24** over time using a 623 nm high power LED irradiation. Reaction conditions: 20 mM substrate, 1 mol% [Cu(dap)$_2$]Cl, 10 mol% DCA and 20 eq. DiPEA in MeCN-d$_3$; IS = internal standard (mesitylene). The inset presents the same spectra (in the same colours) as the main part over a wider ppm range.

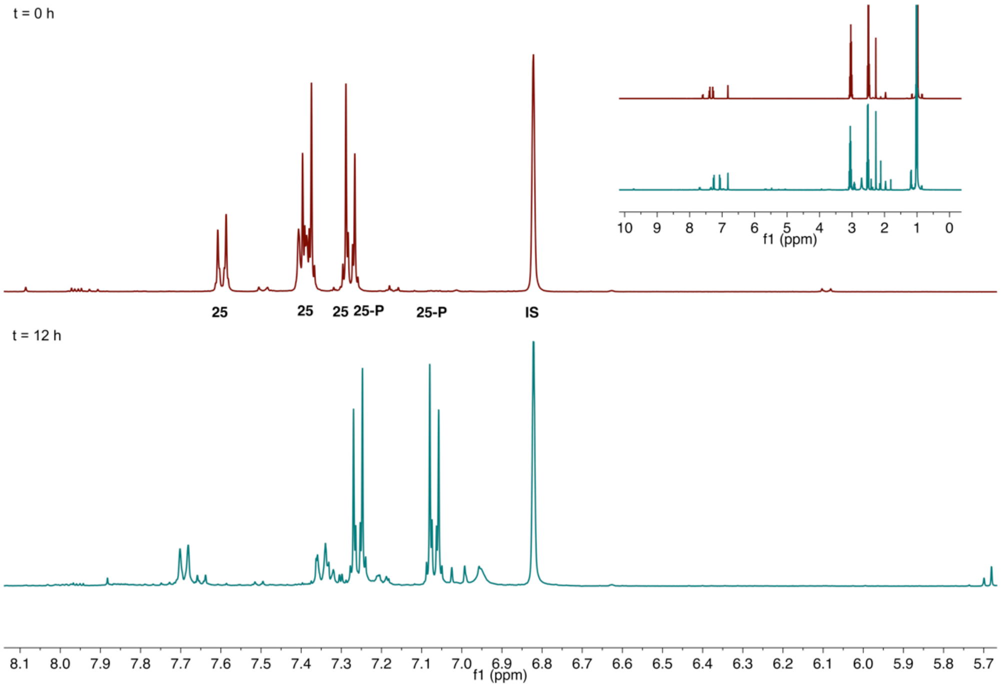

Figure S55. ^{1}H-NMR spectra monitoring the light-driven reaction progress of substrate **25** over time using a 623 nm high power LED irradiation. Reaction conditions: 25 mM substrate, 1 mol% [Cu(dap)$_2$]Cl, 10 mol% DCA and 20 eq. DiPEA in MeCN-d$_3$; IS = internal standard (mesitylene). The inset presents the same spectra (in the same colours) as the main part over a wider ppm range.

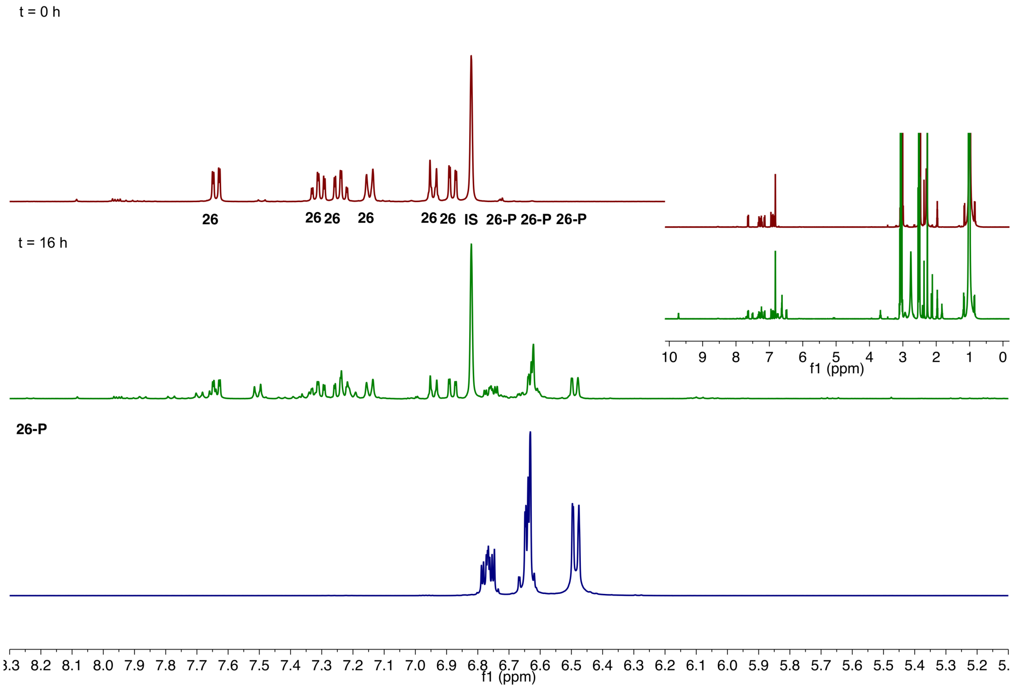

Figure S56. ^{1}H-NMR spectra monitoring the light-driven reaction progress of substrate **26** over time using a 623 nm high power LED irradiation. Reaction conditions: 25 mM substrate, 1 mol% [Cu(dap)$_2$]Cl, 10 mol% DCA and 20 eq. DiPEA in MeCN-d$_3$; IS = internal standard (mesitylene). The inset presents the same spectra (in the same colours) as the main part over a wider ppm range.

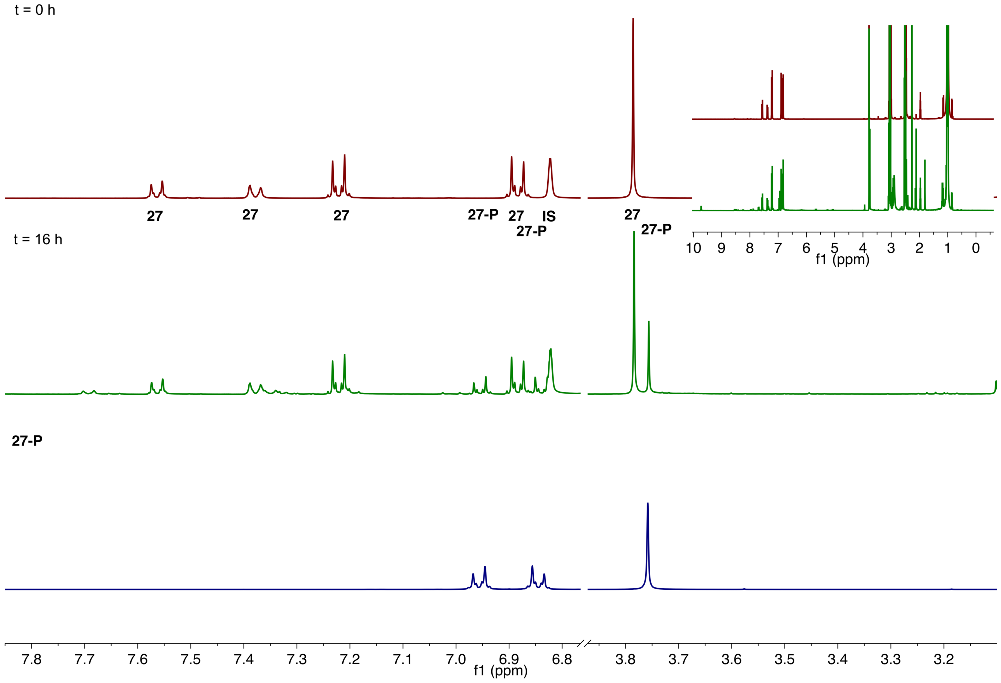

Figure S57. ^{1}H-NMR spectra monitoring the light-driven reaction progress of substrate **27** over time using a 623 nm high power LED irradiation. Reaction conditions: 25 mM substrate, 1 mol% [Cu(dap)$_2$]Cl, 10 mol% DCA and 20 eq. DiPEA in MeCN-d$_3$; IS = internal standard (mesitylene). The inset presents the same spectra (in the same colours) as the main part over a wider ppm range.

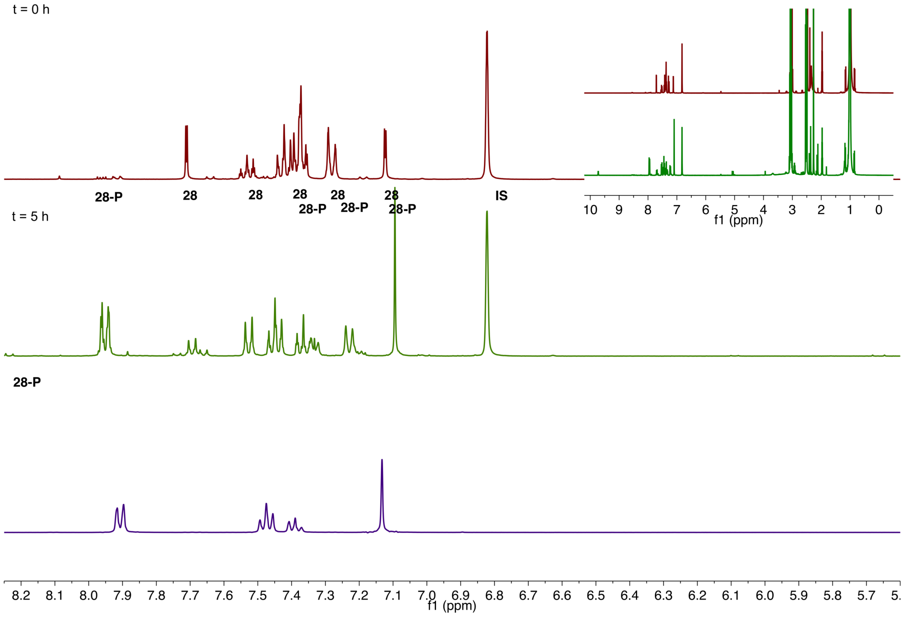

Figure S58. ^{1}H-NMR spectra monitoring the light-driven reaction progress of substrate **28** over time using a 623 nm high power LED irradiation. Reaction conditions: 25 mM substrate, 1 mol% [Cu(dap)$_2$]Cl, 10 mol% DCA and 20 eq. DiPEA in MeCN-d$_3$; IS = internal standard (mesitylene). The difference in chemical shift observed for the proton resonance around 8.0 ppm between the reaction after 5 h of irradiation and the reference compound (**28-P**) is likely caused by partial protonation of the product in the reaction mixture. The inset presents the same spectra (in the same colours) as the main part over a wider ppm range.

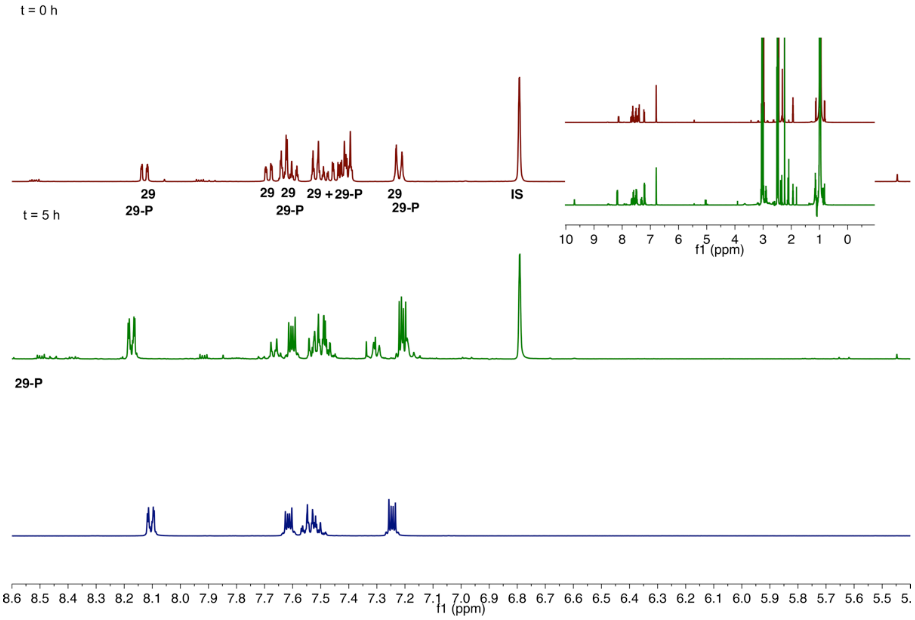

Figure S59. ^{1}H-NMR spectra monitoring the light-driven reaction progress of substrate **29** over time using a 623 nm high power LED irradiation. Reaction conditions: 25 mM substrate, 1 mol% [Cu(dap)$_2$]Cl, 10 mol% DCA and 20 eq. DiPEA in MeCN-d$_3$; IS = internal standard (mesitylene). The difference in chemical shift observed for the proton resonance around 8.1 ppm between the reaction after 5 h of irradiation and the reference compound (**29-P**) is likely caused by partial protonation of the product in the reaction mixture. The inset presents the same spectra (in the same colours) as the main part over a wider ppm range.

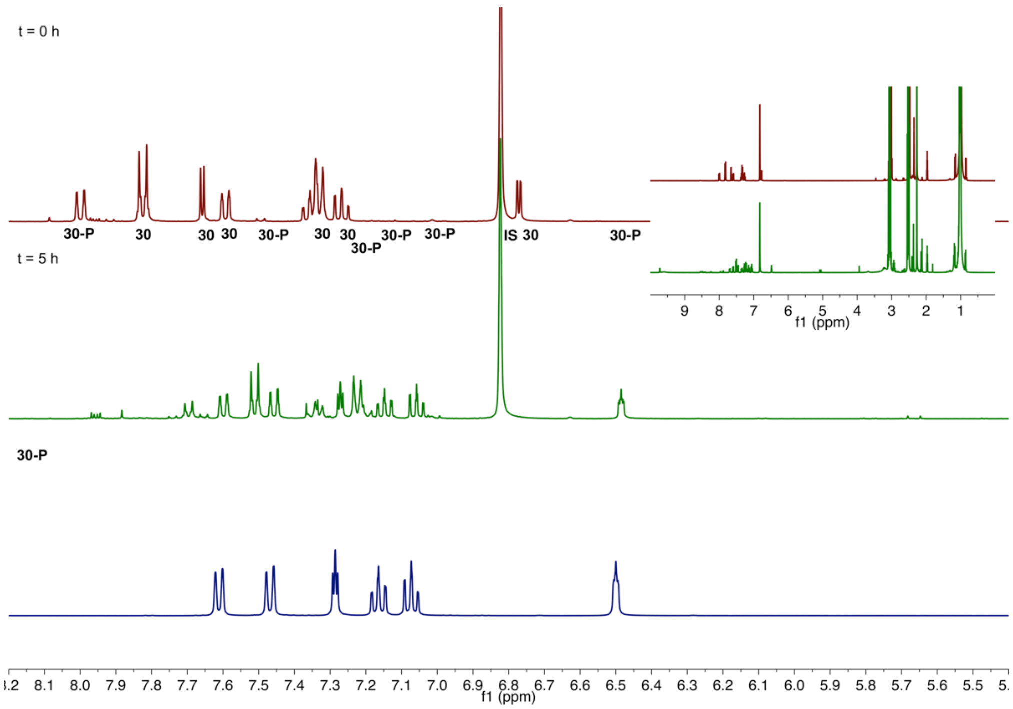

Figure S60. ^{1}H-NMR spectra monitoring the light-driven reaction progress of substrate **30** over time using a 623 nm high power LED irradiation (Thorlabs). Reaction conditions: 25 mM substrate, 1 mol% [Cu(dap)$_2$]Cl, 10 mol% DCA and 20 eq. DiPEA in MeCN-d$_3$; IS = internal standard (mesitylene). The inset presents the same spectra (in the same colours) as the main part over a wider ppm range.

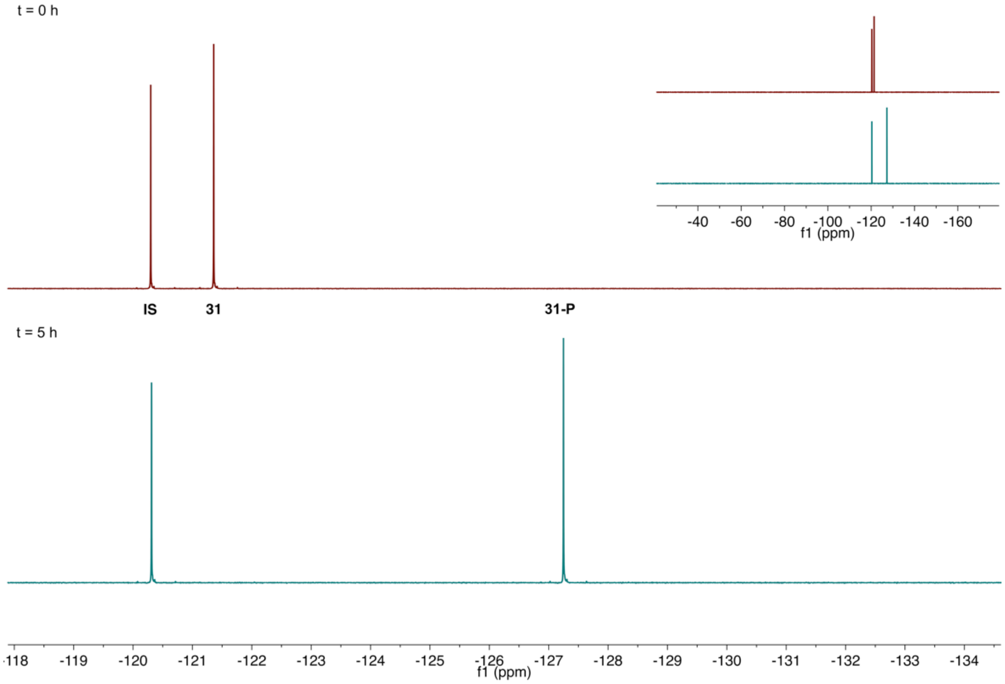

Figure S61. ^{19}F{^{1}H}-NMR spectra monitoring the light-driven reaction progress of substrate **31** over time using a 623 nm high power LED irradiation. Reaction conditions: 25 mM substrate, 1 mol% [Cu(dap)$_2$]Cl, 10 mol% DCA and 20 eq. DiPEA in MeCN-d$_3$; IS = internal standard (4-fluorotoluene). The inset presents the same spectra (in the same colours) as the main part over a wider ppm range.

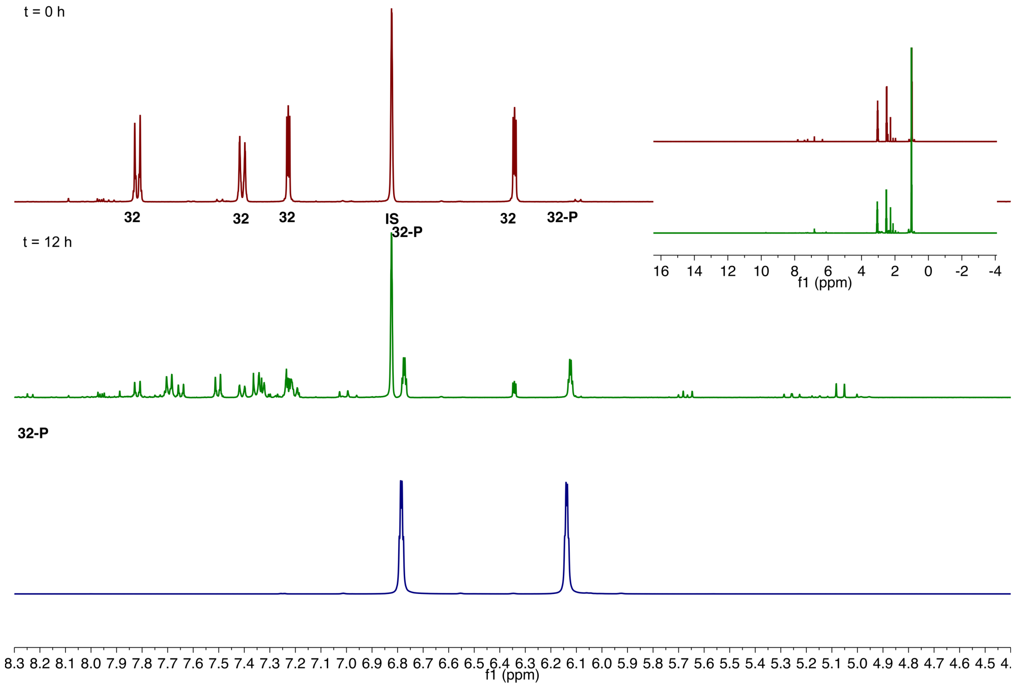

Figure S62. ^{1}H-NMR spectra monitoring the light-driven reaction progress of substrate **32** over time using a 623 nm high power LED irradiation. Reaction conditions: 25 mM substrate, 1 mol% [Cu(dap)$_2$]Cl, 10 mol% DCA and 20 eq. DiPEA in MeCN-d$_3$; IS = internal standard (mesitylene). The inset presents the same spectra (in the same colours) as the main part over a wider ppm range.

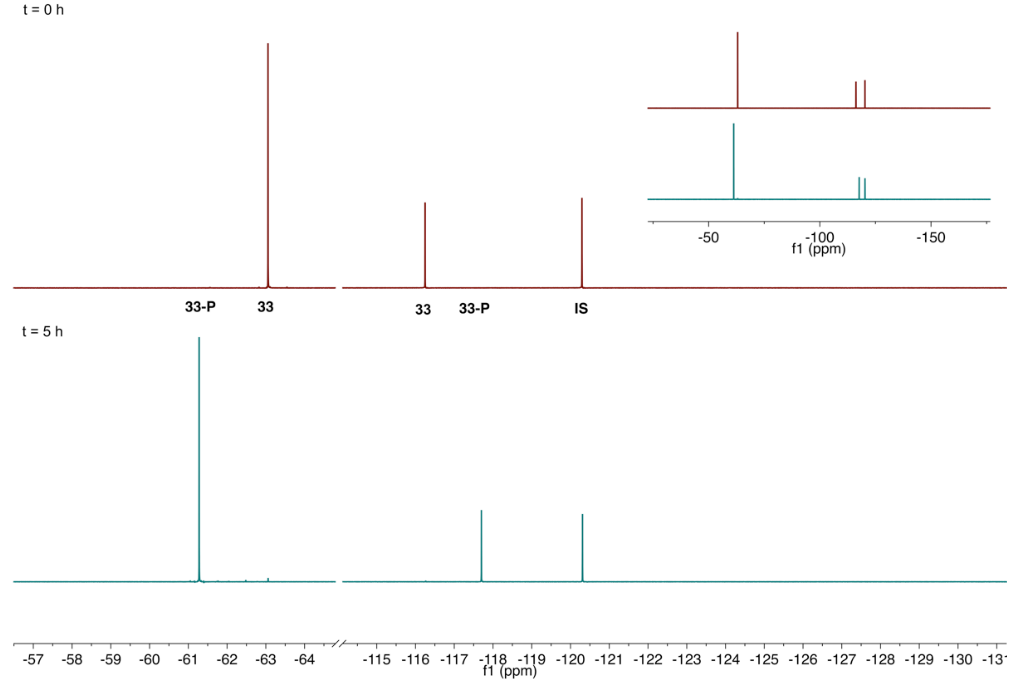

Figure S63. ^{19}F{^{1}H}-NMR spectra monitoring the light-driven reaction progress of substrate **33** over time using a 623 nm high power LED irradiation. Reaction conditions: 25 mM substrate, 1 mol% [Cu(dap)$_2$]Cl, 10 mol% DCA and 20 eq. DiPEA in MeCN-d$_3$; IS = internal standard (4-fluorotoluene). The inset presents the same spectra (in the same colours) as the main part over a wider ppm range.

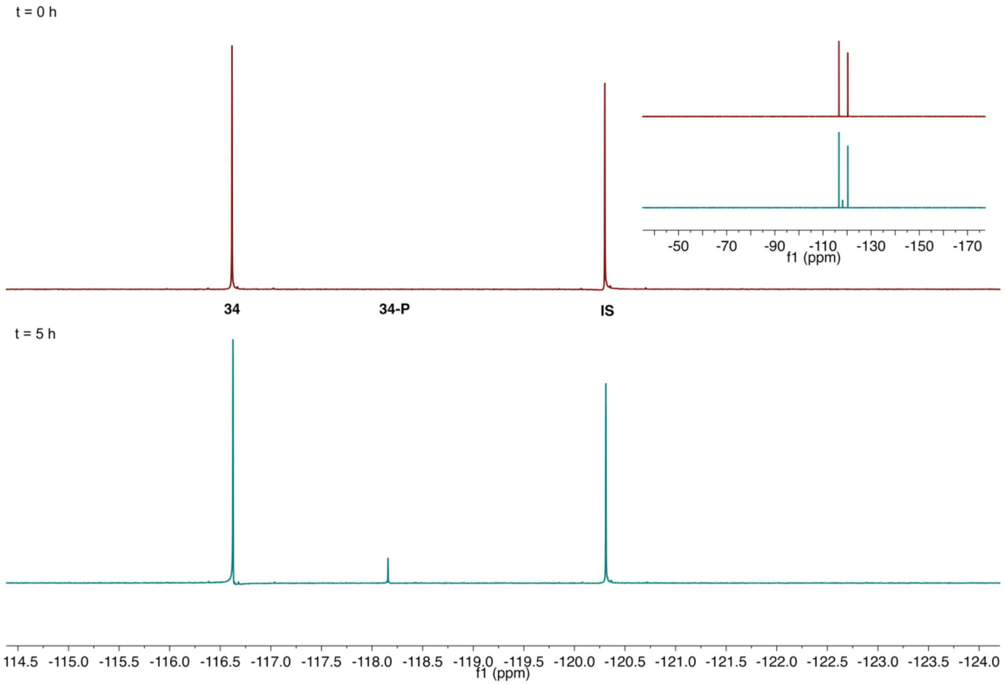

Figure S64. $^{19}F\{^{1}H\}$-NMR spectra monitoring the light-driven reaction progress of substrate **34** over time using a 623 nm high power LED irradiation. Reaction conditions: 25 mM substrate, 1 mol% $[Cu(dap)_2]Cl$, 10 mol% DCA and 20 eq. DiPEA in MeCN-d_3; IS = internal standard (4-fluorotoluene). The inset presents the same spectra (in the same colours) as the main part over a wider ppm range.

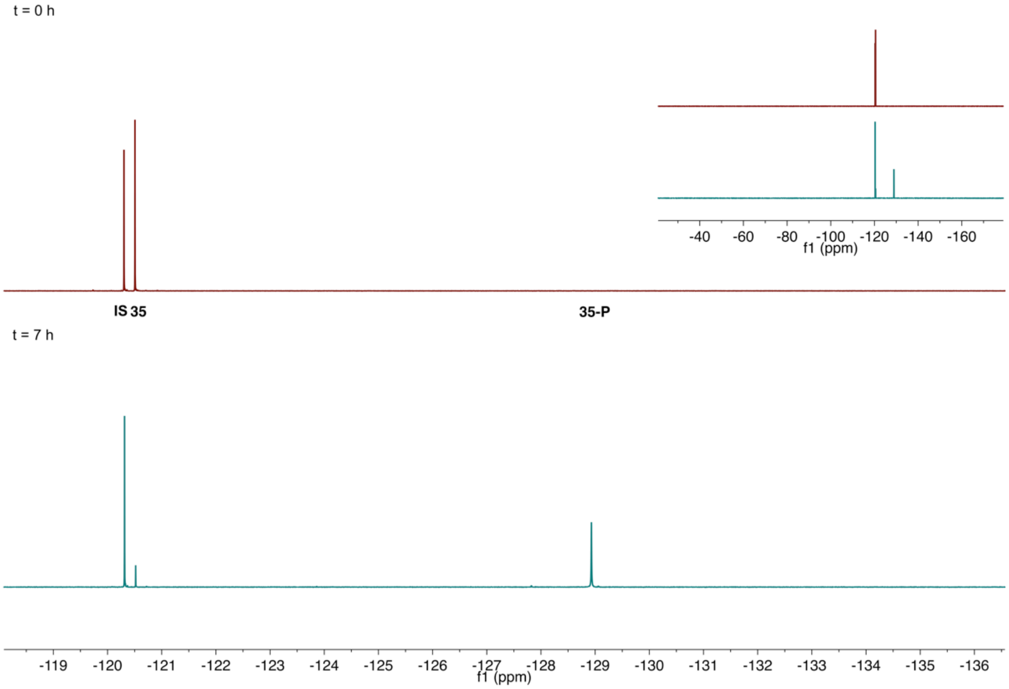

Figure S65. ^{19}F-NMR spectra monitoring the light-driven reaction progress of substrate **35** over time using a 623 nm high power LED irradiation. Reaction conditions: 25 mM substrate, 1 mol% $[Cu(dap)_2]Cl$, 10 mol% DCA and 20 eq. DiPEA in MeCN-d_3; IS = internal standard (4-fluorotoluene). The inset presents the same spectra (in the same colours) as the main part over a wider ppm range.

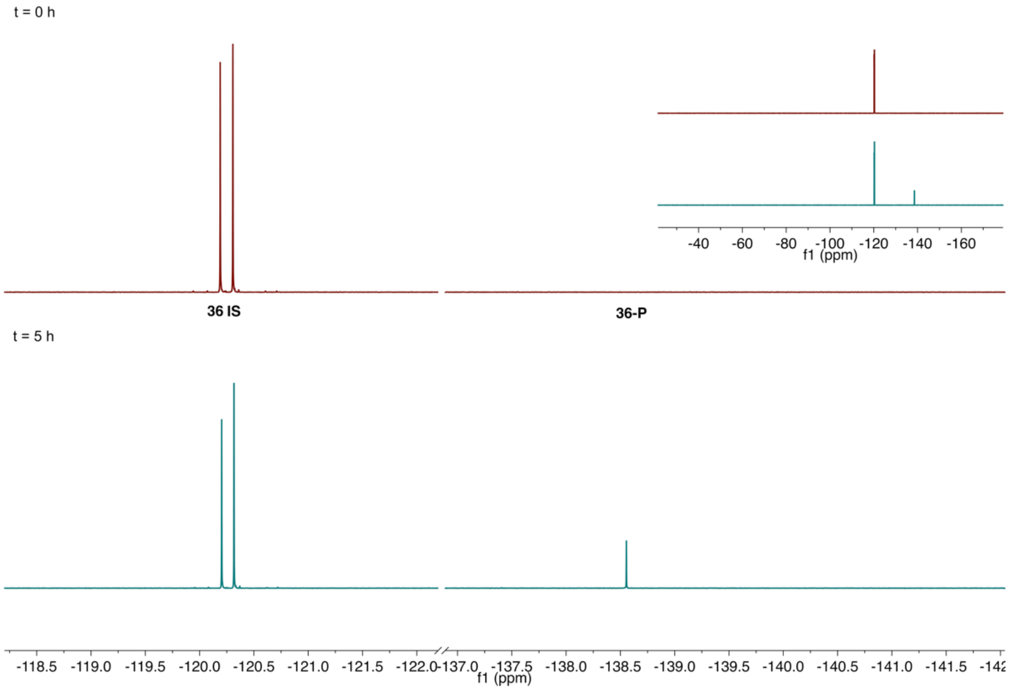

Figure S66. ^{19}F-NMR spectra monitoring the light-driven reaction progress of substrate **36** over time using a 623 nm high power LED irradiation. Reaction conditions: 25 mM substrate, 1 mol% [Cu(dap)$_2$]Cl, 10 mol% DCA and 20 eq. DiPEA in MeCN-d$_3$; IS = internal standard (4-fluorotoluene). The inset presents the same spectra (in the same colours) as the main part over a wider ppm range.

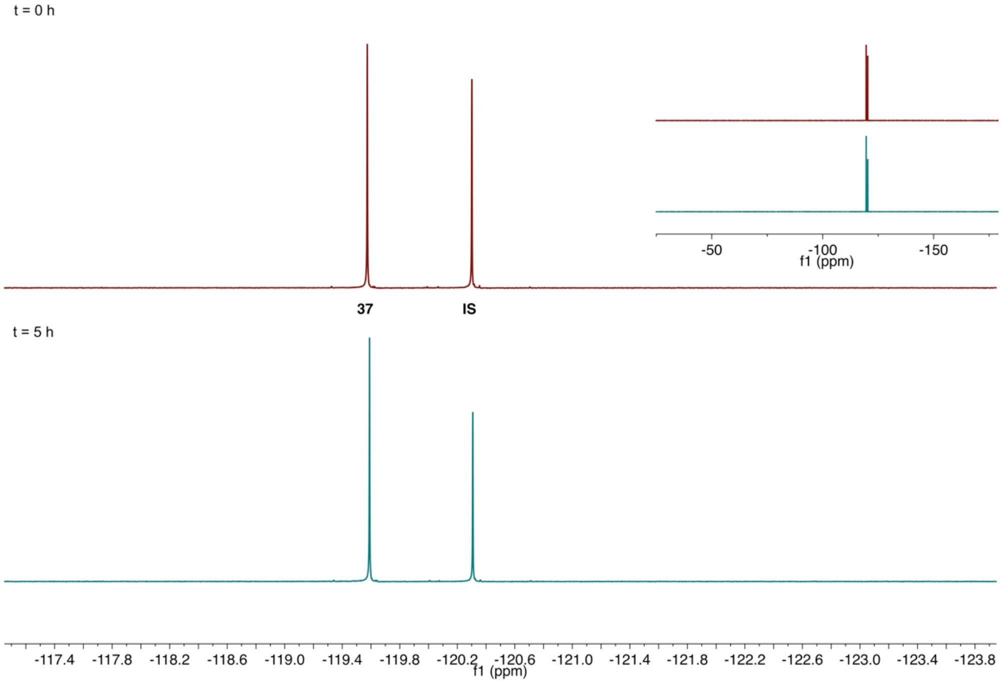

Figure S67. ^{19}F-NMR spectra monitoring the light-driven reaction progress of substrate **37** over time using a 623 nm high power LED irradiation. Reaction conditions: 25 mM substrate, 1 mol% [Cu(dap)$_2$]Cl, 10 mol% DCA and 20 eq. DiPEA in MeCN-d$_3$; IS = internal standard (4-fluorotoluene). The inset presents the same spectra (in the same colours) as the main part over a wider ppm range.

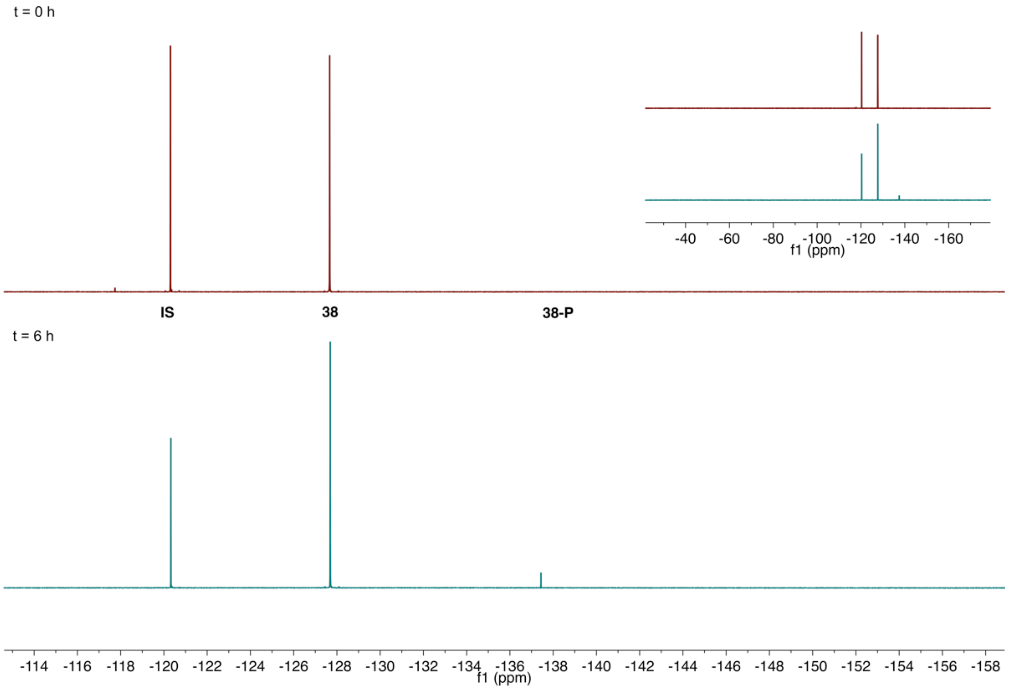

Figure S68. ^{19}F-NMR spectra monitoring the light-driven reaction progress of substrate **38** over time using a 623 nm high power LED irradiation (Thorlabs). Reaction conditions: 25 mM substrate, 1 mol% $[Cu(dap)_2]Cl$, 10 mol% DCA and 20 eq. DiPEA in MeCN-d_3; IS = internal standard (4-fluorotoluene). The inset presents the same spectra (in the same colours) as the main part over a wider ppm range.

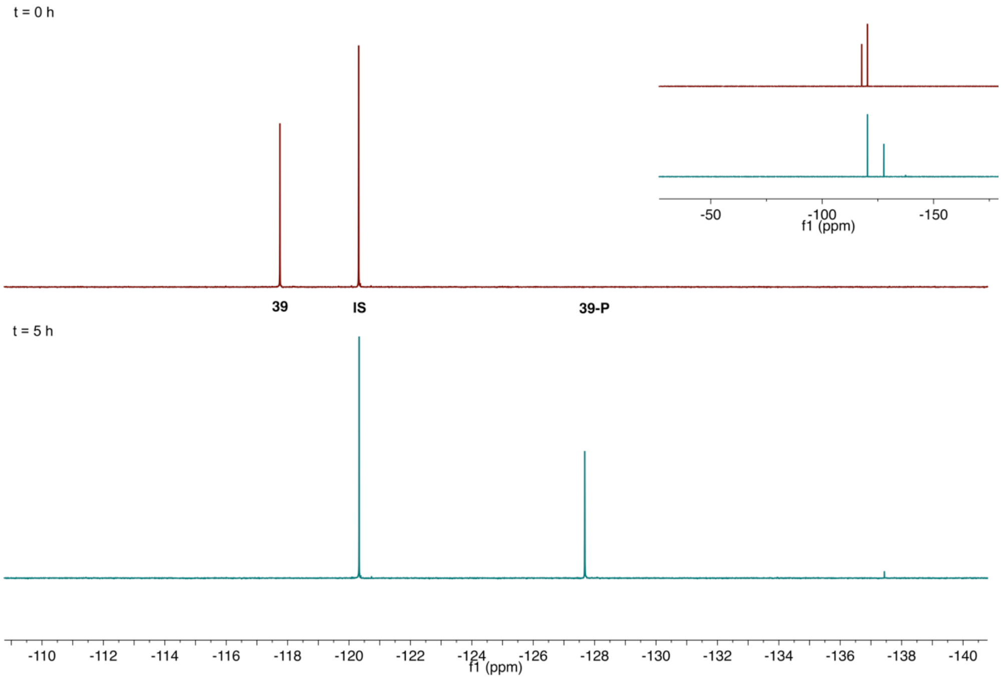

Figure S69. ^{19}F-NMR spectra monitoring the light-driven reaction progress of substrate **39** over time using a 623 nm high power LED irradiation. Reaction conditions: 25 mM substrate, 1 mol% $[Cu(dap)_2]Cl$, 10 mol% DCA and 20 eq. DiPEA in MeCN-d_3; IS = internal standard (4-fluorotoluene). The inset presents the same spectra (in the same colours) as the main part over a wider ppm range.

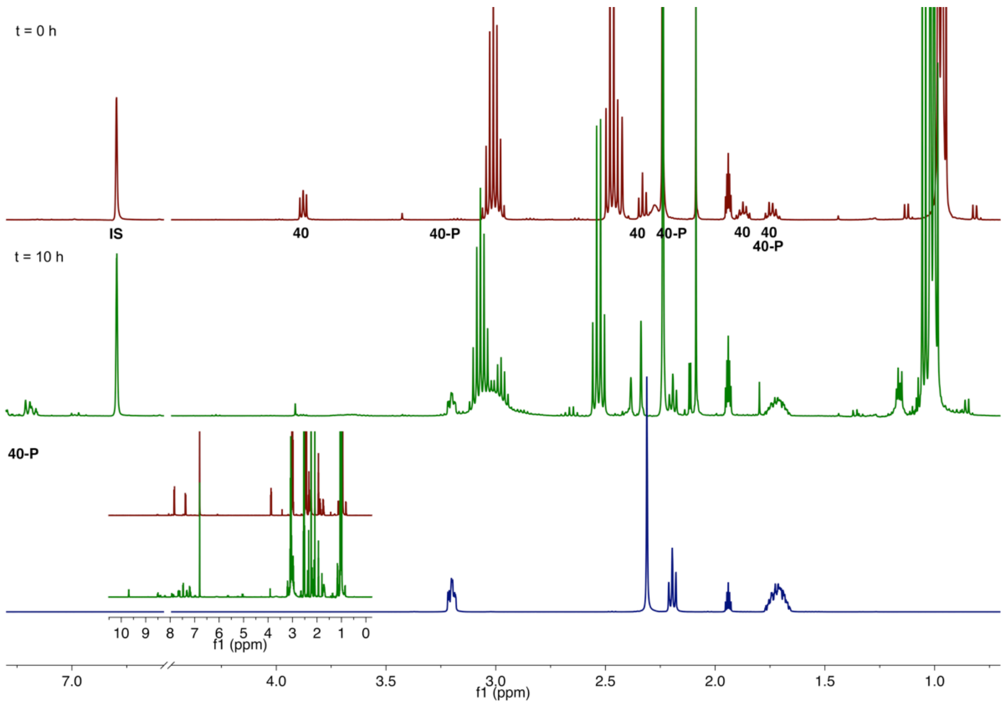

Figure S70. ^{1}H-NMR spectra monitoring the light-driven reaction progress of substrate **40** over time using a 623 nm high power LED irradiation. Reaction conditions: 25 mM substrate, 1 mol% [Cu(dap)$_2$]Cl, 10 mol% DCA and 20 eq. DiPEA in MeCN-d$_3$; IS = internal standard (mesitylene). The inset presents the same spectra (in the same colours) as the main part over a wider ppm range.

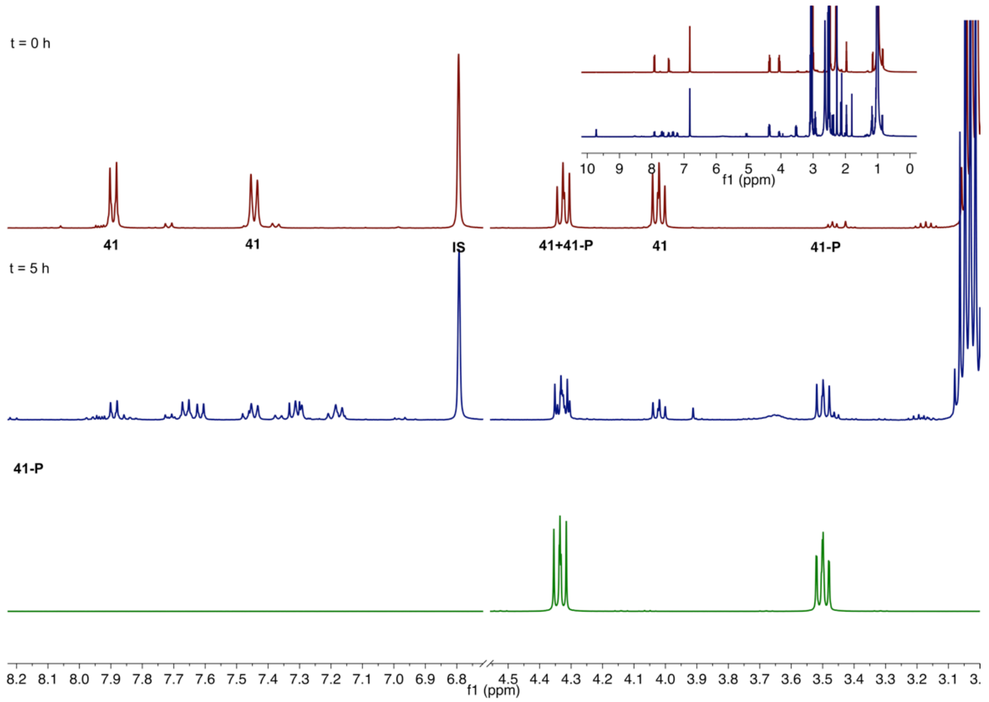

Figure S71. ^{1}H-NMR spectra monitoring the light-driven reaction progress of substrate **41** over time using a 623 nm high power LED irradiation. Reaction conditions: 25 mM substrate, 1 mol% [Cu(dap)$_2$]Cl, 10 mol% DCA and 20 eq. DiPEA in MeCN-d$_3$; IS = internal standard (mesitylene). The inset presents the same spectra (in the same colours) as the main part over a wider ppm range.

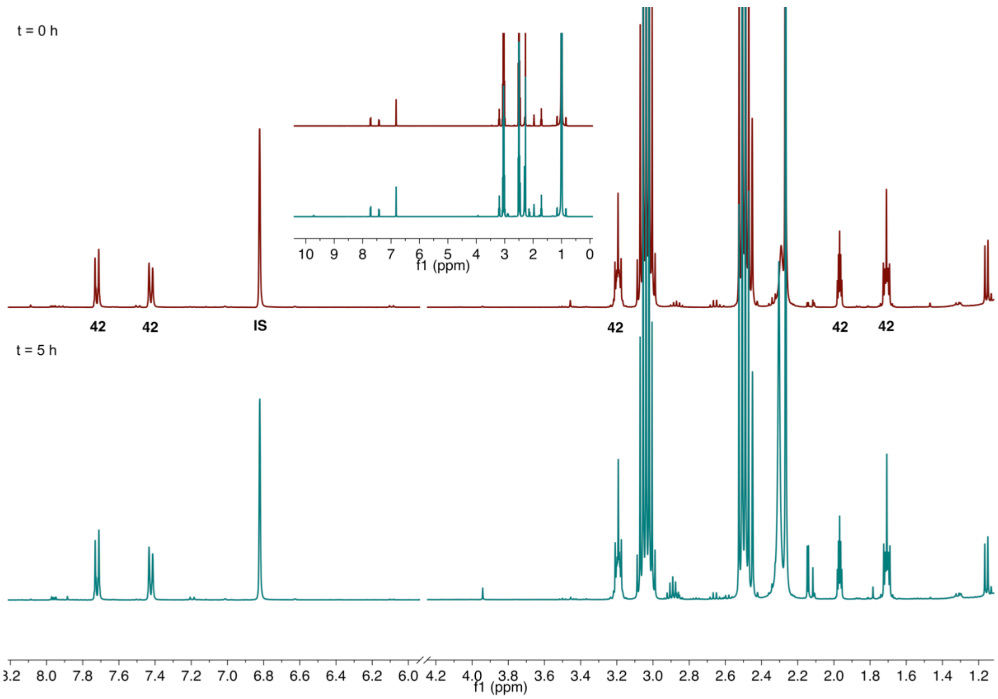

Figure S72. ^{1}H-NMR spectra monitoring the light-driven reaction progress of substrate **42** over time using a 623 nm high power LED irradiation. Reaction conditions: 25 mM substrate, 1 mol% [Cu(dap)$_2$]Cl, 10 mol% DCA and 20 eq. DiPEA in MeCN-d$_3$; IS = internal standard (mesitylene). The inset presents the same spectra (in the same colours) as the main part over a wider ppm range.

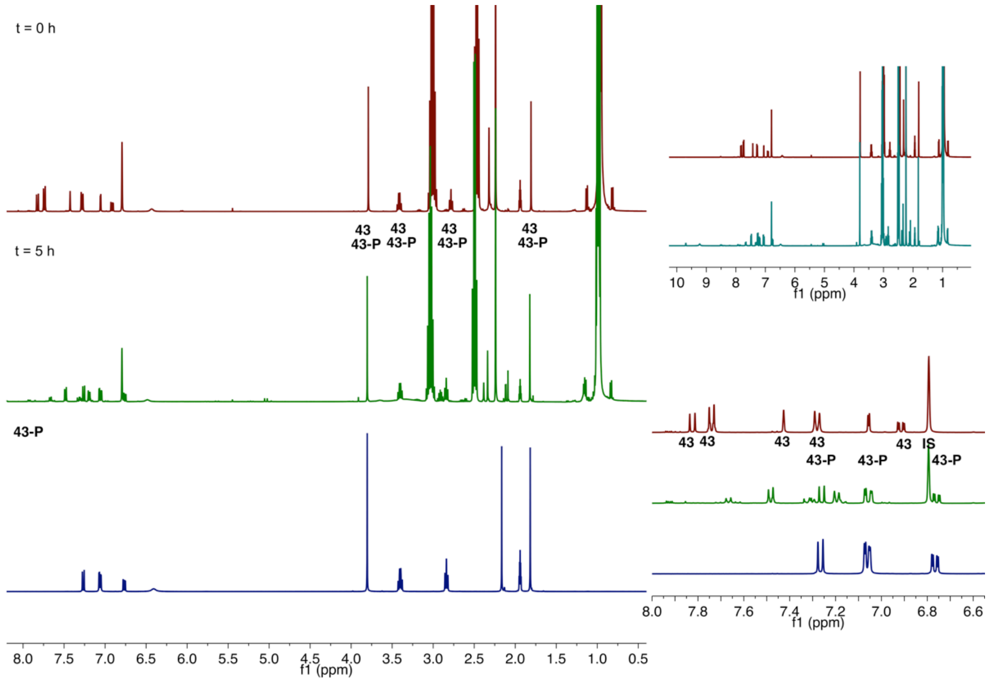

Figure S73. ^{1}H-NMR spectra monitoring the light-driven reaction progress of substrate **43** over time using a 623 nm high power LED irradiation. Reaction conditions: 25 mM substrate, 1 mol% [Cu(dap)$_2$]Cl, 10 mol% DCA and 20 eq. DiPEA in MeCN-d$_3$; IS = internal standard (mesitylene). The upper inset presents the same spectra (in identical color coding) as the main part without zoom of relevant spectral range. The bottom inset presents a zoom of the aromatic region of the ^{1}H-NMR spectra.

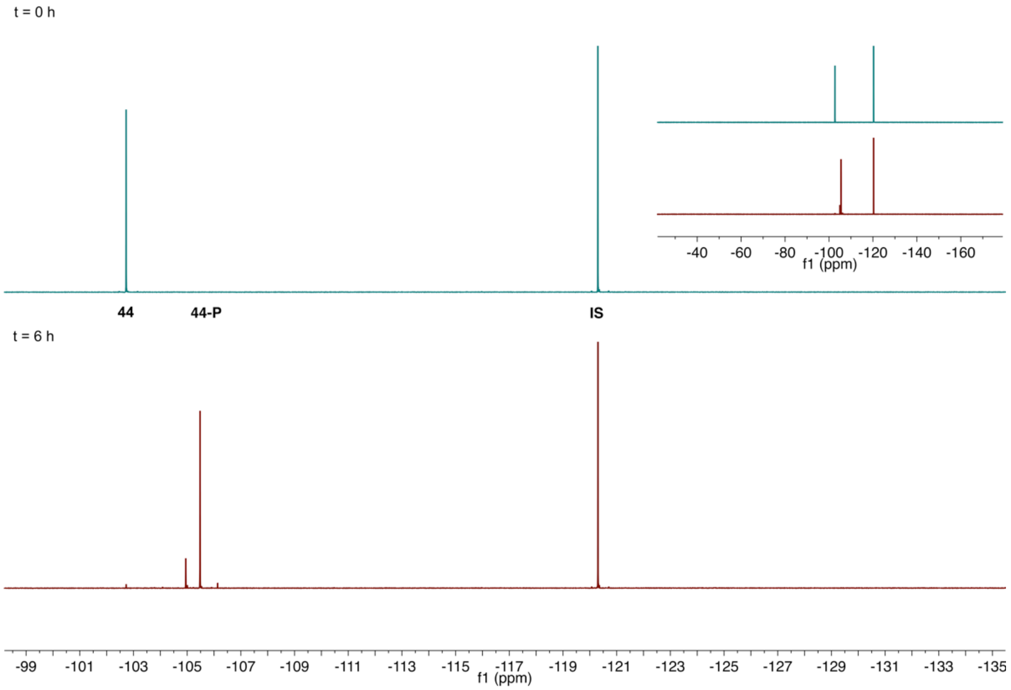

Figure S74. $^{19}F\{^{1}H\}$-NMR spectra monitoring the light-driven reaction progress of substrate **1** over time using a 623 nm high power LED irradiation. Reaction conditions: 25 mM substrate, 20 eq. *N*-methylpyrrole **44**, 1 mol% $[Cu(dap)_2]Cl$, 10 mol% DCA and 20 eq. DiPEA in MeCN-d_3; IS = internal standard (4-fluorotoluene). The inset presents the same spectra (in the same colours) as the main part over a wider ppm range.

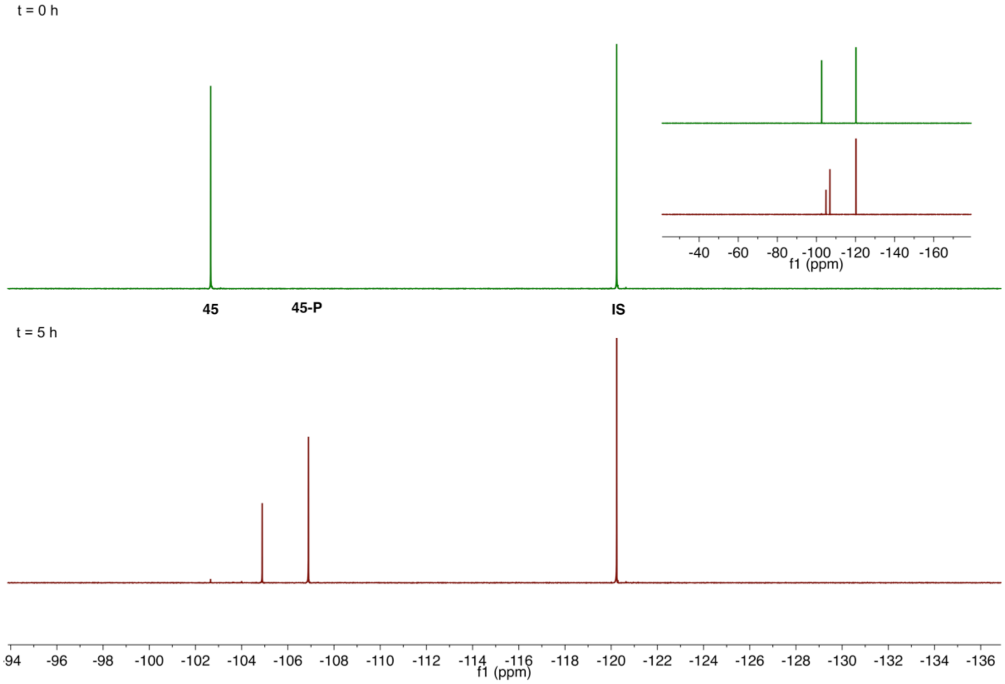

Figure S75. $^{19}F\{^{1}H\}$-NMR spectra monitoring the light-driven reaction progress of substrate **1** over time using a 623 nm high power LED irradiation. Reaction conditions: 25 mM substrate, 20 eq. 1,3,5-trimethoxybenzene **45**, 1 mol% $[Cu(dap)_2]Cl$, 10 mol% DCA, 0.5 eq. Cs_2CO_3 and 20 eq. DiPEA in MeCN-d_3; IS = internal standard (4-fluorotoluene). The inset presents the same spectra (in the same colours) as the main part over a wider ppm range.

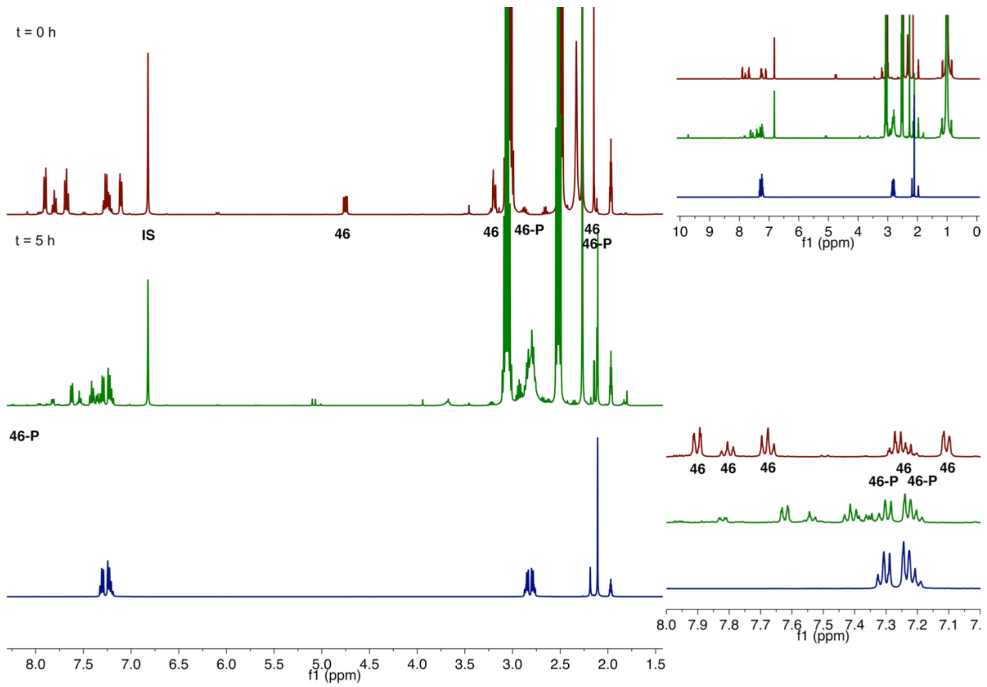

Figure S76. ^{1}H-NMR spectra monitoring the light-driven reaction progress of substrate **46** over time using a 623 nm high power LED irradiation. Reaction conditions: 25 mM substrate, 1 mol% [Cu(dap)$_2$]Cl, 10 mol% DCA and 20 eq. DiPEA in MeCN-d$_3$; IS = internal standard (mesitylene). The upper inset presents the same spectra (in identical color coding) as the main part without zoom of relevant spectral range. The bottom inset presents a zoom of the aromatic region of the ^{1}H-NMR spectra.

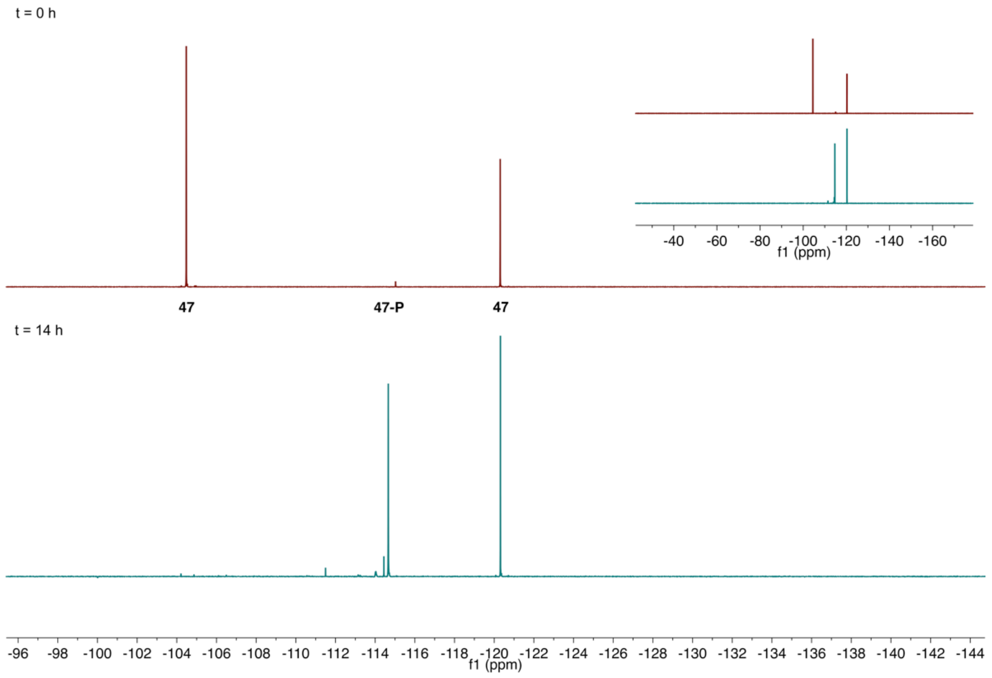

Figure S77. ^{19}F{^{1}H}-NMR spectra monitoring the light-driven reaction progress of substrate **47** over time using a 623 nm high power LED irradiation. Reaction conditions: 25 mM substrate, 1 mol% [Cu(dap)$_2$]Cl, 10 mol% DCA and 20 eq. DiPEA in MeCN-d$_3$; IS = internal standard (4-fluorotoluene). The inset presents the same spectra (in the same colours) as the main part over a wider ppm range.

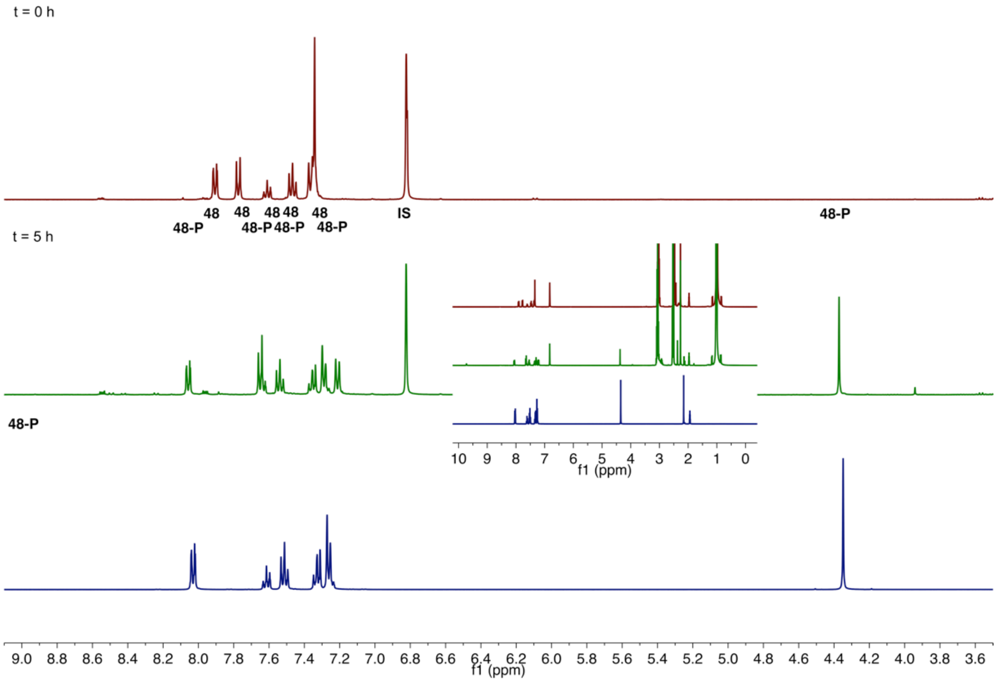

Figure S78. ^{1}H-NMR spectra monitoring the light-driven reaction progress of substrate **48** over time using a 623 nm high power LED irradiation. Reaction conditions: 25 mM substrate, 1 mol% [Cu(dap)$_2$]Cl, 10 mol% DCA and 20 eq. DiPEA in MeCN-d$_3$; IS = internal standard (mesitylene). The inset presents the same spectra (in the same colours) as the main part over a wider ppm range.

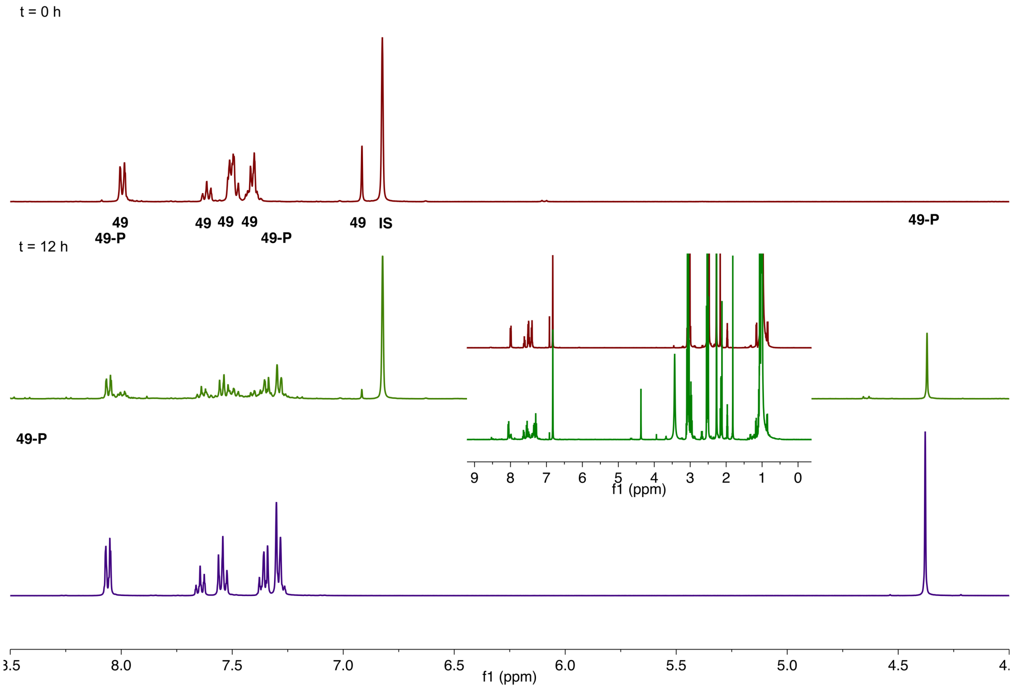

Figure S79. ^{1}H-NMR spectra monitoring the light-driven reaction progress of substrate **49** over time using a 623 nm high power LED irradiation. Reaction conditions: 25 mM substrate, 1 mol% [Cu(dap)$_2$]Cl, 10 mol% DCA and 20 eq. DiPEA in MeCN-d$_3$; IS = internal standard (mesitylene). The inset presents the same spectra (in the same colours) as the main part over a wider ppm range.

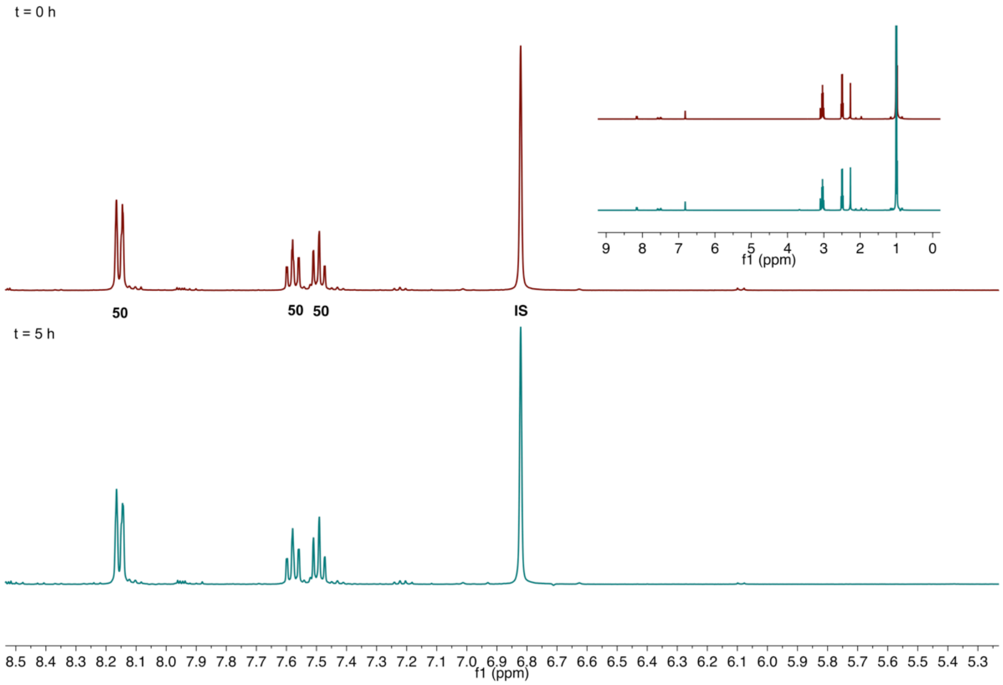

Figure S80. ^{1}H-NMR spectra monitoring the light-driven reaction progress of substrate **50** over time using a 623 nm high power LED irradiation. Reaction conditions: 25 mM substrate, 1 mol% [Cu(dap)$_2$]Cl, 10 mol% DCA and 20 eq. DiPEA in MeCN-d$_3$; IS = internal standard (mesitylene). The inset presents the same spectra (in the same colours) as the main part over a wider ppm range.

6 References

(1) Gottlieb, H. E.; Kotlyar, V.; Nudelman, A., NMR Chemical Shifts of Common Laboratory Solvents as Trace Impurities. *J. Org. Chem.* **1997**, *62*, 7512–7515.

(2) Garcia-Rodriguez, J.; Mendiratta, S.; White, M. A.; Xie, X.-S.; De Brabander, J. K., Synthesis and Structure–Activity Studies of the V-ATPase Inhibitor Saliphenylhalamide (SaliPhe) and Simplified Analogs. *Bioorg. Med. Chem. Lett.* **2015**, *25*, 4393–4398.

(3) Liu, X.; Liu, B.; Liu, Q., Migratory Hydrogenation of Terminal Alkynes by Base/Cobalt Relay Catalysis. *Angew. Chem. Int. Ed.* **2020**, *59*, 6750–6755.

(4) Duczynski, J.; Sobolev, A. N.; Moggach, S. A.; Dorta, R.; Stewart, S. G., The Synthesis and Catalytic Activity of New Mixed NHC-Phosphite Nickel(0) Complexes. *Organometallics* **2020**, *39*, 105–115.

(5) Song, W.; Dong, K.; Li, M., Visible Light-Induced Amide Bond Formation. *Org. Lett.* **2020**, *22*, 371–375.

(6) Bao, Z.-P.; Miao, R.-G.; Qi, X.; Wu, X.-F., A Novel Construction of Acetamides from Rhodium-Catalyzed Aminocarbonylation of DMC with Nitro Compounds. *Chem. Commun.* **2021**, *57*, 1955–1958.

(7) Gooßen, L. J.; Rodríguez, N.; Lange, P. P.; Linder, C., Decarboxylative Cross-Coupling of Aryl Tosylates with Aromatic Carboxylate Salts. *Angew. Chem.* **2010**, *122*, 1129–1132.

(8) Lei, X.; Jalla, A.; Abou Shama, M. A.; Stafford, J. M.; Cao, B., Chromatography-Free and Eco-Friendly Synthesis of Aryl Tosylates and Mesylates. *Synthesis* **2015**, *47*, 2578–2585.

(9) Wang, T.; Hoffmann, M.; Dreuw, A.; Hasagić, E.; Hu, C.; Stein, P. M.; Witzel, S.; Shi, H.; Yang, Y.; Rudolph, M., A Metal-free Direct Arene C–H Amination. *Adv. Synth. Catal.* **2021**, *363*, 2783–2795.

(10) Rowland, C. A.; Yap, G. P. A.; Bloch, E. D., Novel Syntheses of Carbazole-3,6-Dicarboxylate Ligands and Their Utilization for Porous Coordination Cages. *Dalt. Trans.* **2020**, *49*, 16340–16347.

(11) Wang, Y.; Fan, J.; Shi, J.; Qi, H.; Baranoff, E.; Xie, G.; Li, Q.; Tan, H.; Liu, Y.; Zhu, W., Influence of Integrated Alkyl-Chain Length on the Mesogenic and Photophysical Properties of Platinum-Based Metallomesogens and Their Application for Polarized White OLEDs. *Dye. Pigment.* **2016**, *133*, 238–247.

(12) Murase, T.; Fujita, M., Highly Blue Luminescent Triazine–Amine Conjugated Oligomers. *J. Org. Chem.* **2005**, *70*, 9269–9278.

(13) Escobar, R. A.; Johannes, J. W., A Unified and Practical Method for Carbon–Heteroatom Cross-Coupling Using Nickel/Photo Dual Catalysis. *Chem. Eur. J.* **2020**, *26*, 5168–5173.

(14) Yoo, W.; Tsukamoto, T.; Kobayashi, S., Visible-Light-Mediated Chan–Lam Coupling Reactions of Aryl Boronic Acids and Aniline Derivatives. *Angew. Chem. Int. Ed.* **2015**, *54*, 6587–6590.

(15) Hernandez-Olmos, V.; Abdelrahman, A.; El-Tayeb, A.; Freudendahl, D.; Weinhausen, S.; Müller, C. E., N-Substituted Phenoxazine and Acridone Derivatives: Structure–Activity Relationships of Potent P2X4 Receptor Antagonists. *J. Med. Chem.* **2012**, *55*, 9576–9588.

(16) Qiang-Liu; Liu, Y. X.; Song, H. J.; Wang, Q. M., Electron Transfer Photoredox Catalysis: Development

of a Photoactivated Reductive Desulfonylation of an Aza-Heteroaromatic Ring. *Adv. Synth. Catal.* **2020**, *362*, 3110–3115.

(17) Hu, Z.; Zhao, T.; Wang, M.; Wu, J.; Yu, W.; Chang, J., I_2-Mediated Intramolecular C–H Amidation for the Synthesis of *N*-Substituted Benzimidazoles. *J. Org. Chem.* **2017**, *82*, 3152–3158.

(18) Sailer, A.; Meiring, J. C. M.; Heise, C.; Pettersson, L. N.; Akhmanova, A.; Thorn-Seshold, J.; Thorn-Seshold, O., Pyrrole Hemithioindigo Antimitotics with Near-Quantitative Bidirectional Photoswitching That Photocontrol Cellular Microtubule Dynamics with Single-Cell Precision. *Angew. Chem. Int. Ed.* **2021**, *60*, 23695–23704.

(19) Gong, K.; Li, C.; Zhang, D.; Lu, H.; Wang, Y.; Li, H.; Zhang, H., Sulfonic Acid Functionalized Covalent Organic Frameworks as Efficient Catalyst for the One-Pot Tandem Reactions. *Mol. Catal.* **2022**, *519*, 112139.

(20) Ergen, S.; Nişancı, B.; Metin, Ö., One-Pot Reductive Amination of Aldehydes with Nitroarenes Using Formic Acid as the Hydrogen Donor and Mesoporous Graphitic Carbon Nitride Supported AgPd Alloy Nanoparticles as the Heterogeneous Catalyst. *New J. Chem.* **2018**, *42*, 10000–10006.

(21) Pecak, J.; Stöger, B.; Mastalir, M.; Veiros, L. F.; Ferreira, L. P.; Pignitter, M.; Linert, W.; Kirchner, K., Five-Coordinate Low-Spin $\{FeNO\}^7$ PNP Pincer Complexes. *Inorg. Chem.* **2019**, *58*, 4641–4646.

(22) Fu, Y.; Xu, Q. S.; Shi, C. Z.; Du, Z.; Xiao, C., Charge-Transfer Complex Promoted Regiospecific C–N Bond Cleavage of Vicinal Tertiary Diamines. *Adv. Synth. Catal.* **2018**, *360*, 3502–3506.

(23) Jiang, X.; Zhang, K.; Zhao, R.; Bai, D.; Wang, J.; Li, B.; Liu, Q.; Zhou, H., Metal-Free One-Pot Synthesis of N-Arylsulfonamides from Nitroarenes and Sodium Sulfinates in an Aqueous Medium. *Tetrahedron Lett.* **2020**, *61*, 152397.

(24) Schammel, A. W.; Boal, B. W.; Zu, L.; Mesganaw, T.; Garg, N. K., Exploration of the Interrupted Fischer Indolization Reaction. *Tetrahedron* **2010**, *66*, 4687–4695.

(25) Sirindil, F.; Miaskiewicz, S.; Kern, N.; Lalaut, A.; Felten, A.-S.; Weibel, J.-M.; Pale, P.; Blanc, A., Synthesis of 2-Carboxylated Aza-Ring Derivatives through α-Monohalogenation/Ring-Contraction of N-Sulfonyl Lactams. *Tetrahedron* **2017**, *73*, 5096–5106.

(26) Poeira, D. L.; Macara, J.; Faustino, H.; Coelho, J. A. S.; Gois, P. M. P.; Marques, M. M. B., Hypervalent Iodine Mediated Sulfonamide Synthesis. *Eur. J. Org. Chem.* **2019**, *2019*, 2695–2701.

(27) MacKenzie, I. A.; Wang, L.; Onuska, N. P. R.; Williams, O. F.; Begam, K.; Moran, A. M.; Dunietz, B. D.; Nicewicz, D. A., Discovery and Characterization of an Acridine Radical Photoreductant. *Nature* **2020**, *580*, 76–80.

(28) Xu, J.; Tong, R., An Environmentally Friendly Protocol for Oxidative Halocyclization of Tryptamine and Tryptophol Derivatives. *Green Chem.* **2017**, *19*, 2952–2956.

(29) Kern, C.; Selau, J.; Streuff, J., A Titanium-Catalyzed Reductive α-Desulfonylation. *Chem. Eur. J.* **2021**,

27, 6178–6182.

(30) Liu, Y.; Liu, R.; Szostak, M., $Sc(OTf)_3$-Catalyzed Synthesis of Anhydrides from Twisted Amides. *Org. Biomol. Chem.* **2017**, *15*, 1780–1785.

(31) Gonda, Z.; Novák, Z., Transition-Metal-Free *N*-Arylation of Pyrazoles with Diaryliodonium Salts. *Chem. Eur. J.* **2015**, *21*, 16801–16806.

(32) Boelke, A.; Nachtsheim, B. J., Evolution of *N*‐Heterocycle‐Substituted Iodoarenes (NHIAs) to Efficient Organocatalysts in Iodine(I/III)‐Mediated Oxidative Transformations. *Adv. Synth. Catal.* **2020**, *362*, 184–191.

(33) Speckmeier, E.; Padié, C.; Zeitler, K., Visible Light Mediated Reductive Cleavage of C–O Bonds Accessing α-Substituted Aryl Ketones. *Org. Lett.* **2015**, *17*, 4818–4821.

(34) Wang, Q.; Zhang, X.; Wang, P.; Gao, X.; Zhang, H.; Lei, A., Electrochemical Palladium‐Catalyzed Intramolecular C—H Amination of 2‐Amidobiaryls for Synthesis of Carbazoles. *Chinese J. Chem.* **2021**, *39*, 143–148.

(35) Huang, J.; Sun, N.; Chen, P.; Tang, R.; Li, Q.; Ma, D.; Li, Z., Largely Blue-Shifted Emission through Minor Structural Modifications: Molecular Design, Synthesis, Aggregation-Induced Emission and Deep-Blue OLED Application. *Chem. Commun.* **2014**, *50*, 2136–2138.

(36) Ghosh, I.; Shaikh, R. S.; König, B., Sensitization-Initiated Electron Transfer for Photoredox Catalysis. *Angew. Chem. Int. Ed.* **2017**, *56*, 8544–8549.

(37) Herr, P.; Glaser, F.; Büldt, L. A.; Larsen, C. B.; Wenger, O. S., Long-Lived, Strongly Emissive, and Highly Reducing Excited States in Mo(0) Complexes with Chelating Isocyanides. *J. Am. Chem. Soc.* **2019**, *141*, 14394–14402.

(38) Pellegrin, Y.; Odobel, F., Sacrificial Electron Donor Reagents for Solar Fuel Production. *Comptes Rendus Chim.* **2017**, *20*, 283–295.

(39) Kellett, M. A.; Whitten, D. G.; Gould, I. R.; Bergmark, W. R., Surprising Differences in the Reactivity of Cyanoaromatic Radical Anions Generated by Photoinduced Electron Transfer. *J. Am. Chem. Soc.* **1991**, *113*, 358–359.

(40) Engl, S.; Reiser, O., Making Copper Photocatalysis Even More Robust and Economic: Photoredox Catalysis with $[Cu^{II}(dmp)_2Cl]Cl$. *Eur. J. Org. Chem.* **2020**, *2020*, 1523–1533.

(41) Fayad, R.; Engl, S.; Danilov, E. O.; Hauke, C. E.; Reiser, O.; Castellano, F. N., Direct Evidence of Visible Light-Induced Homolysis in Chlorobis(2,9-dimethyl-1,10-phenanthroline)copper(II). *J. Phys. Chem. Lett.* **2020**, *11*, 5345–5349.

(42) Armaroli, N.; De Cola, L.; Balzani, V.; Sauvage, J.-P.; Dietrich-Buchecker, C. O.; Kern, J.-M., Absorption and Luminescence Properties of 1,10-Phenanthroline, 2,9-Diphenyl-1,10-phenanthroline, 2,9-Dianisyl-1,10-phenanthroline and Their Protonated Forms in Dichloromethane Solution. *J. Chem. Soc. Faraday*

Trans. **1992**, *88*, 553.

(43) Glaser, F.; Kerzig, C.; Wenger, O. S., Sensitization-Initiated Electron Transfer via Upconversion: Mechanism and Photocatalytic Applications. *Chem. Sci.* **2021**, *12*, 9922–9933.

(44) Darmanyan, A. P., Experimental Study of Singlet-Triplet Energy Transfer in Liquid Solutions. *Chem. Phys. Lett.* **1984**, *110*, 89–94.

(45) Montalti, M.; Credi, A.; Prodi, L.; Gandolfi, M. T., *Handbook of Photochemistry*, Third Edit.; CRC Press, 2006.

(46) Pedersen, S. U.; Christensen, T. B.; Thomasen, T.; Daasbjerg, K., New Methods for the Accurate Determination of Extinction and Diffusion Coefficients of Aromatic and Heteroaromatic Radical Anions in *N,N*-Dimethylformamide. *J. Electroanal. Chem.* **1998**, *454*, 123–143.

(47) Beckwith, J. S.; Aster, A.; Vauthey, E., The Excited-State Dynamics of the Radical Anions of Cyanoanthracenes. *Phys. Chem. Chem. Phys.* **2022**, *24*, 568–577.

(48) Blanc, S.; Pigot, T.; Cugnet, C.; Brown, R.; Lacombe, S., A New Cyanoaromatic Photosensitizer vs. 9,10-Dicyanoanthracene: Systematic Comparison of the Photophysical Properties. *Phys. Chem. Chem. Phys.* **2010**, *12*, 11280–11290.

(49) Knorn, M.; Rawner, T.; Czerwieniec, R.; Reiser, O., [Copper(phenanthroline)(bisisonitrile)]$^{+}$ -Complexes for the Visible-Light-Mediated Atom Transfer Radical Addition and Allylation Reactions. *ACS Catal.* **2015**, *5*, 5186–5193.

(50) Pirtsch, M.; Paria, S.; Matsuno, T.; Isobe, H.; Reiser, O., [Cu(dap)$_2$Cl] As an Efficient Visible-Light-Driven Photoredox Catalyst in Carbon-Carbon Bond-Forming Reactions. *Chem. Eur. J.* **2012**, *18*, 7336–7340.

(51) Lazorski, M. S.; Castellano, F. N., Advances in the Light Conversion Properties of Cu(I)-Based Photosensitizers. *Polyhedron* **2014**, *82*, 57–70.

(52) Armaroli, N., Photoactive Mono- and Polynuclear Cu(I)–Phenanthrolines. A Viable Alternative to Ru(II)–Polypyridines? *Chem. Soc. Rev.* **2001**, *30*, 113–124.

(53) Kern, J.-M.; Sauvage, J.-P., Photoassisted C – C Coupling via Electron Transfer to Benzylic Halides by a Bis(di-imine) Copper(I) Complex. *J. Chem. Soc., Chem. Commun.* **1987**, No. 8, 546–548.

(54) Cetin, M. M.; Hodson, R. T.; Hart, C. R.; Cordes, D. B.; Findlater, M.; Casadonte Jr., D. J.; Cozzolino, A. F.; Mayer, M. F., Characterization and Photocatalytic Behavior of 2,9-Di(aryl)-1,10-phenanthroline Copper(I) Complexes. *Dalt. Trans.* **2017**, *46*, 6553–6569.

(55) Kikuchi, K.; Hoshi, M.; Niwa, T.; Takahashi, Y.; Miyashi, T., Heavy-Atom Effects on the Excited Singlet-State Electron-Transfer Reaction. *J. Phys. Chem.* **1991**, *95*, 38–42.

(56) Kim, H.; Kim, H.; Lambert, T. H.; Lin, S., Reductive Electrophotocatalysis: Merging Electricity and Light to Achieve Extreme Reduction Potentials. *J. Am. Chem. Soc.* **2020**, *142*, 2087–2092.

(57) Arias-Rotondo, D. M.; McCusker, J. K., The Photophysics of Photoredox Catalysis: A Roadmap for Catalyst Design. *Chem. Soc. Rev.* **2016**, *45*, 5803–5820.

(58) McMillin, D. R.; Kirchhoff, J. R.; Goodwin, K. V., Exciplex Quenching of Photo-Excitd Copper Complexes. *Coord. Chem. Rev.* **1985**, *64*, 83–92.

(59) Everly, R. M.; McMillin, D. R., Concentration-Dependent Lifetime of $Cu(NN)_2^+$ Systems: Exciplex Quenching from the Ion Pair State. *Photochem. Photobiol.* **1989**, *50*, 711–716.

(60) Mara, M. W.; Fransted, K. A.; Chen, L. X., Interplays of Excited State Structures and Dynamics in Copper(I) Diimine Complexes: Implications and Perspectives. *Coord. Chem. Rev.* **2015**, *282–283*, 2–18.

(61) Mandigma, M. J. P.; Žurauskas, J.; MacGregor, C. I.; Edwards, L. J.; Shahin, A.; D'Heureuse, L.; Yip, P.; Birch, D. J. S.; Gruber, T.; Heilmann, J.; John, M. P.; Barham, J. P., An Organophotocatalytic Late-Stage N–CH_3 Oxidation of Trialkylamines to *N*-Formamides with O_2 in Continuous Flow. *Chem. Sci.* **2022**, *13*, 1912–1924.

(62) Bachilo, S. M.; Weisman, R. B., Determination of Triplet Quantum Yields from Triplet-Triplet Annihilation Fluorescence. *J. Phys. Chem. A* **2000**, *104*, 7713–7714.

(63) Pfund, B.; Steffen, D. M.; Schreier, M. R.; Bertrams, M.-S.; Ye, C.; Börjesson, K.; Wenger, O. S.; Kerzig, C., UV Light Generation and Challenging Photoreactions Enabled by Upconversion in Water. *J. Am. Chem. Soc.* **2020**, *142*, 10468–10476.

(64) Fujita, M.; Ishida, A.; Majima, T.; Takamuku, S., Lifetimes of Radical Anions of Dicyanoanthracene, Phenazine, and Anthraquinone in the Excited State from the Selective Electron-Transfer Quenching. *J. Phys. Chem.* **1996**, *100*, 5382–5387.

(65) Eriksen, J.; Lund, H.; Nyvad, A. I.; Yamato, T.; Mitchell, R. H.; Dingle, T. W.; Williams, R. V.; Mahedevan, R., Electron-Transfer Fluorescence Quenching of Radical Ions. *Acta Chem. Scand.* **1983**, *37b*, 459–466.

(66) Zeman, C. J.; Kim, S.; Zhang, F.; Schanze, K. S., Direct Observation of the Reduction of Aryl Halides by a Photoexcited Perylene Diimide Radical Anion. *J. Am. Chem. Soc.* **2020**, *142*, 2204–2207.

(67) Haimerl, J.; Ghosh, I.; König, B.; Vogelsang, J.; Lupton, J. M., Single-Molecule Photoredox Catalysis. *Chem. Sci.* **2019**, *10*, 681–687.

(68) Wu, S.; Žurauskas, J.; Domański, M.; Hitzfeld, P. S.; Butera, V.; Scott, D. J.; Rehbein, J.; Kumar, A.; Thyrhaug, E.; Hauer, J.; Barham, J. P., Hole-Mediated Photoredox Catalysis: Tris(P-substituted)biarylaminium Radical Cations as Tunable, Precomplexing and Potent Photooxidants. *Org. Chem. Front.* **2021**, *8*, 1132–1142.

(69) Connell, T. U.; Fraser, C. L.; Czyz, M. L.; Smith, Z. M.; Hayne, D. J.; Doeven, E. H.; Agugiaro, J.; Wilson, D. J. D.; Adcock, J. L.; Scully, A. D.; Gómez, D. E.; Barnett, N. W.; Polyzos, A.; Francis, P. S., The Tandem Photoredox Catalysis Mechanism of $[Ir(ppy)_2(dtb\text{-}bpy)]^+$ Enabling Access to Energy Demanding Organic Substrates. *J. Am. Chem. Soc.* **2019**, *141*, 17646–17658.

(70) Marchini, M.; Gualandi, A.; Mengozzi, L.; Franchi, P.; Lucarini, M.; Cozzi, P. G.; Balzani, V.; Ceroni, P., Mechanistic Insights into Two-Photon-Driven Photocatalysis in Organic Synthesis. *Phys. Chem. Chem.*

Phys. **2018**, *20*, 8071–8076.

(71) Pause, L.; Robert, M.; Savéant, J. M., Can Single-Electron Transfer Break an Aromatic Carbon - Heteroatom Bond in One Step? A Novel Example of Transition between Stepwise and Concerted Mechanisms in the Reduction of Aromatic Iodides. *J. Am. Chem. Soc.* **1999**, *121*, 7158–7159.

(72) Tian, X.; Karl, T. A.; Reiter, S.; Yakubov, S.; de Vivie-Riedle, R.; König, B.; Barham, J. P., Electro-Mediated PhotoRedox Catalysis for Selective C(sp^3)–O Cleavages of Phosphinated Alcohols to Carbanions. *Angew. Chem. Int. Ed.* **2021**, *60*, 20817–20825.

(73) Sandford, C.; Edwards, M. A.; Klunder, K. J.; Hickey, D. P.; Li, M.; Barman, K.; Sigman, M. S.; White, H. S.; Minteer, S. D., A Synthetic Chemist's Guide to Electroanalytical Tools for Studying Reaction Mechanisms. *Chem. Sci.* **2019**, *10*, 6404–6422.

(74) Elgrishi, N.; Rountree, K. J.; McCarthy, B. D.; Rountree, E. S.; Eisenhart, T. T.; Dempsey, J. L., A Practical Beginner's Guide to Cyclic Voltammetry. *J. Chem. Educ.* **2018**, *95*, 197–206.

(75) Hasegawa, E.; Tanaka, T.; Izumiya, N.; Kiuchi, T.; Ooe, Y.; Iwamoto, H.; Takizawa, S. Y.; Murata, S., Protocol for Visible-Light-Promoted Desulfonylation Reactions Utilizing Catalytic Benzimidazolium Aryloxide Betaines and Stoichiometric Hydride Donor Reagents. *J. Org. Chem.* **2020**, *85*, 4344–4353.

(76) Hamada, T.; Nishida, A.; Yonemitsu, O., Selective Removal of Electron-Accepting p-Toluene- and Naphthalenesulfonyl Protecting Groups for Amino Function via Photoinduced Donor-Acceptor Ion Pairs with Electron-Donating Aromatics. *J. Am. Chem. Soc.* **1986**, *108*, 140–145.
